# Optimizing the role and functions of CHWs in service of a people-centred community health system in sub-Saharan Africa. A realist synthesis

**DOI:** 10.1016/j.ssmhs.2025.100089

**Published:** 2025-12

**Authors:** Usangiphile E. Buthelezi, André J. van Rensburg, Mosa Moshabela, Zamasomi Luvuno, Tasneem Kathree, Arvin Bhana, Inge Petersen

**Affiliations:** a Centre for Research in Health Systems, University of KwaZulu Natal, School of Nursing and Public Health, University of KwaZulu-Natal, South Africa; b Centre for Research in Health Systems, University of KwaZulu Natal, South Africa; c University of Cape Town, South Africa, School of Nursing and Public Health, University of KwaZulu-Natal, South Africa; d Centre for Research in Health Systems, University of KwaZulu Natal, South Africa; e Centre for Research in Health systems, School of Nursing and Public Health, University of KwaZulu-Natal, South Africa; f Centre for Research in Health Systems, School of Nursing and Public Health, UKZN, Health Systems Research Unit, South African Medical Research Council, Durban, South Africa; g Institute of Global Health, University College London, London, United Kingdom; h Centre for Research in Health systems, University of KwaZulu Natal, South Africa

**Keywords:** Community Health Workers, Realist Evaluation, Realist Review, Integrated People-Centred Health Services, Initial Programme Theories, Retroduction

## Abstract

**Introduction:**

The role of Community Health Workers (CHWs) in sub-Saharan Africa is critical to achieve people-centred health systems. Despite a large evidence base, there is a dearth of knowledge regarding the contextual factors and mechanisms that shape CHW performance in providing people-centred care. This study aims to map out conditions that enable people-centred care by CHWs in sub-Saharan Africa by identifying the key mechanisms and contextual factors.

**Methodology:**

A realist synthesis approach was employed to explore how, why, and under what conditions CHW interventions lead to desired outcomes for people-centred care. A systematic review of the literature was undertaken from 2014 to 2024, focusing on studies conducted in sub-Saharan Africa. The review followed a six-phase process, including the development of the initial programme theory, search for evidence, evidence review and quality appraisal, data extraction, data synthesis and analysis, and refinement of the programme theory, followed by formulation of context-mechanism-outcome (CMO) configurations.

**Results:**

This synthesis included 36 studies from 14 sub-Saharan African countries. In total, 101 CMO configurations were identified and condensed into 17 preliminary configurations. Specific contexts and mechanisms emerged that influence outcomes related to CHW programmes. The study identified trust, motivation, and adaptive leadership as fundamental meta-mechanisms that challenge the siloed structure of the IPCHS framework, emphasizing the need for greater flexibility to capture interactions across different strategies of the framework.

**Conclusion:**

This study demonstrates that integrating CHWs into formal systems, aligning CHW-specific interventions with community-based initiatives, establishing intersectoral partnerships, and updating the IPCHS framework to incorporate adaptive leadership and feedback mechanisms can enhance the delivery of people-centred care.

## 1 Introduction

People’s health and life expectancy are unequal between different countries and within countries. It is reported that over one billion of the world population do not have access to health care and that satisfaction with health services remains low globally (World Health Organization, 2015). Health care remains fragmented and inefficient, especially in low and middle-income countries including the sub-Saharan region (Azevedo, 2017). A hospicentric and “silo” curative approach to health care hinders the health system’s ability to respond to people’s health demands and challenges the capacity of health systems to provide universal, equitable, high-quality, and financially sustainable care (World Health Organization, 2015, Freijser et al., 2023). Despite much progress in health system reform over past decades, service fragmentation, a neglect of population needs, and lack of empowerment persists, especially in the context of increasing health system shocks from infectious disease outbreaks and a rapidly increasing non-communicable disease (NCD) burden (Azevedo, 2017, Ndumwa et al.,). To redress these problems and achieve universal health coverage, the World Health Organisation (WHO) has suggested a people-centredness approach for primary health care (PHC) and developed strategies to accomplish this goal (World Health Assembly 69 69, 2016).

People-centred care (PCC) embodies integrated care that is centred around people’s needs, aligning closely with population health management approaches that aim to improve outcomes across communities (Farmanova et al., 2019). It deviates from the conventional approach to healthcare, which is centred around the treatment of illnesses and diseases in an episodic approach, regarding patients as passive recipients of care. Founded upon the principles of person-centred care, it presents a departure from the biomedical and paternalistic healthcare models of the past (Håkansson Eklund et al., 2019), and advocates for a paradigm shift that places individuals squarely at the helm of their healthcare journey (World Health Organization, 2024). It emphasizes recognizing individuals and actively prioritizes their unique needs, preferences, voices and participation in decision-making processes regarding their health (World Health Organization, 2024). Furthermore, in addition to treating a person’s medical condition, PCC places emphasis on providing support for the individual by incorporating communities and families into the care cycle (Khatri et al., 2023). Furthermore, it includes a focus on empowering communities and individuals to take control of their health.

The WHO Framework on Integrated People-Centred Health Services (IPCHS), delineates strategies to: engage and empower people and communities, strengthening governance and accountability, reorienting the model of care, coordinating services within and across sectors, and creating an enabling environment (World Health Assembly 69 69, 2016). Integrated care and people-centred care complement each other, working together to improve both healthcare outcomes and patient experiences by focusing on the individual, their family, and the community (World Health Assembly 69 69, 2016). Community Health Workers (CHWs) are well placed to assist with the implementation of these IPCHs strategies. Their functions such as care coordination, resource linking, providing social support, health coaching, health assessment, case management, health literacy support and targeted health education (Hartzler et al., 2018), transcend the provision of healthcare embodying the fundamental principles of people-centred care. Through their direct engagement with communities, CHWs serve as promoters for change, forging meaningful connections and delivering personalized care that resonates with the diverse backgrounds and beliefs of individuals they serve (Hartzler et al., 2018, Brownstein et al., 2011, Schaaf et al., 2020). Furthermore, as frontline workers who come from the same communities they support, CHWs share the same language, cultural norms, and social values, which allows them to deliver services that are both linguistically and culturally relevant, while also helping people navigate complex health systems (Bourgeault et al., 2025). However, the effectiveness of CHW programmes is not a uniform phenomenon and is profoundly influenced by a myriad of intricate contextual factors and underlying mechanisms (Schneider, 2019).

The absence of standardized training together with unrecognized professional status creates role ambiguity which diminishes CHWs’ credibility in healthcare systems (Ballard et al., 2020, Kane et al., 2016). CHWs face multiple resource constraints which include poor renumeration and irregular supervision and insufficient logistical support leading to high employee turnover and burnout (Kane et al., 2016, Perry et al., 2021). The work of CHWs becomes more complex because they must overcome both community mistrust and resistance to health interventions while maintaining cultural sensitivity (LeBan et al., 2021 Oct 12). The fragmented nature of health systems further creates difficulties because CHWs need to link patients with different services yet they lack proper referral systems and data integration capabilities (LeBan et al., 2021, Tseng et al., 2019). In addition, structural inequities, including gender disparities (as many CHWs are women in undervalued roles) and geographic isolation in rural areas, further exacerbate these difficulties (Steege et al., 2020, Malatji et al., 2024). Although the responsibilities, challenges, and effectiveness of CHWs in diverse healthcare settings have been studied previously (Hartzler et al., 2018, Mm, 2020, Glenton et al., 2021), using a realist evaluation framework can lead to a more comprehensive understanding of their roles and functions by unpacking the underlying contextual factors and mechanisms.

Realist evaluation, which aims to elucidate the complexities of how, why, and under what conditions outcomes emerge, provides a valuable framework for uncovering the generative mechanisms steering the roles and performance of CHWs (Greenhalgh and Manzano, 2022, Pawson et al., 2005). This evaluation approach has been widely utilized in healthcare research (McNeil et al., 2016, Mills et al., 2014, Mukumbang et al., 2016). Although there have been many reviews of CHW programmes, there exists a dearth of research evidence investigating the convergence of realist evaluation, people-centred care, and the roles and functions of CHWs.

The aim of this realist review was to generate a deeper understanding of the underlying generative mechanisms informing CHWs’ performance in alignment with the principles of people-centred care. Specifically, the review sought to: (World Health Organization, 2015) identify the mechanisms through which CHW roles and responsibilities can be enhanced in support of IPCHS in sub-Saharan Africa; (Azevedo, 2017) examine the contextual factors that enable or constrain these mechanisms; and (Freijser et al., 2023) explore how key mechanisms and contextual elements within CHW activities, programmes, and interventions interact to produce outcomes aligned with IPCHS in sub-Saharan Africa.

## 2 Methodology

### 2.1 Design

This study is grounded in scientific realism, which posits that both material and social contexts exert tangible influences on the outcomes of interventions by shaping the mechanisms through which these outcomes are achieved, thereby emphasizing the pursuit of a deeper comprehension of the factors underlying the change processes (Mukumbang et al., 2016)**.** This means that the physical environment, available resources, cultural norms, and social relationships all play crucial roles in determining how and why certain interventions succeed or fail.

This realist review was conducted in accordance with Pawson and Tilley's theory-driven methodology, with the goal of understanding why particular outcomes occur, for whom, under what conditions, and to what extent (Fleming et al., 2023). As outlined by Pawson et al. (2005) (Pawson et al., 2005), the initiation of a realist review involves delineating the scope of the review and formulating preliminary hypotheses, termed as an initial program theory (IPT). A program theory is characterized as "a set of explicit or implicit assumptions established by stakeholders about what action is required to solve a social, educational, or health problem and why the problem will respond to this activity" (Goicolea et al., 2015)**.** A program theory can undergo examination and further refinement, underscoring the notion that a realist review begins and concludes with a programme theory. Thus, the IPT functioned as a guiding roadmap for this realist synthesis, providing a structured framework for data synthesis and laying the foundation for subsequent refinement through literature (Mukumbang et al., 2016).

A realist review methodology allows for a wide range of evidence, including published peer reviewed literature, grey literature, and stakeholder input at various stages of the review as evidence (Power et al., 2019). It also focuses on causality, attempting to discover circumstances in which an intervention or action might activate a mechanism (M) to create a given outcome (O) under specified contextual conditions (C) (Dalkin et al., 2015). This is accomplished through the development of context-mechanism-outcome configurations (CMOCs), which play an important role in the analysis and theory-building process (Pawson et al., 2005, Pawson and Tilley, 1997). Mechanisms are typically seen as the critical link between context and outcome (Dalkin et al., 2015).

**Fig. 1:**
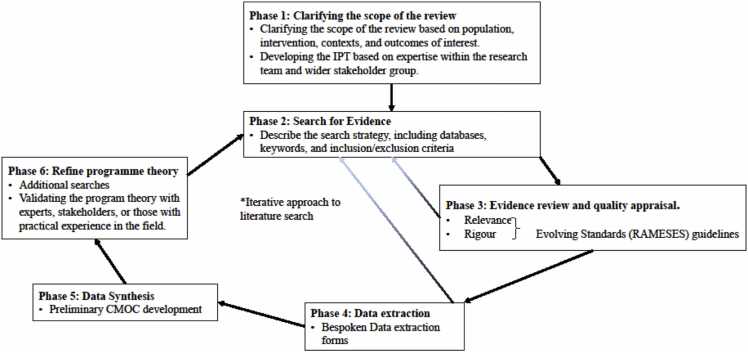
Flow diagram of the realist review (adapted from Pawson et al. 2005; Wong et al. 2015) (Pawson et al., 2005, Wong et al., 2015).

Realist evaluation is particularly well-suited for evaluating complex interventions as it focuses on understanding the underlying mechanisms and contextual factors that influence the outcomes of the intervention, such as those involving CHWs. This approach enhances the understanding of the values and norms of CHWs and communities, as well as the power dynamics and relationships within the health system (Kok et al., 2015a). Additionally, the realist philosophy supports the idea of considering the context in which CHW programs and services operate to understand how specific outcomes are achieved (Kok et al., 2015a). This review used a 6-phase approach to examine existing literature (Fig. 1).

### 2.2 Phase 1: Clarifying the scope of the review and development of the initial programme theory

The aim of this realist synthesis was to offer insights into enhancing CHW programmes to align more with the principles of people-centred care. In the initial phase of this realist review, program theories related to CHWs and their roles and functions in delivering people-centred care were developed. We utilized the IPCHS as a guide to develop the initial program theories. This framework offers strategies, policy options, and interventions aimed at promoting people-centred care. Furthermore, preliminary literature searches were conducted to enhance our understanding of potential theories, thereby enabling its articulation and advancement. The program theories established the framework for subsequent searches and synthesis stages, with the aim of substantiating, refining, or refuting each established initial programme theory.

Our initial program theory was developed in consultation with a team of researchers and experts in the fields of realist evaluation, people-centred care, and CHW programmes, ensuring diverse perspectives. This was to ensure that we gained insight from “real life” field experience or practice, and research perspective through literature.

To package the IPTs, we used the "If-Then" statements: Where IF represents context (C), Then represents mechanisms (M) leading to outcome (O) (Flynn et al., 2020).

**Fig. 2:**
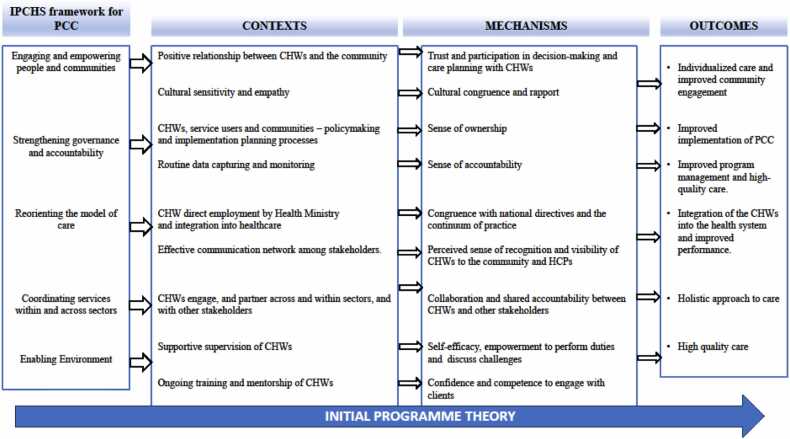
Schematic representation of the Initial Programme Theory.

Using "If-Then" statements enabled a methodical examination of conceptual ideas and assumptions about how interventions work, for whom, and in what conditions. This approach streamlined evidence testing, ensuring a thorough analysis of the uncovered evidence as the review advanced (Bunn et al., 2017). The "If-Then" statements provided a detailed narrative representation of the CMOs through rich descriptions (appendix A), followed by the visualization of the CMOs through a diagram for a clear and concise presentation (Fig. 2).

**Table 1:** Search terms for PubMed, EBSCOhost, Science direct, Scopus, and Web of Science.

**Term 1**	**Term 2**
Community Healthcare Worker	Sub-Saharan countries
Community Health Worker	
CHWs	

*Other terms that fell under the umbrella term of community health worker were included in this review

### 2.3 Phase 2: Search for Evidence

The literature search for this realist review encompassed peer-reviewed primary research articles derived from intervention studies. Only intervention studies were considered eligible for inclusion. A systematic search of online databases, including PubMed, EBSCOhost, ScienceDirect, Scopus, and Web of Science, was conducted using Boolean search terms (see Appendix B) to identify relevant literature on CHWs' roles and functions in service of people-centred care. Additionally, the following steps were undertaken to identify relevant evidence from a range of sources for inclusion in the review:

➣ Reference lists from primary studies and review articles (snowballing).
➣ Input from the review team to uncover other relevant publications.
➣ Input from the research consisting of experts in the field.

Inclusion and exclusion criteria for the identified literature were based on its publication date between 2014 and 2024. The selection of this time period was motivated by the author team’s intention to examine publications from the previous decade and the 2014 publication of the policy framework for people-centred care by the World Health Organization (World Health Organization, 2024). Additional criteria for inclusion and exclusion are outlined below (Table 2).

**Table 2:** Inclusion and exclusion criteria.

**Inclusion Criteria**	**Exclusion Criteria**
1. Studies conducted in sub-Saharan African countries	Studies conducted outside sub-Saharan countries
2. Studies that include CHWs and terms that encompass them in the sub-Saharan African countries’ context as listed in table 3. If other terms that encompass CHWs during the search for evidence arose, they were included.	Source not written in English
3. Studies that address the roles and functions of CHWs to promote people-centred care.	Studies that do not include CHWs.
4. Intervention studies	Studies that are not interventional

The systematic literature search strategy was initially piloted to ascertain the relevance of selected databases, the scope of covered literature, and key search terms used. Pilot testing was conducted by the two independent reviewers (UB and AVR) before screening the titles, abstracts, and full texts. A subset of articles was selected for initial screening to assess consistency in the inclusion process. The preliminary agreement among screeners was conducted using Rayyan (Rayyan, 2021), a web-based AI-powered tool. During the screening process, blinding was applied and later removed at the end of screening. The software then revealed which articles each reviewer included or excluded, highlighted disagreements, and indicated cases where reviewers were uncertain. Any disagreements were resolved through discussions via zoom meetings before proceeding with including articles. To ensure consistency in article inclusion, any disagreements were resolved by involving a third reviewer (IP), who referenced both the protocol and the IPT.

Search results were managed using Endnote X20 (The EndNote Team, 2013) and Web Rayyan (Rayyan, 2021) for intelligent systematic review. This ensured real time correspondence between reviewers, including removal of duplicates, locating full-text articles and screening. The study selection process was summarized using the Preferred Reporting Items for Systematic Reviews and Meta-Analysis (PRISMA) (Arksey and O’Malley, 2005).

### 2.4 Phase 3: Evidence review and quality appraisal

The titles and abstracts of the identified records, along with the full-text papers selected in stage two, underwent independent screening by two reviewers (UB and AVR). The objective was to identify records that provided evidence illuminating one or more aspects of the program theories identified in stage one. Depending on the number of papers retained, the review team considered further adjustments to the review scope. Additionally, new, or revised selection criteria were determined as necessary for additional searches. The decision to incorporate these additional studies depended on their potential to enhance the refinement of program theories.

We employed systematic techniques for screening and selecting studies, following the guidance provided by the Preferred Reporting Items for Systematic Reviews and Meta-Analyses (RAMESES) (Wong et al., 2013). According to the RAMESES guidance, the assessment of any data section was based on two criteria:

1. Relevance – assessing its potential contribution to theory building and/or testing.
2. Rigour – evaluating the credibility and trustworthiness of the method used to generate that specific piece of data.

Relevance was assessed based on criteria from Pearson et al., 2012; 2015 (Pearson et al., 2012, Pearson et al., 2015, Brennan et al., 2017), while rigour was evaluated using criteria from Ohly et al., 2017 (Ohly et al., 2017) as showed in the data extraction form (Appendix C). Test for relevance and rigor was done by same reviewers responsible for screening and selecting studies (UB and AVR), to maintain consistency in the study inclusion/exclusion process.

In the context of realist reviews, the criteria for inclusion are focused on evaluating the reported evidence's relevance and rigor, with the intention of facilitating significant contributions to the development of CMO configurations (Kantilal et al., 2020). The studies were chosen in accordance with their applicability in advancing the formulation or testing of program theories. Furthermore, proponents of realism propose that the conventional hierarchy of evidence is unsuitable for the execution of realist assessments or evaluations (Kantilal et al., 2020). This suggests that studies that may have lower methodological quality (according to the conventional hierarchy of evidence), can still provide valuable insights that contribute to the refinement of the programme theory. Therefore, we considered such evidence not in terms of its methodological limitations alone, but in relation to its capacity to inform and advance theoretical development.

#### 2.4.1 Phase 4: Data extraction

In phase three, studies that met the test for relevance and rigor had their data extracted onto bespoke data extraction forms by two independent reviewers (UB and AVR) (Appendix D). This facilitated the compilation of evidence on context, mechanism, and outcomes. The extracted data underwent organization into categories, delineating information concerning CHW roles, contextual factors impacting their work, mechanisms prompting change, and achieved outcomes. Additionally, patterns and variations across diverse contexts were identified. This exploration allowed for an enhanced synthesis, incorporating contextual nuances regarding the operation of CHWs across various community health contexts.

### 2.5 Phase 5: Data synthesis and analysis

After completing the data extraction from the included studies for analysis, the retrieved data served as the cornerstone for investigating the relationships between mechanisms, context, and outcomes using retroductive methods (Jagosh, 2020). Retroductive analysis was employed to uncover mechanisms or hidden causal processes, facilitating the generation of initial program theories encapsulated in the form of CMOCs. We used retrodiction to assess, compare, and elucidate observable patterns in the data, actively seeking and analysing information not accounted for by our IPT. Two independent researchers (UE and AVR) analysed each article to unpack CMOs (Appendix E). The arising CMOs were further analysed to generate themes across the articles.

Throughout this process, the research team engaged in discussions to explore potential explanations and strategies for refining and revising CMOCs. The data from the arising CMOs was further categorized according to the IPCHS as a framework for analysis used in this study. This included organizing the findings by five strategies: Engaging and empowering people and communities, strengthening governance and accountability, reorienting the model of care, coordinating services within and across sectors, and creating an enabling environment.

## 3 Results

### 3.1 Description of the included studies

A search yielded 7372 records from all databases (Fig. 2). We removed 3369 duplicated records. Then, studies were screened for relevance based on title and abstract, whereby 4107 were excluded, leaving 213 studies for full-text screening. A further 168 studies were excluded after the full-text screening with reasons, leaving 44 studies being assessed for eligibility and 8 studies being excluded. Ultimately, there were 36 studies that were included in the final review (country-level counts exceed this due to multi-country studies). These studies were from, South Africa (n=12), Nigeria (n=2), Zambia (n=3), Madagascar (n=2), Uganda (n=3), Ghana (n=2), Democratic Republic of Congo (DRC) (n=1), Mozambique (n=1), Zimbabwe (n=4), Tanzania (n=3), Kenya (n=2), Malawi (n=1), Cameroon (n=1) and Liberia (n=1). Seven studies were quantitative in nature, twelve qualitative, sixteen mixed methods, and one realist evaluation study (Appendix F). Following the initial program theory (Fig. 2), findings are presented using the WHO IPCHS framework for people-centred care. Out of the 36 studies included, 21 studies contributed to Engaging and empowering people and communities, 17 to strengthening governance and accountability, 10 to reorienting the model of care, 14 to coordinating services within and across sectors, and 9 to creating an enabling environment. However, it is important to note that multiple strategies of the WHO IPCHS framework for people-centred care may exist within one article and across different articles.

**Fig. 3:**
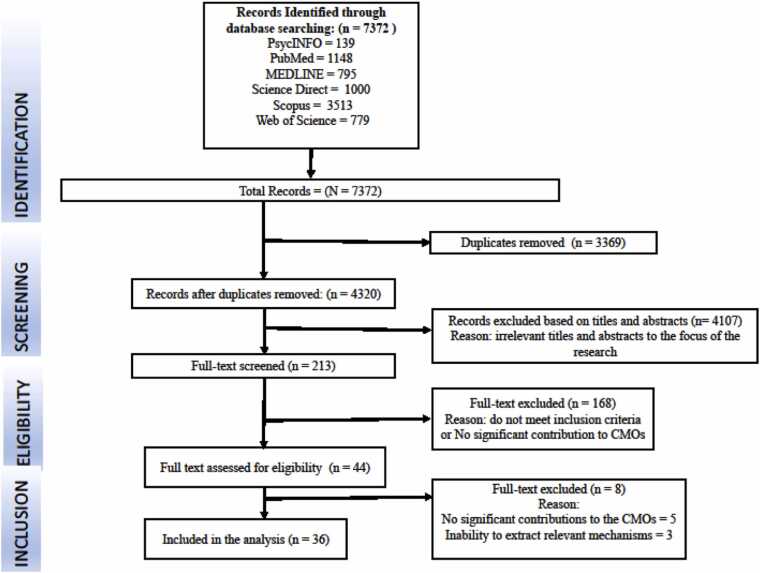
PRISMA flow chart showing the selection of studies for the review.

**Fig. 4:**
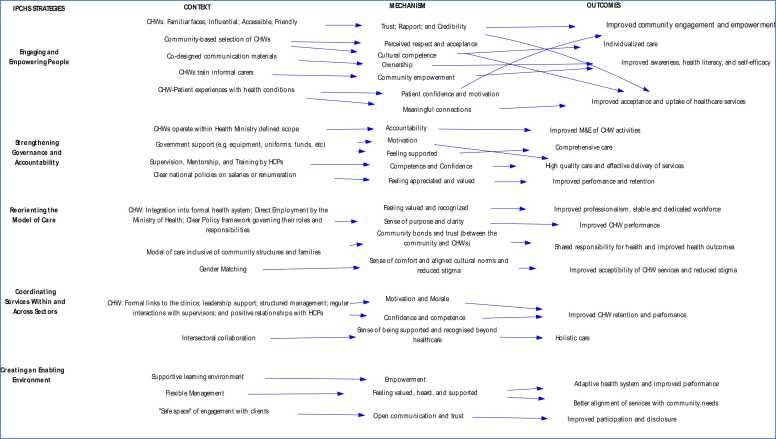
Schematic representation of the refined programme theory for enhancing CHW roles and responsibilities in service of IPCHS.

In the final results, 101 CMO configurations were generated through rigorous coding, reviewing, and rereading of the included articles (Appendix E). These CMOs were condensed to 17 preliminary CMOs in 3 meetings with the research team using the If-then statements (see Appendix J). This process was done through combining or deduplicating CMOs with very similar content and packaging CMOs according to the IPCHS framework strategies. Furthermore, the CMOs and the if-then propositions, were used to develop three visualizations that capture the strategies for optimizing the roles and functions of CHWs using the IPCHS framework as a guide or “backdrop” (Fig. 4). The if-then propositions were used to narratively explain the theories through thick descriptions using thematic analysis (Appendix J).

### 3.2 Engaging and empowering people and communities

Empowering and engaging individuals involves equipping them with the necessary resources, skills, and opportunities to effectively advocate for a reformed health system and to be active participants in using health services. This strategy aims to activate community and individual resources to enable individuals to make informed health choices and promote community involvement in creating healthy environments. Moreover, it underscores the significance of equipping informal caregivers with the requisite knowledge and skills to improve their effectiveness and aid them in fulfilling their duties. Empowering and engaging individuals also involves ensuring that underserved and marginalized groups have universal access to and benefit from high-quality services that are co-produced to meet their specific needs (World Health Assembly 69 69, 2016). In this study, twenty-one articles contributed to the strategy of engaging and empowering people and families (Pollard et al., 2022, Adam et al., 2014, Stansert Katzen et al., 2021, le Roux et al., 2015, Murphy et al., 2021, Goudge et al., 2023, Malatji et al., 2022, Yuh et al., 2023, Olakkengil et al., 2024, Soepnel et al., 2024a, Abbey et al., 2014, Abbey et al., 2015, D’Ambruoso et al., 2023, Jenson et al., 2018, Rogers et al., 2023, Mendin et al., 2023, Busza et al., 2018, Dziva Chikwari et al., 2018, Hayward et al., 2024, Youngui et al., 2024). Out of these 21 articles, five CMOs were developed (Fig. 4).

These CMOs fell under the following thematic areas: building trust and engagement through community integration, empowering communities through health education and skills training, strengthening community trust and healthcare effectiveness through collaborative communication, community-centred selection of CHWs, and empathy and personalized care through CHW-patient related personal experiences. There were seven mechanisms that were found under the strategy of engaging and empowering people and families, which are: (World Health Organization, 2015) trust, rapport, and credibility; (Azevedo, 2017) sense of ownership; (Freijser et al., 2023) community empowerment; (Ndumwa et al.) meaningful connections; (World Health Assembly 69 69, 2016) patient confidence and motivation; (Farmanova et al., 2019) respect (CHWs feeling respected and accepted); and (Håkansson Eklund et al., 2019) cultural competency.

Trust and rapport between CHWs and the community were observed in South Africa, where community members often approached CHWs informally to seek health-related information, indicating a level of trust and rapport between them. This shows that CHWs built positive relationships with community members through their availability, approachability, and the trust that community members placed in them to seek health-related information and support (Malatji et al., 2022). In Cameroon, a study by Yuh et al. (2023) demonstrated that building trust between community health workers and families of people with disabilities was crucial for the success of the intervention (Yuh et al., 2023). Trust allowed for open communication, collaboration, and acceptance of recommendations, fostering a positive environment for change. Furthermore, establishing a strong rapport and trust between community health workers and families facilitated effective communication and the implementation of recommended practices (Yuh et al., 2023).

Abbey et al. (2015) found that involving the community in creating communication materials for treating fever in children under 5 improved understanding and acceptance due to a sense of ownership and involvement in care decisions (Abbey et al., 2015). Furthermore, Adam et al. (2014) showed that providing training and education to the community (informal caregivers) on delivering health messages and educating women about maternal and newborn care results in empowerment, which includes increased knowledge, awareness, improved health literacy, enhanced self-confidence, and positive changes in behaviour towards maternal and newborn care (Adam et al., 2014). These findings underscore the importance of community involvement and education in enhancing health outcomes.

A study by Ferrand et al. (2017) found that CHWs who have personal experiences related to patient conditions either through their own lives or within their communities showed empathy and developed meaningful connections, fostering increasing confidence and motivation for the patients to engage in care, resulting in reduced stigma and better engagement with CHWs (Ferrand et al., 2017). The selection of CHWs using a community-based participatory process was shown by studies from different countries, including South Africa, Liberia, Kenya, and Ghana, to foster respect and acceptance, cultural competency, and understanding of local needs, leading to increased adoption of healthier behaviours, individualized care, and greater engagement with CHWs for support and adherence to treatment plans (Adam et al., 2014, Stansert Katzen et al., 2021, le Roux et al., 2015, Abbey et al., 2014, Rogers et al., 2023, Mendin et al., 2023).

### 3.3 Strengthening governance and accountability

**Fig. 5:**
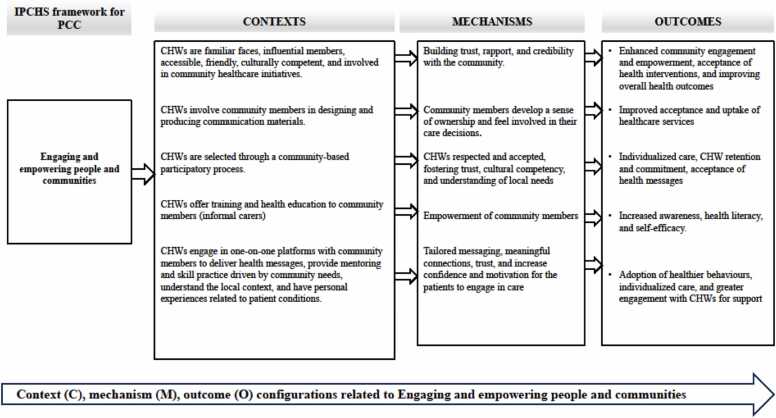
Context, Mechanisms, and Outcome configurations (CMOCs) for engaging and empowering people and communities.

Enhancing governance entails the adoption of a participatory approach that encompasses all facets of the healthcare system, spanning from policy formulation to clinical intervention. This approach ensures that decision-making and performance evaluation processes are both transparent and inclusive. The involvement of policymakers, managers, providers, and users in a collaborative accountability system, with customized incentives to prioritize the needs and wellbeing of individuals, enhances this approach (World Health Organization, 2015). Seventeen articles elaborated on strengthening governance and accountability strategies. Fig. 5 depicts key contextual factors such as CHWs operating within the scope of work defined by the Ministry of Health, receiving government support for community-based tasks, regular mentorship, supervision, and training by health professionals, and contrasts in salaries and support for CHWs. These contextual factors interact with some of the mechanisms identified in the articles to produce outcomes such as improved monitoring and evaluation of CHW activities, comprehensive care, improved retention, enhanced performance, high-quality care, and poor performance and delivery of services to the community. Six mechanisms were found in the strengthening governance and accountability strategy: (World Health Organization, 2015) sense of accountability, (Azevedo, 2017) motivation, (Freijser et al., 2023) feeling supported, (Ndumwa et al.) competence and confidence, (World Health Assembly 69 69, 2016) feeling demotivated and reluctant to take on new tasks, and (Farmanova et al., 2019) feeling appreciated and valued.

In Kenya, where CHWs operate within the scope of work defined by the Ministry of Health, there was a sense of responsibility and accountability to deliver services aligned with the national health priorities and guidelines, leading to a more effective implementation of the healthcare program and improved monitoring and evaluation of CHW activities (Adam et al., 2014). Furthermore, the activities of CHWs gained credibility and trust within the community due to their recognition and support by the Ministry of Health. In studies conducted in Ghana and Nigeria, where CHWs received support from the government to undertake their community-based tasks (e.g., equipment, stationery, uniforms, or funds for transport or communication), CHWs felt motivated to perform their roles and functions (Abbey et al., 2014, Adesoro et al., 2021). Therefore, they were able to provide comprehensive care that covers a broader range of health needs.

A number of studies elaborated on mechanisms such as feeling supported, motivated, accountable, confident, and competent for CHWs to perform their duties and how these interacted with the context where there is regular mentorship, supervision, support, and ongoing intensive training of CHWs by health service professionals and government officials (Tseng et al., 2019, le Roux et al., 2015, Goudge et al., 2023, Malatji et al., 2022, Yuh et al., 2023, Busza et al., 2018, Dziva Chikwari et al., 2018, Adesoro et al., 2021, Enguita-Fernàndez et al., 2021, Goudge et al., 2020, Ndaba et al., 2019, Viljoen et al., 2021a, Wanduru et al., 2016, Razafinjato et al., 2024). In Nigeria, training non-clinical CHWs known as Community-Oriented Resource Persons (CORPs) empowered them to provide treatment for severe acute malnutrition (SAM) and improved their competency in treating SAM cases. Furthermore, training provided CORPs with the necessary knowledge and skills to assess, identify, and treat both severe and uncomplicated SAM cases (Adesoro et al., 2021). A study by Goudge et al. (2023) found that the roving nurse mentor intervention helped and trained CHWs and their supervisors to learn and practice new skills (Goudge et al., 2023). It also helped them deal with their fears of failing and set up operational systems to make CHWs' work more efficient, which led to better service provision (Goudge et al., 2023, Malatji et al., 2022). A study conducted in Zimbabwe to enhance testing and improve treatment of HIV in children demonstrated that intensive supervision and mentoring were critical in ensuring CHWs' long-term satisfaction and improving their performance, and that refresher training helped CHWs deliver the intervention effectively (Busza et al., 2018).

In Uganda, Wanduru et al. (2016) found that regular meetings with supervisors were positively associated with better CHW performance, suggesting that ongoing support and supervision can motivate CHWs and enhance their job satisfaction (Wanduru et al., 2016). Furthermore, CHWs who had met with their supervisors in the previous month were more likely to have better performance, indicating that regular interactions and recognition from supervisors improved CHWs' performance (Wanduru et al., 2016). Another study by Tseng et al. (2019) found that effective supervision led to increased motivation, job satisfaction, and engagement among CHWs, resulting in improved performance and integration into the health system. Furthermore, it is demonstrated that senior supervisors played a crucial role in guiding and mentoring CHWs, building relationships, and passing down knowledge to junior supervisors, which enhanced their skills and confidence. Effective supervision resolved frustrations, working condition issues, and prevented demotivation and passive protests among CHWs (Tseng et al., 2019). In Madagascar, it was shown that regular supervision and quality training sessions for CHWs were vital for ensuring competence and confidence in service delivery (Razafinjato et al., 2024). In addition, Dziva Chikwari et al. (2018) demonstrated that extensive training, ongoing mentorship, and support through monthly supervisory meetings ensured CHWs were well-equipped to deliver the HIV treatment intervention effectively in Zimbabwe, leading to positive outcomes (Dziva Chikwari et al., 2018).

Two studies from South Africa and Zimbabwe reported that in contexts where CHWs faced contextual challenges such as late payment of salaries, poor remuneration, and dissatisfaction with their working conditions, they felt demotivated and reluctant to take on new tasks and to perform their roles and responsibilities, resulting in poor performance, job satisfaction, and the delivery of services to the community (Busza et al., 2018, Klingberg et al., 2021). On the other hand, three studies from Kenya, Madagascar, and South Africa reported that CHWs felt appreciated, valued, and motivated to carry out their responsibilities effectively in the context where there were clear national policies that ensured fair and consistent salaries and support for CHWs (Goudge et al., 2023, Malatji et al., 2022, Rogers et al., 2023, Razafinjato et al., 2024).

### 3.4 Reorienting the model of care

**Fig. 6:**
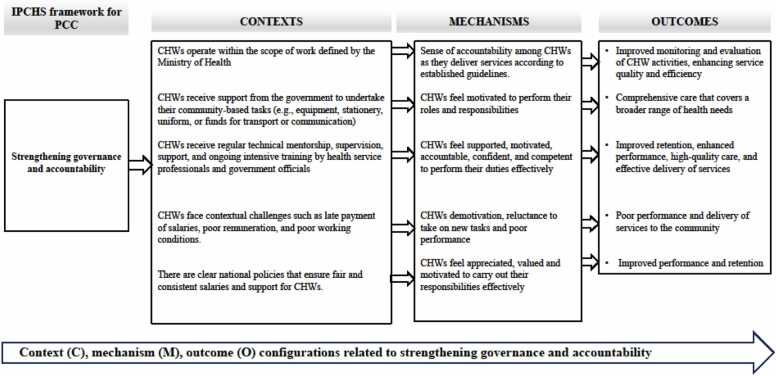
Context, Mechanisms, and Outcome configurations (CMOCs) for strengthening governance and accountability.

The process of reorienting the model of care entails implementing innovative strategies to enhance the delivery of healthcare services, with a particular focus on primary and community care services and the collaborative involvement of individuals in promoting health. This involves transitioning from inpatient to outpatient and ambulatory care, as well as from curative to preventive care. It calls for investing in a holistic and comprehensive approach to care, which includes strategies for promoting health and preventing illness in order to improve people's overall health and well-being. Furthermore, it acknowledges and accommodates gender and cultural preferences when designing and implementing health services (World Health Assembly 69 69, 2016). Ten articles discussed reorienting the model of care strategy. Fig. 6 shows the common contextual factors seen in these articles, such as CHW integration into the formal health system, integrating community support structures and families into the health system, and gender matching into the design and operation of health services (Murphy et al., 2021, Goudge et al., 2023, Malatji et al., 2022, Yuh et al., 2023, Hayward et al., 2024, Youngui et al., 2024, Enguita-Fernàndez et al., 2021, Ndaba et al., 2019, Razafinjato et al., 2024, Feldhaus et al., 2015).

These interact with identified mechanisms in the studies, leading to outcomes like enhanced professionalism, workforce stability, improved performance, health outcomes, and CHW service acceptability. Five mechanisms were identified in the reorienting the model of care strategy, which were, (World Health Organization, 2015) feeling valued and recognized, (Azevedo, 2017) sense of purpose and clarity, (Freijser et al., 2023) trust, (Ndumwa et al.) community bonds, and (Ndumwa et al.) sense of comfort and reduced stigma. CHWs’ direct employment by the Ministry of health was elaborated in a study by Malatji et al. (2022) and Goudge et. al (2023) as a contributing factor that led to feeling valued and recognized as contributing members of the healthcare system, fostering a sense of belonging and support from the system, which resulted in improved job satisfaction, motivation, and retention, and ultimately benefiting both the healthcare system and the communities they serve (Goudge et al., 2023, Malatji et al., 2022). Moreover, being employed by the Ministry of Health led to facility managers taking more responsibility for the CHW team. This allowed them to enhance the existing improvements by the nurse mentor, improving the integration of the CHW program into the facility. Additionally, boosting CHWs' capacity by enhancing clinical knowledge, client engagement skills, and relationships with clinic staff, shifted the care model towards more comprehensive and effective service provision (Goudge et al., 2023, Malatji et al., 2022). A study by Murphy et al., 2021 on the implementation of the CHW policy framework for CHW outreach teams known as Ward-Based Primary Healthcare Outreach Teams (WBPHCOT) in South Africa showed that clarity in the CHW policy framework and understanding of their roles and responsibilities of CHWs provided them with a sense of purpose and clarity (Murphy et al., 2021). Whereas the presence of non-government organisations (NGOs) sometimes created confusion about work roles, lack of communication between CHWs and healthcare facilities was a symptom of transitioning from NGO to Department of Health supervision (Murphy et al., 2021). In addition, clarity on elements of the policy framework, such as required qualifications of CHWs, was raised as an issue at the facility and community level (Murphy et al., 2021). Moreover, in an intervention to enhance maternal and newborn survival in South Africa, CHWs were integrated into the formal health system. They were assigned specific roles and responsibilities, with institutional support provided for their training and performance. As a result, CHWs felt recognized and acknowledged as legitimate healthcare providers, leading to an enhancement in the delivery of maternal and child healthcare services (Ndaba et al., 2019). A study by Enguita-Fernàndez et al., 2021 showed that when CHWs are integrated into the formal health system, this assures the communities that the care they provide meets quality and standard requirements set by the health system, fostering trust, which leads to acceptability of the services they provide (Enguita-Fernàndez et al., 2021). This study further suggested that public display of CHWs’ training credentials to the community increased perceived competence of CHWs, fostering trust from the community, leading to acceptability of the services they offer.

In Cameroon, CHWs engaged families through household visits, providing direct support and guidance to improve the livelihoods of persons with disabilities (Yuh et al., 2023). This approach led to the development of strong community bonds between CHWs, individuals with disabilities, and their families. These relationships were crucial for the success of the intervention, as they facilitated open communication, collaboration, and acceptance of recommendations, fostering a positive environment for change and people-centred care. Similarly, an intervention in South Africa focused on reducing tuberculosis stigma through a community-centred care model, showcasing its benefits. By integrating TB support groups and family counselling to dispel myths and provide essential support, the intervention led to improved overall well-being, stronger community bonds, and fewer missed treatment doses among TB and HIV patients, indicating enhanced treatment adherence (Hayward et al., 2024). Furthermore, a study by Feldhaus et al. (2015) uncovered the importance of addressing cultural norms through gender matching, particularly when dealing with culturally sensitive issues such as sexual and reproductive health. This approach promoted better communication, cultural understanding, and participation, highlighting the need for culturally sensitive strategies in healthcare interventions to enhanced acceptability of CHW services, reduce stigma and access barriers (Feldhaus et al., 2015). These findings highlight the transformative potential of reorienting the model of care towards community-centred care models in addressing diverse health needs and fostering people-centred healthcare systems.

### 3.5 Coordinating services within and across sectors

Coordinating services strategy involves arranging them in accordance with the needs and preferences of individuals. This involves the integration of healthcare providers within and across diverse healthcare settings, the establishment of referral systems and networks across multiple levels of care, and the formation of linkages between the health sector and other sectors. Intersectoral action at the community level is required to address the social determinants of health and make the most efficient use of limited resources. This may involve forming partnerships with the private sector and other government sectors beyond the ministry of health. In addition, coordination of services aims to improve care delivery by synchronizing and integrating processes and information across various services, without merging their structures, services, or workflows (World Health Assembly 69 69, 2016). Fourteen articles elaborated on coordinating services within and across sectors. Fig. 6 illustrates key contextual factors observed in these articles, including positive relationships with healthcare professionals, formal connections to clinics, support from leadership, structured administration, regular engagement with supervisors, and collaboration across sectors (Adam et al., 2014, le Roux et al., 2015, Yuh et al., 2023, Abbey et al., 2014, Busza et al., 2018, Dziva Chikwari et al., 2018, Youngui et al., 2024, Ferrand et al., 2017, Goudge et al., 2020, Ndaba et al., 2019, Viljoen et al., 2021a, Razafinjato et al., 2024, Mulubwa et al., 2020, Tinago et al., 2024). These interact with some of the mechanisms identified in the articles to produce outcomes such as improved retention, performance, service delivery, and holistic care. Three mechanisms were identified in the strategy of coordinating services within and across sectors: (World Health Organization, 2015) motivation and morale, (Azevedo, 2017) competence and confidence, and (Freijser et al., 2023) recognition, support, and rapport.

In Ghana, cordial relationships with professional health staff and the community at large contributed to CHWs' motivation to stay in the program. Furthermore, recognition and appreciation from the community and professional health staff also served as a source of motivation for CHWs to continue their work. In addition, CHWs who received approval from the community and their immediate family were more likely to remain in the program, indicating the importance of social support and rapport (Abbey et al., 2014). In a study conducted in Zimbabwe, CHWs facilitated the seamless transfer of patients between clinics, ensuring continuous access to HIV care and highlighting the significance of establishing formalized links between clinics and CHWs in improving referrals (Dziva Chikwari et al., 2018). Similarly, in Zambia, CHWs were shown to facilitate coordination by providing a range of services beyond HIV testing, care, and referrals, contributing to a more integrated approach to healthcare delivery (Viljoen et al., 2021b). Furthermore, a study by Adam et al. (2014) indicated that CHWs served as a bridge between the community and clinical services, facilitating the referral process and ensuring timely access to healthcare

In South Africa, a study revealed that having a senior supervisor facilitated collaboration between the CHW teams and the local facility (Goudge et al., 2020). This collaboration enhanced the coordination of care and ensured that the CHWs had access to necessary information and support, leading to the provision of quality services. Furthermore, supervisors accompanied CHWs on home visits, providing on-the-spot training and correcting any practice errors, boosting CHWs’ confidence and competence and further enhancing the quality of care provided (Goudge et al., 2020). In a mentor mothers intervention study by le Roux et al. (2015), it was shown that the supportive clinic and hospital leadership, as well as the enthusiastic support from chiefs, headmen, and families in the area, contributed to the recognition and satisfaction of the mentor mothers, making them feel respected and appreciated in their roles. This integration of CHWs into the healthcare system allowed them to identify and refer new TB/HIV cases, assist sick children, and support at-risk pregnant women, ensuring timely and appropriate care. It established a healthcare network involving hospitals, clinics, and CHWs, promoting coordination and collaboration across various levels of care (le Roux et al., 2015).

In a trial in Zambia and South Africa, a collaboration between CHWs from the study and those employed by the government utilized existing CHW structures and services tailored to the community's needs, resulting in the successful implementation of the intervention (Viljoen et al., 2021b). Furthermore, Ferrand et al. (2017) demonstrated that utilizing already existing community health workers in standard healthcare settings is a practical and expandable method for caring for HIV-infected children and adolescents in resource-limited areas (Ferrand et al., 2017). A study in Cameroon aimed at enhancing the livelihood of people with disabilities highlighted the importance of coordinated efforts across sectors like health, social services, and civil society organizations (Yuh et al., 2023). These efforts were essential in establishing a comprehensive support system for individuals with disabilities. Additionally, collaboration and communication among various service providers facilitated the smooth delivery of resources and assistance to families in need. This study showed that including or collaborating with other sectors also helps to address other social determinants of health that might not be covered by the scope or budget of the Ministry of Health (Yuh et al., 2023). Furthermore, a study conducted in Zimbabwe showed that coordinating services within the community and involving key stakeholders ensured a comprehensive approach to supporting adolescent mothers to mitigate social isolation and stigma (Tinago et al., 2024). In addition, Goudge et al. (2020) indicated that CHWs were able to refer within the health sector by collaborating with other clinic staff, ensuring coordination and integration of care within the health system, and across sectors by referring clients to social services, ensuring they received the necessary care and support (Goudge et al., 2020).

### 3.6 Creating an enabling environment

An enabling environment is one that unites all stakeholders to undertake transformational change, making operational strategies possible. This complex task involves a diverse set of processes to bring about necessary changes in leadership and management, information systems, methods to improve quality, workforce reorientation, legislative frameworks, financial arrangements, and incentives. Furthermore, it enables the four previous strategies to be operational. Eight articles discussed creating an enabling environment strategy (Goudge et al., 2023, Malatji et al., 2022, D’Ambruoso et al., 2023, Hayward et al., 2024, Goudge et al., 2020, Razafinjato et al., 2024, Lindsay et al., 2022, Kletter et al., 2024). Fig. 6 illustrates key contextual factors observed in these articles, such as a supportive environment, flexible management [(an adaptive leadership style characterized by fluidity, responsiveness, and the capacity to adjust to evolving situations (Kuluski et al., 2021)], and safe spaces for CHWs to engage with community members. Through their interaction with certain mechanisms identified in the articles, these result in outcomes such as improvements in care practices, enhanced CHW performance, improved disclosure, enhanced participation, individualized care and better alignment of services with community needs. The creation of an enabling environment strategy involved five key mechanisms: (World Health Organization, 2015) feeling heard, valued, supported, (Azevedo, 2017) empowerment, and (Freijser et al., 2023) open communication and trust (within the team and with the community).

Studies by Malatjie et al. (2022) and Goudge et al. (2023) indicated that a nursing mentor helped CHWs feel heard, valued, and supported. For example, the nurse mentor identified the inefficiencies in CHW activities and developed operational systems to address them. The process included negotiating with facility staff and setting up essential systems to enhance CHW performance. Moreover, by working closely with CHWs, the nurse mentor facilitated continuous learning and professional growth, creating a supportive environment for skill development (Goudge et al., 2023, Malatji et al., 2022).

Support from the local facility and collaboration with clinic staff have been shown to create a supportive environment for the program's implementation and delivery of quality care (le Roux et al., 2015, Goudge et al., 2020). A study by le Roux et al. (2015) also demonstrated that the enthusiastic support of chiefs and headmen and the welcoming attitudes of families in the area create a supportive environment for CHWs to carry out their work (le Roux et al., 2015). Hayward et al. (2024) indicated that empowering TB survivors to be peer research associates was a crucial factor in informing stigma interventions and driving policy change, and that incorporating community representatives throughout the research process promoted cultural sensitivity and equalized power dynamics, fostering a supportive and conducive atmosphere (Hayward et al., 2024). Furthermore, a study by Kletter et al. (2024) showed that engaging CHWs in a co-design process ensured that they felt valued and appreciated, resulting in a boost in morale and confidence (Kletter et al., 2024).

In Zambia, a study by Lindsay et al. (2022) created a supportive environment by using peer CHWs to reach key populations (KPs), establish rapport, and build trust within the communities, which contributed to the positive outcomes (Lindsay et al., 2022). Furthermore, offering HIV testing services in safe spaces to KPs was considered to be a crucial factor in the success of the intervention, as this created a supportive environment where they felt safe and comfortable, fostering trust, rapport, open communication, and ultimately improved disclosure and participation (Lindsay et al., 2022). A study by D’ambruoso et al. (2023) showed that having regular spaces for dialogue and mutual learning supported CHWs to gain tools and skills to rework their agency in more empowered ways (D’Ambruoso et al., 2023). Furthermore, the training intervention created a supportive environment for learning through the peer modality of support and exchange, which helped CHWs develop analytical, facilitation, and public speaking skills.

**Fig. 7:**
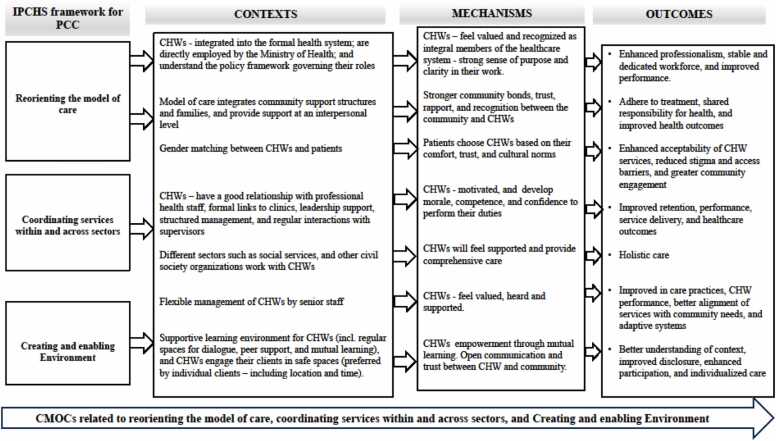
Context, Mechanisms, and Outcome configurations (CMOCs) for the reorientation of the care model, coordination of services within and across sectors, and creating an enabling of an environment.

### 3.7 Synthesized insights, Cross-cutting Mechanisms, and Contributions to the IPCHS framework

Context of successful community engagement included CHWs embedded within local cultures (Malatji et al., 2022, Yuh et al., 2023), participatory selection processes (Adam et al., 2014, Abbey et al., 2014), and co-designed health materials (Abbey et al., 2015). These contexts activated critical mechanisms such as trust and rapport evidenced by informal health-seeking behaviours (Malatji et al., 2022), cultural competence demonstrated by CHWs with shared lived experiences reducing stigma (Ferrand et al., 2017), and community ownership through involvement in care decisions (Abbey et al., 2015). These mechanisms collectively led to outcomes of improved service uptake and health behaviours (Adam et al., 2014, Abbey et al., 2015). Trust emerged as a fundamental mechanism not only for engagement but also for governance and coordination – a cross cutting finding that challenges the categorical boundaries of IPCHS. For example, in Cameroon, trust between CHWs and families of people with disabilities helped in both community participation (IPCHS Strategy 1) and effective service coordination (Strategy 4) (Yuh et al., 2023).

CHW programmes were effective in contexts that activated certain critical governance mechanisms. Government support—such as giving out equipment, transport money and uniforms in Ghana and Nigeria (Abbey et al., 2014, Adesoro et al., 2021) boosted CHW motivation, which in turn enhanced the quality of care given. Supervision and mentorship on regular basis [Zimbabwe (Busza et al., 2018, Dziva Chikwari et al., 2018); Uganda (Wanduru et al., 2016)] enhanced CHWs’ competence and confidence hence enhancing the quality of service delivery. On the other hand, contexts of delayed payments and poor working conditions [South Africa (Klingberg et al., 2021)] lead to demotivation and, therefore, performance. Good national policies on salaries [Kenya, South Africa (Goudge et al., 2023, Rogers et al., 2023)] enhanced the sense of value of the CHWs and, therefore, retain them. Another crucial finding was that governance mechanisms (i.e. motivation) also supported service coordination, which indicates that IPCHS’s clear distinction between governance (Strategy 2) and coordination (Strategy 4) might need to be further unpacked, as there are cross-cutting mechanisms between the two strategies.

To shift care to people-centred models it was necessary to integrate CHWs into formal health systems [South Africa (Goudge et al., 2023, Malatji et al., 2022)] and involve families in care [Cameroon (Yuh et al., 2023)]. These contexts activated mechanisms of legitimacy, where CHWs felt recognized as healthcare providers, improving retention (Malatji et al., 2022). Gender-matching in Tanzania (Feldhaus et al., 2015) increased cultural sensitivity, which helped in the reduction of stigma for sexual and reproductive health services. Community trust was built when CHWs showed credentials [Madagascar (Adesoro et al., 2021)] thus improving service acceptability.

Success in coordination was dependent on intersectoral collaboration [Cameroon (Yuh et al., 2023)] and formal clinic linkages [Zimbabwe (Dziva Chikwari et al., 2018)]. CHWs with positive facility relations [South Africa (Goudge et al., 2020)] had higher levels of morale, which in turn enhanced the referral systems. Intersectoral partnerships for instance, between health and social services in Cameroon (Yuh et al., 2023) provided comprehensive care, and this included addressing issues like poverty. For instance, Zambian CHWs (Viljoen et al., 2021b) worked with government teams to ensure that HIV services were appropriate for the people they served, thus showing that coordination works better when there is governance (for instance, proper management) and community trust. This is counter to IPCHS’s view of coordination as a separate activity, as good coordination needed elements of governance (supervision) and engagement (trust). This suggests the need to incorporate feedback loops between IPCHS strategies to enhance coherence and comprehensiveness to the framework.

Flexible management [South Africa (Goudge et al., 2023)], peer-learning and safe spaces [South Africa (D’Ambruoso et al., 2023)] were critical, yet underemphasized contexts. Nurse mentors who had flexible workflows based on feedback (Goudge et al., 2023) empowered CHWs, thus, enhancing services delivery. Safe spaces for key populations in Zambia (Lindsay et al., 2022) helped establish rapport, which in turn led to higher rates of HIV disclosure. These findings show that there is a gap in IPCHS: ‘Adaptive leadership’ (Kuluski et al., 2021) should be incorporated as a sub-strategy under enabling environments.

Our analysis reveals three fundamental cross-cutting insights that transcend the IPCHS framework's categorical boundaries. First, trust emerges as a foundational meta-mechanism (higher-order or overarching mechanisms that operate across multiple contexts or layers within a system) that operates bidirectionally. Community trust in CHWs [built through cultural embeddedness (Malatji et al., 2022, Yuh et al., 2023) and participatory selection (Adam et al., 2014, Abbey et al., 2014)] enables service uptake, while CHWs' trust in the system through integration (Goudge et al., 2023, Malatji et al., 2022) and fair compensation (Rogers et al., 2023) enhances performance. This challenges IPCHS's siloed structure, as seen in Cameroon where trust simultaneously strengthened community participation (Strategy 1) and service coordination (Strategy 4) (Yuh et al., 2023). Second, motivation functions as an ecosystem where systemic [government support (Abbey et al., 2014; Adesoro et al., 2021)], interpersonal [supervision - as a social interaction between supervisors and CHWs, influencing motivation (Busza et al., 2018; Goudge et al., 2020)], and individual [skill mastery (Adesoro et al., 2021)]) factors interact. For instance, Zimbabwe's mentorship program boosted both CHW competence and community trust (Busza et al., 2018, Dziva Chikwari et al., 2018)], demonstrating the interdependence of CHW-focused and community-focused mechanisms. Third, we identify adaptive implementation as a critical gap in IPCHS, evidenced by flexible management (Goudge et al., 2023) and peer-learning (D’Ambruoso et al., 2023) that enabled CHWs to respond to contextual complexities - a dimension absent in the original framework.

### 3.8 Refinement of the programme theory

Throughout the review, we engaged in an iterative process that involved analysing individual articles, refining the overarching program theory, and, if required, conducted additional iterative searches for data to assess specific theories or components of the theory. If further searches were needed to elaborate on the CMOCs and the higher-order constructs in the program theory, we revisited earlier steps in the process. This iterative approach continued until we constructed a plausible refined program theory (Fig. 4). We met with the study research committee to present and obtain input on findings and revised the program theory, discussed conclusions, and planned for knowledge translation.

The IPT was refined to include CMOCs that were previously not part of it (see Fig. 4). Four contextual factors under the engaging and empowering people and communities’ strategy were added, these interacted with mechanisms to produce outcomes, such as improved community engagement, empowerment, and individualized care. Cultural sensitivity and empathy, initially part of the context in the IPT, were moved to mechanisms in the refined program theory.

Two theories proposed under strengthening governance and accountability were removed due to lack of support in the literature. Additionally, four new theories were proposed under the refined program theory. Furthermore, two contextual factors from the IPT, a clear policy framework governing CHW roles and gender matching, were added to the reorienting the model of care strategy in the refined program theory. These contextual factors interacted with mechanisms to produce outcomes such as improved professionalism, stable and dedicated healthcare workforce, improved CHW performance, and the acceptability of CHW services by the community.

The IPT highlighted that intersectoral collaboration contributes to a holistic care approach. This concept was further emphasized in the refined program theory, which stresses the importance of collaboration within the healthcare sector as a strategy to achieve IPCHS. This includes formal links to clinics, leadership support, structured management, and regular interactions with supervisors. These contextual factors interact with mechanisms like motivation, morale, competence, and confidence, leading to improved retention and improved CHW performance.

In the creating the enabling environment strategy, CMOs in the IPT were shifted to the strengthening governance and accountability strategy. This change was based on studies indicating that factors like supervision, training, and mentorship were more aligned with the governance and accountability strategy than the creating an enabling environment strategy. In the refined program theory, studies highlighted the importance of contextual factors like a supportive environment, flexible management, and safe spaces (preferred by individual clients in terms of location and time) in creating an enabling environment. These factors interact with mechanisms to achieve outcomes such as adaptive health systems, improved care practices, enhanced CHW performance, and better alignment of services with community needs.

## 4 Discussion

The current study explains how contextual factors and mechanisms interact to produce outcomes for optimizing the roles and functions of CHWs in the service of IPCHS in sub-Saharan Africa. The analysis shows how CHWs' roles can be improved using the five key strategies of the IPCHS framework to deliver people-centred care, namely, (1) engaging and empowering people and communities, (2) strengthening governance and accountability, (3) reorienting the model of care, (4) coordinating services within and across sectors, and (5) creating an enabling environment.

In terms of engaging and empowering people and communities, all the CMOCs entail community participation in service delivery and caring activities. This is a central tenet of people-centeredness and has a strong evidence base in strengthening the impact of programmes such as HIV prevention, and maternal and newborn health in sub-Sahara African contexts (Pollard et al., 2022, Adam et al., 2014, Stansert Katzen et al., 2021, Soepnel et al., 2024a, Soepnel et al., 2024b). Furthermore, CHW cultural competence is key in engaging people and communities as it has been shown by several studies that cultural competence fosters acceptance of interventions by the community (Pollard et al., 2022, Olakkengil et al., 2024, Rogers et al., 2023, Hayward et al., 2024, Adesoro et al., 2021, Feldhaus et al., 2015, Lindsay et al., 2022). This means that a key consideration for CHW programmes should be formalized engagement models with communities, across the cascade of community health activities, which further consider the cultural norms of the community.

The existing evidence gathered in this review emphasized effective supervision, mentorship, and training indicating their central role in the domains of governance and accountability. This implies that all activities of CHWs should be supported by effective supervision models, training, and mentorship. This is strongly supported by evidence showing that proper support leads to improved retention, enhanced performance, high-quality care, and effective delivery of services by CHWs to the communities (Tseng et al., 2019, le Roux et al., 2015, Goudge et al., 2023, Malatji et al., 2022, Yuh et al., 2023, Adesoro et al., 2021, Enguita-Fernàndez et al., 2021, Ndaba et al., 2019, Wanduru et al., 2016, Rotheram-Borus et al., 2023). Another important aspect of this strategy is consistency in salaries and renumeration of CHWs (Murphy et al., 2021, Goudge et al., 2023, Malatji et al., 2022). The findings from studies conducted in Kenya, Madagascar, and South Africa indicate that CHWs felt appreciated, valued, and motivated when national policies were clear, ensuring fair salaries and support (Goudge et al., 2023, Malatji et al., 2022, Rogers et al., 2023, Razafinjato et al., 2024). However, the contrary is often true for unregulated health workers. These individuals are usually paid less because there are no formal regulatory frameworks that set the rates of pay or provide support. Unregulated workers, who are not recognized formally or protected by national policies, are at a disadvantage in terms of salary and job security (Ballard et-al.). This difference points to the need for more comprehensive regulations to guarantee fair remuneration and the recognition of their work, as is the case with their regulated counterparts in the healthcare system. In addition, effective supervision, mentorship, and government support are critically important as they increase the likelihood for CHW success, driving improved performance and high-quality community care (Kok et al., 2015a). Furthermore, clear policies and consistent provision of resources by the government further strengthen CHW roles and functions, ensuring sustainable and impactful service delivery to the communities in need (Ballard et al., 2020). CMOCs pertaining to the direct employment and integration of CHWs into the formal health system were prominent in reorienting the model of care strategy. Studies suggested that direct employment of CHWs by the Department of Health boosted motivation and integration into formal primary healthcare and fostered institutional support for CHWs (Goudge et al., 2023, Malatji et al., 2022). Furthermore, integration of CHWs into the formal health system was associated with positive outcomes, such as effective identification and referral of the community to the clinic. This has been shown in TB/HIV studies, in people with disabilities, ill children, and at-risk pregnant women, ensuring timely and appropriate care (le Roux et al., 2015, Yuh et al., 2023, Ndaba et al., 2019). Key to achieving formal integration of CHWs into the formal health system, as highlighted by Goudge et al., (2020) and Murphy et al., (2021) is having an experienced professional nurse (roving nurse) or outreach team leader (OTL), working with CHW teams to provide the link between the community and the clinic, fostering relationships between the CHW team and health facility staff, and encouraging the sharing of resources (Murphy et al., 2021, Goudge et al., 2020). Additionally, being directly employed and integrated into the formal health system is likely to enhance perceptions of legitimacy by the community of CHWs, leading to greater acceptance of the services they provide (Goudge et al., 2023). Therefore, key to reorienting the model of care to be people-centred is the formal employment of CHWs by the ministry of health and integration into the formal health system as doing this may lead to improved health outcomes at a community level.

In terms of coordinating services within and across sectors, all CMOCs that were uncovered focused on two aspects which were intersectoral collaboration and coordination of services within the healthcare system. Intersectoral collaboration is essential to provide comprehensive and holistic care to the community by combining services and resources from various stakeholders and departments to ensure all necessary services are accessible to the people and communities in need. A study enhancing the livelihoods of individuals with disabilities in Cameroon shows that coordinated efforts among various sectors such as health, social services, and civil society organizations were crucial as part of a comprehensive support system for persons with disabilities (Yuh et al., 2023). Furthermore, collaboration and communication between different service providers facilitated the smooth delivery of resources and assistance to families in need (Yuh et al., 2023). This has also been shown in a study on supporting adolescent mothers (Tinago et al., 2024). Establishing formal connections between CHWs and the clinic, receiving leadership support, and implementing structured management are vital components for service coordination within the healthcare system. These factors help CHWs establish a supportive environment, establish clear expectations with consistent backing, establish their credibility, and gain access to necessary resources (Adam et al., 2014, le Roux et al., 2015, Busza et al., 2018, Dziva Chikwari et al., 2018, Youngui et al., 2024, Goudge et al., 2020, Ndaba et al., 2019, Razafinjato et al., 2024, Mulubwa et al., 2020, Viljoen et al., 2021b). Further evidence of support from the local facility together with facilitated collaboration from a senior supervisor improved the quality of care provided by the CHW teams (Goudge et al., 2020). Therefore, collaboration and coordination of services within and across sectors is crucial in providing comprehensive and holistic care which are key to IPCHS.

The evidence in this review suggests that flexible management of CHWs helps to create an enabling environment. With a flexible management, systems, schedules, and strategies can be adjusted based on feedback and changing circumstances within the communities. Flexibility and a leadership open to CHW concerns is likely to ensure better delivery of services aligned with people's needs, central to IPCHS. Flexible management was demonstrated in the study by Goudge et al. (2023), where a dedicated senior person within the CHW teams, the nurse mentor, was able to work out what systems were required, negotiate with facility staff to establish them, and navigate problems when they arose, contributing to the improvement of CHW performance and the delivery of services that are aligned with people and community needs. Additionally, a supportive learning environment is crucial for CHWs. It enables regular spaces for dialogue, peer support, and mutual learning, helping CHWs gain the tools and skills to rework their agency in more empowered ways (D’Ambruoso et al., 2023, Razafinjato et al., 2024, Lindsay et al., 2022, Kletter et al., 2024). This increases the prospects of improved communication and relationships among CHWs and communities they serve and thereby enhancing the tenets of a people-centred care that encourages understanding of contexts and increased community involvement.

The synthesized insights from this study show that trust, motivation, and adaptive implementation are interconnected mechanisms that go beyond the categorical boundaries of the WHO’s IPCHS framework. Trust was identified as a meta-mechanism that underpinned both community engagement and service coordination, a finding that is consistent with previous research that has highlighted trust as a key component of successful CHW programmes (Schaaf et al., 2020). In addition, the study supports previous findings that components of governance such as supervision and fair compensation increase CHW motivation and performance (Kok et al., 2015b), and also shows how these mechanisms have spillover effects into service coordination, which is not fully captured by the IPCHS framework. The finding that adaptive implementation is a critical gap is in line with calls for more flexible and context-responsive health systems (Kuluski et al., 2021), and suggests that adaptive leadership should be integrated as a formal sub-strategy. These cross-cutting insights challenge the siloed structure of the IPCHS framework and highlight the dynamic interplay between its strategies, providing a more holistic understanding of how CHW programmes can achieve people-centred care.

### 4.1 Strengths and limitations

The review focused on intervention studies, with most describing the theories they applied to reach conclusions. Furthermore, some of the studies conducted evaluations which helped to unpack the context, mechanisms, and outcomes of the interventions. Another strength of this review is the diversity of the research team comprising individuals from varying professional backgrounds including spatial epidemiology, public health, nursing, psychology, sociology and political science. The workshop model that was implemented during the data analysis phase facilitated a critical analysis of the data.

Since the review only included English peer-reviewed articles, our findings may not apply to non-English speaking sub-Saharan African countries without translation. Relevant non-English articles may have been excluded from this study since the authors were linguistically limited in reviewing non-English text, leading to a potential loss of important information. Another limitation is the possibility of publication bias, as unpublished or non-indexed studies may have been missed. While we included researchers from diverse disciplinary backgrounds to mitigate interpretation bias, variations in disciplinary perspectives may have influenced how findings were interpreted. However, this provoked valuable debate and discussions that contributed to theory refinement. Furthermore, the review protocol was not published, which may limit the transparency and replicability of the review process.

### 4.2 Practical implications and framework recommendations

The findings from this review article suggest concrete actions for optimizing CHW programmes. Firstly, interventions should combine CHW-focused and community-focused components, such as pairing supervision with trust-building activities. Second, trust should be measured as a core programme metric, recognizing its role across all IPCHS strategies. Third, the framework requires revision to: (World Health Organization, 2015) incorporate feedback loops between strategies; (Azevedo, 2017) add adaptive leadership as a formal sub-strategy; and (Freijser et al., 2023) acknowledge overlapping mechanisms that are simultaneously in play across different IPCHs strategies. Additionally, from a realist perspective, imposing categorical constraints to address the complexities of CHW roles, responsibilities, and the delivery of people-centered care using the IPCHS framework limits the ability to fully understand and unpack the contextual factors and mechanisms at play and how they interact realistically as they do in the real world without restrictions.

## 5 Conclusion

This realist synthesis provides important insights on optimizing the roles of CHWs to promote people-centred care in sub-Saharan Africa. The study demonstrates how context, mechanisms, outcomes, and the five IPCHS framework strategies interact. It explores CHW performance by examining important contextual factors and mechanisms. The study identified trust, motivation, and adaptive leadership as fundamental meta-mechanisms. These mechanisms challenged the isolated structure of the IPCHS framework and highlighted the need for improvements to allow more flexibility to study interactions that occur within different strategies of the IPCHS framework to address complexities that CHWs face when delivering people-centred care. The research shows that service delivery and health outcomes improve through community involvement, formal CHW system integration, intersectoral partnerships, and flexible management systems. The study offers practical recommendations, such as using trust as a key measurement tool, integrating CHW-specific interventions with community-based interventions, and updating the IPCHS framework to incorporate adaptive leadership and feedback mechanisms.

Further empirical research should validate the proposed theories and build on the study's strong foundation for understanding CHW programmes. Additionally, research should examine intersectoral collaboration and its potential to drive change in CHW performance and the delivery of people-centred care, given the limited existing research on this topic. Ultimately, this realist synthesis enriches the discussion on CHWs by providing a detailed realist analysis of effective strategies to enhance their impact in delivering people-centred care in resource-limited settings.

## Ethical declaration

This study did not need ethical approval, as it focused on reviewing published literature and did not involve collecting primary or individual-level data from human subjects.

## Funding

This research has been supported by funding from the UK Foreign, Commonwealth & Development Office (FCDO), Medical Research Council (10.13039/501100000265MRC) and 10.13039/100004440Wellcome Trust (MR/V015044/1) (Duration - Sept 2021 - September 2024). And “The work reported herein was supported by the South African Medical Research Council through its Division of Research Capacity Development under the SAMRC Researcher Development Award with funding received from the South African National Department of Health. The content hereof is the sole responsibility of the authors and do not necessarily represent the official views of the 10.13039/501100001322SAMRC”.

## CRediT authorship contribution statement

**Zamasomi Luvuno:** Writing – review & editing, Supervision. **Arvin Bhana:** Writing – review & editing, Validation, Supervision, Methodology. **Tasneem Kathree:** Writing – review & editing, Supervision, Project administration. **Inge Petersen:** Writing – review & editing, Validation, Supervision, Resources, Funding acquisition, Conceptualization. **Buthelezi Usangiphile Evile:** Writing – review & editing, Writing – original draft, Visualization, Methodology, Investigation, Formal analysis, Data curation, Conceptualization. **Mosa Moshabela:** Writing – review & editing, Supervision, Conceptualization. **André J van Rensburg:** Writing – review & editing, Validation, Supervision, Resources, Methodology, Investigation, Funding acquisition, Formal analysis, Data curation, Conceptualization.

## Declaration of Competing Interest

The authors declare that they have no known competing financial interests or personal relationships that could have appeared to influence the work reported in this paper.

## Data Availability

All papers used are published online. Other information can be obtained from the corresponding author by request.

## References

[bib1] Abbey M., Bartholomew L.K., Nonvignon J., Chinbuah M.A., Pappoe M., Gyapong M. (2014). Factors related to retention of community health workers in a trial on community-based management of fever in children under 5 years in the Dangme West District of Ghana. Int Health.

[bib2] Abbey M., Bartholomew L.K., Pappoe M., van den Borne B. (2015). Treating fever in children under 5 years of age: caregiver perceptions of community health worker services in Dangme West district, Ghana. Int Health.

[bib3] Adam M.B., Dillmann M., Chen M. kuang, Mbugua S., Ndung’u J., Mumbi P. (2014). Improving Maternal and Newborn Health: Effectiveness of a Community Health Worker Program in Rural Kenya. PLoS One.

[bib4] Adesoro O., Oresanya O., Counihan H., Hamade P., Eguavon D., Emebo C. (2021). A feasibility study to assess non-clinical community health workers’ capacity to use simplified protocols and tools to treat severe acute malnutrition in Niger state Nigeria. BMC Health Serv. Res..

[bib5] Arksey H., O’Malley L. (2005). Scoping studies: towards a methodological framework. Int. J. Soc. Res. Methodol..

[bib6] Azevedo M.J. (2017). The State of Health System(s) in Africa: Challenges and Opportunities. Hist. Perspect. State Health Health Syst. Afr..

[bib7] Ballard M., Westgate C., Alban R., Choudhury N., Adamjee R., Schwarz R., et al. Compensation models for community health workers: Comparison of legal frameworks across five countries. J Glob Health. 11:04010. 10.7189/jogh.11.04010 PMC7916445 33692894

[bib8] Ballard M., Bancroft E., Nesbit J., Johnson A., Holeman I., Foth J. (2020). Prioritising the role of community health workers in the COVID-19 response. BMJ Glob. Health.

[bib9] Bourgeault I.L., Myles S., McMurchy D. (2025). https://www.hhr-rhs.ca/images/PDFs/A%20Compendium%20of%20Roles%20in%20Team-Based%20Primary%20Care%20TPC%20April%202025.pdf.

[bib10] Brennan N., Bryce M., Pearson M., Wong G., Cooper C., Archer J. (2017). Towards an understanding of how appraisal of doctors produces its effects: a realist review. Med. Educ..

[bib11] Brownstein J.N., Hirsch G.R., Rosenthal E.L., Rush C.H. (2011). Community health workers “101” for primary care providers and other stakeholders in health care systems. J. Ambul. Care Manag..

[bib12] Bunn F., Goodman C., Manthorpe J., Durand M.A., Hodkinson I., Rait G. (2017). Supporting shared decision-making for older people with multiple health and social care needs: a protocol for a realist synthesis to inform integrated care models. BMJ Open.

[bib13] Busza J., Dauya E., Bandason T., Simms V., Chikwari C.D., Makamba M. (2018). The role of community health workers in improving HIV treatment outcomes in children: lessons learned from the ZENITH trial in Zimbabwe. Health Policy Plan.

[bib14] D’Ambruoso L., Abruquah N.A., Mabetha D., van der Merwe M., Goosen G., Sigudla J. (2023). Expanding Community Health Worker decision space: learning from a Participatory Action Research training intervention in a rural South African district. Hum. Resour. Health.

[bib15] Dalkin S.M., Greenhalgh J., Jones D., Cunningham B., Lhussier M. (2015). What’s in a mechanism? Development of a key concept in realist evaluation. Implement. Sci..

[bib16] Dziva Chikwari C., Simms V., Busza J., Dauya E., Bandason T., Chonzi P. (2018). Community health worker support to improve HIV treatment outcomes for older children and adolescents in Zimbabwe: a process evaluation of the ZENITH trial. Implement Sci..

[bib17] Enguita-Fernàndez C., Alonso Y., Lusengi W., Mayembe A., Manun’Ebo M.F., Ranaivontiavina S. (2021). Trust, community health workers and delivery of intermittent preventive treatment of malaria in pregnancy: a comparative qualitative analysis of four sub-Saharan countries. Glob. Public Health.

[bib18] Farmanova E., Baker G.R., Cohen D. (2019). Combining Integration of Care and a Population Health Approach: A Scoping Review of Redesign Strategies and Interventions, and their Impact. Int J. Integr. Care.

[bib19] Feldhaus I., Silverman M., LeFevre A.E., Mpembeni R., Mosha I., Chitama D. (2015). Equally able, but unequally accepted: Gender differentials and experiences of community health volunteers promoting maternal, newborn, and child health in Morogoro Region, Tanzania. Int. J. Equity Health.

[bib20] Ferrand R.A., Simms V., Dauya E., Bandason T., Mchugh G., Mujuru H. (2017). The effect of community-based support for caregivers on the risk of virological failure in children and adolescents with HIV in Harare, Zimbabwe (ZENITH): an open-label, randomised controlled trial. Lancet Child Adolesc. Health.

[bib21] Fleming P., Caffrey L., Van Belle S., Barry S., Burke S., Conway J. (2023). How International Health System Austerity Responses to the 2008 Financial Crisis Impacted Health System and Workforce Resilience – A Realist Review. Int. J. Health Policy Manag..

[bib22] Flynn R., Schick-Makaroff K., Levay A., Greenhalgh J. (2020). Developing an Initial Program Theory to Explain How Patient-Reported Outcomes Are Used in Health Care Settings: Methodological Process and Lessons Learned. Int. J. Qual. Methods.

[bib23] Freijser L., Annear P., Tenneti N., Gilbert K., Chukwujekwu O., Hazarika I. (2023). The role of hospitals in strengthening primary health care in the Western Pacific. Lancet Reg. Health West Pac..

[bib24] Glenton C., Javadi D., Perry H.B. (2021). Community health workers at the dawn of a new era: 5. Roles and tasks. Health Res Policy Sys.

[bib25] Goicolea I., Hurtig A.K., San Sebastian M., Vives-Cases C., Marchal B. (2015). Developing a programme theory to explain how primary health care teams learn to respond to intimate partner violence: a realist case-study. BMC Health Serv. Res.

[bib26] Goudge J., de Kadt J., Babalola O., Muteba M., Tseng Y.H., Malatji H. (2020). Household coverage, quality and costs of care provided by community health worker teams and the determining factors: findings from a mixed methods study in South Africa. BMJ Open.

[bib27] Goudge J., Babalola O., Malatji H., Levin J., Thorogood M., Griffiths F. (2023). The effect of a roving nurse mentor on household coverage and quality of care provided by community health worker teams in South Africa: a longitudinal study with a before, after and 6 months post design. BMC Health Serv. Res..

[bib28] Greenhalgh J., Manzano A. (2022). Understanding ‘context’ in realist evaluation and synthesis. Int. J. Soc. Res. Methodol..

[bib29] Håkansson Eklund J., Holmström I.K., Kumlin T., Kaminsky E., Skoglund K., Höglander J. (2019). Same same or different?” A review of reviews of person-centered and patient-centered care. Patient Educ. Couns..

[bib30] Hartzler A.L., Tuzzio L., Hsu C., Wagner E.H. (2018). Roles and Functions of Community Health Workers in Primary Care. Ann. Fam. Med.

[bib31] Hayward S.E., Vanqa N., Makanda G., Tisile P., Ngwatyu L., Foster I. (2024). “As a patient I do not belong to the clinic, I belong to the community.” Co-developing a multi-level, person-centred tuberculosis stigma intervention in Cape Town, South Africa. Res Sq..

[bib32] Jagosh J. (2020). Retroductive theorizing in Pawson and Tilley’s applied scientific realism. J. Crit. Realism.

[bib33] Jenson A., Roter D.L., Mkocha H., Munoz B., West S. (2018). Patient-centered communication of community treatment assistants in Tanzania predicts coverage of future mass drug administration for trachoma. Patient Educ. Couns..

[bib34] Kane S., Kok M., Ormel H., Otiso L., Sidat M., Namakhoma I. (2016). Limits and opportunities to community health worker empowerment: A multi-country comparative study. Soc. Sci. Med..

[bib35] Kantilal K., Hardeman W., Whiteside H., Karapanagiotou E., Small M., Bhattacharya D. (2020). Realist review protocol for understanding the real-world barriers and enablers to practitioners implementing self-management support to people living with and beyond cancer. BMJ Open.

[bib36] Khatri R.B., Wolka E., Nigatu F., Zewdie A., Erku D., Endalamaw A. (2023). People-centred primary health care: a scoping review. BMC Prim. Care.

[bib37] Kletter M., Harris B., Connolly E., Namathanga C., Nhlema B., Makungwa H. (2024). Mixed method evaluation of a learning from excellence programme for community health workers in Neno, Malawi. BMC Health Serv. Res.

[bib38] Klingberg S., van Sluijs E.M.F., Jong S.T., Draper C.E. (2021). Can public sector community health workers deliver a nurturing care intervention in South Africa? The Amagugu Asakhula feasibility study. Pilot Feasibility Stud..

[bib39] Kok M.C., Kane S.S., Tulloch O., Ormel H., Theobald S., Dieleman M. (2015). How does context influence performance of community health workers in low- and middle-income countries? Evidence from the literature. Health Res. Policy Syst..

[bib40] Kok M.C., Kane S.S., Tulloch O., Ormel H., Theobald S., Dieleman M. (2015). How does context influence performance of community health workers in low- and middle-income countries? Evidence from the literature. Health Res Policy Syst..

[bib41] Kuluski K., Reid R.J., Baker G.R. (2021). Applying the principles of adaptive leadership to person-centred care for people with complex care needs: Considerations for care providers, patients, caregivers and organizations. Health Expect..

[bib42] le Roux K., le Roux I., Mbewu N., Davis E. (2015). The Role of Community Health Workers in the Re-Engineering of Primary Health Care in Rural Eastern Cape. S Afr. Fam. Pr. (2004).

[bib43] LeBan K., Kok M., Perry H.B. (2021). Community health workers at the dawn of a new era: 9. CHWs’ relationships with the health system and communities. Health Res. Policy Syst..

[bib44] LeBan K., Kok M., Perry H.B. (2021 Oct 12). Community health workers at the dawn of a new era: 9. CHWs’ relationships with the health system and communities. Health Res. Policy Syst..

[bib45] Lindsay B.R., Mwango L., Toeque M.G., Malupande S.L., Nkhuwa E., Moonga C.N. (2022). Peer community health workers improve HIV testing and ART linkage among key populations in Zambia: retrospective observational results from the Z-CHECK project, 2019–2020. J. Int. AIDS Soc..

[bib46] Malatji H., Griffiths F., Goudge J. (2022). Supportive supervision from a roving nurse mentor in a community health worker programme: a process evaluation in South Africa. BMC Health Serv. Res..

[bib47] Malatji H., Griffiths F., Goudge J. (2024). Mobilisation towards formal employment in the healthcare system: A qualitative study of community health workers in South Africa. PLOS Glob. Public Health.

[bib48] McNeil H., Elliott J., Huson K., Ashbourne J., Heckman G., Walker J. (2016). Engaging older adults in healthcare research and planning: a realist synthesis. Res Involv Engag..

[bib49] Mendin S.F., Krause J.A., Gweh A., Baysah M., Nyumah J., Gaye C.J. (2023). Measuring health system responsiveness in a national community health worker primary care programme in rural Liberia. Int J. Qual. Health Care.

[bib50] Mills S.L., Pumarino J., Clark N., Carroll S., Dennis S., Koehn S. (2014). Understanding how self-management interventions work for disadvantaged populations living with chronic conditions: protocol for a realist synthesis. BMJ Open.

[bib51] Mm R. (2020). https://pubmed.ncbi.nlm.nih.gov/31392601/.

[bib52] Mukumbang F.C., Van Belle S., Marchal B., Van Wyk B. (2016). Realist evaluation of the antiretroviral treatment adherence club programme in selected primary healthcare facilities in the metropolitan area of Western Cape Province, South Africa: a study protocol. BMJ Open.

[bib53] Mulubwa C., Hurtig A.K., Zulu J.M., Michelo C., Sandøy I.F., Goicolea I. (2020). Can sexual health interventions make community-based health systems more responsive to adolescents? A realist informed study in rural Zambia. Reprod. Health.

[bib54] Murphy J.P., Moolla A., Kgowedi S., Mongwenyana C., Mngadi S., Ngcobo N. (2021). Community health worker models in South Africa: a qualitative study on policy implementation of the 2018/19 revised framework. Health Policy Plan..

[bib55] Ndaba T., Taylor M., Mabaso M. (2019). Training and Evaluation of Community Health Workers (CHWs): Towards Improving Maternal and Newborn Survival in an Urban Setting in KwaZulu-Natal, South Africa. Open Public Health J. [Internet].

[bib56] Ndumwa H.P., Amani D.E., Ngowi J.E., Njiro B.J., Munishi C., Mboya E.A., et al. Mitigating the Rising Burden of Non-Communicable Diseases through Locally Generated Evidence-Lessons from Tanzania. Ann Glob Health. 89(1):77. 10.5334/aogh.4111 PMC10655751 38025921

[bib57] Ohly H., Crossland N., Dykes F., Lowe N., Hall-Moran V. (2017). A realist review to explore how low-income pregnant women use food vouchers from the UK’s Healthy Start programme. BMJ Open.

[bib58] Olakkengil M., Said S., Abdalla O., Hofmann R., Hedt-Gauthier B., Fulcher I. (2024). Are populations of postpartum women differentially served by community health worker programs: an observational cohort study from Zanzibar, Tanzania. BMC Pregnancy Childbirth.

[bib59] Pawson R., Tilley N. (1997).

[bib60] Pawson R., Greenhalgh T., Harvey G., Walshe K. (2005). Realist review - a new method of systematic review designed for complex policy interventions. J. Health Serv. Res Policy.

[bib61] Pearson M., Chilton R., Woods H.B., Wyatt K., Ford T., Abraham C. (2012). Implementing health promotion in schools: protocol for a realist systematic review of research and experience in the United Kingdom (UK). Syst. Rev..

[bib62] Pearson M., Brand S.L., Quinn C., Shaw J., Maguire M., Michie S. (2015). Using realist review to inform intervention development: methodological illustration and conceptual platform for collaborative care in offender mental health. Implement. Sci..

[bib63] Perry H.B., Chowdhury M., Were M., LeBan K., Crigler L., Lewin S. (2021). Community health workers at the dawn of a new era: 11. CHWs leading the way to “Health for All. Health Res Policy Syst..

[bib64] Pollard R., Kennedy C.E., Hutton H.E., Mulamba J., Mbabali I., Anok A. (2022). HIV Prevention and Treatment Behavior Change and the Situated Information Motivation Behavioral Skills (sIMB) Model: A Qualitative Evaluation of a Community Health Worker Intervention in Rakai, Uganda. AIDS Behav..

[bib65] Power J., Gilmore B., Vallières F., Toomey E., Mannan H., McAuliffe E. (2019). Adapting health interventions for local fit when scaling-up: a realist review protocol. BMJ Open.

[bib66] Rayyan – Intelligent Systematic Review - Rayyan [Internet]. 2021 [cited 2024 Aug 2]. Available from: 〈https://www.rayyan.ai/〉.

[bib67] Razafinjato B., Rakotonirina L., Cordier L.F., Rasoarivao A., Andrianomenjanahary M., Marovavy L. (2024). Evaluation of a novel approach to community health care delivery in Ifanadiana District, Madagascar. PLOS Glob. Public Health.

[bib68] Rogers A., Goore L.L., Wamae J., Starnes J.R., Okong’o S.O., Okoth V. (2023). Training and experience outperform literacy and formal education as predictors of community health worker knowledge and performance, results from Rongo sub-county, Kenya. Front Public Health [Internet].

[bib69] Rotheram-Borus M.J., le Roux K.W., Norwood P., Stansert Katzen L., Snyman A., le Roux I. (2023). The effect of supervision on community health workers’ effectiveness with households in rural South Africa: A cluster randomized controlled trial. PLoS Med.

[bib70] Schaaf M., Warthin C., Freedman L., Topp S.M. (2020). The community health worker as service extender, cultural broker and social change agent: a critical interpretive synthesis of roles, intent and accountability. BMJ Glob. Health.

[bib71] Schneider H. (2019). The Governance of National Community Health Worker Programmes in Low- and Middle-Income Countries: An Empirically Based Framework of Governance Principles, Purposes and Tasks. Int. J. Health Policy Manag..

[bib72] Soepnel L.M., Mabetha K., Norris S.A., Motlhatlhedi M., Nkosi N., Klingberg S. (2024). The role of a community health worker-delivered preconception and pregnancy intervention in achieving a more positive pregnancy experience: the Bukhali trial in Soweto, South Africa. BMC Women’s. Health.

[bib73] Soepnel L.M., Norris S.A., Mabetha K., Motlhatlhedi M., Nkosi N., Lye S. (2024). A qualitative analysis of community health worker perspectives on the implementation of the preconception and pregnancy phases of the Bukhali randomised controlled trial. PLOS Glob. Public Health.

[bib74] Stansert Katzen L., le Roux K.W., Almirol E., Hayati Rezvan P., le Roux I.M., Mbewu N. (2021). Community health worker home visiting in deeply rural South Africa: 12-month outcomes. Glob. Public Health.

[bib75] Steege R., Taegtmeyer M., Ndima S., Give C., Sidat M., Ferrão C. (2020). Redressing the gender imbalance: a qualitative analysis of recruitment and retention in Mozambique’s community health workforce. Hum. Resour. Health.

[bib76] The EndNote Team, 2013. EndNote. Philadelphia, PA. Clarivate. Version: EndNote 20.

[bib77] Tinago C.B., Frongillo E.A., Warren A.M., Chitiyo V., Jackson T.N., Cifarelli A.K. (2024). Testing the Effectiveness of a Community-Based Peer Support Intervention to Mitigate Social Isolation and Stigma of Adolescent Motherhood in Zimbabwe. Matern Child Health J..

[bib78] Tseng Y.H., Griffiths F., de Kadt J., Nxumalo N., Rwafa T., Malatji H. (2019). Integrating community health workers into the formal health system to improve performance: a qualitative study on the role of on-site supervision in the South African programme. BMJ Open.

[bib79] Viljoen L., Mainga T., Casper R., Mubekapi-Musadaidzwa C., Wademan D.T., Bond V.A. (2021). Community-based health workers implementing universal access to HIV testing and treatment: lessons from South Africa and Zambia-HPTN 071 (PopART). Health Policy Plan.

[bib80] Viljoen L., Bond V.A., Reynolds L.J., Mubekapi-Musadaidzwa C., Baloyi D., Ndubani R. (2021). Universal HIV testing and treatment and HIV stigma reduction: a comparative thematic analysis of qualitative data from the HPTN 071 (PopART) trial in South Africa and Zambia. Socio Health Illn..

[bib81] Wanduru P., Tetui M., Tuhebwe D., Ediau M., Okuga M., Nalwadda C. (2016). The performance of community health workers in the management of multiple childhood infectious diseases in Lira, northern Uganda – a mixed methods cross-sectional study. Glob. Health Action.

[bib82] Wong G., Greenhalgh T., Westhorp G., Buckingham J., Pawson R. RAMESES publication standards: realist syntheses. 2013;14. 10.1186/1741-7015-11-21 PMC3558331 23360677

[bib83] Wong G., Brennan N., Mattick K., Pearson M., Briscoe S., Papoutsi C. (2015). Interventions to improve antimicrobial prescribing of doctors in training: the IMPACT (IMProving Antimicrobial presCribing of doctors in Training) realist review. BMJ Open.

[bib84] World Health Assembly 69 (2016). https://apps.who.int/iris/handle/10665/252698.

[bib85] World Health Organization. WHO global strategy on people-centred and integrated health services. 2015. (Interim Report).

[bib86] World Health Organization. People-centred health care: a policy framework [Internet]. [cited 2024 May 21]. Available from: 〈https://www.who.int/publications-detail-redirect/9789290613176〉.

[bib87] Youngui B.T., Atwine D., Otai D., Vasiliu A., Ssekyanzi B., Sih C. (2024). Integration of HIV Testing in a Community Intervention for Tuberculosis Screening Among Household Contacts of Patients with Tuberculosis in Cameroon and Uganda. J. Acquir Immune Defic. Syndr..

[bib88] Yuh M.N., Ndum Okwen G.A., Miong R.H.P., Bragazzi N.L., Kong J.D., Movahedi Nia Z. (2023). Using an innovative family-centered evidence toolkit to improve the livelihood of people with disabilities in Bamenda (Cameroon): a mixed-method study. Front Public Health.

