# Supplementary material for: Optimizing the role and functions of CHWs in service of a people-centred community health system in sub-Saharan Africa. A realist synthesis
Source: SSM Health Syst. 2025 Dec;5:100089. doi: 10.1016/j.ssmhs.2025.100089 (PMC12678229; doi:10.1016/j.ssmhs.2025.100089)
Supplement: Supplementary file 1 — Supplementary material [file mmc1.docx]

**Initial ‘Context-mechanism-outcome’ hypotheses using the If, Then propositions.**

| **IPCHS framework strategies** | **Context *("If")*** | **Mechanism *("Then")*** | **Outcome** |
| --- | --- | --- | --- |
| Engaging and empowering people and communities | If there is an ongoing positive relationship between CHWs and individuals and their families | then Individuals and their families will develop trust and actively participate in decision-making and care planning with CHWs (Co-creation process and ownership of one’s health) | And this may lead to Individualized care and improved community engagement |
|  | If CHWs demonstrate cultural sensitivity and empathy when providing services. | then there will be cultural congruence, rapport, and communities will become more accepting of the CHW presence. |  |
| Strengthening governance and accountability | If CHWs, service users and communities are part of the policymaking and implementation planning processes. | then, this will foster ownership of the policy directives (people-centred care) | And this will result in better implementation of people-centredness as indicated in policy in the real world |
|  | If CHW activities are routinely captured and monitored | This will increase accountability and provide space for strategic interventions where needed. | And this will result in improved program management, enhanced service delivery, and higher quality care. |
| Reorienting the model of care | If the CHWs are directly employed by the government’s health ministry or department | then CHW roles and responsibilities will be congruent with national directives and with the continuum of practice of the healthcare system | leading to stronger integration of the CHWs into the health system, performance. and higher quality care. |
|  | If the roles and responsibilities of CHWs are formalised and integrated into the healthcare system. | Then there will be a perceived sense of recognition and visibility of CHWs by the community and other healthcare professionals. |  |
| Coordinating services within and across sectors | If there are platforms or structures for CHWs to engage and partner with other sectors and stakeholders | then there will be collaboration and shared accountability between CHWs and other stakeholders | and this may result in a holistic approach to care |
| Enabling Environment | If there is supportive supervision of CHWs | Then CHWs will have self-efficacy, feel empowered to perform their duties and to discuss challenges | And this may result in higher quality of care |
|  | If there is an ongoing training and mentorship of CHWs | Then CHWs will develop confidence and competence in engaging with their clients |  |
