# Supplementary material for: Optimizing the role and functions of CHWs in service of a people-centred community health system in sub-Saharan Africa. A realist synthesis
Source: SSM Health Syst. 2025 Dec;5:100089. doi: 10.1016/j.ssmhs.2025.100089 (PMC12678229; doi:10.1016/j.ssmhs.2025.100089)
Supplement: Supplementary file 2 — Supplementary material [file mmc2.docx]

**Search phrase**

### ("community healthcare worker*" OR "community health worker*" OR "CHW*") AND "Intervention" AND ("Angola" OR "Benin" OR "Botswana" OR "Burkina Faso" OR "Burundi" OR "Cabo Verde" OR "Cameroon" Or "Central African Republic" OR "Chad" OR "Comoros" OR "Congo" OR "Code D’Ivoire" OR "Equatorial Guinea" OR "Eritrea" OR "Eswatini" OR "Ethiopia" OR "Gabon" OR "Gambia" OR "Ghana" OR "GUINEA" OR "Guinea-Bissau" OR "Kenya" OR "Malawi" OR "Lesotho" OR "Liberia" OR "Madagascar" OR "Mali" OR "Mauritania" OR "Mauritius" OR "Mozambique" OR "Namibia" OR "Niger" OR "Nigeria" OR "Rwanda" OR "Senegal" OR "Seychelles" OR "Sierra Leone" OR "Somalia" OR "South Africa" OR "South Sudan" OR "Sudan" OR "Tanzania" OR "Togo" OR "Uganda" OR "Zambia" OR "Zimbabwe")
