# Supplementary material for: Optimizing the role and functions of CHWs in service of a people-centred community health system in sub-Saharan Africa. A realist synthesis
Source: SSM Health Syst. 2025 Dec;5:100089. doi: 10.1016/j.ssmhs.2025.100089 (PMC12678229; doi:10.1016/j.ssmhs.2025.100089)
Supplement: Supplementary file 3 — Supplementary material [file mmc3.docx]

**A realist evaluation of the role and functions of community health workers in service of a people-centred community health system.**

**Investigator CMO Data Extraction Sheet Data Extraction**

The purpose of this document is to extract data from the identified studies in order to formulate one or more explanatory theories that explain Context-Mechanism-Outcome Configurations (CMOC) and to gain insight into how these CMOCs contribute to optimizing the role and functions of community health workers within a people-centred community health system.

In realist methodologies, knowledge accumulation centres on our comprehension of how mechanisms behave in various contexts and the reasons behind how and why this interplay of context and mechanism leads to different outcomes (1). Please fill in the details below for the study you will be assessing. In the subsequent three tables, locate and specify the Context, Mechanism, and Outcomes (CMO) of the intervention discussed in the study you've examined. Definitions and illustrations of ICMO are provided within the tables.

**Kindly fill out the information below for the study you will be reviewing.**

| **Reviewer Name** |  |
| --- | --- |
| **Reference** |  |
| **Country** |  |
| **Setting and population** |  |
| **Aim** |  |
| **Objectives** |  |
| **Methodology** |  |
| **Intervention description** |  |
| **Intervention outcomes** |  |
| **Challenges and limitations** |  |

**Table 1 – Context**

| **Including (but not limited to) beliefs, social and cultural norms, regulations and economic factors (2,3). For example:**   - Individual capabilities (i.e. values, roles, knowledge, purpose). - Interpersonal relationships supporting the interventions (i.e. communication, collaboration, network, influences). - Institutional settings (i.e. informal rules, organisational culture, leadership, policies, resource allocation, local priorities). - Infra-structural system (i.e. political support). - Country and rurality (i.e. small or large/rural vs regional vs remote). - Socio-demographic characteristics: - Funding context or source (i.e. free, personalised, group vs. individual, government funded). |
| --- |
|  |

**Table 2 – Mechanisms**

| - **“An element of reasoning and/or reactions of an individual or collective agents(s) in regard of resources available in a given context to bring about changes through the implementation of an intervention” (4). Check what are the mediators to produce outcomes (not only primary, but also secondary or unintended outcomes).** |
| --- |
|  |

**Table 3 – Outcomes**

| - **Please make note of all (intended or not) outcomes. Please also classify the role and functions outcomes of CHWs using the following Pillars for people-centred care:**   1. Engaging and empowering people and communities  2. Strengthening governance and accountability (Incl. Supervision, training, and support)  3. Reorienting the model of care  4. Coordinating services within and across sectors  5. Enabling environment |
| --- |
|  |

**Table 4 – If, then statements depicting the CMOs.**

| **Please detail the CMOs for the study you reviewed using the If (Context), Then (Mechanism) statements.** |
| --- |
|  |

**Test for relevance (Pearson et al. 2012; 2015; Brennan et al. 2017)**

| **Conceptually Rich** | **Thicker description’ but not ‘conceptually rich’** | **Conceptually Thin** |
| --- | --- | --- |
| Unambiguous theoretical concepts are described in sufficient depth. | Description of programme theory or sufficient information to enable it to ‘surface’. | Insufficient information to enable the programme theory to surface. |
| Relationships between, amongst concepts are clearly articulated. | Consideration of the context in which the programme takes place. | Limited or no consideration of the context in which the programme took place. |
| Concepts are sufficiently developed, defined to enable understanding without the reader needing to have first-hand experience of an area of practice. | Discussion of the differences between the design and orientation of programme theory (what was intended) and implementation (what really happened). | Limited or no discussion of the differences between the design and orientation of programme theory (what was intended) and implementation (what really happened). |
| Concepts are grounded strongly in a cited body of literature. | Recognition and discussion of the strengths/weaknesses of the implemented programme. | Limited or no discussion of the strengths/ weaknesses of the implemented programme. |
| Concepts are parsimonious (i.e., provide the simplest, but not over-simplified, explanation) | Some attempt to explain anomalous results and findings with reference to context and data. | No attempts to explain anomalous results and findings with reference to context and data. |
|  | Description of the factor affecting implementation. | Limited or no description of the factors affecting implementation. |

**Test for relevance (Pearson et al. 2012; 2015; Brennan et al. 2017)**

| **Conceptually Rich** | **Thicker description’ but not ‘conceptually rich’** | **Conceptually Thin** |
| --- | --- | --- |
|  |  |  |

**Test for rigour (Ohly et al. 2017)**

|  | **Yes** | **Fairly** | **No** |
| --- | --- | --- | --- |
| The study methods are clearly reported. |  |  |  |
| The study methods are appropriate to answer RQ. |  |  |  |
| The sample characteristics enable generalizability. |  |  |  |
| Raw data supports the study findings (conclusions). |  |  |  |
| Limitations of the study are acknowledged and clearly reported. |  |  |  |

**References**

1. Wong G, Westhorp G, Manzano A, Greenhalgh J, Jagosh J, Greenhalgh T. (2016). RAMESES II

reporting standards for realist evaluations. *BMC Medicine*, 14(1), 1-18.

2. Wong G, Greenhalgh T, Westhorp G, Pawson R. (2012). Realist methods in medical education

research: what are they and what can they contribute? *Medical Education*, 46(1), 89-96.

3. Macfarlane F, Greenhalgh T, Humphrey C, Hughes J, Butler C, Pawson R. (2011). A new workforce

in the making?: A case study of strategic human resource management in a whole-system change

effort in healthcare. *Journal of Health Organization and Management*, 25(1), 55-72.

4. Lacouture A, Breton E, Guichard A, Ridde V. (2015). The concept of mechanism from a realist

approach: a scoping review to facilitate its operationalization in public health program evaluation.

*Implementation Science*, 10(1), 1-10.

5. Pearson M, Chilton R, Woods HB, Wyatt K, Ford T, Abraham C, et al. Implementing health promotion in schools: protocol for a realist systematic review of research and experience in the United Kingdom (UK). Syst Rev. 2012 Oct 20;1(1):48.

6. Pearson M, Brand SL, Quinn C, Shaw J, Maguire M, Michie S, et al. Using realist review to inform intervention development: methodological illustration and conceptual platform for collaborative care in offender mental health. Implementation Sci. 2015 Sep 28;10(1):134.

7. Brennan N, Bryce M, Pearson M, Wong G, Cooper C, Archer J. Towards an understanding of how appraisal of doctors produces its effects: a realist review. Medical Education. 2017;51(10):1002–13.

8. Ohly H, Crossland N, Dykes F, Lowe N, Hall-Moran V. A realist review to explore how low-income pregnant women use food vouchers from the UK’s Healthy Start programme. BMJ Open. 2017 Apr 21;7(4):e013731.
