# Supplementary material for: Optimizing the role and functions of CHWs in service of a people-centred community health system in sub-Saharan Africa. A realist synthesis
Source: SSM Health Syst. 2025 Dec;5:100089. doi: 10.1016/j.ssmhs.2025.100089 (PMC12678229; doi:10.1016/j.ssmhs.2025.100089)
Supplement: Supplementary file 4 — Supplementary material [file mmc4.docx]

**A realist evaluation of the role and functions of community health workers in service of a people-centred community health system.**

**Investigator CMOc Data Extraction Sheet Data Extraction**

The purpose of this document is to extract data from the identified studies in order to formulate one or more explanatory theories that explain Context-Mechanism-Outcome Configurations (CMOC) and to gain insight into how these CMOCs contribute to optimizing the role and functions of community health workers within a people-centred community health system.

In realist methodologies, knowledge accumulation centres on our comprehension of how mechanisms behave in various contexts and the reasons behind how and why this interplay of context and mechanism leads to different outcomes (1). Please fill in the details below for the study you will be assessing. In the subsequent three tables, locate and specify the Context, Mechanism, and Outcomes (CMO) of the intervention discussed in the study you've examined. Definitions and illustrations of CMO are provided within the tables.

**Kindly fill out the information below for the study you will be reviewing.**

| **Reviewer Name** | Usangiphile Buthelezi |
| --- | --- |
| **Reference** | Tseng YH, Griffiths F, de Kadt J, Nxumalo N, Rwafa T, Malatji H, et al. Integrating community health workers into the formal health system to improve performance: a qualitative study on the role of on-site supervision in the South African programme. BMJ Open. 2019 Feb 27;9(2):e022186. |
| **Country** | South Africa |
| **Setting and population** | - The study was conducted in the Sedibeng Health District, Gauteng Province, South Africa. - The population consisted of community health workers (CHWs) and their supervisors, clinic managers and staff, district managers, key informants from the community, and CHW clients. - The CHWs in the study were part of a national CHW program being implemented in South Africa, which aimed to integrate CHWs into the formal health system. - The CHWs worked in underserved communities where healthcare needs were immense and multifaceted. - The study focused on CHW teams with different configurations of supervision and location, including clinic-based teams and community-based teams. - The supervisors of the CHW teams included senior nurses and junior nurses, who played a crucial role in supporting and guiding the CHWs |
| **Aim** | - The aim of the study was to explore the role of on-site supervision in community health worker (CHW) programs and the integration of CHWs into the formal health system in South Africa. |
| **Objectives** | - The study aimed to compare the functioning of CHW teams reporting to a clinic-based nurse with teams supervised by a community-based nurse. - It also aimed to determine whether a junior nurse could provide adequate supervision given the shortage of senior nurses. - The study aimed to assess the impact of different levels of supervision and the location of CHW teams on their motivation, performance, and integration into the health system. |
| **Methodology** | - The study utilized a case study approach to examine six CHW teams with different configurations of supervision and location in the Sedibeng Health District, Gauteng Province, South Africa. - Qualitative methods, including participant observation, focus group discussions, and interviews, were employed to collect data. - The researchers observed CHWs and their supervisors, conducted focus group discussions, and interviewed various stakeholders such as CHWs, supervisors, clinic managers, district managers, key informants from the community, and CHW clients. - The study employed triangulation of data sources and sites to enhance the validity of the findings. - The researchers collected a substantial amount of data, allowing for comparison and contrast of the functioning of different CHW teams. - The research team selected CHW teams based on their supervision and location configurations, ensuring similarity between pairs of teams. The teams were categorized into three types, and two teams of each type were selected for the study. - The study also involved reviewing raw and summarized data, obtaining informed consent from participants, and providing feedback to relevant stakeholders. |
| **Intervention description** | - The study focused on the integration of community health workers (CHWs) into the formal health system in South Africa. - The intervention involved on-site supervision of CHW teams by senior nurses, which included household visits, on-the-job training, debriefing, reviewing daily logs, and assistance with compiling reports. - CHW teams led by senior nurses were found to be more motivated and performed a greater range of tasks, while junior nurses in these teams were able to fulfill their role effectively. - Clinic-based teams with senior supervisors were better integrated into the health system and ensured continuity of care. - In contrast, teams with only junior supervisors or based in the community had less engagement with clinic staff and were less able to ensure necessary care for patients, resulting in lower levels of trust from clients. - The study recommended strengthening HR management practices, setting guidelines for supervisor engagement in clinics, and supervisory strategies such as home visits to support CHWs' knowledge and skills. |
| **Intervention outcomes** | - CHW teams with senior supervisors showed higher motivation, job satisfaction, and engagement, leading to improved performance and increased trust from clients. - Senior supervisors successfully negotiated a place for CHWs in the health system, resulting in better integration of clinic-based teams and continuity of care. - Effective supervision supported CHWs through household visits, on-the-job training, debriefing, and assistance with compiling reports, leading to improved skills and performance. - CHWs in teams with effective supervision felt valued and part of the team, leading to increased job satisfaction and professional confidence. - In the absence of supportive supervision, CHWs faced challenges in assisting patients, leading to demotivation and frustrations over working conditions. - The study highlighted the importance of supervision in enabling CHWs to provide comprehensive, promotive, and preventative care, and emphasized the need for integration into the health system. |
| **Challenges and limitations** | - The study acknowledges the observer effect (Hawthorne effect) during data collection, which may have influenced the behavior and performance of CHWs. - The study relied on subjective evaluations of CHW performance rather than objective indicators such as coverage and quality of care. - The data collection was limited to a single health district, which may limit the generalizability of the findings to other contexts. - The study focused on the role of different levels of supervision and location of CHW teams, but there may be other factors influencing CHW motivation, performance, and integration into the health system that were not explored. - The study did not provide information on the specific challenges faced by CHWs or the barriers to their integration - into the health system. |

**Table 1 – Context**

| **Including (but not limited to) beliefs, social and cultural norms, regulations and economic factors (2,3). For example:**   - Individual capabilities (i.e. values, roles, knowledge, purpose). - Interpersonal relationships supporting the interventions (i.e. communication, collaboration, network, influences). - Institutional settings (i.e. informal rules, organisational culture, leadership, policies, resource allocation, local priorities). - Infra-structural system (i.e. political support). - Country and rurality (i.e. small or large/rural vs regional vs remote). - Socio-demographic characteristics: - Funding context or source (i.e. free, personalised, group vs. individual, government funded). |
| --- |
| - The study was conducted in South Africa, specifically in the Sedibeng Health District. Sedibeng is a relatively affluent district, but over 20% of its residents fall below the food poverty line. The district has disadvantaged communities with limited access to services such as clinics, transport, water, and electricity. - The study focused on Community Health Worker (CHW) teams in the Sedibeng Health District. There were 39 CHW teams in the district, with each team consisting of 6 to 20 CHWs. Sixteen teams were based at health posts, while the remaining 23 were clinic-based. The CHWs performed primary healthcare-related functions in the community, including health promotion, prevention, and screening. - The CHW teams were part of the government's Ward-Based Outreach Team (WBOT) program, which aimed to provide comprehensive healthcare services. The teams were meant to comprise a professional nurse, CHWs, a health promoter, and an environmental officer, although this was not always the case. The CHWs received standardized training covering various healthcare tasks. |

**Table 2 – Mechanisms**

| - **“An element of reasoning and/or reactions of an individual or collective agents(s) in regard of resources available in a given context to bring about changes through the implementation of an intervention” (4). Check what are the mediators to produce outcomes (not only primary, but also secondary or unintended outcomes).** |
| --- |
| - Effective supervision led to increased motivation, job satisfaction, and engagement among CHWs, resulting in improved performance and integration into the health system. - Senior supervisors played a crucial role in guiding and mentoring CHWs, building relationships, and passing down knowledge to junior supervisors, which enhanced their skills and confidence. - Supportive supervision and collaboration with clinic staff improved communication, coordination, and trust between CHWs and clinic staff. This resulted in better integration, acceptance of referrals, and inclusion of CHWs in training sessions. - Adequate supervision provided opportunities for CHWs to strengthen their knowledge and skills, demonstrate their value in the community, and stay updated on the community's status. - Supervisors addressing deficiencies in resources and facilities, such as negotiating for meeting rooms, improved CHWs' job satisfaction and morale. - The presence of supervisors, particularly professional nurses, made CHWs feel valued and recognized, leading to increased motivation and a sense of being part of the healthcare team. - Effective supervision helped resolve frustrations and working condition issues, preventing demotivation and passive protests among CHWs. - Supervision facilitated the establishment of better relationships, rapport, and trust between CHWs and their supervisors, resulting in improved performance and outcomes. |

**Table 3 – Outcomes**

| - **Please make note of all (intended or not) outcomes. Please also classify the role and functions outcomes of CHWs using the following Pillars for people-centred care:**   1. Engaging and empowering people and communities  2. Strengthening governance and accountability (Incl. Supervision, training, and support)  3. Reorienting the model of care  4. Coordinating services within and across sectors  5. Enabling environment |
| --- |
| - CHW teams with senior supervisors showed higher motivation, job satisfaction, and engagement, leading to improved performance and increased trust from clients. - Senior supervisors successfully negotiated a place for CHWs in the health system, resulting in better integration of clinic-based teams and continuity of care. - Effective supervision supported CHWs through household visits, on-the-job training, debriefing, and assistance with compiling reports, leading to improved skills and performance. - CHWs in teams with effective supervision felt valued and part of the team, leading to increased job satisfaction and professional confidence. - In the absence of supportive supervision, CHWs faced challenges in assisting patients, leading to demotivation and frustrations over working conditions. - The study highlighted the importance of supervision in enabling CHWs to provide comprehensive, promotive, and preventative care, and emphasized the need for integration into the health system.   **Outcomes by PCC Pillars:**  **Engaging and empowering people and communities:**   - Effective supervision and support from senior nurses empowered CHWs to provide comprehensive, promotive, and preventative care, improving access to care in underserved communities. - CHWs led by senior nurses felt more valued and recognized, leading to increased motivation and job satisfaction.   **Strengthening governance and accountability (Incl. Supervision, training, and support):**   - Adequate supervision and integration into the health system were essential for the effectiveness of CHW programs. - Effective supervision by senior nurses supported CHWs through on-the-job training, debriefing, and assistance with compiling reports, improving CHW performance. - Supervision facilitated better communication, coordination, and trust between CHWs and clinic staff, ensuring accountability and quality of care.   **Reorienting the model of care:**   - CHWs, with no professional qualification, performed primary healthcare-related functions in the community, addressing the multifaceted needs of underserved communities. - Effective supervision and integration of CHW programs into the health system required government financing, national-level planning, and training.   **Coordinating services within and across sectors:**   - Clinic-based teams with senior supervisors were better integrated into the health system, ensuring continuity of care and coordination with clinic staff. - Support from the clinic facilitated CHW performance and client trust, enabling coordination and referrals.   **Enabling environment:**   - Effective supervision addressed deficiencies in resources and facilities, improving CHWs' job satisfaction and morale. - Supervision facilitated the establishment of better relationships, rapport, and trust between CHWs and their supervisors, creating an enabling environment for CHW performance. |

**Table 4 – If, then statements depicting the CMOs.**

| **Please detail the CMOs for the study you reviewed using the If (Context), Then (Mechanism) statements.** |
| --- |
| **If, Then statements**   - If CHWs receive supervision under an experienced supervisor, then they feel empowered, valued, recognized and competent, leading to improved performance and effective delivery of care. - If CHWs receive supervision from senior staff, then CHWs will feel valued and recognized by the clinic staff, leading to increased motivation and a sense of being part of the healthcare team, fostering collaboration and trust, leading to the overall integration into the healthcare system. - If supervisors address deficiencies in resources within the facilities on behalf of CHWs, then CHWs will have higher morale, feel supported and empowered to conduct their tasks, leading to improved performance, productivity, and healthcare delivery. |

**Test for relevance (Pearson et al. 2012; 2015; Brennan et al. 2017)**

| **Conceptually Rich** | **Thicker description’ but not ‘conceptually rich’** | **Conceptually Thin** |
| --- | --- | --- |
| Unambiguous theoretical concepts are described in sufficient depth. | Description of programme theory or sufficient information to enable it to ‘surface’. | Insufficient information to enable the programme theory to surface. |
| Relationships between, amongst concepts are clearly articulated. | Consideration of the context in which the programme takes place. | Limited or no consideration of the context in which the programme took place. |
| Concepts are sufficiently developed, defined to enable understanding without the reader needing to have first-hand experience of an area of practice. | Discussion of the differences between the design and orientation of programme theory (what was intended) and implementation (what really happened). | Limited or no discussion of the differences between the design and orientation of programme theory (what was intended) and implementation (what really happened). |
| Concepts are grounded strongly in a cited body of literature. | Recognition and discussion of the strengths/weaknesses of the implemented programme. | Limited or no discussion of the strengths/ weaknesses of the implemented programme. |
| Concepts are parsimonious (i.e., provide the simplest, but not over-simplified, explanation) | Some attempt to explain anomalous results and findings with reference to context and data. | No attempts to explain anomalous results and findings with reference to context and data. |
|  | Description of the factor affecting implementation. | Limited or no description of the factors affecting implementation. |

**Test for relevance (Pearson et al. 2012; 2015; Brennan et al. 2017)**

| **Conceptually Rich** | **Thicker description’ but not ‘conceptually rich’** | **Conceptually Thin** |
| --- | --- | --- |
|  | X |  |

**Test for rigour (Ohly et al. 2017)**

|  | **Yes** | **Fairly** | **No** |
| --- | --- | --- | --- |
| The study methods are clearly reported. | X |  |  |
| The study methods are appropriate to answer RQ. | X |  |  |
| The sample characteristics enable generalizability. |  |  | X |
| Raw data supports the study findings (conclusions). | X |  |  |
| Limitations of the study are acknowledged and clearly reported. | X |  |  |

**References**

1. Wong G, Westhorp G, Manzano A, Greenhalgh J, Jagosh J, Greenhalgh T. (2016). RAMESES II

reporting standards for realist evaluations. *BMC Medicine*, 14(1), 1-18.

2. Wong G, Greenhalgh T, Westhorp G, Pawson R. (2012). Realist methods in medical education

research: what are they and what can they contribute? *Medical Education*, 46(1), 89-96.

3. Macfarlane F, Greenhalgh T, Humphrey C, Hughes J, Butler C, Pawson R. (2011). A new workforce

in the making?: A case study of strategic human resource management in a whole-system change

effort in healthcare. *Journal of Health Organization and Management*, 25(1), 55-72.

4. Lacouture A, Breton E, Guichard A, Ridde V. (2015). The concept of mechanism from a realist

approach: a scoping review to facilitate its operationalization in public health program evaluation.

*Implementation Science*, 10(1), 1-10.

5. Pearson M, Chilton R, Woods HB, Wyatt K, Ford T, Abraham C, et al. Implementing health promotion in schools: protocol for a realist systematic review of research and experience in the United Kingdom (UK). Syst Rev. 2012 Oct 20;1(1):48.

6. Pearson M, Brand SL, Quinn C, Shaw J, Maguire M, Michie S, et al. Using realist review to inform intervention development: methodological illustration and conceptual platform for collaborative care in offender mental health. Implementation Sci. 2015 Sep 28;10(1):134.

7. Brennan N, Bryce M, Pearson M, Wong G, Cooper C, Archer J. Towards an understanding of how appraisal of doctors produces its effects: a realist review. Medical Education. 2017;51(10):1002–13.

8. Ohly H, Crossland N, Dykes F, Lowe N, Hall-Moran V. A realist review to explore how low-income pregnant women use food vouchers from the UK’s Healthy Start programme. BMJ Open. 2017 Apr 21;7(4):e013731.

**A realist evaluation of the role and functions of community health workers in service of a people-centred community health system.**

**Investigator CMOc Data Extraction Sheet Data Extraction**

The purpose of this document is to extract data from the identified studies in order to formulate one or more explanatory theories that explain Context-Mechanism-Outcome Configurations (CMOC) and to gain insight into how these CMOCs contribute to optimizing the role and functions of community health workers within a people-centred community health system.

In realist methodologies, knowledge accumulation centres on our comprehension of how mechanisms behave in various contexts and the reasons behind how and why this interplay of context and mechanism leads to different outcomes (1). Please fill in the details below for the study you will be assessing. In the subsequent three tables, locate and specify the Context, Mechanism, and Outcomes (CMO) of the intervention discussed in the study you've examined. Definitions and illustrations of CMO are provided within the tables.

**Kindly fill out the information below for the study you will be reviewing.**

| **Reviewer Name** | Usangiphile Buthelezi |
| --- | --- |
| **Reference** | Viljoen L, Mainga T, Casper R, Mubekapi-Musadaidzwa C, Wademan DT, Bond VA, et al. Community-based health workers implementing universal access to HIV testing and treatment: lessons from South Africa and Zambia-HPTN 071 (PopART). Health Policy Plan. 2021 Jun 25;36(6):881–90. |
| **Country** | South Africa and Zambia |
| **Setting and population** | - The research was conducted in Zambia and South Africa as part of the HPTN 071 (PopART) trial, focusing on HIV prevention and treatment. - The study involved a cadre of 737 study-specific Community Health Workers (CHWs) working alongside government-employed CHWs to deliver a 'universal' door-to-door HIV prevention package, including HIV testing, in these two countries. - CHWs in both countries provided services beyond HIV testing, care, and referrals, integrating HIV services into wider health screenings and services to enhance the rollout of Universal Test and Treat (UTT) programs. - Notable differences existed in the structure and management of pre-existing CHWs in Zambia and South Africa, leading to variability in recruitment, management, and retention strategies tailored to local institutional requirements and community-specific needs. |
| **Aim** | - The aim of the research was to investigate the role of Community Health Workers (CHWs) in supporting the implementation of a 'universal' door-to-door HIV prevention package, including HIV testing, as part of the HPTN 071 (PopART) trial in Zambia and South Africa |
| **Objectives** | - The study aimed to assess the sociodemographic characteristics of CHWs in both countries, focusing on their recruitment, training, retention, and the challenges they faced during the implementation of the intervention. - Additionally, the research aimed to evaluate the effectiveness of CHWs in delivering consistent HIV services, building trusting relationships with community members, and ensuring the success of the intervention over time |
| **Methodology** | - The study employed a mixed-methods approach, combining qualitative and quantitative data collection methods to evaluate the implementation of the Community Health Worker (CHW) intervention in the HPTN 071 (PopART) trial in Zambia and South Africa. - Quantitative data was collected through baseline surveys with study-specific CHWs from 2014 to 2018, while qualitative data included key informant interviews with study management, observations of CHW training events, and group discussions with intervention staff. - Thematic analysis, following the Braun and Clarke (2014) approach, was conducted by co-authors in Zambia and South Africa, involving steps such as reading through transcripts, identifying key themes, refining themes collaboratively, and clarifying data with the data collection teams when needed. - The study focused on aspects such as recruitment, retention, training, and support of CHWs to assess fidelity to the intervention design and provide insights for potential transferability to other settings |
| **Intervention description** | - The intervention involved deploying a cadre of 737 study-specific Community Health Workers (CHWs) in Zambia and South Africa to deliver a 'universal' door-to-door HIV prevention package, including an annual offer of HIV testing. - Supervisors conducted weekly meetings with CHWs to monitor progress and provide support, with monthly accompanied visits to ensure protocol adherence and address challenges like reluctant clients and data quality issues. - CHWs received regular monthly refresher training sessions focusing on frontline challenges like client communication and data integrity, with additional training between annual intervention rounds and ad hoc sessions for site-specific issues. - The intervention implementation was guided by routine primary healthcare indicators, and data from the trial was used to document recruitment, retention, training, and support of CHWs, ensuring fidelity to the intervention design and providing insights for future implementations. |
| **Intervention outcomes** | - The intervention led to increases in the proportion of people living with HIV (PLHIV) who knew their HIV status and were on antiretroviral therapy (ART) in the intervention arms where Community-based Health Workers (CHWs) were active. - These increases in HIV testing and treatment resulted in higher viral suppression and lower HIV incidence in the intervention arms compared to control arms. - CHWs played a crucial role in supporting the scale-up of Universal Test and Treat (UTT) in intervention communities in Zambia and South Africa. - Over time, many challenges faced during the initial rounds of implementing the intervention were addressed, either through increased support or collaboration with community engagement staff, fostering positive regard for CHWs by the communities they served. - Supervisors had regular meetings with CHWs to monitor progress, provide support, conduct quality control visits, and address challenges related to reluctant clients, data capturing issues, and difficulties reaching study targets. |
| **Challenges and limitations** | - CHWs faced challenges related to physical and emotional strain due to the demanding nature of their work, leading to concerns about burnout and absenteeism. - Absenteeism among CHWs in Zambia was common, especially during physically taxing conditions like the rainy season and hot summer days. - In South Africa, the lack of prior work experience of many CHWs resulted in issues such as low work ethic, unauthorized leave, and sudden resignations. - Challenges included low morale, disciplinary issues, staff turnover, failure to meet targets, and data quality problems, necessitating responsive management strategies. - Despite efforts to address challenges, the physically and emotionally demanding nature of the CHWs' roles required ongoing support and management to maintain morale and ensure effective intervention delivery. |

**Table 1 – Context**

| **Including (but not limited to) beliefs, social and cultural norms, regulations and economic factors (2,3). For example:**   - Individual capabilities (i.e. values, roles, knowledge, purpose). - Interpersonal relationships supporting the interventions (i.e. communication, collaboration, network, influences). - Institutional settings (i.e. informal rules, organisational culture, leadership, policies, resource allocation, local priorities). - Infra-structural system (i.e. political support). - Country and rurality (i.e. small or large/rural vs regional vs remote). - Socio-demographic characteristics: - Funding context or source (i.e. free, personalised, group vs. individual, government funded). |
| --- |
| - **Individual Capabilities:**   - The study communities' responses to the household-based intervention were influenced by the level and comparison of knowledge of HIV status among adults following contact with the intervention. - **Interpersonal Relationships:**   - The presence and participation of men, as well as the communication and collaboration within the communities, played a role in the uptake of the intervention. - **Institutional Settings:**   - Factors such as the informal economy, community leadership, and local priorities influenced the response to the intervention, highlighting the importance of organizational culture and resource allocation. - **Infrastructural System:**   - Political support within the communities was crucial for the success of the interventions, indicating that the support at the institutional level impacted the outcomes. - **Country and Rurality:**   - Variances in responses were observed between small and large communities, rural versus regional settings, and remote areas, showcasing the impact of country and rurality on intervention outcomes. - **Socio-demographic Characteristics:**   - Socio-demographic factors such as population size and composition influenced the uptake of interventions, demonstrating the significance of understanding the community's demographic profile. - **Funding Context:**   - The research was funded by various organizations, including the International Initiative for Impact Evaluation and the Bill and Melinda Gates Foundation, highlighting the diverse funding sources that can impact the implementation and success of interventions.   Bond et al. 2016 |

**Table 2 – Mechanisms**

| - **“An element of reasoning and/or reactions of an individual or collective agents(s) in regard of resources available in a given context to bring about changes through the implementation of an intervention” (4). Check what are the mediators to produce outcomes (not only primary, but also secondary or unintended outcomes).** |
| --- |
| - **Training and Support:** Adequate and regular training, along with consistent emotional and moral support, helped CHWs better adapt and prepare for the demanding work associated with community-based care. - **Supervision and Quality Control:** Supervisors conducted weekly meetings and monthly accompanied visits to ensure CHWs were implementing interventions correctly, providing immediate feedback and support. - **Addressing Challenges:** Managers had to be responsive to daily challenges faced by CHWs, such as absenteeism, low morale, disciplinary issues, and data quality challenges, to maintain intervention effectiveness. - **Collaborative Approach:** The collaboration between study-specific CHWs and government-employed CHWs in delivering a universal HIV prevention package through door-to-door allowed for the leveraging of existing CHW structures and community-specific needs services, leading to successful implementation of the intervention. - **Adaptation and Preparedness:** By addressing concerns and providing necessary resources, CHWs were better adapted and prepared to conduct the demanding work associated with HIV prevention and treatment services, leading to improved outcomes in the study. |

**Table 3 – Outcomes**

| - **Please make note of all (intended or not) outcomes. Please also classify the role and functions outcomes of CHWs using the following Pillars for people-centred care:**   1. Engaging and empowering people and communities  2. Strengthening governance and accountability (Incl. Supervision, training, and support)  3. Reorienting the model of care  4. Coordinating services within and across sectors  5. Enabling environment |
| --- |
| - The trial evaluated the effect of a combination prevention intervention on HIV incidence, showing a 30% lower incidence in group B compared to standard care, while group A did not exhibit a significant reduction in HIV incidence. - Viral suppression rates at 24 months were 71.9% in group A, 67.5% in group B, and 60.2% in group C. - The estimated percentage of HIV-positive adults receiving ART at 36 months was 81% in group A and 80% in group B. - Group B had a significantly lower HIV incidence compared to group C, with a rate ratio of 0.70 (P = 0.006), while group A did not show a significant difference compared to group C. - The intervention had a greater effect on HIV incidence in group B compared to group C across all matched triplets, with a permutation test showing strong evidence of this effect (P < 0.001). **Hayes et al. 2019** - The intervention led to increases in the proportion of people living with HIV (PLHIV) who knew their HIV status and were on antiretroviral therapy (ART) in the intervention arms where Community-based Health Workers (CHWs) were active. - These increases in HIV testing and treatment resulted in higher viral suppression and lower HIV incidence in the intervention arms compared to control arms. - CHWs played a crucial role in supporting the scale-up of Universal Test and Treat (UTT) in intervention communities in Zambia and South Africa. - Over time, many challenges faced during the initial rounds of implementing the intervention were addressed, either through increased support or collaboration with community engagement staff, fostering positive regard for CHWs by the communities they served. - Supervisors had regular meetings with CHWs to monitor progress, provide support, conduct quality control visits, and address challenges related to reluctant clients, data capturing issues, and difficulties reaching study targets. **Viljoen et al. 2021**   **Outcomes according to PCC:**   - **Engaging and empowering people and communities:**   - Community-based health workers (CHWs) can engage communities effectively by providing door-to-door HIV prevention services, including testing, which was demonstrated in the HPTN 071 (PopART) trial in Zambia and South Africa.   - Integrating HIV services into broader health screenings can empower communities and enhance the reach of services, promoting engagement and ownership of health initiatives. - **Strengthening governance and accountability (Incl. Supervision, training, and support):**   - Challenges such as absenteeism, low morale, and staff turnover were addressed through responsive management strategies, emphasizing the importance of supportive supervision, and addressing daily challenges faced by CHWs.   - Implementing layered management structures, regular training, and emotional support improved CHWs' preparedness and adaptation to the demanding nature of community-based care, enhancing governance and accountability. - **Reorienting the model of care:**   - By incorporating HIV services into existing health systems and expanding training to address community concerns, the model of care can be reoriented to be more inclusive and responsive to local needs. - **Coordinating services within and across sectors:**   - CHWs can facilitate coordination by providing a range of services beyond HIV testing, care, and referrals, contributing to a more integrated approach to healthcare delivery. - **Enabling environment:**   - Adequate training, supervision, and emotional support create an enabling environment for CHWs to effectively deliver services, highlighting the importance of supportive structures in enhancing community-based care initiatives. |

**Table 4 – If, then statements depicting the CMOs.**

| **Please detail the CMOs for the study you reviewed using the If (Context), Then (Mechanism) statements.** |
| --- |
| **If Then statements:**   - If CHWs receive adequate and regular training, along with consistent emotional and moral support, then CHWs will feel prepared, competent, and confident in their roles, enabling them to deliver high-quality services effectively. - If CHW supervisors conducted weekly meetings and monthly visits to ensure that CHWs were implementing interventions correctly and provided immediate feedback and support, then they would develop a sense of accountability and responsibility for their actions and performance. This is because the regular interactions and oversight from supervisors establish a structured framework in which CHWs are accountable for their duties and actions. Consequently, CHWs will take ownership of their role in community health initiatives, striving to meet expectations, which will lead to improved overall performance and outcomes. - If study-specific CHWs collaborate with government-employed CHWs in delivering a universal HIV prevention package through door-to-door services, then existing CHW structures will be leveraged and community specific needs will be addressed, leading to a successful implementation of the intervention.   If there is a layered management structure (intervention managers, district managers, and supervisors) coordinating the activities of CHWs, monitoring their performance, providing ongoing training and support, and ensuring that healthcare interventions are implemented effectively and efficiently in the community, then CHWs will feel prepared and equipped to carry out their duties efficiently and develop a sense of accountability. This will lead to improved healthcare delivery outcomes and enhanced governance and accountability within the healthcare system. |

**Test for relevance (Pearson et al. 2012; 2015; Brennan et al. 2017)**

| **Conceptually Rich** | **Thicker description’ but not ‘conceptually rich’** | **Conceptually Thin** |
| --- | --- | --- |
| Unambiguous theoretical concepts are described in sufficient depth. | Description of programme theory or sufficient information to enable it to ‘surface’. | Insufficient information to enable the programme theory to surface. |
| Relationships between, amongst concepts are clearly articulated. | Consideration of the context in which the programme takes place. | Limited or no consideration of the context in which the programme took place. |
| Concepts are sufficiently developed, defined to enable understanding without the reader needing to have first-hand experience of an area of practice. | Discussion of the differences between the design and orientation of programme theory (what was intended) and implementation (what really happened). | Limited or no discussion of the differences between the design and orientation of programme theory (what was intended) and implementation (what really happened). |
| Concepts are grounded strongly in a cited body of literature. | Recognition and discussion of the strengths/weaknesses of the implemented programme. | Limited or no discussion of the strengths/ weaknesses of the implemented programme. |
| Concepts are parsimonious (i.e., provide the simplest, but not over-simplified, explanation) | Some attempt to explain anomalous results and findings with reference to context and data. | No attempts to explain anomalous results and findings with reference to context and data. |
|  | Description of the factor affecting implementation. | Limited or no description of the factors affecting implementation. |

**Test for relevance (Pearson et al. 2012; 2015; Brennan et al. 2017)**

| **Conceptually Rich** | **Thicker description’ but not ‘conceptually rich’** | **Conceptually Thin** |
| --- | --- | --- |
|  | X |  |

**Test for rigour (Ohly et al. 2017)**

|  | **Yes** | **Fairly** | **No** |
| --- | --- | --- | --- |
| The study methods are clearly reported. | X |  |  |
| The study methods are appropriate to answer RQ. | X |  |  |
| The sample characteristics enable generalizability. | X |  |  |
| Raw data supports the study findings (conclusions). | X |  |  |
| Limitations of the study are acknowledged and clearly reported. | X |  |  |

**References**

1. Wong G, Westhorp G, Manzano A, Greenhalgh J, Jagosh J, Greenhalgh T. (2016). RAMESES II

reporting standards for realist evaluations. *BMC Medicine*, 14(1), 1-18.

2. Wong G, Greenhalgh T, Westhorp G, Pawson R. (2012). Realist methods in medical education

research: what are they and what can they contribute? *Medical Education*, 46(1), 89-96.

3. Macfarlane F, Greenhalgh T, Humphrey C, Hughes J, Butler C, Pawson R. (2011). A new workforce

in the making?: A case study of strategic human resource management in a whole-system change

effort in healthcare. *Journal of Health Organization and Management*, 25(1), 55-72.

4. Lacouture A, Breton E, Guichard A, Ridde V. (2015). The concept of mechanism from a realist

approach: a scoping review to facilitate its operationalization in public health program evaluation.

*Implementation Science*, 10(1), 1-10.

5. Pearson M, Chilton R, Woods HB, Wyatt K, Ford T, Abraham C, et al. Implementing health promotion in schools: protocol for a realist systematic review of research and experience in the United Kingdom (UK). Syst Rev. 2012 Oct 20;1(1):48.

6. Pearson M, Brand SL, Quinn C, Shaw J, Maguire M, Michie S, et al. Using realist review to inform intervention development: methodological illustration and conceptual platform for collaborative care in offender mental health. Implementation Sci. 2015 Sep 28;10(1):134.

7. Brennan N, Bryce M, Pearson M, Wong G, Cooper C, Archer J. Towards an understanding of how appraisal of doctors produces its effects: a realist review. Medical Education. 2017;51(10):1002–13.

8. Ohly H, Crossland N, Dykes F, Lowe N, Hall-Moran V. A realist review to explore how low-income pregnant women use food vouchers from the UK’s Healthy Start programme. BMJ Open. 2017 Apr 21;7(4):e013731.

**A realist evaluation of the role and functions of community health workers in service of a people-centred community health system.**

**Investigator CMOc Data Extraction Sheet Data Extraction**

The purpose of this document is to extract data from the identified studies in order to formulate one or more explanatory theories that explain Context-Mechanism-Outcome Configurations (CMOC) and to gain insight into how these CMOCs contribute to optimizing the role and functions of community health workers within a people-centred community health system.

In realist methodologies, knowledge accumulation centres on our comprehension of how mechanisms behave in various contexts and the reasons behind how and why this interplay of context and mechanism leads to different outcomes (1). Please fill in the details below for the study you will be assessing. In the subsequent three tables, locate and specify the Context, Mechanism, and Outcomes (CMO) of the intervention discussed in the study you've examined. Definitions and illustrations of CMO are provided within the tables.

**Kindly fill out the information below for the study you will be reviewing.**

| **Reviewer Name** | Usangiphile Buthelezi |
| --- | --- |
| **Reference** | Wanduru P, Tetui M, Tuhebwe D, Ediau M, Okuga M, Nalwadda C, et al. The performance of community health workers in the management of multiple childhood infectious diseases in Lira, northern Uganda – a mixed methods cross-sectional study. Glob Health Action. 2016 Nov 22;9:10.3402/gha.v9.33194. |
| **Country** | Uganda |
| **Setting and population** | **Study Setting:**   - The study was conducted in the Lira district, northern Uganda. Lira has a population of about 368,100 people and is divided into four counties: Erute north, Erute south, Moroto, and Lira municipal. Each county is a health sub-district, and below that, the administration structure includes sub-counties, parishes, and villages. Community health workers (CHWs) serve at the village level.   **Population:**   - The study included 393 eligible CHWs in the Lira district of Uganda. - The respondents in the study were CHWs who had attained at least a primary level of education, with an almost equal distribution of females and males. |
| **Aim** | The aim of the study was to assess the performance of community health workers (CHWs) in managing malaria, pneumonia, and diarrhea in the rural district of Lira, northern Uganda. |
| **Objectives** | - The study aimed to determine the factors associated with CHW performance and identify potential solutions to improve their performance. |
| **Methodology** | - The study utilized a cross-sectional mixed methods design to assess the performance of community health workers (CHWs) in managing childhood infectious diseases in Lira, northern Uganda. - A total of 393 eligible CHWs were included in the study. Case scenarios were conducted with a medical officer observing CHWs in their management of children suspected of having malaria, pneumonia, or diarrhea. - Performance data were collected using a pretested questionnaire with a checklist used by the medical officer to score the CHWs. - The performance of CHWs was assessed based on their ability to diagnose and treat malaria, diarrhea, and pneumonia appropriately. - Participants were categorized into three groups based on their performance scores: good, moderate, and poor. - A binary measure of performance (good vs. poor) was used in multivariable logistic regression to analyze the association between performance and independent variables. - In addition to the quantitative component, a qualitative component was included in the study. - Seven key informant interviews were conducted with experts who had informed knowledge regarding the functionality of CHWs in Lira district. - Thematic data analysis was used to develop themes from the transcripts, which complemented the findings from the quantitative analysis. |
| **Intervention description** | - The intervention in focus was the use of community health workers (CHWs) to manage childhood infectious diseases in the rural district of Lira, northern Uganda. - CHWs were trained individuals who provide basic healthcare services, including diagnosing and treating malaria, pneumonia, and diarrhea in children. - The study assessed the performance of CHWs in managing these diseases and identified factors associated with their performance. |
| **Intervention outcomes** | - The performance of community health workers (CHWs) in managing childhood infectious diseases in Lira, northern Uganda was assessed. Overall, 88.3% of CHWs had poor scores, 6.6% had moderate scores, and 5.1% had good scores in managing malaria, diarrhea, and pneumonia. - Factors positively associated with CHW performance included secondary-level education and regular meetings with supervisors. - Factors negatively associated with performance included serving a high number of households and shorter initial training duration. - The qualitative findings reinforced the quantitative results and highlighted the importance of refresher training, workload management, and in-kind incentives in improving CHW performance. - Recommendations to improve CHW performance included pre-qualification testing, ongoing support and supervision, secondary education for CHWs, and moderation of their workload. |
| **Challenges and limitations** | - The study only focused on the Lira district in northern Uganda, limiting the generalizability of the findings to other regions. - The study used a cross-sectional design, providing a snapshot of CHW performance at a specific point in time and may not capture long-term trends or changes. - The study relied on self-reported data from CHWs, which may be subject to recall bias or social desirability bias. - The study did not assess the impact of other potential factors, such as availability of resources or community support, on CHW performance. |

**Table 1 – Context**

| **Including (but not limited to) beliefs, social and cultural norms, regulations and economic factors (2,3). For example:**   - Individual capabilities (i.e. values, roles, knowledge, purpose). - Interpersonal relationships supporting the interventions (i.e. communication, collaboration, network, influences). - Institutional settings (i.e. informal rules, organisational culture, leadership, policies, resource allocation, local priorities). - Infra-structural system (i.e. political support). - Country and rurality (i.e. small or large/rural vs regional vs remote). - Socio-demographic characteristics: - Funding context or source (i.e. free, personalised, group vs. individual, government funded). |
| --- |
| **Study Setting:**   - The study was conducted in the Lira district, northern Uganda. Lira has a population of about 368,100 people and is divided into four counties: Erute north, Erute south, Moroto, and Lira municipal. Each county is a health sub-district, and below that, the administration structure includes sub-counties, parishes, and villages. Community health workers (CHWs) serve at the village level.   **Population:**   - The study included 393 eligible CHWs in the Lira district of Uganda. - The respondents in the study were CHWs who had attained at least a primary level of education, with an almost equal distribution of females and males. |

**Table 2 – Mechanisms**

| - **“An element of reasoning and/or reactions of an individual or collective agents(s) in regard of resources available in a given context to bring about changes through the implementation of an intervention” (4). Check what are the mediators to produce outcomes (not only primary, but also secondary or unintended outcomes).** |
| --- |
| - **Perceptions and knowledge:** CHWs with secondary-level education were more likely to have better performance, indicating that higher education levels contribute to improved knowledge and understanding of disease management. - **Motivation and satisfaction:** Regular meetings with supervisors were positively associated with better CHW performance, suggesting that ongoing support and supervision can motivate CHWs and enhance their job satisfaction. - **Rapport, trust, and recognition:** CHWs who had met with their supervisors in the previous month were more likely to have better performance, indicating that regular interactions and recognition from supervisors can build rapport, trust, and motivation among CHWs. - **Values and value clarification:** CHWs who had shorter initial training durations (2-3 days) were less likely to perform well, suggesting that a longer training period may be necessary for CHWs to clarify their values and develop a deeper understanding of their roles. - **Communication and social connection:** Refresher training and regular meetings were identified as important determinants of performance by key informants, highlighting the importance of continuous communication and social connection in maintaining CHWs' knowledge and skills |

**Table 3 – Outcomes**

| - **Please make note of all (intended or not) outcomes. Please also classify the role and functions outcomes of CHWs using the following Pillars for people-centred care:**   1. Engaging and empowering people and communities  2. Strengthening governance and accountability (Incl. Supervision, training, and support)  3. Reorienting the model of care  4. Coordinating services within and across sectors  5. Enabling environment |
| --- |
| - The performance of community health workers (CHWs) in managing childhood infectious diseases in Lira, northern Uganda was assessed. Overall, 88.3% of CHWs had poor scores, 6.6% had moderate scores, and 5.1% had good scores in managing malaria, diarrhea, and pneumonia. - Factors positively associated with CHW performance included secondary-level education and regular meetings with supervisors. - Factors negatively associated with performance included serving a high number of households and shorter initial training duration. - The qualitative findings reinforced the quantitative results and highlighted the importance of refresher training, workload management, and in-kind incentives in improving CHW performance. - Recommendations to improve CHW performance included pre-qualification testing, ongoing support and supervision, secondary education for CHWs, and moderation of their workload.   **Engaging and empowering people and communities:**   - The performance of CHWs in managing childhood infectious diseases in Lira, Uganda was inadequate, indicating a need for interventions to engage and empower CHWs to improve their performance. - Regular meetings with supervisors were positively associated with better CHW performance, suggesting that ongoing support and supervision can empower CHWs and enhance their job satisfaction. - Refresher training and regular meetings were identified as important determinants of performance, highlighting the importance of continuous communication and social connection in engaging CHWs and enabling them to provide better care.   **Strengthening governance and accountability (Incl. Supervision, training, and support):**   - Meeting with supervisors in the previous month was positively associated with CHW performance, indicating the importance of supervision in strengthening governance and accountability. - Providing ongoing support and supervision, as well as ensuring that CHWs have at least secondary education, can be helpful in improving their performance and strengthening governance. - Short training programs were found to be beneficial to some degree, but they should be followed up with regular refresher training to enhance CHW performance and ensure accountability.   **Reorienting the model of care:**   - The study focused on assessing the performance of CHWs in managing malaria, pneumonia, and diarrhea, indicating the need to reorient the model of care towards community-based management of these childhood infectious diseases.   **Coordinating services within and across sectors:**   - The study did not explicitly address the coordination of services within and across sectors in the context of CHW performance.   **Enabling environment:**   - The study highlighted the importance of workload moderation for CHWs, as work overload can reduce their performance, indicating the need for an enabling environment that supports manageable workloads for CHWs. |

**Table 4 – If, then statements depicting the CMOs.**

| **Please detail the CMOs for the study you reviewed using the If (Context), Then (Mechanism) statements.** |
| --- |
| **If, Then statements**   - If there is ongoing support and supervision of CHWs by supervisors, then CHWs will feel motivated, valued, and empowered to carry out their tasks effectively, leading to increased job satisfaction, higher levels of engagement, and improved quality of healthcare delivery. - If there are regular interactions between CHWs and their supervisors, then CHWs perform better indicating that regular interactions and recognition from supervisors can build rapport, trust, and motivation among CHWs, ultimately resulting in improved healthcare outcomes for the community. |

**Test for relevance (Pearson et al. 2012; 2015; Brennan et al. 2017)**

| **Conceptually Rich** | **Thicker description’ but not ‘conceptually rich’** | **Conceptually Thin** |
| --- | --- | --- |
| Unambiguous theoretical concepts are described in sufficient depth. | Description of programme theory or sufficient information to enable it to ‘surface’. | Insufficient information to enable the programme theory to surface. |
| Relationships between, amongst concepts are clearly articulated. | Consideration of the context in which the programme takes place. | Limited or no consideration of the context in which the programme took place. |
| Concepts are sufficiently developed, defined to enable understanding without the reader needing to have first-hand experience of an area of practice. | Discussion of the differences between the design and orientation of programme theory (what was intended) and implementation (what really happened). | Limited or no discussion of the differences between the design and orientation of programme theory (what was intended) and implementation (what really happened). |
| Concepts are grounded strongly in a cited body of literature. | Recognition and discussion of the strengths/weaknesses of the implemented programme. | Limited or no discussion of the strengths/ weaknesses of the implemented programme. |
| Concepts are parsimonious (i.e., provide the simplest, but not over-simplified, explanation) | Some attempt to explain anomalous results and findings with reference to context and data. | No attempts to explain anomalous results and findings with reference to context and data. |
|  | Description of the factor affecting implementation. | Limited or no description of the factors affecting implementation. |

**Test for relevance (Pearson et al. 2012; 2015; Brennan et al. 2017)**

| **Conceptually Rich** | **Thicker description’ but not ‘conceptually rich’** | **Conceptually Thin** |
| --- | --- | --- |
| X |  |  |

**Test for rigour (Ohly et al. 2017)**

|  | **Yes** | **Fairly** | **No** |
| --- | --- | --- | --- |
| The study methods are clearly reported. | X |  |  |
| The study methods are appropriate to answer RQ. | X |  |  |
| The sample characteristics enable generalizability. |  |  | X |
| Raw data supports the study findings (conclusions). | X |  |  |
| Limitations of the study are acknowledged and clearly reported. | X |  |  |

**References**

1. Wong G, Westhorp G, Manzano A, Greenhalgh J, Jagosh J, Greenhalgh T. (2016). RAMESES II

reporting standards for realist evaluations. *BMC Medicine*, 14(1), 1-18.

2. Wong G, Greenhalgh T, Westhorp G, Pawson R. (2012). Realist methods in medical education

research: what are they and what can they contribute? *Medical Education*, 46(1), 89-96.

3. Macfarlane F, Greenhalgh T, Humphrey C, Hughes J, Butler C, Pawson R. (2011). A new workforce

in the making?: A case study of strategic human resource management in a whole-system change

effort in healthcare. *Journal of Health Organization and Management*, 25(1), 55-72.

4. Lacouture A, Breton E, Guichard A, Ridde V. (2015). The concept of mechanism from a realist

approach: a scoping review to facilitate its operationalization in public health program evaluation.

*Implementation Science*, 10(1), 1-10.

5. Pearson M, Chilton R, Woods HB, Wyatt K, Ford T, Abraham C, et al. Implementing health promotion in schools: protocol for a realist systematic review of research and experience in the United Kingdom (UK). Syst Rev. 2012 Oct 20;1(1):48.

6. Pearson M, Brand SL, Quinn C, Shaw J, Maguire M, Michie S, et al. Using realist review to inform intervention development: methodological illustration and conceptual platform for collaborative care in offender mental health. Implementation Sci. 2015 Sep 28;10(1):134.

7. Brennan N, Bryce M, Pearson M, Wong G, Cooper C, Archer J. Towards an understanding of how appraisal of doctors produces its effects: a realist review. Medical Education. 2017;51(10):1002–13.

8. Ohly H, Crossland N, Dykes F, Lowe N, Hall-Moran V. A realist review to explore how low-income pregnant women use food vouchers from the UK’s Healthy Start programme. BMJ Open. 2017 Apr 21;7(4):e013731.

**A realist evaluation of the role and functions of community health workers in service of a people-centred community health system.**

**Investigator CMOc Data Extraction Sheet Data Extraction**

The purpose of this document is to extract data from the identified studies in order to formulate one or more explanatory theories that explain Context-Mechanism-Outcome Configurations (CMOC) and to gain insight into how these CMOCs contribute to optimizing the role and functions of community health workers within a people-centred community health system.

In realist methodologies, knowledge accumulation centres on our comprehension of how mechanisms behave in various contexts and the reasons behind how and why this interplay of context and mechanism leads to different outcomes (1). Please fill in the details below for the study you will be assessing. In the subsequent three tables, locate and specify the Context, Mechanism, and Outcomes (CMO) of the intervention discussed in the study you've examined. Definitions and illustrations of CMO are provided within the tables.

**Kindly fill out the information below for the study you will be reviewing.**

| **Reviewer Name** | Usangiphile Buthelezi |
| --- | --- |
| **Reference** | Youngui BT, Atwine D, Otai D, Vasiliu A, Ssekyanzi B, Sih C, et al. Integration of HIV Testing in a Community Intervention for Tuberculosis Screening Among Household Contacts of Patients with Tuberculosis in Cameroon and Uganda. J Acquir Immune Defic Syndr. 2024 Apr 15;95(5):431–8. |
| **Country** | Cameroon and Uganda |
| **Setting and population** | - The study took place in Cameroon and Uganda as part of the CONTACT study, focusing on TB management. - It targeted household contacts of patients with confirmed TB cases who were older than 15 years. - The intervention was carried out in 9 clusters that included health facilities with TB diagnostic and treatment capabilities. - The participants included all contacts aged 5 years or older who were enrolled in the intervention arm of the CONTACT study. |
| **Aim** | Aim to integrate HIV testing into community-based household TB contact screening in Cameroon and Uganda |
| **Objectives** | - Objectives include improving access to HIV care for TB-exposed individuals, enhancing follow-up visits based on household size, and ensuring linkage to treatment services for TB-exposed people living with HIV (PLHIV). - Focus on increasing acceptance rates of the integrated approach among household contacts aged 5 years and older. |
| **Methodology** | - The study employed a **community-based intervention** model, where trained community health workers (CHWs) and community nurses (CNs) conducted home visits to provide TB screening and HIV testing to household contacts of TB patients. - **Participants** included household contacts aged **5 years and older** who were identified during the TB screening process. A total of **2206 eligible contacts** were identified, with **1983** (89.9%) enrolled in the study. - **HIV testing** was offered to contacts with unknown HIV status, utilizing rapid test kits (Alere Determine VIH-1/2) recommended by national programs. Testing was conducted only with consent, ensuring confidentiality and privacy during the process. - The **cascade of care** for HIV testing was analyzed, tracking the number of contacts who accepted testing, were tested, and subsequently linked to care if positive. - **Statistical analysis** involved generalized linear mixed models to assess factors influencing the acceptance of HIV testing, with adjustments for age, country, and other relevant variables |
| **Intervention description** | - Integration of HIV testing into community-based household TB contact screening was the main focus of the intervention. - The intervention targeted household contacts aged 5 years and older to increase access to HIV testing and identify TB-exposed PLHIV who were unaware of their HIV status |
| **Intervention outcomes** | - Integrating voluntary HIV testing and counseling into a community-based intervention for household TB contact management in Cameroon and Uganda led to 88.2% of household contacts aged 5 years or older learning their HIV status. - Furthermore, this integration identified 20 TB-exposed PLHIV who were unaware of their HIV status, successfully linking them to treatment services. - The integration of HIV testing with household TB contact screening increased access to HIV care and TB prevention services among TB contacts. - Implementing the intervention did not show statistically significant associations between household size, geographic location, or the type of familiar relationship and VCT acceptance, indicating acceptability across various settings and family structures. |
| **Challenges and limitations** | - The study did not address potential challenges or barriers to implementing the intervention, which could impact its scalability and sustainability. - Limited information was provided on the specific characteristics of the household contacts, such as socio-economic status, education level, or access to healthcare, which could influence the outcomes of the intervention. - The study focused on two countries (Cameroon and Uganda), and the findings may not be generalizable to other regions or settings. - There was no discussion on the long-term effectiveness or follow-up of the integrated intervention, raising questions about the durability of the outcomes seen in the short term. |

**Table 1 – Context**

| **Including (but not limited to) beliefs, social and cultural norms, regulations and economic factors (2,3). For example:**   - Individual capabilities (i.e. values, roles, knowledge, purpose). - Interpersonal relationships supporting the interventions (i.e. communication, collaboration, network, influences). - Institutional settings (i.e. informal rules, organisational culture, leadership, policies, resource allocation, local priorities). - Infra-structural system (i.e. political support). - Country and rurality (i.e. small or large/rural vs regional vs remote). - Socio-demographic characteristics: - Funding context or source (i.e. free, personalised, group vs. individual, government funded). |
| --- |
| **Study Setting and Population**   - The study took place in Cameroon and Uganda as part of the CONTACT study, focusing on TB management. - It targeted household contacts of patients with confirmed TB cases who were older than 15 years. - The intervention was carried out in 9 clusters that included health facilities with TB diagnostic and treatment capabilities. - The participants included all contacts aged 5 years or older who were enrolled in the intervention arm of the CONTACT study.   **Training and Education Level**   - Community health workers (CHWs) and community nurses (CNs) who provided services underwent training to deliver TB and HIV services. - The CHWs and CNs were supervised by the cluster facility TB focal person during the intervention.   **Available Resources and Funding**   - The community intervention was supported by the CaP-TB UNITAID funded project in both countries. - CHWs and CNs utilized resources within the communities to provide services at the households of TB contacts.   **Facilities and Economic Status**   - Facilities utilized for the study included district hospitals in Cameroon and primary health centres or district hospitals in Uganda. - The study was conducted in resource-limited settings with a focus on high TB burden areas.   **Education Level of Community Health Workers**   - The CHWs involved in the study were trained individuals who provided services during home visits to TB contacts. - Their education level was not specified in the provided context.   **Challenges and Contextual Issues**   - Community-based approaches, although effective, can face challenges related to access to care and resource limitations. - Integrating HIV testing into TB screening at household visits presented a feasible approach despite contextual issues. |

**Table 2 – Mechanisms**

| - **“An element of reasoning and/or reactions of an individual or collective agents(s) in regard of resources available in a given context to bring about changes through the implementation of an intervention” (4). Check what are the mediators to produce outcomes (not only primary, but also secondary or unintended outcomes).** |
| --- |
| **Building trust and rapport**   - Establishing a **positive relationship** may have made individuals more willing to accept HIV testing during TB screening.   **Motivation and Empowerment**   - Empowering TB contacts with knowledge about HIV testing and TB prevention may have increased their engagement with the intervention.   **Competence and Credibility**   - The credibility of the CHWs could have influenced the trust that TB contacts placed in the intervention.   **Recognition and Support**   - Feeling supported and valued within the community could have motivated CHWs to effectively deliver services during home visits.   **Accountability and Confidence**   - Holding CHWs accountable for their tasks and ensuring they felt confident in their roles likely impacted the intervention's outcomes. - Confidence in the intervention may have influenced the willingness of household contacts to engage in HIV testing.   **Cultural Sensitivity**   - Considering cultural norms and practices may have played a role in overcoming potential barriers to HIV testing. |

**Table 3 – Outcomes**

| - **Please make note of all (intended or not) outcomes. Please also classify the role and functions outcomes of CHWs using the following Pillars for people-centred care:**   1. Engaging and empowering people and communities  2. Strengthening governance and accountability (Incl. Supervision, training, and support)  3. Reorienting the model of care  4. Coordinating services within and across sectors  5. Enabling environment |
| --- |
| **Outcomes according to PCC pillars:**   - **Engaging and empowering people and communities:** The integration of HIV testing with household TB contact screening in Cameroon and Uganda showed high acceptance rates among household contacts aged 5 years or older, allowing them to learn their HIV status, which facilitated access to care services. - **Strengthening governance and accountability:** The community intervention implemented in the CONTACT study provided comprehensive support to contacts, including scheduling follow-up visits to complete screenings, regardless of household size, demonstrating a robust approach to ensure participation. - **Reorienting the model of care:** The model described in the study integrated voluntary HIV testing and counselling into a community-based intervention, improving access to HIV care and TB prevention services for TB contacts. - **Coordinating services within and across sectors:** The integrated approach in the study enabled the identification of TB-exposed persons living with HIV who were unaware of their status, successfully linking them to treatment services, highlighting effective coordination among health services. - **Enabling environment:** HIV testing was seamlessly integrated into the community-based household TB contact screening, demonstrating high acceptance rates and successful linkage to care services, indicating a conducive environment for service integration. |

**Table 4 – If, then statements depicting the CMOs.**

| **Please detail the CMOs for the study you reviewed using the If (Context), Then (Mechanism) statements.** |
| --- |
|  |

**Test for relevance (Pearson et al. 2012; 2015; Brennan et al. 2017)**

| **Conceptually Rich** | **Thicker description’ but not ‘conceptually rich’** | **Conceptually Thin** |
| --- | --- | --- |
| Unambiguous theoretical concepts are described in sufficient depth. | Description of programme theory or sufficient information to enable it to ‘surface’. | Insufficient information to enable the programme theory to surface. |
| Relationships between, amongst concepts are clearly articulated. | Consideration of the context in which the programme takes place. | Limited or no consideration of the context in which the programme took place. |
| Concepts are sufficiently developed, defined to enable understanding without the reader needing to have first-hand experience of an area of practice. | Discussion of the differences between the design and orientation of programme theory (what was intended) and implementation (what really happened). | Limited or no discussion of the differences between the design and orientation of programme theory (what was intended) and implementation (what really happened). |
| Concepts are grounded strongly in a cited body of literature. | Recognition and discussion of the strengths/weaknesses of the implemented programme. | Limited or no discussion of the strengths/ weaknesses of the implemented programme. |
| Concepts are parsimonious (i.e., provide the simplest, but not over-simplified, explanation) | Some attempt to explain anomalous results and findings with reference to context and data. | No attempts to explain anomalous results and findings with reference to context and data. |
|  | Description of the factor affecting implementation. | Limited or no description of the factors affecting implementation. |

**Test for relevance (Pearson et al. 2012; 2015; Brennan et al. 2017)**

| **Conceptually Rich** | **Thicker description’ but not ‘conceptually rich’** | **Conceptually Thin** |
| --- | --- | --- |
|  | **X** |  |

**Test for rigour (Ohly et al. 2017)**

|  | **Yes** | **Fairly** | **No** |
| --- | --- | --- | --- |
| The study methods are clearly reported. | X |  |  |
| The study methods are appropriate to answer RQ. | X |  |  |
| The sample characteristics enable generalizability. |  |  | X |
| Raw data supports the study findings (conclusions). | X |  |  |
| Limitations of the study are acknowledged and clearly reported. | X |  |  |

**References**

1. Wong G, Westhorp G, Manzano A, Greenhalgh J, Jagosh J, Greenhalgh T. (2016). RAMESES II

reporting standards for realist evaluations. *BMC Medicine*, 14(1), 1-18.

2. Wong G, Greenhalgh T, Westhorp G, Pawson R. (2012). Realist methods in medical education

research: what are they and what can they contribute? *Medical Education*, 46(1), 89-96.

3. Macfarlane F, Greenhalgh T, Humphrey C, Hughes J, Butler C, Pawson R. (2011). A new workforce

in the making?: A case study of strategic human resource management in a whole-system change

effort in healthcare. *Journal of Health Organization and Management*, 25(1), 55-72.

4. Lacouture A, Breton E, Guichard A, Ridde V. (2015). The concept of mechanism from a realist

approach: a scoping review to facilitate its operationalization in public health program evaluation.

*Implementation Science*, 10(1), 1-10.

5. Pearson M, Chilton R, Woods HB, Wyatt K, Ford T, Abraham C, et al. Implementing health promotion in schools: protocol for a realist systematic review of research and experience in the United Kingdom (UK). Syst Rev. 2012 Oct 20;1(1):48.

6. Pearson M, Brand SL, Quinn C, Shaw J, Maguire M, Michie S, et al. Using realist review to inform intervention development: methodological illustration and conceptual platform for collaborative care in offender mental health. Implementation Sci. 2015 Sep 28;10(1):134.

7. Brennan N, Bryce M, Pearson M, Wong G, Cooper C, Archer J. Towards an understanding of how appraisal of doctors produces its effects: a realist review. Medical Education. 2017;51(10):1002–13.

8. Ohly H, Crossland N, Dykes F, Lowe N, Hall-Moran V. A realist review to explore how low-income pregnant women use food vouchers from the UK’s Healthy Start programme. BMJ Open. 2017 Apr 21;7(4):e013731.

**A realist evaluation of the role and functions of community health workers in service of a people-centred community health system.**

**Investigator CMOc Data Extraction Sheet Data Extraction**

The purpose of this document is to extract data from the identified studies in order to formulate one or more explanatory theories that explain Context-Mechanism-Outcome Configurations (CMOC) and to gain insight into how these CMOCs contribute to optimizing the role and functions of community health workers within a people-centred community health system.

In realist methodologies, knowledge accumulation centres on our comprehension of how mechanisms behave in various contexts and the reasons behind how and why this interplay of context and mechanism leads to different outcomes (1). Please fill in the details below for the study you will be assessing. In the subsequent three tables, locate and specify the Context, Mechanism, and Outcomes (CMO) of the intervention discussed in the study you've examined. Definitions and illustrations of CMO are provided within the tables.

**Kindly fill out the information below for the study you will be reviewing.**

| **Reviewer Name** | Usangiphile Buthelezi |
| --- | --- |
| **Reference** | Dziva Chikwari C, Simms V, Busza J, Dauya E, Bandason T, Chonzi P, et al. Community health worker support to improve HIV treatment outcomes for older children and adolescents in Zimbabwe: a process evaluation of the ZENITH trial. Implement Sci. 2018 May 23;13:70. |
| **Country** | Zimbambwe |
| **Setting and population** | - The study was conducted in Zimbabwe as part of the ZENITH trial. - The intervention was delivered by community health workers (CHWs) to children living with HIV and their caregivers. - The trial enrolled a total of 172 participants in the intervention arm. - Out of the 334 children recruited into the trial, 166 were randomized to receive the intervention. - The intervention was acceptable for CHWs to deliver, with almost all of them (19 out of 20) retained at the end of the intervention. - The median number of participants allocated per CHW was 9, with a range of 5-15. |
| **Aim** | - The aim of the study was to conduct a process evaluation of the community health worker (CHW)-delivered support visits to children living with HIV and their caregivers in Zimbabwe, as part of the ZENITH trial |
| **Objectives** | - To assess the fidelity, acceptability, and feasibility of the intervention, in order to identify lessons that could inform replication and scale-up of this approach. - To evaluate the implementation of the intervention by analyzing data from field manuals, records from supervisory meetings, and participant data collected throughout the trial. - To assess the acceptability of the intervention to participants and the retention of CHWs in delivering the intervention. - Additionally, the study aimed to explore the feasibility of the intervention, including the number and type of visits conducted, as well as any challenges or barriers encountered during implementation. |
| **Methodology** | - The study conducted a process evaluation of the CHW-delivered support visits to children living with HIV and their caregivers in Zimbabwe as part of the ZENITH trial. - Data for the evaluation was collected from various sources, including field manuals kept by each CHW, records from monthly supervisory meetings, and participant data collected throughout the trial. - The field manuals provided information on visit type, content, and duration, which was used to assess the implementation of the intervention. - Minutes from monthly supervisory meetings were used to capture CHW attendance and retention. - The study also analyzed participant data collected through end line trial questionnaires to assess participant outcomes, such as transfers out from the study catchment area or death, and disclosure of HIV status. - The intervention included activities such as family mapping exercises, review of personal treatment plans, answering questions about HIV treatment, and making referrals for support services. - The study assessed the acceptability and feasibility of the intervention, with high staff retention and fidelity to the intervention identified as key strengths |
| **Intervention description** | - The intervention involved community health workers (CHWs) delivering support visits to children living with HIV and their caregivers in Zimbabwe as part of the ZENITH trial. - The CHWs conducted a total of 1553 visits, with a median of 11 visits per participant. - The visits included activities such as discussing disclosure of HIV status with the child and family, assisting clients with developing and reviewing their personal treatment plans, and answering questions about HIV, its treatment, and monitoring. - The CHWs were provided with field manuals that guided their work and documented their experiences during each visit. - The intervention was found to be acceptable to participants, with most receiving and accepting the required number of visits. - The CHWs received extensive training and ongoing mentorship and support through on-the-job supervision and monthly meetings, which contributed to their retention and the acceptability of the intervention. - The intervention was implemented with high fidelity, as evidenced by the high staff retention and adherence to the intervention activities. - In summary, the intervention involved CHWs conducting support visits to children living with HIV and their caregivers, providing various services and support to improve HIV treatment outcomes. |
| **Intervention outcomes** | - The intervention significantly reduced the odds of virological failure among children living with HIV in the ZENITH trial conducted in Zimbabwe. - Children who received the intervention had significantly reduced treatment failure at 12 months post ART initiation compared to those who received standard HIV care. - The intervention also resulted in a significantly lower proportion of children experiencing a composite outcome of mortality, treatment failure, non-initiation of ART, and loss to follow-up. - The intervention was found to be acceptable and feasible, with high staff retention and fidelity to the intervention. - Participants in the intervention arm received and accepted the required number of visits, indicating their willingness to engage with the CHWs and the intervention. - The intervention showed promise in improving clinical outcomes and reducing virological failure among children living with HIV in Zimbabwe |
| **Challenges and limitations** | - Some CHWs did not complete their manuals fully, which affected the accuracy of data collection and reporting. - The intervention faced challenges in delivering support to participants who had transferred outside the study area, hindering continuity of care. - The intervention's labour-intensive model, with a median of 11 visits per participant, may not be scalable for large populations and national programs. - The inability to definitively ascertain to whom the intervention was delivered and the relationship of caregivers to the child limited the understanding of the intervention's impact. - The study did not provide specific numerical data on the effect size or exact impact of the intervention outcomes. |

**Table 1 – Context**

| **Including (but not limited to) beliefs, social and cultural norms, regulations and economic factors (2,3). For example:**   - Individual capabilities (i.e. values, roles, knowledge, purpose). - Interpersonal relationships supporting the interventions (i.e. communication, collaboration, network, influences). - Institutional settings (i.e. informal rules, organisational culture, leadership, policies, resource allocation, local priorities). - Infra-structural system (i.e. political support). - Country and rurality (i.e. small or large/rural vs regional vs remote). - Socio-demographic characteristics: - Socio-economic status - Funding context or source (i.e. free, personalised, group vs. individual, government funded). |
| --- |
| **Institutional settings:**   - The intervention was implemented within the existing cadre of community health workers, utilizing their pre-existing roles and responsibilities **[2]**. - The intervention required ongoing mentorship, support, and monthly meetings to ensure fidelity and retention of CHWs   **Country and rurality:**   - The study was conducted in Zimbabwe, Harare, a large urban area and the generalizability of the findings to other settings, particularly rural areas, was acknowledged as a limitation   **Socio-demographic characteristics:**   - The study did not provide specific information on the socio-demographic characteristics of the participants or how these factors may have influenced the intervention outcomes. |

**Table 2 – Mechanisms**

| - **“An element of reasoning and/or reactions of an individual or collective agents(s) in regard of resources available in a given context to bring about changes through the implementation of an intervention” (4). Check what are the mediators to produce outcomes (not only primary, but also secondary or unintended outcomes).** |
| --- |
| ***The specific individual mechanisms that made the intervention work were not explicitly mentioned in the article. The information provided below is based on the overall findings and observations from the process evaluation of the intervention.***   - **Family Mapping Exercise:** CHWs conducted a family mapping exercise during the introductory visit, identifying potential support sources like neighbors and school teachers, which likely enhanced rapport and support for the children living with HIV. - **Disclosure and Treatment Planning:** CHWs discussed disclosure with families and assisted in developing personal treatment plans, promoting adherence and improved treatment outcomes. - **Training and Supervision:** Extensive training, ongoing mentorship, and support through monthly supervisory meetings ensured CHWs were well-equipped to deliver the intervention effectively, contributing to positive outcomes. - **Acceptability and Feasibility:** The intervention's acceptability to both participants and CHWs, along with its feasibility, played a crucial role in its successful implementation and subsequent positive outcomes. - **Retention and Fidelity:** High staff retention rates and fidelity to the intervention were key strengths, indicating CHWs' commitment and job satisfaction, which likely contributed to the positive outcomes observed in the trial. |

**Table 3 – Outcomes**

| - **Please make note of all (intended or not) outcomes. Please also classify the role and functions outcomes of CHWs using the following Pillars for people-centred care:**   1. Engaging and empowering people and communities  2. Strengthening governance and accountability (Incl. Supervision, training, and support)  3. Reorienting the model of care  4. Coordinating services within and across sectors  5. Enabling environment |
| --- |
| - The intervention significantly reduced the odds of virological failure among children living with HIV in the ZENITH trial conducted in Zimbabwe. - Children who received the intervention had significantly reduced treatment failure at 12 months post ART initiation compared to those who received standard HIV care. - The intervention also resulted in a significantly lower proportion of children experiencing a composite outcome of mortality, treatment failure, non-initiation of ART, and loss to follow-up. - The intervention was found to be acceptable and feasible, with high staff retention and fidelity to the intervention. - Participants in the intervention arm received and accepted the required number of visits, indicating their willingness to engage with the CHWs and the intervention. - The intervention showed promise in improving clinical outcomes and reducing virological failure among children living with HIV in Zimbabwe   **Intervention outcomes as PCC Pillars:**  **Engaging and empowering people and communities:**   - The community health worker (CHW) support visits were acceptable and feasible for participants, with most receiving and accepting the required number of visits. - CHWs discussed disclosure with the child/family for over 89% of participants and assisted clients with developing and reviewing their personal treatment plan with over 85% of participants.   **Strengthening governance and accountability (Incl. Supervision, training, and support):**   - CHWs received extensive training over 2 weeks followed by 2 weeks of intense on-the-job supervision. Refresher training was conducted after 1 year. [2] - Ongoing mentorship and support through on-the-job supervision and monthly meetings contributed to the high retention of CHWs.   **Reorienting the model of care:**   - The CHW-delivered support visits focused on providing information and resources on HIV and treatment, assisting with disclosure and post-disclosure discussions, following up on issues from clinical monitoring appointments, and providing information on locally available services.   **Coordinating services within and across sectors:**   - Study nurses communicated regularly with CHWs, resulting in unscheduled visits to address issues affecting retention in care and adherence to treatment. This contributed to significantly higher HIV virological suppression in the intervention arm.   **Enabling environment:**   - The intervention provided social protection through psychosocial support and assistance in navigating government systems, which may have contributed to the observed intervention effect. |

**Table 4 – If, then statements depicting the CMOs.**

| **Please detail the CMOs for the study you reviewed using the If (Context), Then (Mechanism) statements.** |
| --- |
| **If, Then statements:**   - If CHWs receive ongoing intensive training, supervision and mentoring (equipping CHWs with the necessary knowledge and skills), then CHWs will feel confident and competent to do their tasks effectively, leading to increased job satisfaction over time. - If there are formalized links between CHWs and clinics, CHWs will feel valued and recognized, fostering motivation and job satisfaction, which leads to retention, Integration into the formal health system and increased effectiveness in delivering healthcare services to the community. - If CHWs' have an understanding of the community they serve or understand the local context, then this familiarity builds a sense of trust among community members as they perceive CHWs as individuals who understand and respect their local customs and challenges, leading to tailored services that meet specific needs of the community effectively. - If CHWs have personal experiences related to the condition of the patient, either through their own lives or within their communities, then CHWs can empathize; and this shared experience can create a deeper connection with community members, fostering trust and acceptability of the CHWs as credible sources of support and information, As a result, community members will be more to engage with CHWs, follow their advice, and adhere to treatment plans. |

**Test for relevance (Pearson et al. 2012; 2015; Brennan et al. 2017)**

| **Conceptually Rich** | **Thicker description’ but not ‘conceptually rich’** | **Conceptually Thin** |
| --- | --- | --- |
| Unambiguous theoretical concepts are described in sufficient depth. | Description of programme theory or sufficient information to enable it to ‘surface’. | Insufficient information to enable the programme theory to surface. |
| Relationships between, amongst concepts are clearly articulated. | Consideration of the context in which the programme takes place. | Limited or no consideration of the context in which the programme took place. |
| Concepts are sufficiently developed, defined to enable understanding without the reader needing to have first-hand experience of an area of practice. | Discussion of the differences between the design and orientation of programme theory (what was intended) and implementation (what really happened). | Limited or no discussion of the differences between the design and orientation of programme theory (what was intended) and implementation (what really happened). |
| Concepts are grounded strongly in a cited body of literature. | Recognition and discussion of the strengths/weaknesses of the implemented programme. | Limited or no discussion of the strengths/ weaknesses of the implemented programme. |
| Concepts are parsimonious (i.e., provide the simplest, but not over-simplified, explanation) | Some attempt to explain anomalous results and findings with reference to context and data. | No attempts to explain anomalous results and findings with reference to context and data. |
|  | Description of the factor affecting implementation. | Limited or no description of the factors affecting implementation. |

**Test for relevance (Pearson et al. 2012; 2015; Brennan et al. 2017)**

| **Conceptually Rich** | **Thicker description’ but not ‘conceptually rich’** | **Conceptually Thin** |
| --- | --- | --- |
|  | X |  |

**Test for rigour (Ohly et al. 2017)**

|  | **Yes** | **Fairly** | **No** |
| --- | --- | --- | --- |
| The study methods are clearly reported. | X |  |  |
| The study methods are appropriate to answer RQ. | X |  |  |
| The sample characteristics enable generalizability. |  |  | X |
| Raw data supports the study findings (conclusions). | X |  |  |
| Limitations of the study are acknowledged and clearly reported. | X |  |  |

**References**

1. Wong G, Westhorp G, Manzano A, Greenhalgh J, Jagosh J, Greenhalgh T. (2016). RAMESES II

reporting standards for realist evaluations. *BMC Medicine*, 14(1), 1-18.

2. Wong G, Greenhalgh T, Westhorp G, Pawson R. (2012). Realist methods in medical education

research: what are they and what can they contribute? *Medical Education*, 46(1), 89-96.

3. Macfarlane F, Greenhalgh T, Humphrey C, Hughes J, Butler C, Pawson R. (2011). A new workforce

in the making?: A case study of strategic human resource management in a whole-system change

effort in healthcare. *Journal of Health Organization and Management*, 25(1), 55-72.

4. Lacouture A, Breton E, Guichard A, Ridde V. (2015). The concept of mechanism from a realist

approach: a scoping review to facilitate its operationalization in public health program evaluation.

*Implementation Science*, 10(1), 1-10.

5. Pearson M, Chilton R, Woods HB, Wyatt K, Ford T, Abraham C, et al. Implementing health promotion in schools: protocol for a realist systematic review of research and experience in the United Kingdom (UK). Syst Rev. 2012 Oct 20;1(1):48.

6. Pearson M, Brand SL, Quinn C, Shaw J, Maguire M, Michie S, et al. Using realist review to inform intervention development: methodological illustration and conceptual platform for collaborative care in offender mental health. Implementation Sci. 2015 Sep 28;10(1):134.

7. Brennan N, Bryce M, Pearson M, Wong G, Cooper C, Archer J. Towards an understanding of how appraisal of doctors produces its effects: a realist review. Medical Education. 2017;51(10):1002–13.

8. Ohly H, Crossland N, Dykes F, Lowe N, Hall-Moran V. A realist review to explore how low-income pregnant women use food vouchers from the UK’s Healthy Start programme. BMJ Open. 2017 Apr 21;7(4):e013731.

**A realist evaluation of the role and functions of community health workers in service of a people-centred community health system.**

**Investigator CMOs Data Extraction Sheet Data Extraction**

The purpose of this document is to extract data from the identified studies in order to formulate one or more explanatory theories that explain Context-Mechanism-Outcome Configurations (CMOC) and to gain insight into how these CMOs contribute to optimizing the role and functions of community health workers within a people-centred community health system.

In realist methodologies, knowledge accumulation centres on our comprehension of how mechanisms behave in various contexts and the reasons behind how and why this interplay of context and mechanism leads to different outcomes (1). Please fill in the details below for the study you will be assessing. In the subsequent three tables, locate and specify the Intervention, Context, Mechanism, and Outcomes (CMO) of the intervention discussed in the study you've examined. Definitions and illustrations of CMO are provided within the tables.

**Kindly fill out the information below for the study you will be reviewing.**

| **Reviewer Name** | Usangiphile Buthelezi |
| --- | --- |
| **Reference** | Abbey M, Bartholomew LK, Nonvignon J, Chinbuah MA, Pappoe M, Gyapong M, et al. Factors related to retention of community health workers in a trial on community-based management of fever in children under 5 years in the Dangme West District of Ghana. Int Health. 2014 Jun;6(2):99–105. |
| **Country** | Ghana |
| **Setting and population** | - The study was conducted in the Dangme West District of Ghana, a rural area in a resource-constrained setting of a developing country. - The population of interest in the study was community health workers (CHWs) who were volunteers participating in the cluster randomized trial on community management of fever in children under 5 years. - The study included a total of 520 CHWs who were interviewed and provided data for analysis. |
| **Aim** | - The aim of the study was to examine factors influencing the retention of volunteer community health workers (CHWs) in a cluster randomized trial on community-based management of fever in children under 5 years in the Dangme West District of Ghana. |
| **Objectives** | - The study aimed to identify the factors that influenced CHWs' decisions to remain or leave the program and understand the motivations for retention. - The study also aimed to explore the role of community involvement in the selection process and its impact on CHW retention. |
| **Methodology** | - The study applied a mixed-method approach, combining structured interviews and focus group discussions with community health workers (CHWs) in the Dangme West District of Ghana. - Data were obtained from structured interviews with 520 CHWs and focus group discussions with 5 groups of CHWs. - Attrition rates and sociodemographic characteristics of CHWs were abstracted from the CHW database built as part of the project. - The study used a probit regression model to examine factors related to CHW attrition, including approval of the CHW by the community and immediate family, number of children, age, years of schooling, and previous involvement in similar programs. - The analysis of focus group discussions and interviews was an iterative process, involving transcription, coding, and thematic analysis. - The study also utilized information from field reports and informal discussions with project staff to understand the reasons for attrition |
| **Intervention description** | - The intervention focused on community-based management of fever in children under 5 years in the Dangme West District of Ghana. - The intervention involved the engagement of 660 volunteer community health workers (CHWs) for a period of 30 months. - CHWs operated in their homes and were responsible for assessing febrile children brought to them by their caregivers. They either treated the children or referred those with danger signs to the nearest health facility. They also provided counseling to caregivers on medication administration and referral compliance. - The selection process for CHWs involved community involvement, where community members openly discussed criteria for selection and nominated and voted for individuals of their choice. - The study provided free medications, including antimalarial medication and antibiotics, to the CHWs for the treatment of sick children. |
| **Intervention outcomes** | - The attrition rate of community health workers (CHWs) in the intervention was 21.2% over the 30-month period. This attrition rate was considered moderate compared to other studies in Senegal, Nigeria, and Kenya. - Factors such as community approval and support, as well as approval from the CHWs' immediate family, were found to be significant in influencing the probability of CHWs remaining in the program. - The high level of community involvement in the selection process and the support of immediate family members contributed to the relatively moderate attrition rate. - The intervention successfully kept the majority of CHWs motivated and retained in the program. The recognition and appreciation of CHWs' work by health workers and the community were important in motivating and sustaining the CHWs. - The study suggested that attention to community involvement, as well as the inclusion of appropriate and adequate incentives, could further improve CHW retention in similar community-based health interventions. |
| **Challenges and limitations** | - The study faced challenges in conducting interviews with CHWs who had left the program, as many of them did not give prior notice and were difficult to reach. This limited the ability to gather first-hand information from those who had attrited from the program. - The study acknowledged the shortfall of not being able to follow up with CHWs who were lost to attrition, which would have provided more in-depth information on the reasons for attrition. Future studies could address this limitation. - The study relied on self-reported data from CHWs, which may be subject to recall bias or social desirability bias. - The study was conducted in a specific rural district in Ghana, which may limit the generalizability of the findings to other settings or populations. - The study did not explore the specific incentives or support mechanisms that could further improve CHW retention, which could be an area for future research. |

**Table 1 – Context**

| **Including (but not limited to) beliefs, social and cultural norms, regulations and economic factors (2,3). For example:**   - Individual capabilities (i.e. values, roles, knowledge, purpose). - Interpersonal relationships supporting the interventions (i.e. communication, collaboration, network, influences). - Institutional settings (i.e. informal rules, organisational culture, leadership, policies, resource allocation, local priorities). - Infra-structural system (i.e. political support). - Country and rurality (i.e. small or large/rural vs regional vs remote). - Socio-demographic characteristics: - Funding context or source (i.e. free, personalised, group vs. individual, government funded). |
| --- |
| - The study was conducted in the Dangme West District of Ghana, a rural district in the Greater Accra Region. - The district had limited access to healthcare facilities, with only four government-owned health centres and six community clinics. - The district had a population of approximately 109,459 people living in 376 communities. - The study acknowledged the challenges of poor road networks and limited access to healthcare facilities in the district. |

**Table 2 – Mechanisms**

| - **“An element of reasoning and/or reactions of an individual or collective agents(s) in regard of resources available in a given context to bring about changes through the implementation of an intervention” (4). Check what are the mediators to produce outcomes (not only primary, but also secondary or unintended outcomes).** |
| --- |
| **Perceptions and Motivation:**   - CHWs who perceived their work as a way to serve their communities and had a strong sense of commitment to preventing childhood deaths were motivated to stay in the program. - Recognition and appreciation from the community and professional health staff also served as a source of motivation for CHWs to continue their work.   **Perceived Support and Rapport:**   - CHWs who received approval from the community and their immediate family were more likely to remain in the program, indicating the importance of social support and rapport. - Cordial relationships with professional health staff and the community at large also contributed to CHWs' motivation to stay in the program.   **Values and Trust:**   - CHWs expressed a sense of social responsibility and a desire to fulfill their moral obligation to prevent childhood deaths, which influenced their decision to continue in the program.   **Recognition and Satisfaction:**   - CHWs derived satisfaction from the recognition and appreciation of their work by health workers and the community, which played a role in motivating and sustaining them. |

**Table 3 – Outcomes**

| - **Please make note of all (intended or not) outcomes. Please also classify the role and functions outcomes of CHWs using the following Pillars for people-centred care:**   1. Engaging and empowering people and communities  2. Strengthening governance and accountability (Incl. Supervision, training, and support)  3. Reorienting the model of care  4. Coordinating services within and across sectors  5. Enabling environment |
| --- |
| - The attrition rate of community health workers (CHWs) in the intervention was 21.2% over the 30-month period. This attrition rate was considered moderate compared to other studies in Senegal, Nigeria, and Kenya. - Factors such as community approval and support, as well as approval from the CHWs' immediate family, were found to be significant in influencing the probability of CHWs remaining in the program. - The high level of community involvement in the selection process and the support of immediate family members contributed to the relatively moderate attrition rate. - The intervention successfully kept the majority of CHWs motivated and retained in the program. The recognition and appreciation of CHWs' work by health workers and the community were important in motivating and sustaining the CHWs. - The study suggested that attention to community involvement, as well as the inclusion of appropriate and adequate incentives, could further improve CHW retention in similar community-based health interventions.   **Outcomes by PCC pillars:**  **Engaging and empowering people and communities:**   - The high level of community involvement in the selection process of CHWs and the support of their immediate family members contributed to the retention of CHWs in the program.   **Strengthening governance and accountability (Incl. Supervision, training, and support):**   - CHWs received training from the Ghana Health Service using simplified guidelines for Integrated Management of Childhood Illnesses. They also received regular supportive supervision and replenishment of stock from project supervisors.   **Reorienting the model of care:**   - The study did not explicitly mention the reorientation of the model of care as an outcome.   **Coordinating services within and across sectors:**   - The study did not explicitly mention the coordination of services within and across sectors as an outcome.   **Enabling environment:**   - Factors such as community approval, recognition by professional health staff, and support from the community created an enabling environment for CHWs, contributing to their retention in the program. |

**Table 4 – If, then statements depicting the CMOs.**

| **Please detail the CMOs for the study you reviewed using the If (Context), Then (Mechanism) statements.** |
| --- |
| **If, Then statements**   - If CHWs receive regular 1) technical mentorship, 2) supervision, and 3) support by making sure that they are well-resourced to undertake their community-based tasks (i.e. supported by health service professionals and government officials), then CHWs will feel supported and motivated to undertake their community-based tasks, leading to improved retention of CHWs in the system. - If CHWs receive approval from the community and their immediate family, Then CHWs will feel supported and there will be Rapport, leading to CHWs retention in the system. - If there is a cordial relationship with professional health staff and the community at large, Then CHWs will be motivated to stay in the programme, leading to improved retention of CHWs in the system. - If CHWs perceived their work as a way to serve their communities, Then, they will develop a strong sense of commitment to the programme (values), leading to CHW retention in the system. - If CHWs are selected through a participatory process from the communities that they serve, then CHWs will perceive their work as a way to serve their communities, which contributes to the retention of CHWs in community health programmes. - If the community is involved in the selection process of CHWs and are supported by their immediate family members, then this creates a of sense of social responsibility and accountability among CHWs, leading to a stronger commitment to the programme. |

**Test for relevance (Pearson et al. 2012; 2015; Brennan et al. 2017)**

| **Conceptually Rich** | **Thicker description’ but not ‘conceptually rich’** | **Conceptually Thin** |
| --- | --- | --- |
| Unambiguous theoretical concepts are described in sufficient depth. | Description of programme theory or sufficient information to enable it to ‘surface’. | Insufficient information to enable the programme theory to surface. |
| Relationships between, amongst concepts are clearly articulated. | Consideration of the context in which the programme takes place. | Limited or no consideration of the context in which the programme took place. |
| Concepts are sufficiently developed, defined to enable understanding without the reader needing to have first-hand experience of an area of practice. | Discussion of the differences between the design and orientation of programme theory (what was intended) and implementation (what really happened). | Limited or no discussion of the differences between the design and orientation of programme theory (what was intended) and implementation (what really happened). |
| Concepts are grounded strongly in a cited body of literature. | Recognition and discussion of the strengths/weaknesses of the implemented programme. | Limited or no discussion of the strengths/ weaknesses of the implemented programme. |
| Concepts are parsimonious (i.e., provide the simplest, but not over-simplified, explanation) | Some attempt to explain anomalous results and findings with reference to context and data. | No attempts to explain anomalous results and findings with reference to context and data. |
|  | Description of the factor affecting implementation. | Limited or no description of the factors affecting implementation. |

**Test for relevance (Pearson et al. 2012; 2015; Brennan et al. 2017)**

| **Conceptually Rich** | **Thicker description’ but not ‘conceptually rich’** | **Conceptually Thin** |
| --- | --- | --- |
|  | X |  |

**Test for rigour (Ohly et al. 2017)**

|  | **Yes** | **Fairly** | **No** |
| --- | --- | --- | --- |
| The study methods are clearly reported. | X |  |  |
| The study methods are appropriate to answer RQ. | X |  |  |
| The sample characteristics enable generalizability. |  |  | X |
| Raw data supports the study findings (conclusions). | X |  |  |
| Limitations of the study are acknowledged and clearly reported. | X |  |  |

**References**

1. Wong G, Westhorp G, Manzano A, Greenhalgh J, Jagosh J, Greenhalgh T. (2016). RAMESES II

reporting standards for realist evaluations. *BMC Medicine*, 14(1), 1-18.

2. Wong G, Greenhalgh T, Westhorp G, Pawson R. (2012). Realist methods in medical education

research: what are they and what can they contribute? *Medical Education*, 46(1), 89-96.

3. Macfarlane F, Greenhalgh T, Humphrey C, Hughes J, Butler C, Pawson R. (2011). A new workforce

in the making?: A case study of strategic human resource management in a whole-system change

effort in healthcare. *Journal of Health Organization and Management*, 25(1), 55-72.

4. Lacouture A, Breton E, Guichard A, Ridde V. (2015). The concept of mechanism from a realist

approach: a scoping review to facilitate its operationalization in public health program evaluation.

*Implementation Science*, 10(1), 1-10.

5. Pearson M, Chilton R, Woods HB, Wyatt K, Ford T, Abraham C, et al. Implementing health promotion in schools: protocol for a realist systematic review of research and experience in the United Kingdom (UK). Syst Rev. 2012 Oct 20;1(1):48.

6. Pearson M, Brand SL, Quinn C, Shaw J, Maguire M, Michie S, et al. Using realist review to inform intervention development: methodological illustration and conceptual platform for collaborative care in offender mental health. Implementation Sci. 2015 Sep 28;10(1):134.

7. Brennan N, Bryce M, Pearson M, Wong G, Cooper C, Archer J. Towards an understanding of how appraisal of doctors produces its effects: a realist review. Medical Education. 2017;51(10):1002–13.

8. Ohly H, Crossland N, Dykes F, Lowe N, Hall-Moran V. A realist review to explore how low-income pregnant women use food vouchers from the UK’s Healthy Start programme. BMJ Open. 2017 Apr 21;7(4):e013731.

**A realist evaluation of the role and functions of community health workers in service of a people-centred community health system.**

**Investigator CMOc Data Extraction Sheet Data Extraction**

The purpose of this document is to extract data from the identified studies in order to formulate one or more explanatory theories that explain Context-Mechanism-Outcome Configurations (CMOC) and to gain insight into how these CMOCs contribute to optimizing the role and functions of community health workers within a people-centred community health system.

In realist methodologies, knowledge accumulation centres on our comprehension of how mechanisms behave in various contexts and the reasons behind how and why this interplay of context and mechanism leads to different outcomes (1). Please fill in the details below for the study you will be assessing. In the subsequent three tables, locate and specify the Context, Mechanism, and Outcomes (CMO) of the intervention discussed in the study you've examined. Definitions and illustrations of CMO are provided within the tables.

**Kindly fill out the information below for the study you will be reviewing.**

| **Reviewer Name** | Usangiphile Buthelezi |
| --- | --- |
| **Reference** | Abbey M, Bartholomew LK, Pappoe M, van den Borne B. Treating fever in children under 5 years of age: caregiver perceptions of community health worker services in Dangme West district, Ghana. Int Health. 2015 Nov;7(6):455–63. |
| **Country** | Ghana |
| **Setting and population** | - The study was conducted in the Dangme West district, which is one of the ten districts in the Greater Accra region of Ghana. The district has an estimated population of 109,459 and consists of 376 communities or villages. Malaria transmission occurs throughout the year, with peaks during the rainy seasons in April and October. The majority of the inhabitants are Christians, and the predominant ethnic group is Ga-Adangme. The district is largely rural, with poor road networks and limited access to health facilities. The inhabitants are primarily subsistence farmers, fishermen, and petty traders. - The study population consisted of caregivers of children under 5 years old living in the Dangme West district. Caregivers were defined as individuals, male or female, who were identified by household members as bearing the primary responsibility for an under-5 child. The study included approximately 700 children aged 2-59 months, selected through random cluster sampling. |
| **Aim** | - The aim of the study was to assess the community utilization, perceptions, and related factors of community health worker (CHW) services in the management of childhood fevers in children under 5 years old in the Dangme West district, Ghana. |
| **Objectives** | - The study aimed to determine the factors influencing the utilization of CHW services, explore caregiver perceptions of CHW activities, and identify recommendations for improving the CHW program. |
| **Methodology** | - The study utilized a cross-sectional survey to assess the utilization of community health worker (CHW) services for childhood fever and its related factors. The survey included approximately 700 children aged 2-59 months, selected through random cluster sampling. - Logistic regression analysis was conducted to analyze factors related to the utilization of CHW services for the management of childhood fevers. - Focus group discussions were held with 84 caregivers to explore community perceptions of the CHW program. The discussions aimed to gather qualitative data on caregiver perceptions of CHW activities, including awareness of CHWs, utilization and perceptions of CHW services, perceived benefits of CHW work, and recommendations for program improvement. - The study also collected information from project records to complement the survey findings and focus group discussions. - Triangulation of both quantitative and qualitative data was used to minimize biases and provide useful insights for program planning and implementation. |
| **Intervention description** | - The intervention in the study involved the implementation of a community health worker (CHW) program for the management of childhood fevers in children under 5 years old in the Dangme West district, Ghana. - A total of 660 CHWs were selected by their communities and trained by professional health staff. The selection of CHWs was influenced by population density and community size. - The CHWs worked in their homes and provided treatment for fevers in children aged 2-59 months. They also counseled caregivers on medication administration and referral compliance when necessary. Medicines provided to sick children were free-of-cost. - A complementary communications program was implemented to enhance awareness and utilization of CHW services. The program included oral presentations, audio tapes, a mobile van, and a locally-produced video to deliver key messages about the availability of trained CHWs and the importance of treatment and referral adherence. - The intervention also included a communication intervention targeting caregivers, which significantly increased the utilization of CHW services for fever management in children under 5 years old. |
| **Intervention outcomes** | - The utilization of community health worker (CHW) services for the management of fever in children under 5 years old was found to be 59.4%. - Caregivers who were exposed to the communication intervention were about four times more likely to use the services of CHWs compared to those who were not exposed. - Farmers were 84% more likely to use CHW services for children sick with fever compared to those who were unemployed. - Caregiver perceptions of the CHW program were generally positive, with satisfaction expressed for prompt treatment, friendliness, and free medicines. - Male involvement in the CHW program was comparatively low, suggesting the need for strategies to increase male participation in community-based child health programs. - The intervention led to positive changes in the management of fevers in children, improvements in knowledge about causes and treatment of convulsions, and a decrease in the episodes of fever in children under 5 years, potentially reducing childhood deaths. - The overall outcome of the intervention showed a reduction in all-cause mortality among the children studied. - The intervention had a high retention rate of CHWs, with an attrition rate of 21.2%. - The complementary communications program implemented alongside the CHW program enhanced awareness and utilization of CHW services in the communities. |
| **Challenges and limitations** | - The study had limited variables in assessing caregiver utilization of community health worker (CHW) services, which may have affected the comprehensive understanding of care-seeking practices. - There may have been a reporting bias from respondents in face-to-face interviews, as they may have provided socially desirable responses instead of reflecting the real-life situation, potentially resulting in incomplete and socially biased information. - Recall bias was a possibility as caregivers were asked to remember past events, which may have affected the accuracy of their responses. - The study was cross-sectional, which means that causal inferences cannot be made from the findings. - The recommendations provided by participants to address the perceived challenges of the CHW program were mostly directed at the government, indicating a reliance on external solutions. - The study was conducted in a specific district in Ghana, which may limit the generalizability of the findings to other settings. |

**Table 1 – Context**

| **Including (but not limited to) beliefs, social and cultural norms, regulations and economic factors (2,3). For example:**   - Individual capabilities (i.e. values, roles, knowledge, purpose). - Interpersonal relationships supporting the interventions (i.e. communication, collaboration, network, influences). - Institutional settings (i.e. informal rules, organisational culture, leadership, policies, resource allocation, local priorities). - Infra-structural system (i.e. political support). - Country and rurality (i.e. small or large/rural vs regional vs remote). - Socio-demographic characteristics: - Funding context or source (i.e. free, personalised, group vs. individual, government funded). |
| --- |
| - The study was conducted in the Dangme West district, which is one of the ten districts in the Greater Accra region of Ghana. - The district is largely rural, with poor road networks and limited access to health facilities. - The population in the district is predominantly made up of subsistence farmers, fishermen, and petty traders, with poverty being widespread. - The study focused on caregivers of children under 5 years old living in the Dangme West district. - The communities in the district are predominantly of the Ga-Adangme ethnic group, with other ethnic groups such as Ewes and Akans also present. |

**Table 2 – Mechanisms**

| - **“An element of reasoning and/or reactions of an individual or collective agents(s) in regard of resources available in a given context to bring about changes through the implementation of an intervention” (4). Check what are the mediators to produce outcomes (not only primary, but also secondary or unintended outcomes).** |
| --- |
| **Perceptions and Awareness:**   - Caregivers had positive perceptions of the community health worker (CHW) services, citing prompt treatment, friendliness, and free medicines. - The dissemination of information and communication interventions increased awareness and utilization of CHW services. - Caregivers found the messages disseminated understandable, appropriate, and acceptable, which may have sustained their interest.   **Trust and Satisfaction:**   - Caregivers expressed satisfaction with the CHW services, indicating a level of trust in the program. - The majority of caregivers perceived the quality of the services received as 'good' or 'excellent'.   **Motivation and Recognition:**   - Male involvement in the CHW program was comparatively low, suggesting a need for strategies to increase male participation. - Recognition of the CHW program by the community can provide a strong foundation for the roll-out of other effective community-based interventions.   **Communication and Social Connection:**   - The communication program used various methods such as oral presentations, audio tapes, mobile vans, and locally produced videos to enhance awareness and utilization of CHW services. - The involvement of the community in the design and production of communication materials may have contributed to the understanding and acceptability of the messages.   **Psychosocial Factors:**   - Caregivers' positive perceptions and satisfaction with CHW services may have influenced their care-seeking behaviors and utilization of services. - The decrease in episodes of fever and perceived reduction in childhood deaths in the communities may have increased confidence in the CHW program and promoted early care-seeking. |

**Table 3 – Outcomes**

| - **Please make note of all (intended or not) outcomes. Please also classify the role and functions outcomes of CHWs using the following Pillars for people-centred care:**   1. Engaging and empowering people and communities  2. Strengthening governance and accountability (Incl. Supervision, training, and support)  3. Reorienting the model of care  4. Coordinating services within and across sectors  5. Enabling environment |
| --- |
| - The utilization of community health worker (CHW) services for the management of fever in children under 5 years old was found to be 59.4%. - Caregivers who were exposed to the communication intervention were about four times more likely to use the services of CHWs compared to those who were not exposed. - Farmers were 84% more likely to use CHW services for children sick with fever compared to those who were unemployed. - Caregiver perceptions of the CHW program were generally positive, with satisfaction expressed for prompt treatment, friendliness, and free medicines. - Male involvement in the CHW program was comparatively low, suggesting the need for strategies to increase male participation in community-based child health programs. - The intervention led to positive changes in the management of fevers in children, improvements in knowledge about causes and treatment of convulsions, and a decrease in the episodes of fever in children under 5 years, potentially reducing childhood deaths. - The overall outcome of the intervention showed a reduction in all-cause mortality among the children studied. - The intervention had a high retention rate of CHWs, with an attrition rate of 21.2%. - The complementary communications program implemented alongside the CHW program enhanced awareness and utilization of CHW services in the communities.   **Outcomes by PCC pillars:**  **Engaging and empowering people and communities:**   - Utilization of community health worker (CHW) services for the management of childhood fevers was 59.4%, indicating community engagement and empowerment in seeking healthcare services. - Dissemination of information and communication interventions increased awareness and utilization of CHW services, empowering caregivers to seek prompt treatment for their children.   **Strengthening governance and accountability:**   - Caregiver perceptions of the CHW program were generally positive, with satisfaction expressed for prompt treatment, friendliness, and free medicines, indicating effective governance and accountability. - The majority of caregivers perceived the quality of CHW services as 'good' or 'excellent', indicating the effectiveness of supervision, training, and support provided to CHWs.   **Reorienting the model of care:**   - The CHW program provided accessible and prompt treatment for childhood fevers, reorienting the model of care towards community-based services. - Caregivers expressed satisfaction with the CHW services, citing effectiveness of medication, friendliness of CHWs, and easy accessibility, indicating a shift towards patient-centered care.   **Coordinating services within and across sectors:**   - The involvement of the community in the CHW program, including the selection of CHWs and information sharing, facilitated coordination within the community and across stakeholders. - The communication intervention, including videos and talks, promoted coordination between the CHW program and the community, enhancing exposure and utilization of services.   **Enabling environment:**   - The study was conducted in a rural district with limited access to health facilities, highlighting the need for an enabling environment to ensure community access to healthcare services. |

**Table 4 – If, then statements depicting the CMOs.**

| **Please detail the CMOs for the study you reviewed using the If (Context), Then (Mechanism) statements.** |
| --- |
| **If Then Statements:**   - If caregivers perceive CHW services positively, citing prompt treatment, friendliness, and free medicines, then they are likely to continue utilizing and advocating for those services within their community, leading to increased trust in the healthcare system, improved health outcomes for community members, and potentially greater community engagement in health-related initiatives. - If caregivers’ express satisfaction with the CHW services, indicating a level of trust in the program, then the majority of caregivers are likely to perceive the quality of the services received as 'good' or 'excellent', leading to increased confidence in the effectiveness of the CHW programme, greater utilization of CHW services by caregivers, and potentially enhanced health outcomes within the community. - If the community is involved in the design and production of communication materials used by CHWs, this collaborative approach is likely to enhance the relevance, cultural appropriateness, and effectiveness of the messages, fostering a stronger sense of ownership and empowerment within the community regarding their healthcare needs. This ultimately results in increased acceptance, understanding, and uptake of healthcare services, leading to better health outcomes. |

**Test for relevance (Pearson et al. 2012; 2015; Brennan et al. 2017)**

| **Conceptually Rich** | **Thicker description’ but not ‘conceptually rich’** | **Conceptually Thin** |
| --- | --- | --- |
| Unambiguous theoretical concepts are described in sufficient depth. | Description of programme theory or sufficient information to enable it to ‘surface’. | Insufficient information to enable the programme theory to surface. |
| Relationships between, amongst concepts are clearly articulated. | Consideration of the context in which the programme takes place. | Limited or no consideration of the context in which the programme took place. |
| Concepts are sufficiently developed, defined to enable understanding without the reader needing to have first-hand experience of an area of practice. | Discussion of the differences between the design and orientation of programme theory (what was intended) and implementation (what really happened). | Limited or no discussion of the differences between the design and orientation of programme theory (what was intended) and implementation (what really happened). |
| Concepts are grounded strongly in a cited body of literature. | Recognition and discussion of the strengths/weaknesses of the implemented programme. | Limited or no discussion of the strengths/ weaknesses of the implemented programme. |
| Concepts are parsimonious (i.e., provide the simplest, but not over-simplified, explanation) | Some attempt to explain anomalous results and findings with reference to context and data. | No attempts to explain anomalous results and findings with reference to context and data. |
|  | Description of the factor affecting implementation. | Limited or no description of the factors affecting implementation. |

**Test for relevance (Pearson et al. 2012; 2015; Brennan et al. 2017)**

| **Conceptually Rich** | **Thicker description’ but not ‘conceptually rich’** | **Conceptually Thin** |
| --- | --- | --- |
|  | X |  |

**Test for rigour (Ohly et al. 2017)**

|  | **Yes** | **Fairly** | **No** |
| --- | --- | --- | --- |
| The study methods are clearly reported. | X |  |  |
| The study methods are appropriate to answer RQ. |  | X |  |
| The sample characteristics enable generalizability. |  |  | X |
| Raw data supports the study findings (conclusions). | X |  |  |
| Limitations of the study are acknowledged and clearly reported. | X |  |  |

**References**

1. Wong G, Westhorp G, Manzano A, Greenhalgh J, Jagosh J, Greenhalgh T. (2016). RAMESES II

reporting standards for realist evaluations. *BMC Medicine*, 14(1), 1-18.

2. Wong G, Greenhalgh T, Westhorp G, Pawson R. (2012). Realist methods in medical education

research: what are they and what can they contribute? *Medical Education*, 46(1), 89-96.

3. Macfarlane F, Greenhalgh T, Humphrey C, Hughes J, Butler C, Pawson R. (2011). A new workforce

in the making?: A case study of strategic human resource management in a whole-system change

effort in healthcare. *Journal of Health Organization and Management*, 25(1), 55-72.

4. Lacouture A, Breton E, Guichard A, Ridde V. (2015). The concept of mechanism from a realist

approach: a scoping review to facilitate its operationalization in public health program evaluation.

*Implementation Science*, 10(1), 1-10.

5. Pearson M, Chilton R, Woods HB, Wyatt K, Ford T, Abraham C, et al. Implementing health promotion in schools: protocol for a realist systematic review of research and experience in the United Kingdom (UK). Syst Rev. 2012 Oct 20;1(1):48.

6. Pearson M, Brand SL, Quinn C, Shaw J, Maguire M, Michie S, et al. Using realist review to inform intervention development: methodological illustration and conceptual platform for collaborative care in offender mental health. Implementation Sci. 2015 Sep 28;10(1):134.

7. Brennan N, Bryce M, Pearson M, Wong G, Cooper C, Archer J. Towards an understanding of how appraisal of doctors produces its effects: a realist review. Medical Education. 2017;51(10):1002–13.

8. Ohly H, Crossland N, Dykes F, Lowe N, Hall-Moran V. A realist review to explore how low-income pregnant women use food vouchers from the UK’s Healthy Start programme. BMJ Open. 2017 Apr 21;7(4):e013731.

**A realist evaluation of the role and functions of community health workers in service of a people-centred community health system.**

**Investigator CMOc Data Extraction Sheet Data Extraction**

The purpose of this document is to extract data from the identified studies in order to formulate one or more explanatory theories that explain Context-Mechanism-Outcome Configurations (CMOC) and to gain insight into how these CMOCs contribute to optimizing the role and functions of community health workers within a people-centred community health system.

In realist methodologies, knowledge accumulation centres on our comprehension of how mechanisms behave in various contexts and the reasons behind how and why this interplay of context and mechanism leads to different outcomes (1). Please fill in the details below for the study you will be assessing. In the subsequent three tables, locate and specify the Context, Mechanism, and Outcomes (CMO) of the intervention discussed in the study you've examined. Definitions and illustrations of CMO are provided within the tables.

**Kindly fill out the information below for the study you will be reviewing.**

| **Reviewer Name** | Usangiphile Buthelezi |
| --- | --- |
| **Reference** | Adam MB, Dillmann M, Chen M kuang, Mbugua S, Ndung’u J, Mumbi P, et al. **Improving Maternal and Newborn Health: Effectiveness of a Community Health Worker Program in Rural Kenya**. PLoS One. 2014 Aug 4;9(8):e104027. |
| **Country** | Kenya |
| **Setting** | The study was conducted in rural Kenya, specifically in three geographically distinct areas: Eburru, Kinale, and Nyakio. |
| **Population** | The study focused on communities where people were primarily subsistence farmers and not employed in the formal economy. |
| **Study design** | Quasi-experimental nonequivalent design, with interviewers conducting oral interviews with women in every third household in the selected areas |
| **Aim** | The study aimed to assess the effectiveness of a community health worker program in improving knowledge of maternal and newborn health and increasing deliveries under skilled attendance. |
| **Methodology** | The study conducted oral interviews with women in every third household in the selected areas, using a questionnaire that included demographic information, retrospective birth history, and knowledge questions about maternal and newborn care.  The study compared the knowledge scores of women who were exposed to health messages from CHWs with those who were not exposed.  The study also assessed the percentage of facility deliveries among women exposed to health messages by CHWs compared to those who were not exposed.  The effectiveness of the CHW program was evaluated by analyzing the impact of health messages on women's knowledge and the uptake of skilled attendance during delivery |
| **Intervention description** | The intervention in the study involved the implementation of a community health worker (CHW) program in rural Kenya.  The CHWs were trained in three geographically distinct areas to deliver health messages and promote behavior change related to maternal and newborn health.  The CHWs delivered health messages to women in the local community, aiming to improve their knowledge of maternal and newborn care.  The intervention focused on promoting earlier case identification, timely referral to trained healthcare providers, and increasing deliveries under skilled attendance.  The intervention was designed to address the specific needs and challenges of rural agrarian populations, taking into account their lifestyle and limited resources. |
| **Intervention outcomes** | The intervention of the community health worker (CHW) program in rural Kenya resulted in increased knowledge of maternal and newborn health among women in the local community.  Women who were exposed to health messages delivered by CHWs had higher knowledge scores compared to those who were not exposed.  The intervention also led to an increase in the percentage of facility deliveries among women exposed to health messages by CHWs.  In the areas where the intervention was implemented, more women chose facility-based deliveries with skilled birth attendants.  The effectiveness of the CHW program was observed even in areas where women had higher educational and economic status, although the effect size was smaller. |
| **Challenges and limitations** | The intervention was adapted for rural agrarian populations, and the improvements in women accessing facility-based deliveries may not be generalizable to other populations, especially pastoralist populations.  The study utilized a quasi-experimental nonequivalent design, which does not address as many potential threats to validity as randomized trials.  Self-report was utilized in the questionnaire, which may introduce sample bias and potential inaccuracies in reporting.  The intervention focused on promoting behavior change and increasing deliveries under skilled attendance, but it did not address other factors that may influence maternal and newborn health outcomes, such as access to healthcare facilities and quality of care.  The study did not include a comparison group of women who did not receive health messages from CHWs, which limits the ability to establish a causal relationship between the intervention and the outcomes observed.  The study acknowledges the need for future qualitative and quantitative research to explore the potential impact of curriculum timing and spacing on retention rates of CHWs.  The intervention was implemented in a resource-limited setting, and while the study demonstrates the opportunity to examine evidence of effectiveness despite cost constraints, resource limitations may have affected the implementation and scalability of the intervention. |

**Table 1 – Context**

| **Including (but not limited to) beliefs, social and cultural norms, regulations and economic factors. For example:**   - Individual capabilities (i.e. values, roles, knowledge, purpose). - Interpersonal relationships supporting the interventions (i.e. communication, collaboration, network, influences). - Institutional settings (i.e. informal rules, organisational culture, leadership, policies, resource allocation, local priorities). - Infra-structural system (i.e. political support). - Country and rurality (i.e. small or large/rural vs regional vs remote). - Socio-demographic characteristics: - Funding context or source (i.e. free, personalised, group vs. individual, government funded). |
| --- |
| **Individual capabilities:**  The education level of CHWs varied by area, with women in Eburru having the least education, with an average of 7.38 years of education.  **Interpersonal relationships supporting the interventions:**   - CHWs served as volunteers in their local villages, forming an important element of the health system in Kenya. - They provided a link between the community and clinical services, promoting health education and earlier case identification. - The CHWs delivered health messages and engaged in communication and collaboration with women in the local community.   **Institutional settings:**   - The CHW program was consistent with the Kenya national strategy for improving maternal and neonatal health. - The program operated within the scope of work defined by the Kenyan Ministry of Health. - The study examined the impact of the intervention in areas with different levels of educational opportunity and economic resources.   **Infrastructural system:**   - The study was conducted in rural areas of Kenya, where most births and deaths occur at home.   **Country and rurality:**   - The study was conducted in rural areas of Kenya, specifically in three geographically distinct areas: Eburru, Kinale, and Nyakio. - The interviews were conducted in the **vernacular language spoken** by the participants, as most of them did not speak English or Kiswahili, the declared national languages of Kenya.   **Socio-demographic characteristics:**   - The study included women of different ages and marital statuses, with the modal age being 26-30 years and around 80% of women being married. |

**Table 2 – Mechanisms**

| - **“An element of reasoning and/or reactions of an individual or collective agents(s) in regard of resources available in a given context to bring about changes through the implementation of an intervention”. Check what are the mediators to produce outcomes (not only primary, but also secondary or unintended outcomes).** |
| --- |
| CHWs served as volunteers and were recruited from influential local community members, which helped them establish **trust and credibility** within the community.  The CHWs utilized a **community-based participatory approach**, involving **community input and addressing community needs** (sense of being involved). This approach likely contributed to building **trust and rapport** with the community.  CHWs engaged in **one-on-one interactions and small group sessions** to deliver health messages, allowing for **personalized and meaningful connections** with community members.  The **slower pace of training**, adapted to the agrarian lifestyle, and skill practice under supervision may have been effective in building **trust and motivation** among adult learners with low literacy.  The CHWs engaged with women in the community, delivering health messages and promoting behavior change, which **motivated women to actively participate** in the intervention.  CHWs formed strong relational connections through mentoring and skill practice, which were driven by community needs. This approach leveraged the oral and relational capacity of community members, further enhancing **trust and rapport**.  By being present in the community and actively engaging with community members, the CHWs became **familiar faces and built relationships with individuals and families**, allowing them to form **connections and gain trust.** |

**Table 3 – Outcome**

| - **Please make note of all (intended or not) outcomes. Please also classify the role and functions outcomes of CHWs using the following Pillars for people-centred care:**   1. Engaging and empowering people and communities  2. Strengthening governance and accountability (Incl. Supervision, training, and support)  3. Reorienting the model of care  4. Coordinating services within and across sectors  5. Enabling environment |
| --- |
| - The intervention of the community health worker (CHW) program in rural Kenya resulted in increased knowledge of maternal and newborn health among women in the local community. - Women who were exposed to health messages delivered by CHWs had higher knowledge scores compared to those who were not exposed. - The intervention also led to an increase in the percentage of facility deliveries among women exposed to health messages by CHWs. - In the areas where the intervention was implemented, more women chose facility-based deliveries with skilled birth attendants.   **Intervention outcomes according to PCC:**  **Engaging and empowering people and communities:**   - The community health worker (CHW) program engaged and empowered community members by training them to deliver health messages and educate women on maternal and newborn care. - The CHWs served as volunteers and formed an important element of the health system, promoting behaviour change and timely referral to trained healthcare providers. - The intervention increased knowledge of maternal and newborn care among women in the local community, empowering them to make informed decisions about their health and the health of their newborns.   **Strengthening governance and accountability:**   - The CHW program operated within the scope of work defined by the Kenyan Ministry of Health, ensuring governance and accountability. - The CHWs continued to volunteer their time and actively worked with district health teams even 18 months after completing training, demonstrating their commitment and accountability to the program.   **Reorienting the model of care:**   - The CHW program reoriented the model of care by providing a link between the community and clinical services, promoting health education and earlier case identification. - The intervention aimed to increase deliveries under skilled attendance, shifting the focus from home deliveries or deliveries with unskilled birth attendants to facility-based deliveries.   **Coordinating services within and across sectors:**   - The CHWs collaborated with district health teams, ensuring coordination of services within the healthcare system. - The CHWs served as a bridge between the community and clinical services, facilitating the referral process and ensuring timely access to healthcare.   **Enabling environment:**   - The CHW program operated within the Kenya national strategy for improving maternal and neonatal health, creating an enabling environment for the intervention. - The intervention was implemented in rural areas of Kenya, where most births and deaths occur at home, highlighting the need for interventions to improve maternal and newborn health in these settings. |

**Table 4 – If, then statements depicting the CMOs.**

| **Please detail the CMOs for the study you reviewed using the If (Context), Then (Mechanism) statements.** |
| --- |
| **If, Then statements**   - If CHWs are influential community members, then this will establish trust and credibility within the community, leading to higher acceptance of health interventions and recommendations, fostering behavior change. - If CHWs utilize a community-based participatory approach (involving community’s input and addressing community needs), then the community members will develop a sense of value, feel empowered and respected when their input is considered, leading to a stronger sense of ownership and commitment to the intervention. - If CHWs engaged in one-on-one interactions and small group sessions to deliver health messages, then there will be tailored messaging, meaningful connection, trust and understanding between CHWs and the community, leading to community members adopting healthier behaviors surrounding birth, delivery, and newborn care. - If CHWs provide mentoring and skill practice, which is driven by community needs, then this will improve relational capacity of community members, further enhancing trust and rapport, leading to increased adoption of healthier behaviors related to maternal and newborn care. - If CHWs are present in the community and actively engaging with community members, then CHWs become familiar faces and build relationships with individuals and families, allowing them to form connections and gain trust, leading to enhanced acceptance of health messages and advice, effectively dissemination of healthcare information, and improved access to essential services to the community. - If the CHWs offered training to the community members on to deliver health messages and educate women on maternal and newborn care, then the community members will feel empowered, leading to increased knowledge and awareness, improved Health Literacy, enhanced self-efficacy, and positive behavior change. - If CHWs operated within the scope of work defined by the Ministry of Health, then there will be a sense of accountability among CHWs, as they are responsible for delivering services as per the established guidelines, leading to improved monitoring and evaluation of their activities. - If CHWs act as intermediaries, connecting community members with clinical services (i.e. by providing health education, raising awareness about maternal and newborn care, and identifying potential health issues at an early stage), then there will be increased awareness of maternal and newborn health, timely identification of health issues, and enhanced access to clinical services, leading to a more efficient and effective healthcare delivery system. - If the CHW programme operate within the scope of work defined by the Ministry of Health, then CHWs gain credibility and trust within the community, as their activities are recognized and supported by the Ministry of Health, leading to enhanced community engagement and participation in healthcare initiatives. - If the CHW programme operate within the scope of work defined by the Ministry of Health, then CHWs will have a sense of responsibility and accountability to deliver services aligned with the national health priorities and guidelines, leading to a more effective implementation of healthcare interventions. |

**Test for relevance (Pearson et al. 2012; 2015; Brennan et al. 2017)**

| **Conceptually Rich** | **Thicker description’ but not ‘conceptually rich’** | **Conceptually Thin** |
| --- | --- | --- |
| Unambiguous theoretical concepts are described in sufficient depth. | Description of programme theory or sufficient information to enable it to ‘surface’. | Insufficient information to enable the programme theory to surface. |
| Relationships between, amongst concepts are clearly articulated. | Consideration of the context in which the programme takes place. | Limited or no consideration of the context in which the programme took place. |
| Concepts are sufficiently developed, defined to enable understanding without the reader needing to have first-hand experience of an area of practice. | Discussion of the differences between the design and orientation of programme theory (what was intended) and implementation (what really happened). | Limited or no discussion of the differences between the design and orientation of programme theory (what was intended) and implementation (what really happened). |
| Concepts are grounded strongly in a cited body of literature. | Recognition and discussion of the strengths/weaknesses of the implemented programme. | Limited or no discussion of the strengths/ weaknesses of the implemented programme. |
| Concepts are parsimonious (i.e., provide the simplest, but not over-simplified, explanation) | Some attempt to explain anomalous results and findings with reference to context and data. | No attempts to explain anomalous results and findings with reference to context and data. |
|  | Description of the factor affecting implementation. | Limited or no description of the factors affecting implementation. |

**Test for relevance (Pearson et al. 2012; 2015; Brennan et al. 2017)**

| **Conceptually Rich** | **Thicker description’ but not ‘conceptually rich’** | **Conceptually Thin** |
| --- | --- | --- |
| X |  |  |

**Test for rigour (Ohly et al. 2017)**

|  | **Yes** | **Fairly** | **No** |
| --- | --- | --- | --- |
| The study methods are clearly reported. | X |  |  |
| The study methods are appropriate to answer RQ. | X |  |  |
| The sample characteristics enable generalizability. |  |  | X |
| Raw data supports the study findings (conclusions). | X |  |  |
| Limitations of the study are acknowledged and clearly reported. | X |  |  |

**References**

1. Wong G, Westhorp G, Manzano A, Greenhalgh J, Jagosh J, Greenhalgh T. (2016). RAMESES II

reporting standards for realist evaluations. *BMC Medicine*, 14(1), 1-18.

2. Wong G, Greenhalgh T, Westhorp G, Pawson R. (2012). Realist methods in medical education

research: what are they and what can they contribute? *Medical Education*, 46(1), 89-96.

3. Macfarlane F, Greenhalgh T, Humphrey C, Hughes J, Butler C, Pawson R. (2011). A new workforce

in the making?: A case study of strategic human resource management in a whole-system change

effort in healthcare. *Journal of Health Organization and Management*, 25(1), 55-72.

4. Lacouture A, Breton E, Guichard A, Ridde V. (2015). The concept of mechanism from a realist

approach: a scoping review to facilitate its operationalization in public health program evaluation.

*Implementation Science*, 10(1), 1-10.

5. Pearson M, Chilton R, Woods HB, Wyatt K, Ford T, Abraham C, et al. Implementing health promotion in schools: protocol for a realist systematic review of research and experience in the United Kingdom (UK). Syst Rev. 2012 Oct 20;1(1):48.

6. Pearson M, Brand SL, Quinn C, Shaw J, Maguire M, Michie S, et al. Using realist review to inform intervention development: methodological illustration and conceptual platform for collaborative care in offender mental health. Implementation Sci. 2015 Sep 28;10(1):134.

7. Brennan N, Bryce M, Pearson M, Wong G, Cooper C, Archer J. Towards an understanding of how appraisal of doctors produces its effects: a realist review. Medical Education. 2017;51(10):1002–13.

8. Ohly H, Crossland N, Dykes F, Lowe N, Hall-Moran V. A realist review to explore how low-income pregnant women use food vouchers from the UK’s Healthy Start programme. BMJ Open. 2017 Apr 21;7(4):e013731.

**A realist evaluation of the role and functions of community health workers in service of a people-centred community health system.**

**Investigator CMOc Data Extraction Sheet Data Extraction**

The purpose of this document is to extract data from the identified studies in order to formulate one or more explanatory theories that explain Context-Mechanism-Outcome Configurations (CMOC) and to gain insight into how these CMOCs contribute to optimizing the role and functions of community health workers within a people-centred community health system.

In realist methodologies, knowledge accumulation centres on our comprehension of how mechanisms behave in various contexts and the reasons behind how and why this interplay of context and mechanism leads to different outcomes (1). Please fill in the details below for the study you will be assessing. In the subsequent three tables, locate and specify the Context, Mechanism, and Outcomes (CMO) of the intervention discussed in the study you've examined. Definitions and illustrations of CMO are provided within the tables.

**Kindly fill out the information below for the study you will be reviewing.**

| **Reviewer Name** | Usangiphile Buthelezi |
| --- | --- |
| **Reference** | Adesoro O, Oresanya O, Counihan H, Hamade P, Eguavon D, Emebo C, et al. A feasibility study to assess non-clinical community health workers’ capacity to use simplified protocols and tools to treat severe acute malnutrition in Niger state Nigeria. BMC Health Services Research. 2021 Oct 15;21(1):1102. |
| **Country** | Nigeria |
| **Setting and population** | The study was conducted in Mariga and Rijau, two of the six local government areas (LGAs) in Niger state, Nigeria.  The study targeted the under-five population in these communities, which had a projected total population of 104,912.  The families in these communities were described as poor, illiterate, and lacking access to basic social amenities and healthcare. |
| **Aim** | The aim of the study was to assess the feasibility of non-clinical Community Health Workers (CORPs) using simplified protocols and tools to treat severe acute malnutrition (SAM) in Niger State, Nigeria.  The study aimed to determine whether CORPs implementing integrated community case management (iCCM) could effectively treat uncomplicated SAM using simplified tools. |
| **Objectives** | The study also aimed to document the competency of CORPs in treating SAM and the treatment outcomes of enrolled children.  Additionally, the study sought to assess the acceptability of the approach among key stakeholders, including CORPs, supervisors, caregivers, policy makers, and program implementers.  The study aimed to identify operational challenges and the need for government funding to ensure the sustainability of the intervention |
| **Methodology** | - The study used a sequential multi-method design to assess the feasibility of non-clinical Community Health Workers (CORPs) using simplified protocols and tools to treat severe acute malnutrition (SAM) in Niger State, Nigeria - Sixty CORPs who were already providing integrated community case management (iCCM) services were selected and trained to treat uncomplicated SAM using the simplified tools. - The competency of CORPs in treating SAM and the treatment outcomes of enrolled children were documented. Supervision and quantitative data capturing were done weekly, while qualitative data were collected after the intervention. - Data analysis focused on treatment outcomes, number of weeks in treatment, and associations between child CORP characteristics and treatment outcomes. Single and multiple regression models were used for analysis. - The study also included a qualitative component, which involved Focus Group Discussions (FGDs) and in-depth interviews (IDIs) with CORPs, supervisors, caregivers, policy makers, and program implementers to assess the acceptability of the intervention. - The simplified SAM treatment protocol and tools used in South Sudan in 2016 were pretested and adapted based on feedback from officials and CORPs in Niger State. The tools were made more user-friendly and compliant with the cultural context and Nigerian SAM treatment protocol. |
| **Intervention description** | - Sixty CORPs who were already providing integrated community case management (iCCM) services were selected and trained to treat uncomplicated SAM cases. They were provided with the necessary resources, including 400 cartons of Ready-to-Use Therapeutic Food (RUTF), additional doses of amoxicillin and albendazole, and metal boxes to securely store the RUTF. - The intervention involved the use of a modified color-coded Mid-Upper Arm Circumference (MUAC) strip to identify children with SAM. Children who fell in the severe malnutrition zone of the MUAC were further screened for eligibility using an appetite test. Those who passed the test were enrolled in the study and received treatment from the CORPs. Treatment included the administration of amoxicillin and albendazole, as well as the provision of RUTF based on weight indicators. CORPs also provided counseling to caregivers on administering the treatment at home. - The intervention was monitored through weekly supervision and data collection. Treatment outcomes, including recovery rates and length of treatment, were documented. Operational challenges, such as bad terrains for supervision and supply chain management, were reported by supervisors. The need for government funding was identified for the sustainability of the intervention. |
| **Intervention outcomes** | - The competency of non-clinical Community Health Workers (CORPs) in treating severe acute malnutrition (SAM) using simplified protocols and tools was assessed. CORPs scored 93.1% on the first assessment, with an increment of 0.11 points per additional supervision conducted. - The cure rate from SAM to full recovery, excluding referrals, was 73.5%, and the median length of treatment was 7 weeks. SAM cases enrolled at a MUAC of 9 cm to < 10.25 cm had a 31% lower likelihood of recovery compared to those enrolled at 10.25 cm to < 11.5 cm. - CORPs were not burdened by the integration of SAM into integrated community case management (iCCM) and felt motivated by children's recovery. Operational challenges such as bad terrains for supervision, supply chain management, and referrals were reported. Government funding was identified as key for sustainability. - Caregivers reported that their family members were relieved to hear that there were no costs associated with the treatment. The intervention was found to be acceptable by key stakeholders. |
| **Challenges and limitations** | - Operational challenges were reported, including bad terrains for supervision, supply chain management difficulties, and challenges with referrals. - The initial procurement of Ready-to-Use Therapeutic Food (RUTF) was challenging due to the absence of an existing community-level nutrition program in the area. - Some CORPs wrongly referred children enrolled in the dark red Mid-Upper Arm Circumference (MUAC) zone due to perceived danger, indicating a need for improved understanding and adherence to the protocol. - The study had a higher default rate compared to the Sphere standard, possibly due to malnutrition not being recognized as a health problem requiring treatment among rural dwellers. Some caregivers preferred seeking alternative, often spiritual, means of treatment. - The study's findings may be specific to the context of Niger State, Nigeria, and may not be generalizable to other settings. - The study was conducted over a relatively short period of time (July 2017 to May 2018), and longer-term sustainability and scalability of the intervention were not fully explored. |

**Table 1 – Context**

| **Including (but not limited to) beliefs, social and cultural norms, regulations and economic factors. For example:**   - Individual capabilities (i.e. values, roles, knowledge, purpose). - Interpersonal relationships supporting the interventions (i.e. communication, collaboration, network, influences). - Institutional settings (i.e. informal rules, organisational culture, leadership, policies, resource allocation, local priorities). - Infra-structural system (i.e. political support). - Country and rurality (i.e. small or large/rural vs regional vs remote). - Socio-demographic characteristics: - Funding context or source (i.e. free, personalised, group vs. individual, government funded). |
| --- |
| - **Country and rurality:**   The study was conducted in Niger State, Nigeria, in communities with poor access to basic social amenities and healthcare.   - **Socio-demographic characteristics:**   The study population consisted of under-five children in poor, illiterate families with limited access to healthcare.   - **Institutional settings:**   The study highlighted the importance of training and supportive supervision provided to the CORPs, indicating the presence of institutional support and policies for their capacity building. |

**Table 2 – Mechanisms**

| - **“An element of reasoning and/or reactions of an individual or collective agents(s) in regard of resources available in a given context to bring about changes through the implementation of an intervention”. Check what are the mediators to produce outcomes (not only primary, but also secondary or unintended outcomes).** |
| --- |
| - Training and deployment of non-clinical Community Health Workers (CORPs) **empowered** them to provide treatment for severe acute malnutrition (SAM) and contributed to their competency in treating SAM cases. - Training provided CORPs with the necessary **knowledge and skills** to assess, identify, and treat uncomplicated SAM cases. - The training included the use of simplified protocols and tools, which **enhanced the CORPs' ability** to deliver effective treatment. - Deployment of CORPs in their communities allowed them to directly engage with and provide care to children with SAM, increasing their **confidence and competency** in managing these cases. - The provision of free treatment for SAM cases eliminated financial barriers and increased acceptability among caregivers, leading to higher treatment uptake and improved outcomes. - **Motivation** derived from witnessing the recovery of children with SAM further enhanced the engagement and commitment of CORPs to the intervention (**motivation of CORPs through witnessing positive treatment outcomes**). |

**Table 3 – Outcomes**

| - **Please make note of all (intended or not) outcomes. Please also classify the role and functions outcomes of CHWs using the following Pillars for people-centred care:**   1. Engaging and empowering people and communities  2. Strengthening governance and accountability (Incl. Supervision, training, and support)  3. Reorienting the model of care  4. Coordinating services within and across sectors  5. Enabling environment |
| --- |
| - Overall, the Interventions that contributed to the outcomes in this study included training and supervision of CORPs, integration of SAM treatment into the iCCM program, simplified protocols and tools, provision of free treatment, and the motivation of CORPs through witnessing positive treatment outcomes. - Competency of non-clinical Community Health Workers (CORPs) in treating severe acute malnutrition (SAM) using simplified protocols and tools was assessed, with CORPs scoring 93.1% on the first assessment and showing improvement with additional supervision. - The cure rate from SAM to full recovery, excluding referrals, was 73.5%, and the median length of treatment was 7 weeks. - SAM cases enrolled at a MUAC of 9 cm to < 10.25 cm had a 31% lower likelihood of recovery compared to those enrolled at 10.25 cm to < 11.5 cm. - CORPs were not burdened by the integration of SAM into integrated community case management (iCCM) and felt motivated by children's recovery. - Operational challenges such as bad terrains for supervision, supply chain management, and referrals were reported, while government funding was identified as key for sustainability. - Caregivers reported relief upon hearing that there were no costs associated with the treatment, and the intervention was found to be acceptable by key stakeholders. - The use of simplified protocols and tools enabled CORPs to effectively treat uncomplicated SAM cases, reducing the burden on healthcare facilities and increasing access to treatment in the community. - The study had a higher default rate compared to the Sphere standard, possibly due to alternative treatment-seeking behaviours and lack of recognition of malnutrition as a health problem among rural dwellers.      - The study's findings may be specific to the context of Niger State, Nigeria, and may not be generalizable to other settings.   **Outcomes according to PCC Pillars:**  **Engaging and empowering people and communities:**   - Non-clinical Community Health Workers (CORPs) were trained and deployed in their communities, empowering them to provide treatment for severe acute malnutrition (SAM). - CORPs felt motivated by the recovery of children, indicating their engagement and commitment to the intervention. - Caregivers expressed positive impressions of the program, highlighting the benefits of child recovery, free care, and shorter distances to access treatment.   **Strengthening governance and accountability (Incl. Supervision, training, and support):**   - CORPs received training on the simplified SAM protocol and its integration with the integrated community case management (iCCM) treatment algorithm. - Weekly supervision was provided by community health extension workers (CHEWs) to ensure quality and adherence to protocols. - Supervisors corrected CORPs' mistakes in person, contributing to their professional development and accountability.   **Reorienting the model of care:**   - The study assessed the feasibility of non-clinical CORPs treating uncomplicated SAM using simplified protocols, reorienting the care model to include community-based treatment. - CORPs acquired the knowledge and skills required to assess, identify, and treat SAM among under-fives, demonstrating the potential for reorienting care delivery.   **Coordinating services within and across sectors:**   - The intervention integrated SAM treatment into the existing iCCM program, leveraging the established network of CORPs providing treatment services for malaria, diarrhea, and pneumonia.   **Enabling environment:**   - Government funding was identified as crucial for the sustainability of the intervention, highlighting the need for an enabling environment to support and scale up the program. |

**Table 4 – If, then statements depicting the CMOs.**

| **Please detail the CMOs for the study you reviewed using the If (Context), Then (Mechanism) statements.** |
| --- |
| **If, Then statements**   - If non-clinical Community Health Workers (CORPs) are trained using simplified protocols and tools to provide treatment for severe acute malnutrition, then they will gain knowledge and skills and feel empowered to provide treatment for severe acute malnutrition, leading to improved treatment outcomes for children with severe acute malnutrition. - If CHWs provide treatment for severe acute malnutrition and witness a positive outcome, then they will derive motivation from witnessing the recovery of children with SAM, leading to further engagement with the intervention. - If there is regular supervision provided by community health extension workers to CHWs, then CHWS will develop a sense of accountability, leading to enhanced performance and quality care. - If the is regular training of CHWs to provide SAM, then CHWs will be equipped with necessary knowledge and skills, fostering empowerment, confidence, and self-efficacy, leading to high-quality care. - If there is support from supervisors and the community, then CHWs will be motivated to provide SAM, leading to improved performance and sustainability in SAM management. - If there is regular supervision, training and support of CHWs to provide SAM by community health extension workers, then CHWs will develop accountability, confidence, self-efficacy, feel empowered and motivated to provide SAM, leading to enhanced performance, sustainability, and high-quality care. |

**Test for relevance (Pearson et al. 2012; 2015; Brennan et al. 2017)**

| **Conceptually Rich** | **Thicker description’ but not ‘conceptually rich’** | **Conceptually Thin** |
| --- | --- | --- |
| Unambiguous theoretical concepts are described in sufficient depth. | Description of programme theory or sufficient information to enable it to ‘surface’. | Insufficient information to enable the programme theory to surface. |
| Relationships between, amongst concepts are clearly articulated. | Consideration of the context in which the programme takes place. | Limited or no consideration of the context in which the programme took place. |
| Concepts are sufficiently developed, defined to enable understanding without the reader needing to have first-hand experience of an area of practice. | Discussion of the differences between the design and orientation of programme theory (what was intended) and implementation (what really happened). | Limited or no discussion of the differences between the design and orientation of programme theory (what was intended) and implementation (what really happened). |
| Concepts are grounded strongly in a cited body of literature. | Recognition and discussion of the strengths/weaknesses of the implemented programme. | Limited or no discussion of the strengths/ weaknesses of the implemented programme. |
| Concepts are parsimonious (i.e., provide the simplest, but not over-simplified, explanation) | Some attempt to explain anomalous results and findings with reference to context and data. | No attempts to explain anomalous results and findings with reference to context and data. |
|  | Description of the factor affecting implementation. | Limited or no description of the factors affecting implementation. |

**Test for relevance (Pearson et al. 2012; 2015; Brennan et al. 2017)**

| **Conceptually Rich** | **Thicker description’ but not ‘conceptually rich’** | **Conceptually Thin** |
| --- | --- | --- |
| X |  |  |

**Test for rigour (Ohly et al. 2017)**

|  | **Yes** | **Fairly** | **No** |
| --- | --- | --- | --- |
| The study methods are clearly reported. | X |  |  |
| The study methods are appropriate to answer RQ. | X |  |  |
| The sample characteristics enable generalizability. |  |  | X |
| Raw data supports the study findings (conclusions). | X |  |  |
| Limitations of the study are acknowledged and clearly reported. | X |  |  |

**References**

1. Wong G, Westhorp G, Manzano A, Greenhalgh J, Jagosh J, Greenhalgh T. (2016). RAMESES II

reporting standards for realist evaluations. *BMC Medicine*, 14(1), 1-18.

2. Wong G, Greenhalgh T, Westhorp G, Pawson R. (2012). Realist methods in medical education

research: what are they and what can they contribute? *Medical Education*, 46(1), 89-96.

3. Macfarlane F, Greenhalgh T, Humphrey C, Hughes J, Butler C, Pawson R. (2011). A new workforce

in the making?: A case study of strategic human resource management in a whole-system change

effort in healthcare. *Journal of Health Organization and Management*, 25(1), 55-72.

4. Lacouture A, Breton E, Guichard A, Ridde V. (2015). The concept of mechanism from a realist

approach: a scoping review to facilitate its operationalization in public health program evaluation.

*Implementation Science*, 10(1), 1-10.

5. Pearson M, Chilton R, Woods HB, Wyatt K, Ford T, Abraham C, et al. Implementing health promotion in schools: protocol for a realist systematic review of research and experience in the United Kingdom (UK). Syst Rev. 2012 Oct 20;1(1):48.

6. Pearson M, Brand SL, Quinn C, Shaw J, Maguire M, Michie S, et al. Using realist review to inform intervention development: methodological illustration and conceptual platform for collaborative care in offender mental health. Implementation Sci. 2015 Sep 28;10(1):134.

7. Brennan N, Bryce M, Pearson M, Wong G, Cooper C, Archer J. Towards an understanding of how appraisal of doctors produces its effects: a realist review. Medical Education. 2017;51(10):1002–13.

8. Ohly H, Crossland N, Dykes F, Lowe N, Hall-Moran V. A realist review to explore how low-income pregnant women use food vouchers from the UK’s Healthy Start programme. BMJ Open. 2017 Apr 21;7(4):e013731.

**A realist evaluation of the role and functions of community health workers in service of a people-centred community health system.**

**Investigator CMOc Data Extraction Sheet Data Extraction**

The purpose of this document is to extract data from the identified studies in order to formulate one or more explanatory theories that explain Context-Mechanism-Outcome Configurations (CMOC) and to gain insight into how these CMOCs contribute to optimizing the role and functions of community health workers within a people-centred community health system.

In realist methodologies, knowledge accumulation centres on our comprehension of how mechanisms behave in various contexts and the reasons behind how and why this interplay of context and mechanism leads to different outcomes (1). Please fill in the details below for the study you will be assessing. In the subsequent three tables, locate and specify the Context, Mechanism, and Outcomes (CMO) of the intervention discussed in the study you've examined. Definitions and illustrations of CMO are provided within the tables.

**Kindly fill out the information below for the study you will be reviewing.**

| **Reviewer Name** | Usangiphile Buthelezi |
| --- | --- |
| **Reference** | D’Ambruoso L, Abruquah NA, Mabetha D, van der Merwe M, Goosen G, Sigudla J, et al. Expanding Community Health Worker decision space: learning from a Participatory Action Research training intervention in a rural South African district. Human resources for health. 2023 Aug 18;21(1):66. |
| **Country** | South Africa |
| **Setting and population** | - The study was conducted in a rural South African district, specifically in three rural villages. - The population of the study setting was approximately 130,000, and the sub-district had a population of 550,000. |
| **Aim** | - The aim of the study was to assess a training intervention designed to support local decision-making capability of Community Health Workers (CHWs) in a rural South African district, particularly in the context of primary healthcare (PHC) reforms and the COVID-19 pandemic. The study aimed to deliver training to CHWs and assess its impacts and learning from their perspectives. |
| **Objectives** | - To implement a training intervention to support the local decision-making capability of Community Health Workers (CHWs) in a rural South African district. - To assess the learning and impacts of the training from the perspectives of the CHWs. - The focus was on understanding how the training intervention supported CHWs in undertaking new and expanded roles during the COVID-19 pandemic and within primary healthcare (PHC) reforms that aimed to bring services closer to the people. |
| **Methodology** | - The study employed a Participatory Action Research (PAR) approach to implement a training intervention for Community Health Workers (CHWs) in a rural South African district. - The training involved CHWs from three rural villages and was conducted through a series of workshops held in community spaces and facilitated by CHW mentors and researchers. - The workshops alternated between practice-based sessions with community stakeholders and theory-based sessions with CHWs and mentors. The PAR tools were used to problematize local health concerns, reflect on action, and appraise the outcomes. - Semi-structured interviews were conducted with CHWs before and after the intervention to gain their perspectives. - The data collected were analyzed using the decision space framework to understand the power dynamics and local actors' ability to affect devolved decision-making. |
| **Intervention description** | - The intervention in this study involved implementing a training program for Community Health Workers (CHWs) in a rural South African district. - The training was conducted using a Participatory Action Research (PAR) approach, which included workshops and sessions with CHWs, community stakeholders, and mentors. - The training aimed to equip CHWs with tools and techniques to convene community groups, raise and respond to local health concerns, understand different perspectives, and facilitate action in communities and public services. - The training focused on building CHWs' management capacity, capabilities for dialogue, role clarity, community mobilization, facilitation, analysis skills, and public speaking skills. - The intervention also aimed to improve connections and accountability mechanisms between CHWs, communities, and the health system. - The training was well-received by CHWs and was seen as an opportunity to assert themselves as a recognized cadre and improve their decision-making capability |
| **Intervention outcomes** | - The training intervention improved CHWs' decision-making capability and expanded their decision space, allowing them to assert themselves as a recognized cadre. It also enhanced their management capacity, role clarity, and community mobilization skills. - CHWs reported a "triple-benefit" from the training, including community acceptance, improved peer relationships, and enhanced communication with the health system. The principles of democratic involvement and respectful dialogue facilitated learning, peer learning, and strategic alliance building. - The training intervention addressed some challenges faced by CHWs, such as limited resources, poor role clarity, and fragile accountability mechanisms. However, there are still issues with low CHW integration in the formal primary healthcare system and a lack of national leadership and financial support. - The intervention contributed to modest improvements in CHWs' roles and resources, including their capacity to use resources effectively. Public speaking skills were particularly valued by CHWs. - The training intervention created a supportive learning environment through regular spaces for dialogue and mutual learning, enabling CHWs to develop analytical, facilitation, and public speaking skills. |
| **Challenges and limitations** | - The study acknowledges the limited sample size, with only nine Community Health Workers (CHWs) from three rural villages participating in the training intervention. - The training intervention was conducted in a specific rural district in South Africa, which may limit the generalizability of the findings to other contexts. - The study primarily relied on self-reported perspectives of CHWs, which may be subject to bias or social desirability effects. - The COVID-19 pandemic and its associated challenges, such as increased workloads and limited resources, posed additional limitations on the implementation and outcomes of the training intervention. - The lack of clear criteria for CHW roles and rotations at different working stations created confusion and hindered role clarity, which was a challenge for the study participants. - Insufficient equipment and supplies, as well as inadequate transportation, were identified as barriers that negatively affected the work of CHWs. |

**Table 1 – Context**

| **Including (but not limited to) beliefs, social and cultural norms, regulations and economic factors (2,3). For example:**   - Individual capabilities (i.e. values, roles, knowledge, purpose). - Interpersonal relationships supporting the interventions (i.e. communication, collaboration, network, influences). - Institutional settings (i.e. informal rules, organisational culture, leadership, policies, resource allocation, local priorities). - Infra-structural system (i.e. political support). - Country and rurality (i.e. small or large/rural vs regional vs remote). - Socio-demographic characteristics: - Funding context or source (i.e. free, personalised, group vs. individual, government funded). |
| --- |
| The training intervention was implemented in a rural South African district, highlighting the specific challenges faced by CHWs in rural settings such as:   - Limited financial, logistical, and health systems support, poor role clarity, precarious employment, low and no pay, unstable organizational capacity, fragile accountability mechanisms, and belittling treatment in clinics. - The COVID-19 pandemic further exacerbated the challenges faced by CHWs, leading to increased workloads and the need to balance responsibilities in both communities and clinics. - Despite the training intervention, there are still limitations in integrating CHWs into the formal primary healthcare system and addressing labour shortages and inadequate support. - Insufficient equipment and supplies, as well as inadequate transportation, negatively impact the work of CHWs. |

**Table 2 – Mechanisms**

| - **“An element of reasoning and/or reactions of an individual or collective agents(s) in regard of resources available in a given context to bring about changes through the implementation of an intervention” (4). Check what are the mediators to produce outcomes (not only primary, but also secondary or unintended outcomes).** |
| --- |
| - The training intervention equipped CHWs with tools and techniques to convene community groups, raise and respond to local health concerns, understand concerns from different perspectives, and facilitate action in communities and public services. This improved their management capacity and capabilities for dialogue, which expanded role clarity and strengthened community mobilization, facilitation, and analysis skills. Development of public speaking skills was especially valued. - Regular spaces for dialogue and mutual learning supported CHWs to gain tools and skills to rework their agency in more empowered ways. This created opportunities for CHWs to assert themselves as a recognized cadre and improved their decision-making capability. - The training intervention created a supportive learning environment through the peer modality for support and exchange, which helped CHWs develop analytical, facilitation, and public speaking skills. This built individual and collective agency and contributed to the expansion of CHW decision space. - The training intervention also improved CHWs' role clarity and capacity building to fulfill better-defined roles. This was achieved through dialogue and the development of shared mindsets with communities and the health system. |

**Table 3 – Outcomes**

| - **Please make note of all (intended or not) outcomes. Please also classify the role and functions outcomes of CHWs using the following Pillars for people-centred care:**   1. Engaging and empowering people and communities  2. Strengthening governance and accountability (Incl. Supervision, training, and support)  3. Reorienting the model of care  4. Coordinating services within and across sectors  5. Enabling environment |
| --- |
| - The training intervention improved CHWs' decision-making capability and expanded their decision space, allowing them to assert themselves as a recognized cadre. It also enhanced their management capacity, role clarity, and community mobilization skills. - CHWs reported a "triple-benefit" from the training, including community acceptance, improved peer relationships, and enhanced communication with the health system. The principles of democratic involvement and respectful dialogue facilitated learning, peer learning, and strategic alliance building. - The training intervention addressed some challenges faced by CHWs, such as limited resources, poor role clarity, and fragile accountability mechanisms. However, there are still issues with low CHW integration in the formal primary healthcare system and a lack of national leadership and financial support. - The intervention contributed to modest improvements in CHWs' roles and resources, including their capacity to use resources effectively. Public speaking skills were particularly valued by CHWs. - The training intervention created a supportive learning environment through regular spaces for dialogue and mutual learning, enabling CHWs to develop analytical, facilitation, and public speaking skills.   **Intervention outcomes according to PCC pillars:**  **Engaging and empowering people and communities:**   - The training intervention provided CHWs with tools and techniques to convene community groups, raise and respond to local health concerns, and facilitate action in communities and public services. - CHWs reported improved community acceptance and built quality relationships with communities, enabling them to engage and empower people.   **Strengthening governance and accountability (Incl. Supervision, training, and support):**   - The training intervention improved CHWs' management capacity, capabilities for dialogue, and role clarity, enhancing their ability to fulfill better-defined roles. - CHWs developed analytical, facilitation, and public speaking skills through the training, which enhanced their ability to engage with communities and the health system. - The intervention created a stable platform for building trust and action alliances in response to local needs, improving accountability mechanisms.   **Reorienting the model of care:**   - The training intervention expanded CHWs' decision space, allowing them to assert themselves as a recognized cadre and have a greater impact on decision-making. - CHWs gained tools and skills to rework their agency in more empowered ways, enabling them to play a more active role in the model of care.   **Coordinating services within and across sectors:**   - The training intervention improved CHWs' community mobilization skills, facilitating their ability to coordinate services within communities and across sectors.   **Enabling environment:**   - The training intervention provided CHWs with a supportive learning environment through regular spaces for dialogue and mutual learning, enabling them to develop skills and knowledge. - CHWs reported the principles of democratic involvement and respectful dialogue unlocked learning for them, enabling peer learning and strategic alliance building. |

**Table 4 – If, then statements depicting the CMOs.**

| **Please detail the CMOs for the study you reviewed using the If (Context), Then (Mechanism) statements.** |
| --- |
| **If, Then statements**   - If CHWs are trained and equipped with tools and techniques for community engagement (i.e convene community groups, raise, and respond to local health concerns, understand concerns from different perspectives, and facilitate action in communities and public services), Then CHWs will feel empowered and capacitated to mobilize communities effectively (i.e.  build quality relationships with communities, understand local health concerns from various perspectives, and raise awareness), leading to improved community engagement and empowerment. - If there is a supportive learning environment for CHWs, that supports regular spaces for dialogue and mutual learning, then CHWs will to gain tools and skills to rework their agency in more empowered ways, leading to improve communication and better relationships with the communities they serve. |

**Test for relevance (Pearson et al. 2012; 2015; Brennan et al. 2017)**

| **Conceptually Rich** | **Thicker description’ but not ‘conceptually rich’** | **Conceptually Thin** |
| --- | --- | --- |
| Unambiguous theoretical concepts are described in sufficient depth. | Description of programme theory or sufficient information to enable it to ‘surface’. | Insufficient information to enable the programme theory to surface. |
| Relationships between, amongst concepts are clearly articulated. | Consideration of the context in which the programme takes place. | Limited or no consideration of the context in which the programme took place. |
| Concepts are sufficiently developed, defined to enable understanding without the reader needing to have first-hand experience of an area of practice. | Discussion of the differences between the design and orientation of programme theory (what was intended) and implementation (what really happened). | Limited or no discussion of the differences between the design and orientation of programme theory (what was intended) and implementation (what really happened). |
| Concepts are grounded strongly in a cited body of literature. | Recognition and discussion of the strengths/weaknesses of the implemented programme. | Limited or no discussion of the strengths/ weaknesses of the implemented programme. |
| Concepts are parsimonious (i.e., provide the simplest, but not over-simplified, explanation) | Some attempt to explain anomalous results and findings with reference to context and data. | No attempts to explain anomalous results and findings with reference to context and data. |
|  | Description of the factor affecting implementation. | Limited or no description of the factors affecting implementation. |

**Test for relevance (Pearson et al. 2012; 2015; Brennan et al. 2017)**

| **Conceptually Rich** | **Thicker description’ but not ‘conceptually rich’** | **Conceptually Thin** |
| --- | --- | --- |
| X |  |  |

**Test for rigour (Ohly et al. 2017)**

|  | **Yes** | **Fairly** | **No** |
| --- | --- | --- | --- |
| The study methods are clearly reported. | X |  |  |
| The study methods are appropriate to answer RQ. | X |  |  |
| The sample characteristics enable generalizability. |  |  | X |
| Raw data supports the study findings (conclusions). | X |  |  |
| Limitations of the study are acknowledged and clearly reported. | X |  |  |

**References**

1. Wong G, Westhorp G, Manzano A, Greenhalgh J, Jagosh J, Greenhalgh T. (2016). RAMESES II

reporting standards for realist evaluations. *BMC Medicine*, 14(1), 1-18.

2. Wong G, Greenhalgh T, Westhorp G, Pawson R. (2012). Realist methods in medical education

research: what are they and what can they contribute? *Medical Education*, 46(1), 89-96.

3. Macfarlane F, Greenhalgh T, Humphrey C, Hughes J, Butler C, Pawson R. (2011). A new workforce

in the making?: A case study of strategic human resource management in a whole-system change

effort in healthcare. *Journal of Health Organization and Management*, 25(1), 55-72.

4. Lacouture A, Breton E, Guichard A, Ridde V. (2015). The concept of mechanism from a realist

approach: a scoping review to facilitate its operationalization in public health program evaluation.

*Implementation Science*, 10(1), 1-10.

5. Pearson M, Chilton R, Woods HB, Wyatt K, Ford T, Abraham C, et al. Implementing health promotion in schools: protocol for a realist systematic review of research and experience in the United Kingdom (UK). Syst Rev. 2012 Oct 20;1(1):48.

6. Pearson M, Brand SL, Quinn C, Shaw J, Maguire M, Michie S, et al. Using realist review to inform intervention development: methodological illustration and conceptual platform for collaborative care in offender mental health. Implementation Sci. 2015 Sep 28;10(1):134.

7. Brennan N, Bryce M, Pearson M, Wong G, Cooper C, Archer J. Towards an understanding of how appraisal of doctors produces its effects: a realist review. Medical Education. 2017;51(10):1002–13.

8. Ohly H, Crossland N, Dykes F, Lowe N, Hall-Moran V. A realist review to explore how low-income pregnant women use food vouchers from the UK’s Healthy Start programme. BMJ Open. 2017 Apr 21;7(4):e013731.

**A realist evaluation of the role and functions of community health workers in service of a people-centred community health system.**

**Investigator CMOc Data Extraction Sheet Data Extraction**

The purpose of this document is to extract data from the identified studies in order to formulate one or more explanatory theories that explain Context-Mechanism-Outcome Configurations (CMOC) and to gain insight into how these CMOCs contribute to optimizing the role and functions of community health workers within a people-centred community health system.

In realist methodologies, knowledge accumulation centres on our comprehension of how mechanisms behave in various contexts and the reasons behind how and why this interplay of context and mechanism leads to different outcomes (1). Please fill in the details below for the study you will be assessing. In the subsequent three tables, locate and specify the Context, Mechanism, and Outcomes (CMO) of the intervention discussed in the study you've examined. Definitions and illustrations of CMO are provided within the tables.

**Kindly fill out the information below for the study you will be reviewing.**

| **Reviewer Name** | Usangiphile Buthelezi |
| --- | --- |
| **Reference** | Enguita-Fernàndez C, Alonso Y, Lusengi W, Mayembe A, Manun’Ebo MF, Ranaivontiavina S, et al. Trust, community health workers and delivery of intermittent preventive treatment of malaria in pregnancy: a comparative qualitative analysis of four sub-Saharan countries. Global Public Health. 2021 Dec 2;16(12):1889–903. |
| **Country** | Democratic Republic of Congo (DRC), Madagascar, Mozambique, and Nigeria |
| **Setting and population** | - The study was conducted in four sub-Saharan African countries: the Democratic Republic of Congo (DRC), Madagascar, Mozambique, and Nigeria. - The target population for the study included pregnant women and women of reproductive age (15-45 years of age). - The study also involved formal health workers, community health workers (CHWs), traditional birth attendants, traditional healers, community leaders, household heads, and other pregnant women's relatives. - CHWs were selected from the communities they serve, and local authorities were involved in the recruitment process. |
| **Aim** | - The aim of the study was to evaluate a community-based approach to the delivery of intermittent preventive treatment of malaria in pregnancy with sulfadoxine-pyrimethamine (IPTp-SP) through community health workers (CHWs) in four sub-Saharan African countries |
| **Objectives** | - The study aimed to understand the factors that influence the anticipated acceptability of this intervention. - The study also aimed to identify the mechanisms that underpin communities' trust in the delivery of IPTp via CHWs, including perceived competence and community and healthcare system integration |
| **Methodology** | - The study followed a qualitative approach based on Grounded Theory, where theory is grounded in the data gathered rather than guided by a pre-existing theoretical framework. - The identification of respondents was based on reasoned choice, with country coordinators contacting local community leaders and regional health authorities to identify key informants for focus group discussions (FGDs) and in-depth interviews (IDIs). - Recruitment combined purposive and convenience sampling approaches. - A total of 216 in-depth interviews and 62 focus group discussions were conducted with pregnant women, women of reproductive age, community leaders, pregnant women's relatives, CHWs, formal and informal health providers. - Semi-structured question guides were used to capture participants' knowledge of maternal health, malaria prevention in pregnancy, IPTp, and perceptions of the community-based approach to IPTp. The question guides were translated into local languages and adapted to meet local context specificities. - Data collection and analysis progressed simultaneously, allowing for modifications in data collection tools based on emerging themes. Content and thematic analysis were used to analyze the data, with qualitative software used for coding. - The analyses from different sites were compared to identify commonalities and divergences. |
| **Intervention description** | - The intervention in focus is the delivery of intermittent preventive treatment of malaria in pregnancy with sulfadoxine-pyrimethamine (IPTp-SP) through community health workers (CHWs) in sub-Saharan African countries. - The intervention aims to improve the acceptability and adherence to IPTp-SP among pregnant women. - CHWs play a crucial role in delivering the intervention, as they are responsible for organizing meetings and explaining the intervention to participants, which helps in expanding awareness within the community. - The intervention requires CHWs to have specialized training in maternal health to enhance their perceived competence and gain the trust of the community. - Integration of the intervention with formal healthcare systems and promotion of socially embedded practices are essential for its success. - The study design followed a qualitative approach based on Grounded Theory, with data collected through in-depth interviews and focus group discussions. - Thematic analysis was performed to identify factors influencing the acceptability and trust-building in the intervention. |
| **Intervention outcomes** | - Trust-building was identified as a critical factor for the success of the community-based intervention. - Two mechanisms that underpin communities' trust in the delivery of IPTp via CHWs were identified: 'perceived competence' and 'community and healthcare system integration'. - Perceived competence of CHWs, including their credentials and specialized training in maternal health, influenced communities' trust in them. - Integration of the intervention with formal healthcare systems and promotion of socially embedded practices were found to be important for trust-building and program sustainability. |
| **Challenges and limitations** | - Comparing different country studies was challenging due to their different socio-economic and cultural contexts, limiting the possibility of providing more detailed localized accounts. - The study design followed a qualitative approach based on Grounded Theory, which is data-driven and does not rely on a pre-existing theoretical framework. - The analysis focused on anticipated acceptability of the intervention, which is an estimation before the intervention takes place. - The study primarily explored the perspectives of communities and healthcare providers and may not capture all factors influencing acceptability from the perspective of pregnant women. - The study did not extensively explore local conceptualizations of maternity and gender roles, which could influence judgments of CHWs' capacities in the context of the intervention. |

**Table 1 – Context**

| **Including (but not limited to) beliefs, social and cultural norms, regulations and economic factors (2,3). For example:**   - Individual capabilities (i.e. values, roles, knowledge, purpose). - Interpersonal relationships supporting the interventions (i.e. communication, collaboration, network, influences). - Institutional settings (i.e. informal rules, organisational culture, leadership, policies, resource allocation, local priorities). - Infra-structural system (i.e. political support). - Country and rurality (i.e. small or large/rural vs regional vs remote). - Socio-demographic characteristics: - Funding context or source (i.e. free, personalised, group vs. individual, government funded). |
| --- |
| - Comparing different country studies was challenging due to their different socio-economic and cultural contexts, limiting the possibility of providing more detailed localized accounts.   **Context for Democratic Republic of Congo (DRC):**   - The DRC has a high burden of malaria, accounting for 57% of total malaria cases in Central Africa, with an incidence rate of 295 per 1,000 individuals at risk. - In 2015, there were 19 million malaria cases and 42,000 deaths attributable to the infection in the country. - The DRC has low coverage of intermittent preventive treatment of malaria in pregnancy (IPTp), with only 11% of pregnant women taking at least 3 doses of IPTp in 2013. - The Ministry of Health (MoH) in the DRC has reoriented the healthcare system to increase the role of communities in healthcare delivery, including the creation of 'cellules d'animation communautaire' (CAC) and the selection of community health workers (CHWs). - CHWs in the DRC, known as 'relais communitaire' or RECO, are volunteers who promote and deliver health interventions in communities, including the distribution of insecticide-treated bed nets, management of mild malaria cases, and health promotion activities.   **Context for Madagascar:**   - Malaria is endemic in 90% of Madagascar, with the entire population considered at risk of the disease. - In 2015, there were 2.4 million cases of malaria and 6,000 deaths attributed to the infection in Madagascar. - The National Community Health Policy in Madagascar, developed in 2009, incorporates community health workers (CHWs) in the delivery of health services, including malaria control interventions. - CHWs in Madagascar, known as 'relais communautaire', are nominated by their communities and work on a voluntary basis, reporting their activities to basic health centers. - CHWs in Madagascar are primarily involved in health promotion and sensitization, and their involvement in maternal health is usually limited to these activities.   **Context for Mozambique:**   - Mozambique is among the six countries with the highest burden of malaria globally, with 8.3 million cases and 15,000 deaths attributed to malaria in 2015. - The Ministry of Health in Mozambique has implemented a community-based approach to healthcare delivery, utilizing community health workers (CHWs) known as 'Agentes Polivalentes Elementares' (APE). - CHWs in Mozambique are selected by communities and receive formal training for four months, with responsibilities including malaria diagnosis, management of non-complicated cases, health education, and promotion. - The APE program in Mozambique has a long and successful implementation history, with CHWs being supervised by district health representatives and having a sense of belonging to the health system. - Trust in CHWs in Mozambique is generally high, with both laypersons and health providers accepting their involvement in maternal health interventions and emphasizing the need for additional specialized training.   **Context for Nigeria:**   - Nigeria accounts for 55% of total malaria cases in West Africa, with 110,000 deaths due to the infection in 2015. - The incidence of malaria has decreased by 50% since 2010, but there were still 61 million cases of malaria in 2015. Only 19% of pregnant women in Nigeria take 3 doses of IPTp-SP, and ANC coverage for attendance of at least one visit is 63.2% with significant regional variation. - Nigeria has a community health provider cadre that includes different subgroups, ranging from community health officers (CHOs) to community health extension workers (CHEWs) and junior CHEWs. - There is also a cadre subgroup referred to as community-directed distributors (CDDs), which are community-selected volunteers trained to provide health education and distribute health commodities, including all doses of IPTp. |

**Table 2 – Mechanisms**

| - **“An element of reasoning and/or reactions of an individual or collective agents(s) in regard of resources available in a given context to bring about changes through the implementation of an intervention” (4). Check what are the mediators to produce outcomes (not only primary, but also secondary or unintended outcomes).** |
| --- |
| - Lack of specialized training in maternal health affects trust in CHWs. - Perceptions of community health workers' (CHWs) competence and specialized training in maternal health influence trust and acceptability of the intervention - Integration of the intervention with formal healthcare systems and promotion of socially embedded practices contribute to trust-building and acceptability. - Making CHWs' training credentials public enhances community confidence in the intervention. - Active and sustained communication channels between CHWs and the community, as well as involvement of formal healthcare systems, nurture trust and acceptance. - Concerns about CHWs' capacity to deliver the intervention, especially due to perceived lack of specific training in maternal health, can impact acceptability. |

**Table 3 – Outcomes**

| - **Please make note of all (intended or not) outcomes. Please also classify the role and functions outcomes of CHWs using the following Pillars for people-centred care:**   1. Engaging and empowering people and communities  2. Strengthening governance and accountability (Incl. Supervision, training, and support)  3. Reorienting the model of care  4. Coordinating services within and across sectors  5. Enabling environment |
| --- |
| - Trust-building was identified as a critical factor for the success of the community-based intervention. - Two mechanisms that underpin communities' trust in the delivery of IPTp via CHWs were identified: 'perceived competence' and 'community and healthcare system integration'. - Perceived competence of CHWs, including their credentials and specialized training in maternal health, influenced communities' trust in them. - Integration of the intervention with formal healthcare systems and promotion of socially embedded practices were found to be important for trust-building and program sustainability.   **Outcomes by PCC pillars:**   - **Engaging and empowering people and communities:** The community-based approach to delivering intermittent preventive treatment of malaria in pregnancy (IPTp) through community health workers (CHWs) aimed to engage and empower communities by involving them in the selection process of CHWs and promoting socially embedded practices. - **Strengthening governance and accountability:** The study emphasized the importance of CHWs' credentials being made public and the need for specialized training in maternal health to enhance their competence and build trust. This highlights the role of governance and accountability in ensuring the quality of care provided by CHWs. - **Reorienting the model of care:** The intervention focused on integrating CHWs' work with formal healthcare systems, emphasizing the involvement of the healthcare system in CHWs' activities. This reorientation aimed to improve the coordination and effectiveness of care delivery. - **Coordinating services within and across sectors:** The community-based approach to delivering IPTp through CHWs required coordination between CHWs, formal healthcare providers, and community leaders. This coordination aimed to ensure seamless service delivery and maximize the impact of the intervention. - **Enabling environment:** The study highlighted the importance of trust-building between CHWs and communities for the success of the intervention **[2]**. Creating an enabling environment involved promoting trust, community participation, and integration of CHWs' work with the healthcare system. |

**Table 4 – If, then statements depicting the CMOs.**

| **Please detail the CMOs for the study you reviewed using the If (Context), Then (Mechanism) statements.** |
| --- |
| **If, Then statements**   - If CHWs lack specialized training in maternal health to manage patients, then there will be negative perceptions and lack of trust among pregnant women and their relatives regarding the CHWs' capacity to deliver effective care, leading to poor acceptability of their services. - If CHWs are integrated into the formal health system, then this will assure communities that the care provided by CHWs meets quality and standard requirements set by the health system, fostering trust, which leads to acceptability. - If CHWs training credentials are available to the community and the public, Then the perceived competence of CHWs (i.e. their credentials and training) will foster trust from the community, leading to acceptability of the services they offer. |

**Test for relevance (Pearson et al. 2012; 2015; Brennan et al. 2017)**

| **Conceptually Rich** | **Thicker description’ but not ‘conceptually rich’** | **Conceptually Thin** |
| --- | --- | --- |
| Unambiguous theoretical concepts are described in sufficient depth. | Description of programme theory or sufficient information to enable it to ‘surface’. | Insufficient information to enable the programme theory to surface. |
| Relationships between, amongst concepts are clearly articulated. | Consideration of the context in which the programme takes place. | Limited or no consideration of the context in which the programme took place. |
| Concepts are sufficiently developed, defined to enable understanding without the reader needing to have first-hand experience of an area of practice. | Discussion of the differences between the design and orientation of programme theory (what was intended) and implementation (what really happened). | Limited or no discussion of the differences between the design and orientation of programme theory (what was intended) and implementation (what really happened). |
| Concepts are grounded strongly in a cited body of literature. | Recognition and discussion of the strengths/weaknesses of the implemented programme. | Limited or no discussion of the strengths/ weaknesses of the implemented programme. |
| Concepts are parsimonious (i.e., provide the simplest, but not over-simplified, explanation) | Some attempt to explain anomalous results and findings with reference to context and data. | No attempts to explain anomalous results and findings with reference to context and data. |
|  | Description of the factor affecting implementation. | Limited or no description of the factors affecting implementation. |

**Test for relevance (Pearson et al. 2012; 2015; Brennan et al. 2017)**

| **Conceptually Rich** | **Thicker description’ but not ‘conceptually rich’** | **Conceptually Thin** |
| --- | --- | --- |
| X |  |  |

**Test for rigour (Ohly et al. 2017)**

|  | **Yes** | **Fairly** | **No** |
| --- | --- | --- | --- |
| The study methods are clearly reported. | X |  |  |
| The study methods are appropriate to answer RQ. | X |  |  |
| The sample characteristics enable generalizability. |  |  | X |
| Raw data supports the study findings (conclusions). | X |  |  |
| Limitations of the study are acknowledged and clearly reported. | X |  |  |

**References**

1. Wong G, Westhorp G, Manzano A, Greenhalgh J, Jagosh J, Greenhalgh T. (2016). RAMESES II

reporting standards for realist evaluations. *BMC Medicine*, 14(1), 1-18.

2. Wong G, Greenhalgh T, Westhorp G, Pawson R. (2012). Realist methods in medical education

research: what are they and what can they contribute? *Medical Education*, 46(1), 89-96.

3. Macfarlane F, Greenhalgh T, Humphrey C, Hughes J, Butler C, Pawson R. (2011). A new workforce

in the making?: A case study of strategic human resource management in a whole-system change

effort in healthcare. *Journal of Health Organization and Management*, 25(1), 55-72.

4. Lacouture A, Breton E, Guichard A, Ridde V. (2015). The concept of mechanism from a realist

approach: a scoping review to facilitate its operationalization in public health program evaluation.

*Implementation Science*, 10(1), 1-10.

5. Pearson M, Chilton R, Woods HB, Wyatt K, Ford T, Abraham C, et al. Implementing health promotion in schools: protocol for a realist systematic review of research and experience in the United Kingdom (UK). Syst Rev. 2012 Oct 20;1(1):48.

6. Pearson M, Brand SL, Quinn C, Shaw J, Maguire M, Michie S, et al. Using realist review to inform intervention development: methodological illustration and conceptual platform for collaborative care in offender mental health. Implementation Sci. 2015 Sep 28;10(1):134.

7. Brennan N, Bryce M, Pearson M, Wong G, Cooper C, Archer J. Towards an understanding of how appraisal of doctors produces its effects: a realist review. Medical Education. 2017;51(10):1002–13.

8. Ohly H, Crossland N, Dykes F, Lowe N, Hall-Moran V. A realist review to explore how low-income pregnant women use food vouchers from the UK’s Healthy Start programme. BMJ Open. 2017 Apr 21;7(4):e013731.

**A realist evaluation of the role and functions of community health workers in service of a people-centred community health system.**

**Investigator CMOc Data Extraction Sheet Data Extraction**

The purpose of this document is to extract data from the identified studies in order to formulate one or more explanatory theories that explain Context-Mechanism-Outcome Configurations (CMOC) and to gain insight into how these CMOCs contribute to optimizing the role and functions of community health workers within a people-centred community health system.

In realist methodologies, knowledge accumulation centres on our comprehension of how mechanisms behave in various contexts and the reasons behind how and why this interplay of context and mechanism leads to different outcomes (1). Please fill in the details below for the study you will be assessing. In the subsequent three tables, locate and specify the Context, Mechanism, and Outcomes (CMO) of the intervention discussed in the study you've examined. Definitions and illustrations of CMO are provided within the tables.

**Kindly fill out the information below for the study you will be reviewing.**

| **Reviewer Name** | Usangiphile Buthelezi |
| --- | --- |
| **Reference** | Ferrand RA, Simms V, Dauya E, Bandason T, Mchugh G, Mujuru H, et al. The effect of community-based support for caregivers on the risk of virological failure in children and adolescents with HIV in Harare, Zimbabwe (ZENITH): an open-label, randomised controlled trial. Lancet Child Adolesc Health. 2017 Nov;1(3):175–83. |
| **Country** | Zimbambwe |
| **Setting and population** | - The study was conducted in Harare, Zimbabwe, specifically in seven communities in southwestern Harare. Each community was served by a primary health-care clinic providing acute and antenatal care services. - The study population consisted of children and adolescents aged 6-15 years with newly-diagnosed HIV infection, residing in and planning to receive HIV care in one of the study communities. - The trial included both boys and girls. |
| **Aim** | - The aim of the study was to assess the effect of community-based support provided by trained community health workers to caregivers of children and adolescents living with HIV in Harare, Zimbabwe. |
| **Objectives** | - The primary objective of the study was to assess the effect of community-based support for caregivers of HIV-infected children and adolescents on treatment outcomes, specifically the proportion of participants who died or had an HIV viral load of 400 copies per mL or higher at 12 months after antiretroviral therapy (ART) initiation, and the proportion who missed two or more scheduled clinic visits by 18 months post-enrollment. - The study also aimed to compare the outcomes between the intervention group, which received structured support visits by trained community health workers, and the control group, which received standard decentralized primary health-care clinic-based HIV care. - Additionally, the study aimed to evaluate the scalability and potential cost-effectiveness of the community-based intervention model, as well as understand contextual factors that could influence its implementation. |
| **Methodology** | - The study was an open-label, randomized controlled trial conducted in Harare, Zimbabwe. - Children aged 6-15 years with newly-diagnosed HIV were recruited from primary health-care clinics. - Participants were randomly assigned to either the intervention group or the control group. The intervention group received decentralised primary health-care clinic-based HIV care along with structured support visits by trained community health workers, while the control group received only decentralized care according to national guidelines. - The primary outcomes assessed were the proportion of participants who died or had an HIV viral load of 400 copies per mL or higher at 12 months after ART initiation, and the proportion who missed two or more scheduled clinic visits by 18 months post-enrollment. - Analyses were done using complete-case, modified-intention-to-treat approach. - Randomization was done using random-number tables generated by a computer software program, and allocation concealment was ensured. |
| **Intervention description** | - The intervention involved providing structured support visits by trained community health workers to caregivers of HIV-infected children and adolescents in addition to decentralized primary health-care clinic-based HIV care. - The community health workers emphasized the importance of staying in care and addressed participants' concerns about their children starting antiretroviral therapy (ART). - The intervention was provided to all participants, irrespective of their eligibility for ART. - The community health workers facilitated a coordinated process for continuity of care, especially for children with unstable caregiving arrangements due to parental illness or orphanhood. |
| **Intervention outcomes** | - The community-based support provided by community health workers to caregivers significantly reduced the risk of virological failure in HIV-infected children and adolescents. This was the first randomized trial of an intervention to improve treatment outcomes in this population, and the outcome was assessed using HIV viral load. - The primary outcomes measured were the proportion of participants who died or had an HIV viral load of 400 copies per mL or higher at 12 months after antiretroviral therapy (ART) initiation, and the proportion who missed two or more scheduled clinic visits by 18 months post-enrollment. - The intervention group had a higher proportion of participants taking ART after 3 months of enrollment and had a significantly lower risk of not starting ART, being lost to follow-up, experiencing death, or virological failure compared to the control group. - The intervention group showed a lower proportion of patients with viral non-suppression or death at 12 months post-ART initiation compared to the control group. - The intervention also resulted in higher retention in care at 18 months compared to the control group. - The intervention also increased the likelihood of intervention group participants informing clinic staff of their intention to transfer to another clinic, ensuring continuity of care. |
| **Challenges and limitations** | - The trial was conducted before the implementation of the "treat all" strategy, so the effect of the intervention on viral load in children who would not have been eligible for ART at the time of the study cannot be extrapolated. - The study was conducted in a routine healthcare setting, which may limit the generalizability of the findings to other settings. - The intervention relied on community health workers, who may have varying levels of training and experience, which could impact the effectiveness of the intervention. - The study focused on children and adolescents in Zimbabwe, so the findings may not be applicable to other populations or countries. - The study did not assess the long-term sustainability and cost-effectiveness of the intervention, which could be important considerations for scaling up the intervention in other settings. - The study did not explore contextual factors that could influence the implementation of the intervention, highlighting the need for further research in this area. |

**Table 1 – Context**

| **Including (but not limited to) beliefs, social and cultural norms, regulations and economic factors (2,3). For example:**   - Individual capabilities (i.e. values, roles, knowledge, purpose). - Interpersonal relationships supporting the interventions (i.e. communication, collaboration, network, influences). - Institutional settings (i.e. informal rules, organisational culture, leadership, policies, resource allocation, local priorities). - Infra-structural system (i.e. political support). - Country and rurality (i.e. small or large/rural vs regional vs remote). - Socio-demographic characteristics: - Funding context or source (i.e. free, personalised, group vs. individual, government funded). |
| --- |
| **Interpersonal relationships supporting the interventions:**   - Trained community health workers provided structured support visits to caregivers, emphasizing communication and collaboration between the health workers and caregivers.   **Institutional settings:**   - The intervention was implemented in primary health-care clinics in Harare, Zimbabwe, following national guidelines.   **Healthcare System Context:**  The study was conducted in Harare, Zimbabwe, which is a large urban area and where primary health-care clinics provided acute and antenatal care services. Provider-initiated HIV testing and counseling were standard of care in Zimbabwe since 2007.  **Population Context:**   - The study focused on children and adolescents aged 6-15 years with newly diagnosed HIV infection. The inclusion criteria were residence in and planning to receive HIV care in one of the study communities. - The study was conducted in seven communities in southwestern Harare, Zimbabwe. These communities were served by primary health-care clinics. - The intervention targeted caregivers of HIV-infected children and adolescents, as they are key mediators for children engaging with care. - The intervention was implemented in a routine healthcare setting, using existing healthcare workers. |

**Table 2 – Mechanisms**

| - **“An element of reasoning and/or reactions of an individual or collective agents(s) in regard of resources available in a given context to bring about changes through the implementation of an intervention” (4). Check what are the mediators to produce outcomes (not only primary, but also secondary or unintended outcomes).** |
| --- |
| - The intervention's success can be attributed to the targeted support provided to caregivers, the emphasis on continuity of care, and the use of existing community health workers in a routine healthcare setting.   **Targeted support provided to caregivers:**   - The intervention focused on caregivers, who are key mediators to children engaging with care, and provided structured support visits by trained community health workers. This support helped caregivers understand the importance of staying in care and address concerns about their children starting ART.   **Emphasis on continuity of care:**   - The intervention ensured a coordinated process for continuity of care, particularly important for children with unstable caregiving arrangements. Community health workers facilitated the transfer of participants to another clinic, ensuring uninterrupted access to HIV care. - This coordinated approach helped in maintaining engagement with care and reducing the risk of participants being lost to follow-up or experiencing interruptions in treatment.   **Use of existing community health workers in a routine healthcare setting:**   - The intervention utilized community health workers who already exist in many African health systems, making it a feasible and scalable approach for implementation in resource-limited settings. - By leveraging existing resources, the intervention maximized the reach and impact of the support provided to caregivers, leading to improved treatment outcomes for HIV-infected children and adolescents.   **NOTE: Mechanisms are unpacked in the process evaluation article** |

**Table 3 – Outcomes**

| - **Please make note of all (intended or not) outcomes. Please also classify the role and functions outcomes of CHWs using the following Pillars for people-centred care:**   1. Engaging and empowering people and communities  2. Strengthening governance and accountability (Incl. Supervision, training, and support)  3. Reorienting the model of care  4. Coordinating services within and across sectors  5. Enabling environment |
| --- |
| - The trial was conducted before the implementation of the "treat all" strategy, so the effect of the intervention on viral load in children who would not have been eligible for ART at the time of the study cannot be extrapolated. - The study was conducted in a routine healthcare setting, which may limit the generalizability of the findings to other settings. - The intervention relied on community health workers, who may have varying levels of training and experience, which could impact the effectiveness of the intervention. - The study focused on children and adolescents in Zimbabwe, so the findings may not be applicable to other populations or countries. - The study did not assess the long-term sustainability and cost-effectiveness of the intervention, which could be important considerations for scaling up the intervention in other settings.   The study did not explore contextual factors that could influence the implementation of the intervention, highlighting the need for further research in this area.  Outcomes according to PCC:  **Engaging and empowering people and communities:**   - The intervention provided structured support visits by trained community health workers to caregivers, empowering them with knowledge and support to engage in the care of HIV-infected children and adolescents. - The intervention focused on caregivers, who are key mediators to children accessing care, and showed an impact across the HIV care cascade, improving engagement with care and treatment outcomes.   **Strengthening governance and accountability (Incl. Supervision, training, and support):**   - The intervention utilized existing community health workers, who already exist in many African health systems, to provide support visits to caregivers, ensuring scalability and sustainability. - The intervention was implemented in routine health-care settings, following national guidelines for HIV care, and supported by research nurses, routine clinical staff, and a study physician.   **Reorienting the model of care:**   - The intervention added structured support visits by community health workers to the decentralised primary health-care clinic-based HIV care, providing a more patient-centered approach.   **Coordinating services within and across sectors:**   - The intervention was conducted in seven communities in Harare, Zimbabwe, where primary health-care clinics provided acute and antenatal care services, including provider-initiated HIV testing and counselling.   **Enabling environment:**   - The intervention was implemented in a routine health-care setting, utilizing existing community health workers, and had high potential for scalability and implementation within weak health systems. |

**Table 4 – If, then statements depicting the CMOs.**

| **Please detail the CMOs for the study you reviewed using the If (Context), Then (Mechanism) statements.** |
| --- |
| **If, Then statement,**  **The Mechanisms below comes from the process evaluation article and the “lessons learnt” article of the same study.**     - If CHWs receive ongoing intensive training, supervision and mentoring (equipping CHWs with the necessary knowledge and skills), then CHWs will feel confident and competent to do their tasks effectively, leading to increased job satisfaction over time. - If there are formalized links between CHWs and clinics, CHWs will feel valued and recognized, fostering motivation and job satisfaction, which leads to retention, Integration into the formal health system and increased effectiveness in delivering healthcare services to the community. - If CHWs' have an understanding of the community they serve or understand the local context, then this familiarity builds a sense of trust among community members as they perceive CHWs as individuals who understand and respect their local customs and challenges, leading to tailored services that meet specific needs of the community effectively. - If CHWs have personal experiences related to the condition of the patient, either through their own lives or within their communities, then CHWs can empathize; and this shared experience can create a deeper connection with community members, fostering trust and acceptability of the CHWs as credible sources of support and information, As a result, community members will be more to engage with CHWs, follow their advice, and adhere to treatment plans. |

**Test for relevance (Pearson et al. 2012; 2015; Brennan et al. 2017)**

| **Conceptually Rich** | **Thicker description’ but not ‘conceptually rich’** | **Conceptually Thin** |
| --- | --- | --- |
| Unambiguous theoretical concepts are described in sufficient depth. | Description of programme theory or sufficient information to enable it to ‘surface’. | Insufficient information to enable the programme theory to surface. |
| Relationships between, amongst concepts are clearly articulated. | Consideration of the context in which the programme takes place. | Limited or no consideration of the context in which the programme took place. |
| Concepts are sufficiently developed, defined to enable understanding without the reader needing to have first-hand experience of an area of practice. | Discussion of the differences between the design and orientation of programme theory (what was intended) and implementation (what really happened). | Limited or no discussion of the differences between the design and orientation of programme theory (what was intended) and implementation (what really happened). |
| Concepts are grounded strongly in a cited body of literature. | Recognition and discussion of the strengths/weaknesses of the implemented programme. | Limited or no discussion of the strengths/ weaknesses of the implemented programme. |
| Concepts are parsimonious (i.e., provide the simplest, but not over-simplified, explanation) | Some attempt to explain anomalous results and findings with reference to context and data. | No attempts to explain anomalous results and findings with reference to context and data. |
|  | Description of the factor affecting implementation. | Limited or no description of the factors affecting implementation. |

**Test for relevance (Pearson et al. 2012; 2015; Brennan et al. 2017)**

| **Conceptually Rich** | **Thicker description’ but not ‘conceptually rich’** | **Conceptually Thin** |
| --- | --- | --- |
|  | X |  |

**Test for rigour (Ohly et al. 2017)**

|  | **Yes** | **Fairly** | **No** |
| --- | --- | --- | --- |
| The study methods are clearly reported. | X |  |  |
| The study methods are appropriate to answer RQ. | X |  |  |
| The sample characteristics enable generalizability. |  |  | X |
| Raw data supports the study findings (conclusions). | X |  |  |
| Limitations of the study are acknowledged and clearly reported. | X |  |  |

**References**

1. Wong G, Westhorp G, Manzano A, Greenhalgh J, Jagosh J, Greenhalgh T. (2016). RAMESES II

reporting standards for realist evaluations. *BMC Medicine*, 14(1), 1-18.

2. Wong G, Greenhalgh T, Westhorp G, Pawson R. (2012). Realist methods in medical education

research: what are they and what can they contribute? *Medical Education*, 46(1), 89-96.

3. Macfarlane F, Greenhalgh T, Humphrey C, Hughes J, Butler C, Pawson R. (2011). A new workforce

in the making?: A case study of strategic human resource management in a whole-system change

effort in healthcare. *Journal of Health Organization and Management*, 25(1), 55-72.

4. Lacouture A, Breton E, Guichard A, Ridde V. (2015). The concept of mechanism from a realist

approach: a scoping review to facilitate its operationalization in public health program evaluation.

*Implementation Science*, 10(1), 1-10.

5. Pearson M, Chilton R, Woods HB, Wyatt K, Ford T, Abraham C, et al. Implementing health promotion in schools: protocol for a realist systematic review of research and experience in the United Kingdom (UK). Syst Rev. 2012 Oct 20;1(1):48.

6. Pearson M, Brand SL, Quinn C, Shaw J, Maguire M, Michie S, et al. Using realist review to inform intervention development: methodological illustration and conceptual platform for collaborative care in offender mental health. Implementation Sci. 2015 Sep 28;10(1):134.

7. Brennan N, Bryce M, Pearson M, Wong G, Cooper C, Archer J. Towards an understanding of how appraisal of doctors produces its effects: a realist review. Medical Education. 2017;51(10):1002–13.

8. Ohly H, Crossland N, Dykes F, Lowe N, Hall-Moran V. A realist review to explore how low-income pregnant women use food vouchers from the UK’s Healthy Start programme. BMJ Open. 2017 Apr 21;7(4):e013731.

**A realist evaluation of the role and functions of community health workers in service of a people-centred community health system.**

**Investigator CMOc Data Extraction Sheet Data Extraction**

The purpose of this document is to extract data from the identified studies in order to formulate one or more explanatory theories that explain Context-Mechanism-Outcome Configurations (CMOC) and to gain insight into how these CMOCs contribute to optimizing the role and functions of community health workers within a people-centred community health system.

In realist methodologies, knowledge accumulation centres on our comprehension of how mechanisms behave in various contexts and the reasons behind how and why this interplay of context and mechanism leads to different outcomes (1). Please fill in the details below for the study you will be assessing. In the subsequent three tables, locate and specify the Context, Mechanism, and Outcomes (CMO) of the intervention discussed in the study you've examined. Definitions and illustrations of CMO are provided within the tables.

**Kindly fill out the information below for the study you will be reviewing.**

| **Reviewer Name** | Usangiphile Buthelezi |
| --- | --- |
| **Reference** | Goudge J, de Kadt J, Babalola O, Muteba M, Tseng YH, Malatji H, et al. Household coverage, quality and costs of care provided by community health worker teams and the determining factors: findings from a mixed methods study in South Africa. BMJ Open. 2020 Aug 20;10(8):e035578. |
| **Country** | South Africa |
| **Setting and population** | - The study was conducted in the Sedibeng Health District in South Africa. - In Sedibeng health district (Gauteng Province), at the time of the study, there were 39 CHW teams in 37 of the district’s 72 wards. - The study involved six community health worker (CHW) teams with different configurations of supervisors, some based in formal clinics and some in community health posts. - The study also included CHWs, their supervisors, clinic staff, CHW clients, facility managers, and community representatives as participants. |
| **Aim** | - The aim of the study was to assess the coverage, quality of care, and costs of services provided by community health worker (CHW) teams in vulnerable communities in Sedibeng, South Africa. |
| **Objectives** | - To examine the performance of six CHW teams with different configurations of supervisors, some based in formal clinics and some in community health posts. - To determine the factors influencing the performance of CHW teams, including training, supervision, motivation, and collaboration with other clinic staff. - To estimate the potential benefits of the CHW program and identify areas that require greater investment in terms of CHW numbers, supervisors, training, and equipment. |
| **Methodology** | - The study used a case study approach, combining qualitative and quantitative data, including a random household survey, focus group discussions, interviews, and observations of CHWs at work, to examine the performance of six CHW teams in vulnerable communities in Sedibeng, South Africa. - Trained fieldworkers conducted interviews and observations, with interview guides and observation templates revised based on feedback. - The survey captured key demographic and career history of the CHWs, while focus group discussions included descriptions of activities carried out by CHWs and the challenges they faced. - Observations of CHWs were conducted over a 4-day period, with detailed notes taken by fieldworkers. - Data from the survey and qualitative evidence were compared to examine how different CHW models functioned and why, and to assess the value for money of the different models. |
| **Intervention description** | - The study involved a 3-year intervention in the Sedibeng Health District in South Africa, where different configurations of community health worker (CHW) teams were studied. - The CHW teams had different models of supervision, including clinic-based teams supervised by a professional nurse (PN) and an enrolled nurse (EN), health post-based teams supervised by a PN and an EN, and clinic-based teams supervised by an EN only. - The CHW teams provided services to households, including household registration visits, delivering health messages, and providing referrals. - The study assessed the coverage, quality of care, and costs of the services provided by the CHW teams. - Different configurations of supervisors were examined, with some based in formal clinics and others in community health posts. - The study evaluated the training, supervision, motivation, and collaboration with other clinic staff of the CHWs. - Observations were conducted to capture the types of activities carried out by CHWs and the challenges they faced. - The study also estimated the potential benefits of the CHW program and identified the need for greater investment in terms of CHW numbers, supervisors, training, and equipment. |
| **Intervention outcomes** | - The coverage of households visited by CHWs in the past year was low, with only 17% of households visited. - CHWs conducted an estimated one to two visits per day. - During household registration visits, CHWs asked only half of the required questions. - Respondents remembered 20%-25% of the health messages delivered by CHWs in the last month, and half of them took the recommended action. - The quality of care and collaboration with other clinic staff were better when CHWs had a senior nurse supervisor. - Increasing the number of CHWs and improving supervision could potentially increase coverage to 30%-90% of households. - The service provided by CHWs was limited, and support from the local facility was crucial for providing a quality service. - Greater investment in CHW numbers, supervisors, training, and equipment is needed to realize the potential benefits of the program. - CHWs with a senior nurse supervisor had better training, supervision, motivation, and collaboration with clinic staff, leading to improved quality of care. - Teams based in a clinic, with a senior supervisor, were more likely to be better integrated into the health system and provide a higher quality service. - CHWs supervised by enrolled nurses (ENs) without senior supervision struggled to negotiate effective working relationships with other facility staff members, resulting in poorer quality care. - In sites where there was less supervision and less training, CHWs visited more households but provided poorer quality care. - Weak management and organizational structures, including inadequate supervision, led to poor quality work, low morale, and absenteeism among CHWs. |
| **Challenges and limitations** | - The low number of respondents reporting a CHW visit in the past month limited the analysis of disease-specific services. - The presence of fieldworkers during observations may have influenced the work effort of CHWs, potentially affecting the accuracy of the observations. - The study focused on vulnerable communities in the Sedibeng District, and generalization to other areas may be limited. - The study did not provide a detailed analysis of the challenges faced by CHWs in terms of resources, infrastructure, and training. - The study did not explore the perspectives of CHW clients in depth, which could have provided valuable insights into the quality of care provided. - The recall of health messages delivered by CHWs was relatively low, with respondents remembering only 20%-25% of the messages. - The presence of fieldworkers during observations may have influenced the work effort of CHWs, potentially affecting the accuracy of the observations. - Weak supervision and management structures were identified as barriers to the effective integration of CHWs into the formal health system. - Insufficient resources and infrastructure in health posts without formal clinics hindered the provision of comprehensive care. |

**Table 1 – Context**

| **Including (but not limited to) beliefs, social and cultural norms, regulations and economic factors (2,3). For example:**   - Individual capabilities (i.e. values, roles, knowledge, purpose). - Interpersonal relationships supporting the interventions (i.e. communication, collaboration, network, influences). - Institutional settings (i.e. informal rules, organisational culture, leadership, policies, resource allocation, local priorities). - Infra-structural system (i.e. political support). - Country and rurality (i.e. small or large/rural vs regional vs remote). - Socio-demographic characteristics - Funding context or source (i.e. free, personalised, group vs. individual, government funded). |
| --- |
|  |

**Table 2 – Mechanisms**

| - **“An element of reasoning and/or reactions of an individual or collective agents(s) in regard of resources available in a given context to bring about changes through the implementation of an intervention” (4). Check what are the mediators to produce outcomes (not only primary, but also secondary or unintended outcomes).** |
| --- |
| - **Training and motivation:** The training, supervision, and motivation of the CHWs were better when they had a senior nurse supervisor. This contributed to better performance and delivery of care. Motivation of CHWs was crucial for their job satisfaction and professional confidence. Teams with a PN supervisor reported higher job satisfaction and motivation. Insufficient training in EN-only sites constrained CHWs' ability to assist patients, leading to demotivation. Lack of training and unresolved working conditions affected their performance. - **Supervision and collaboration:** Support from the local facility and having a senior supervisor facilitated collaboration and improved the quality of care provided by the CHW teams. - Support from the local facility was key to providing a quality service, as it provided necessary resources and assistance to the CHW teams. - Having a senior supervisor helped to facilitate collaboration between the CHW teams and the local facility. This collaboration enhanced the coordination of care and ensured that the CHWs had access to necessary information and support. - Senior supervisors used various mechanisms such as job training, supervised household visits, and debriefing sessions to train, motivate, and monitor CHWs, improving the quality of their work. - The senior supervisors also provided education sessions about common health conditions in the community, strengthening the CHWs' knowledge base and problem-solving abilities. - Supervisors accompanied CHWs on home visits, providing on-the-spot training and correcting any practice errors, further enhancing the quality of care provided. - **Availability of resources:** CHWs often worked without necessary resources, such as equipment, stationery, uniform, or funds for transport or communication. Having necessary resources enables CHWs to provide comprehensive care and deliver services that cover a broader range of health needs. |

**Table 3 – Outcomes**

| - **Please make note of all (intended or not) outcomes. Please also classify the role and functions outcomes of CHWs using the following Pillars for people-centred care:**   1. Engaging and empowering people and communities  2. Strengthening governance and accountability (Incl. Supervision, training, and support)  3. Reorienting the model of care  4. Coordinating services within and across sectors  5. Enabling environment |
| --- |
| - The coverage of households visited by CHWs in the past year was low, with only 17% of households visited. - CHWs conducted an estimated one to two visits per day. - During household registration visits, CHWs asked only half of the required questions. - Respondents remembered 20%-25% of the health messages delivered by CHWs in the last month, and half of them took the recommended action. - The quality of care and collaboration with other clinic staff were better when CHWs had a senior nurse supervisor. - Increasing the number of CHWs and improving supervision could potentially increase coverage to 30%-90% of households. - The service provided by CHWs was limited, and support from the local facility was crucial for providing a quality service. - Greater investment in CHW numbers, supervisors, training, and equipment is needed to realize the potential benefits of the program. - CHWs with a senior nurse supervisor had better training, supervision, motivation, and collaboration with clinic staff, leading to improved quality of care. - Teams based in a clinic, with a senior supervisor, were more likely to be better integrated into the health system and provide a higher quality service. - CHWs supervised by enrolled nurses (ENs) without senior supervision struggled to negotiate effective working relationships with other facility staff members, resulting in poorer quality care. - In sites where there was less supervision and less training, CHWs visited more households but provided poorer quality care. - Weak management and organizational structures, including inadequate supervision, led to poor quality work, low morale, and absenteeism among CHWs.   **Intervention outcomes according to PCC pillars:**  **Engaging and empowering people and communities:**   - The CHW program aimed to improve access to care for vulnerable communities, empowering them to take control of their health. - CHWs conducted household visits, providing health education and delivering medication, engaging with individuals and families to address their health needs. - The program aimed to empower communities by addressing health conditions, promoting health behaviors, and making referrals to health and social services.   **Strengthening governance and accountability (Incl. Supervision, training, and support):**   - Adequate supervision and support from skilled senior supervisors were crucial for the CHW program's success. - Collaboration with other clinic staff and support from the local facility played a key role in providing a quality service. - Training and ongoing professional development of CHWs were important in equipping them with the necessary skills to provide comprehensive care.   **Reorienting the model of care:**   - The CHW program aimed to provide a comprehensive and preventative model of care, addressing a range of health needs. - CHWs conducted household visits, monitoring immunization, chronic medication adherence, and screening for malnutrition and tuberculosis. - The program focused on promoting health behaviors and delivering care in the community, shifting away from a solely facility-based model.   **Coordinating services within and across sectors:**   - CHWs collaborated with other clinic staff, ensuring coordination and integration of care within the health system. - The program aimed to establish effective linkages with the formal health system, being perceived as an integral member of the health team. - CHWs made referrals to health and social services, ensuring individuals received the necessary care and support.   **Enabling environment:**   - The success of the CHW program relied on greater investment in numbers of CHWs, supervisors, training, and equipment. - Availability of resources, such as equipment and funds for transport or mobile communication, enabled CHWs to effectively carry out their duties. - Support from the local facility and collaboration with clinic staff created an enabling environment for the program's implementation and delivery of quality care. |

**Table 4 – If, then statements depicting the CMOs.**

| **Please detail the CMOs for the study you reviewed using the If (Context), Then (Mechanism) statements.** |
| --- |
| **If, Then statements**   - If CHWs receive support from the government to undertake their community-based tasks (i.e. equipment, stationery, uniform, or funds for transport or communication), then CHWs will feel motivated to perform their roles and responsibilities, leading to provision of care that is comprehensive and that covers a broader range of health needs. - If CHWs received supportive supervision and training from their senior supervisors (i.e. job training, supervised household visits, and debriefing sessions to train, motivate, and monitor CHWs), then CHWs will develop professional confidence and competence to undertake their duties, leading to enhanced quality of care. - If there is a senior supervisor (roving nurse) in the CHW team, who can negotiate effective working relationships between CHWs and other facility staff members, then CHWs will feel supported and respected by other healthcare professionals and perceive themselves as integral members of the healthcare system, leading to improved collaboration, enhanced referral systems, and improved overall performance of CHWs and delivery of care. |

**Test for relevance (Pearson et al. 2012; 2015; Brennan et al. 2017)**

| **Conceptually Rich** | **Thicker description’ but not ‘conceptually rich’** | **Conceptually Thin** |
| --- | --- | --- |
| Unambiguous theoretical concepts are described in sufficient depth. | Description of programme theory or sufficient information to enable it to ‘surface’. | Insufficient information to enable the programme theory to surface. |
| Relationships between, amongst concepts are clearly articulated. | Consideration of the context in which the programme takes place. | Limited or no consideration of the context in which the programme took place. |
| Concepts are sufficiently developed, defined to enable understanding without the reader needing to have first-hand experience of an area of practice. | Discussion of the differences between the design and orientation of programme theory (what was intended) and implementation (what really happened). | Limited or no discussion of the differences between the design and orientation of programme theory (what was intended) and implementation (what really happened). |
| Concepts are grounded strongly in a cited body of literature. | Recognition and discussion of the strengths/weaknesses of the implemented programme. | Limited or no discussion of the strengths/ weaknesses of the implemented programme. |
| Concepts are parsimonious (i.e., provide the simplest, but not over-simplified, explanation) | Some attempt to explain anomalous results and findings with reference to context and data. | No attempts to explain anomalous results and findings with reference to context and data. |
|  | Description of the factor affecting implementation. | Limited or no description of the factors affecting implementation. |

**Test for relevance (Pearson et al. 2012; 2015; Brennan et al. 2017)**

| **Conceptually Rich** | **Thicker description’ but not ‘conceptually rich’** | **Conceptually Thin** |
| --- | --- | --- |
| X |  |  |

**Test for rigour (Ohly et al. 2017)**

|  | **Yes** | **Fairly** | **No** |
| --- | --- | --- | --- |
| The study methods are clearly reported. | X |  |  |
| The study methods are appropriate to answer RQ. | X |  |  |
| The sample characteristics enable generalizability. |  |  | X |
| Raw data supports the study findings (conclusions). | X |  |  |
| Limitations of the study are acknowledged and clearly reported. | X |  |  |

**References**

1. Wong G, Westhorp G, Manzano A, Greenhalgh J, Jagosh J, Greenhalgh T. (2016). RAMESES II

reporting standards for realist evaluations. *BMC Medicine*, 14(1), 1-18.

2. Wong G, Greenhalgh T, Westhorp G, Pawson R. (2012). Realist methods in medical education

research: what are they and what can they contribute? *Medical Education*, 46(1), 89-96.

3. Macfarlane F, Greenhalgh T, Humphrey C, Hughes J, Butler C, Pawson R. (2011). A new workforce

in the making?: A case study of strategic human resource management in a whole-system change

effort in healthcare. *Journal of Health Organization and Management*, 25(1), 55-72.

4. Lacouture A, Breton E, Guichard A, Ridde V. (2015). The concept of mechanism from a realist

approach: a scoping review to facilitate its operationalization in public health program evaluation.

*Implementation Science*, 10(1), 1-10.

5. Pearson M, Chilton R, Woods HB, Wyatt K, Ford T, Abraham C, et al. Implementing health promotion in schools: protocol for a realist systematic review of research and experience in the United Kingdom (UK). Syst Rev. 2012 Oct 20;1(1):48.

6. Pearson M, Brand SL, Quinn C, Shaw J, Maguire M, Michie S, et al. Using realist review to inform intervention development: methodological illustration and conceptual platform for collaborative care in offender mental health. Implementation Sci. 2015 Sep 28;10(1):134.

7. Brennan N, Bryce M, Pearson M, Wong G, Cooper C, Archer J. Towards an understanding of how appraisal of doctors produces its effects: a realist review. Medical Education. 2017;51(10):1002–13.

8. Ohly H, Crossland N, Dykes F, Lowe N, Hall-Moran V. A realist review to explore how low-income pregnant women use food vouchers from the UK’s Healthy Start programme. BMJ Open. 2017 Apr 21;7(4):e013731.

**A realist evaluation of the role and functions of community health workers in service of a people-centred community health system.**

**Investigator CMOc Data Extraction Sheet Data Extraction**

The purpose of this document is to extract data from the identified studies in order to formulate one or more explanatory theories that explain Context-Mechanism-Outcome Configurations (CMOC) and to gain insight into how these CMOCs contribute to optimizing the role and functions of community health workers within a people-centred community health system.

In realist methodologies, knowledge accumulation centres on our comprehension of how mechanisms behave in various contexts and the reasons behind how and why this interplay of context and mechanism leads to different outcomes (1). Please fill in the details below for the study you will be assessing. In the subsequent three tables, locate and specify the Context, Mechanism, and Outcomes (CMO) of the intervention discussed in the study you've examined. Definitions and illustrations of CMO are provided within the tables.

**Kindly fill out the information below for the study you will be reviewing.**

| **Reviewer Name** | Usangiphile Buthelezi |
| --- | --- |
| **Reference** | Hayward SE, Vanqa N, Makanda G, Tisile P, Ngwatyu L, Foster I, et al. “As a patient I do not belong to the clinic, I belong to the community.” Co-developing a multi-level, person-centred tuberculosis stigma intervention in Cape Town, South Africa. Res Sq. 2024 Feb 8;rs.3.rs-3921970. |
| **Country** | South Africa |
| **Setting and population** | - Khayelitsha, located in the Western Cape Province of South Africa, was the study setting. - This area is a mix of formal and informal housing and has one of the highest incidences of TB in the region. - The study involved individuals from a Primary Health Clinic called Luvuyo, where the TB program was active. Participants included people with TB, caregivers, TB counsellors, nurses, and community health workers. - The research aimed to include a diverse group of participants by considering factors like age, gender, type of TB, and roles in healthcare. - The population studied included individuals directly impacted by TB, such as those with current or past TB experiences, as well as those involved in providing TB care and support. |
| **Aim** | - The aim of the research was to develop effective interventions to reduce stigma related to TB. |
| **Objectives** | - The study focused on using participatory approaches, like Community-Based Participatory Research (CBPR), to involve TB-affected communities in the research process. - The goal was to empower the community representatives, equalize power dynamics between researchers and participants, and promote cultural sensitivity. - By incorporating Human-Centered Design (HCD) into CBPR, the research aimed to create stigma reduction interventions that are more person-centered, innovative, and widely adopted. - Overall, the aim was to maximize the acceptability and reach of stigma reduction interventions, especially for marginalized groups affected by TB, to improve health equity and outcomes. |
| **Methodology** | - The research utilized a mixed-methods approach combining Community-Based Participatory Research (CBPR) and Human-Centered Design (HCD). - Community representatives, TB-affected individuals, healthcare providers, and researchers collaborated in workshops to co-create interventions. - Data collection methods included interviews, focus groups, and workshops to gather diverse perspectives on TB-related stigma. - The CBPR approach ensured community engagement and empowerment throughout the research process. - Researchers used thematic analysis to identify key themes and patterns from the collected data. - The study followed ethical guidelines, obtaining informed consent and maintaining confidentiality of participants' information. - By involving stakeholders in intervention development and using a mixed-methods design, the research aimed to create culturally sensitive and effective TB stigma reduction strategies. |
| **Intervention description** | - The research utilized a mixed-methods approach combining Community-Based Participatory Research (CBPR) and Human-Centered Design (HCD). - Community representatives, TB-affected individuals, healthcare providers, and researchers collaborated in workshops to co-create interventions. - Data collection methods included interviews, focus groups, and workshops to gather diverse perspectives on TB-related stigma. - The CBPR approach ensured community engagement and empowerment throughout the research process. - Researchers used thematic analysis to identify key themes and patterns from the collected data. - The study followed ethical guidelines, obtaining informed consent and maintaining confidentiality of participants' information. - By involving stakeholders in intervention development and using a mixed-methods design, the research aimed to create culturally sensitive and effective TB stigma reduction strategies. |
| **Intervention outcomes** | - The study showed that the TB stigma intervention led to a decrease in missed treatment doses among TB and HIV patients, indicating a positive impact on treatment adherence. - By involving affected individuals and health workers in the intervention development process, the study aimed to address the limitations of previous stigma interventions and enhance effectiveness. - Components of the intervention, such as education, empowerment, engagement, and innovation, were identified as key factors contributing to its success in tackling TB stigma. - The human-centred design approach allowed for a more personalized and effective intervention by drawing on real-life experiences and service gaps highlighted by the study. |
| **Challenges and limitations** | - Previous TB stigma interventions faced challenges due to limited evidence and inconsistent designs. - The study noted that stigma interventions often focused only on individual levels, which might not fully address the widespread impact of stigma. - Developing effective interventions required involvement from affected individuals and health workers to ensure relevance and success. - Stigma intervention development faced limitations such as inadequate insights from those directly affected by TB stigma. |

**Table 1 – Context**

| **Including (but not limited to) beliefs, social and cultural norms, regulations and economic factors (2,3). For example:**   - Individual capabilities (i.e. values, roles, knowledge, purpose). - Interpersonal relationships supporting the interventions (i.e. communication, collaboration, network, influences). - Institutional settings (i.e. informal rules, organisational culture, leadership, policies, resource allocation, local priorities). - Infra-structural system (i.e. political support). - Country and rurality (i.e. small or large/rural vs regional vs remote). - Socio-demographic characteristics: - Funding context or source (i.e. free, personalised, group vs. individual, government funded). |
| --- |
| - The study was conducted in Khayelitsha, a low-resourced township in South Africa, known for its high incidence of TB. - Participants included individuals with TB, caregivers, TB counsellors, nurses, and community health workers, selected for their diverse backgrounds and roles within the TB program. - The research was focused on designing a stigma intervention for TB survivors to help decrease stigma levels and drive policy changes. |

**Table 2 – Mechanisms**

| - **“An element of reasoning and/or reactions of an individual or collective agents(s) in regard of resources available in a given context to bring about changes through the implementation of an intervention” (4). Check what are the mediators to produce outcomes (not only primary, but also secondary or unintended outcomes).** |
| --- |
| - Participants highlighted the importance of **education** to dispel myths and increase awareness about TB, ensuring accurate information reaches the community. - Empowering TB survivors to be **peer research associates** was a crucial factor in informing stigma interventions and driving policy change. - Building **rapport** between health workers and TB survivors through stigma training led by those with lived experiences enhanced the effectiveness of the intervention at the institutional level. - Incorporating **community representatives** throughout the research process promoted cultural sensitivity and equalized power dynamics, strengthening the intervention's impact. - Providing **examples of successful TB treatment outcomes** helped individuals realize that TB is curable, fostering confidence and motivation to engage in care. |

**Table 3 – Outcomes**

| - **Please make note of all (intended or not) outcomes. Please also classify the role and functions outcomes of CHWs using the following Pillars for people-centred care:**   1. Engaging and empowering people and communities  2. Strengthening governance and accountability (Incl. Supervision, training, and support)  3. Reorienting the model of care  4. Coordinating services within and across sectors  5. Enabling environment |
| --- |
| - The study showed that the TB stigma intervention led to a decrease in missed treatment doses among TB and HIV patients, indicating a positive impact on treatment adherence. - By involving affected individuals and health workers in the intervention development process, the study aimed to address the limitations of previous stigma interventions and enhance effectiveness. - Components of the intervention, such as education, empowerment, engagement, and innovation, were identified as key factors contributing to its success in tackling TB stigma. - The human-centred design approach allowed for a more personalized and effective intervention by drawing on real-life experiences and service gaps highlighted by the study.   **Outcomes according to PCC pillars:**   - By **engaging** TB survivors as peer research associates, the intervention successfully **empowered communities** by involving those directly affected by TB in stigma reduction strategies within their own context. This approach aligned with the behaviour change strategies of education and empowerment. - **Strengthening governance and accountability** was achieved through the recommendation of health worker stigma training that was informed by TB survivors, ensuring a more supportive and informed healthcare environment. This contributed to enhancing service delivery and reducing stigma at the institutional level. - **The model of care was reoriented** towards a community-centred approach, integrating TB support groups and family-centred counselling to dispel myths and provide vital support at the interpersonal level. This shift emphasized not only treatment but holistic well-being, fostering stronger community bonds. - **Through coordinating services within and across sectors,** the intervention facilitated multisectoral collaboration to address TB stigma comprehensively. This included incorporating TB awareness in school curricula and increasing community outreach through Community Health Workers, ensuring a more coordinated and impactful approach at the community level. - **Creating an enabling environment** involved raising awareness through TB survivor-led events and incorporating Human-Centred Design (HCD) principles. These actions not only increased knowledge but also promoted innovation and community participation, fostering a supportive and conducive atmosphere for stigma reduction interventions. |

**Table 4 – If, then statements depicting the CMOs.**

| **Please detail the CMOs for the study you reviewed using the If (Context), Then (Mechanism) statements.** |
| --- |
| **IF** Community Health Workers (CHWs) empower TB survivors (through education and training) to be peer research associates that implement the TB stigma intervention to the community, **THEN** this fosters cultural sensitivity, equalizes power dynamics, increases confidence and motivation to engage in care, and helps individuals to realize TB is curable, **BECAUSE** involving TB survivors directly in the intervention process ensures that the strategies are informed by lived experiences, promotes trust and relatability, and demonstrates successful outcomes, thereby encouraging others to adhere to treatment and participate in stigma reduction efforts.  **IF** the model of care is community-centred, integrating community support structures and families, and providing support at the interpersonal level, **THEN** this fosters stronger community bonds, **BECAUSE** involving community support structures and families creates a supportive network that encourages individuals to seek and adhere to treatment, promotes shared responsibility for health, and enhances the overall well-being of the community, leading to improved health outcomes. |

**Test for relevance (Pearson et al. 2012; 2015; Brennan et al. 2017)**

| **Conceptually Rich** | **Thicker description’ but not ‘conceptually rich’** | **Conceptually Thin** |
| --- | --- | --- |
| Unambiguous theoretical concepts are described in sufficient depth. | Description of programme theory or sufficient information to enable it to ‘surface’. | Insufficient information to enable the programme theory to surface. |
| Relationships between, amongst concepts are clearly articulated. | Consideration of the context in which the programme takes place. | Limited or no consideration of the context in which the programme took place. |
| Concepts are sufficiently developed, defined to enable understanding without the reader needing to have first-hand experience of an area of practice. | Discussion of the differences between the design and orientation of programme theory (what was intended) and implementation (what really happened). | Limited or no discussion of the differences between the design and orientation of programme theory (what was intended) and implementation (what really happened). |
| Concepts are grounded strongly in a cited body of literature. | Recognition and discussion of the strengths/weaknesses of the implemented programme. | Limited or no discussion of the strengths/ weaknesses of the implemented programme. |
| Concepts are parsimonious (i.e., provide the simplest, but not over-simplified, explanation) | Some attempt to explain anomalous results and findings with reference to context and data. | No attempts to explain anomalous results and findings with reference to context and data. |
|  | Description of the factor affecting implementation. | Limited or no description of the factors affecting implementation. |

**Test for relevance (Pearson et al. 2012; 2015; Brennan et al. 2017)**

| **Conceptually Rich** | **Thicker description’ but not ‘conceptually rich’** | **Conceptually Thin** |
| --- | --- | --- |
|  | X |  |

**Test for rigour (Ohly et al. 2017)**

|  | **Yes** | **Fairly** | **No** |
| --- | --- | --- | --- |
| The study methods are clearly reported. | X |  |  |
| The study methods are appropriate to answer RQ. | X |  |  |
| The sample characteristics enable generalizability. |  |  | X |
| Raw data supports the study findings (conclusions). |  | X |  |
| Limitations of the study are acknowledged and clearly reported. | X |  |  |

**References**

1. Wong G, Westhorp G, Manzano A, Greenhalgh J, Jagosh J, Greenhalgh T. (2016). RAMESES II

reporting standards for realist evaluations. *BMC Medicine*, 14(1), 1-18.

2. Wong G, Greenhalgh T, Westhorp G, Pawson R. (2012). Realist methods in medical education

research: what are they and what can they contribute? *Medical Education*, 46(1), 89-96.

3. Macfarlane F, Greenhalgh T, Humphrey C, Hughes J, Butler C, Pawson R. (2011). A new workforce

in the making?: A case study of strategic human resource management in a whole-system change

effort in healthcare. *Journal of Health Organization and Management*, 25(1), 55-72.

4. Lacouture A, Breton E, Guichard A, Ridde V. (2015). The concept of mechanism from a realist

approach: a scoping review to facilitate its operationalization in public health program evaluation.

*Implementation Science*, 10(1), 1-10.

5. Pearson M, Chilton R, Woods HB, Wyatt K, Ford T, Abraham C, et al. Implementing health promotion in schools: protocol for a realist systematic review of research and experience in the United Kingdom (UK). Syst Rev. 2012 Oct 20;1(1):48.

6. Pearson M, Brand SL, Quinn C, Shaw J, Maguire M, Michie S, et al. Using realist review to inform intervention development: methodological illustration and conceptual platform for collaborative care in offender mental health. Implementation Sci. 2015 Sep 28;10(1):134.

7. Brennan N, Bryce M, Pearson M, Wong G, Cooper C, Archer J. Towards an understanding of how appraisal of doctors produces its effects: a realist review. Medical Education. 2017;51(10):1002–13.

8. Ohly H, Crossland N, Dykes F, Lowe N, Hall-Moran V. A realist review to explore how low-income pregnant women use food vouchers from the UK’s Healthy Start programme. BMJ Open. 2017 Apr 21;7(4):e013731.

**A realist evaluation of the role and functions of community health workers in service of a people-centred community health system.**

**Investigator CMOc Data Extraction Sheet Data Extraction**

The purpose of this document is to extract data from the identified studies in order to formulate one or more explanatory theories that explain Context-Mechanism-Outcome Configurations (CMOC) and to gain insight into how these CMOCs contribute to optimizing the role and functions of community health workers within a people-centred community health system.

In realist methodologies, knowledge accumulation centres on our comprehension of how mechanisms behave in various contexts and the reasons behind how and why this interplay of context and mechanism leads to different outcomes (1). Please fill in the details below for the study you will be assessing. In the subsequent three tables, locate and specify the Context, Mechanism, and Outcomes (CMO) of the intervention discussed in the study you've examined. Definitions and illustrations of CMO are provided within the tables.

**Kindly fill out the information below for the study you will be reviewing.**

| **Reviewer Name** | Usangiphile Buthelezi |
| --- | --- |
| **Reference** | Feldhaus I, Silverman M, LeFevre AE, Mpembeni R, Mosha I, Chitama D, et al. Equally able, but unequally accepted: Gender differentials and experiences of community health volunteers promoting maternal, newborn, and child health in Morogoro Region, Tanzania. International Journal for Equity in Health. 2015 Aug 25;14(1):70. |
| **Country** | Tanzania |
| **Setting and population** | - The study was conducted in Morogoro Region, Tanzania, which has a population of over 2.2 million people. It is located 200 km southwest of Dar es Salaam and covers an area of 70,000 km2. - The population of Tanzania is the fifth most populous in Sub-Saharan Africa, with over 49.6 million people. - The Integrated MNCH Program in Morogoro Region recruited male and female volunteers as Community Health Workers (CHWs) from various villages. The CHWs were selected by village leaders and ideally had a minimum of secondary education. - The study included all CHWs who received training from the Integrated MNCH Program between December 2012 and July 2013 in five districts of Morogoro Region. A total of 228 CHWs were interviewed, with 55% being male and 45% female. - A sub-sample of the CHW population was also recruited for a qualitative study in three districts: Morogoro Rural, Kilosa, and Gairo. The study included CHWs, CHW supervisors, village leaders, and health committee members from 12 villages. |
| **Aim** | - The aim of the study was to examine potential gender differences in knowledge, health promotion activities, and client acceptability among Community Health Workers (CHWs) involved in the Integrated Maternal, Newborn, and Child Health (MNCH) Program in Morogoro Region, Tanzania |
| **Objectives** | - The study aimed to assess the knowledge levels of male and female CHWs in various domains of maternal, newborn, and child health. - The study also aimed to explore the experiences and perspectives of CHWs, their supervisors, community leaders, and health committee members regarding gender dynamics and acceptability of CHW services. |
| **Methodology** | - The study utilized a mixed-methods approach, combining quantitative and qualitative data collection and analysis. - All CHWs who received training from the Integrated MNCH Program in Morogoro Region between December 2012 and July 2013 were surveyed, and information on their health promotion activities was drawn from their registers. - Quantitative analysis included bi-and multivariate analyses to examine CHW socio-demographic characteristics, knowledge, and health promotion activities. Composite scores generated across ten knowledge domains were used in ordered logistic regression models. - Thematic analysis was conducted on 60 semi-structured interviews with CHWs, their supervisors, community leaders, and health committee members from 12 villages in three districts. - The interviews were conducted in Swahili and covered various aspects related to CHW experiences, gender dynamics, and acceptability of CHW services. - The study also collected data on CHW contact information from facility-based supervisors and conducted interviews with CHWs in their homes to maintain privacy and observe socioeconomic status. |
| **Intervention description** | - The study examined the gender differentials and experiences of community health workers (CHWs) promoting maternal, newborn, and child health (MNCH) in Morogoro Region, Tanzania. - The intervention involved training both male and female CHWs in MNCH knowledge and health promotion activities. - The CHWs conducted home visits and community health education meetings to promote MNCH. |
| **Intervention outcomes** | - The study found that there were no significant differences in CHW knowledge, or the number of health promotion activities undertaken by gender. - However, gender did influence CHW acceptability, with women more likely to disclose pregnancies earlier to female CHWs and men more comfortable discussing sexual and reproductive concerns with male CHWs. - Pairing male and female CHWs was suggested as a potential strategy to address gender issues and improve community acceptance of CHWs. - The intervention aimed to promote maternal, newborn, and child health (MNCH) through CHWs conducting home visits and community health education meetings. - The study highlighted the importance of considering gender in community-level MNCH interventions and CHW programs to improve service delivery outcomes and promote gender equity. - The research suggested that both male and female CHWs are equally capable of successful health promotion efforts, but further attention is required to address gender dynamics and acceptance of CHW counseling for reproductive health. |
| **Challenges and limitations** | - The evaluation of the Integrated MNCH Program took place soon after CHW training, reflecting short-term recall of health promotion content after training. - The study relied on self-reported data from CHWs, which may be subject to recall bias or social desirability bias. - The quality and reliability of CHW registers, which were used for data collection, have not been fully assessed. - The study did not assess the long-term knowledge retention of CHWs over time. - The study focused on CHWs in rural Tanzania, limiting the generalizability of the findings to other contexts. - The study did not explore the perspectives of community members who interacted with CHWs, which could provide additional insights into gender dynamics and acceptability. - The study did not assess the impact of gender on health outcomes or the effectiveness of the MNCH interventions delivered by CHWs. |

**Table 1 – Context**

| **Including (but not limited to) beliefs, social and cultural norms, regulations, and economic factors (2,3). For example:**   - Individual capabilities (i.e. values, roles, knowledge, purpose). - Interpersonal relationships supporting the interventions (i.e. communication, collaboration, network, influences). - Institutional settings (i.e. informal rules, organisational culture, leadership, policies, resource allocation, local priorities). - Infra-structural system (i.e. political support). - Country and rurality (i.e. small or large/rural vs regional vs remote). - Socio-demographic characteristics: - Funding context or source (i.e. free, personalised, group vs. individual, government funded). |
| --- |
| - **Health System Organization:** The study was conducted within the context of the Integrated Maternal, Newborn, and Child Health (MNCH) Program in Morogoro Region, Tanzania. - **Country and Rurality:** The study took place in Morogoro Region, Tanzania, which is a rural area located southwest of Dar es Salaam. Tanzania has a population of over 49.6 million people and is the fifth most populous country in Sub-Saharan Africa. - **Country and Rurality:** The study took place in Morogoro Region, Tanzania, which is a rural area located southwest of Dar es Salaam. Tanzania has a population of over 49.6 million people and is the fifth most populous country in Sub-Saharan Africa. - **Population and Setting:** The study included CHWs, CHW supervisors, village leaders, and health committee members from 12 villages in Morogoro Region. The population of Morogoro Region is over 2.2 million people. - **Access to Resources:** The study did not provide specific information on the access to resources in the study population or setting. |

**Table 2 – Mechanisms**

| - **“An element of reasoning and/or reactions of an individual or collective agents(s) in regard of resources available in a given context to bring about changes through the implementation of an intervention” (4). Check what are the mediators to produce outcomes (not only primary, but also secondary or unintended outcomes).** |
| --- |
| - Women were more likely to disclose pregnancies earlier to female CHWs, while men were more comfortable discussing sexual and reproductive concerns with male CHWs. This suggests that gender matching between CHWs and clients can enhance acceptability and trust. - The presence of male CHWs was seen as critical in reaching out to husbands, particularly in polygamous households, and educating both husbands and wives. This highlights the importance of gender diversity in CHW teams for effective health promotion. This is because: - The cultural norms in Tanzania dictate that male relatives should not discuss reproductive health matters with their female relatives or kin, and vice versa. Having male CHWs allowed for more effective communication and counseling on sex-specific sensitive topics related to sexual and reproductive health. - The presence of male CHWs helped to address gender dynamics and build trust with husbands, who may have initially been skeptical of CHW visits. This contributed to increased acceptance and utilization of MNCH services. - Performing home visits in pairs, with both male and female CHWs, helped to mitigate gender barriers and encourage the engagement of men in maternal, newborn, and child health (MNCH) services. - Perceptions of CHWs as credible and knowledgeable health providers contributed to their acceptability and effectiveness in delivering maternal, newborn, and child health (MNCH) interventions. - Perceived support from CHWs, especially in terms of providing counseling and guidance, fostered trust and rapport with community members, leading to increased acceptance and utilization of MNCH services. |

**Table 3 – Outcomes**

| - **Please make note of all (intended or not) outcomes. Please also classify the role and functions outcomes of CHWs using the following Pillars for people-centred care:**   1. Engaging and empowering people and communities  2. Strengthening governance and accountability (Incl. Supervision, training, and support)  3. Reorienting the model of care  4. Coordinating services within and across sectors  5. Enabling environment |
| --- |
| - The study found that there were no significant differences in CHW knowledge or the number of health promotion activities undertaken by gender. - However, gender did influence CHW acceptability, with women more likely to disclose pregnancies earlier to female CHWs and men more comfortable discussing sexual and reproductive concerns with male CHWs. - Pairing male and female CHWs was suggested as a potential strategy to address gender issues and improve community acceptance of CHWs. - The intervention aimed to promote maternal, newborn, and child health (MNCH) through CHWs conducting home visits and community health education meetings. - The study highlighted the importance of considering gender in community-level MNCH interventions and CHW programs to improve service delivery outcomes and promote gender equity. - The research suggested that both male and female CHWs are equally capable of successful health promotion efforts, but further attention is required to address gender dynamics and acceptance of CHW counseling for reproductive health.   Outcomes by PCC pillars:   1. **Engaging and empowering people and communities:**    - Community health workers (CHWs) played a crucial role in engaging and empowering communities by providing health promotion activities and continuous support for women before, during, and after labor.    - Male CHWs were seen as critical in reaching out to husbands, particularly in polygamous households, and educating both husbands and wives, which empowered the community by involving men in discussions about reproductive and family health. 2. **Strengthening governance and accountability (Incl. Supervision, training, and support):**    - CHWs received training from the Integrated MNCH Program, which equipped them with the necessary knowledge and skills to deliver effective MNCH interventions.    - Supervision and support from their supervisors, community leaders, and health committee members helped CHWs overcome challenges and improve their performance. 3. **Reorienting the model of care:**    - The introduction of a MNCH CHW cadre in Morogoro Region, Tanzania reoriented the model of care by enlisting both male and female CHWs, challenging the assumption that female-only CHW programs empower women.    - Pairing male and female CHWs helped address gender issues in CHW acceptance and delivery of reproductive health counseling, contributing to improved outcomes. 4. **Coordinating services within and across sectors:**    - CHWs served as a link between the community and the formal health system, coordinating services and referrals to health facilities for further care.    - The Integrated MNCH Program provided job aids to CHWs, which facilitated coordination and standardized the delivery of health promotion activities. 5. **Enabling environment:**    - Strengthened health systems, appropriate training, supervision, and remuneration for CHWs were identified as key factors in creating an enabling environment for effective MNCH interventions. |

**Table 4 – If, then statements depicting the CMOs.**

| **Please detail the CMOs for the study you reviewed using the If (Context), Then (Mechanism) statements.** |
| --- |
| **If, Then statements**   - If there is gender matching between CHWs, particularly when addressing gender-sensitive topics like reproductive health, family planning, and maternal care, then patients will choose CHWs based on their comfort, trust, and cultural norms. This approach enhances the acceptability of CHW services within the community, reduces stigma and access barriers, and fosters greater community engagement. |

**Test for relevance (Pearson et al. 2012; 2015; Brennan et al. 2017)**

| **Conceptually Rich** | **Thicker description’ but not ‘conceptually rich’** | **Conceptually Thin** |
| --- | --- | --- |
| Unambiguous theoretical concepts are described in sufficient depth. | Description of programme theory or sufficient information to enable it to ‘surface’. | Insufficient information to enable the programme theory to surface. |
| Relationships between, amongst concepts are clearly articulated. | Consideration of the context in which the programme takes place. | Limited or no consideration of the context in which the programme took place. |
| Concepts are sufficiently developed, defined to enable understanding without the reader needing to have first-hand experience of an area of practice. | Discussion of the differences between the design and orientation of programme theory (what was intended) and implementation (what really happened). | Limited or no discussion of the differences between the design and orientation of programme theory (what was intended) and implementation (what really happened). |
| Concepts are grounded strongly in a cited body of literature. | Recognition and discussion of the strengths/weaknesses of the implemented programme. | Limited or no discussion of the strengths/ weaknesses of the implemented programme. |
| Concepts are parsimonious (i.e., provide the simplest, but not over-simplified, explanation) | Some attempt to explain anomalous results and findings with reference to context and data. | No attempts to explain anomalous results and findings with reference to context and data. |
|  | Description of the factor affecting implementation. | Limited or no description of the factors affecting implementation. |

**Test for relevance (Pearson et al. 2012; 2015; Brennan et al. 2017)**

| **Conceptually Rich** | **Thicker description’ but not ‘conceptually rich’** | **Conceptually Thin** |
| --- | --- | --- |
|  | **X** |  |

**Test for rigour (Ohly et al. 2017)**

|  | **Yes** | **Fairly** | **No** |
| --- | --- | --- | --- |
| The study methods are clearly reported. | X |  |  |
| The study methods are appropriate to answer RQ. | X |  |  |
| The sample characteristics enable generalizability. |  |  | X |
| Raw data supports the study findings (conclusions). | X |  |  |
| Limitations of the study are acknowledged and clearly reported. | X |  |  |

**References**

1. Wong G, Westhorp G, Manzano A, Greenhalgh J, Jagosh J, Greenhalgh T. (2016). RAMESES II

reporting standards for realist evaluations. *BMC Medicine*, 14(1), 1-18.

2. Wong G, Greenhalgh T, Westhorp G, Pawson R. (2012). Realist methods in medical education

research: what are they and what can they contribute? *Medical Education*, 46(1), 89-96.

3. Macfarlane F, Greenhalgh T, Humphrey C, Hughes J, Butler C, Pawson R. (2011). A new workforce

in the making?: A case study of strategic human resource management in a whole-system change

effort in healthcare. *Journal of Health Organization and Management*, 25(1), 55-72.

4. Lacouture A, Breton E, Guichard A, Ridde V. (2015). The concept of mechanism from a realist

approach: a scoping review to facilitate its operationalization in public health program evaluation.

*Implementation Science*, 10(1), 1-10.

5. Pearson M, Chilton R, Woods HB, Wyatt K, Ford T, Abraham C, et al. Implementing health promotion in schools: protocol for a realist systematic review of research and experience in the United Kingdom (UK). Syst Rev. 2012 Oct 20;1(1):48.

6. Pearson M, Brand SL, Quinn C, Shaw J, Maguire M, Michie S, et al. Using realist review to inform intervention development: methodological illustration and conceptual platform for collaborative care in offender mental health. Implementation Sci. 2015 Sep 28;10(1):134.

7. Brennan N, Bryce M, Pearson M, Wong G, Cooper C, Archer J. Towards an understanding of how appraisal of doctors produces its effects: a realist review. Medical Education. 2017;51(10):1002–13.

8. Ohly H, Crossland N, Dykes F, Lowe N, Hall-Moran V. A realist review to explore how low-income pregnant women use food vouchers from the UK’s Healthy Start programme. BMJ Open. 2017 Apr 21;7(4):e013731.

**A realist evaluation of the role and functions of community health workers in service of a people-centred community health system.**

**Investigator CMOc Data Extraction Sheet Data Extraction**

The purpose of this document is to extract data from the identified studies in order to formulate one or more explanatory theories that explain Context-Mechanism-Outcome Configurations (CMOC) and to gain insight into how these CMOCs contribute to optimizing the role and functions of community health workers within a people-centred community health system.

In realist methodologies, knowledge accumulation centres on our comprehension of how mechanisms behave in various contexts and the reasons behind how and why this interplay of context and mechanism leads to different outcomes (1). Please fill in the details below for the study you will be assessing. In the subsequent three tables, locate and specify the Context, Mechanism, and Outcomes (CMO) of the intervention discussed in the study you've examined. Definitions and illustrations of CMO are provided within the tables.

**Kindly fill out the information below for the study you will be reviewing.**

| **Reviewer Name** | Usangiphile Buthelezi |
| --- | --- |
| **Reference** | Goudge J, Babalola O, Malatji H, Levin J, Thorogood M, Griffiths F. **The effect of a roving nurse mentor on household coverage and quality of care provided by community health worker teams in South Africa: a longitudinal study with a before, after and 6 months post design.** BMC health services research. 2023 Feb 22;23(1):186. |
| **Country** | South Africa |
| **Setting** | - The study was conducted in two vulnerable communities in South Africa. - These communities were located in the Sedibeng District, which is relatively well off by South African standards but still faced challenges such as insufficient food, inadequate housing, and limited access to health services. - The residents in these communities relied on government grants as a source of income and faced high levels of illness. - The housing in these communities consisted of government-provided small brick houses or shacks made of plastic and re-used corrugated iron. - The communities were located 30 km away from the nearest town, which affected their access to transport networks, water, and electricity. |
| **Population** | - The study population consisted of community health workers (CHWs), their supervisors, a roving nurse, and household members in two vulnerable communities in South Africa. |
| **Aim** | - The aim of the study was to assess the effect of a roving nurse mentor on the coverage and quality of care provided by community health worker (CHW) teams in two vulnerable communities in South Africa . |
| **Objectives** | - The aim of the intervention was to mentor the CHW team as a whole, including supervisors and CHWs, by increasing their clinical knowledge and skills in client engagement. It also aimed to provide supportive supervision to improve supervision skills and enhance the understanding of its benefits. - The study aimed to evaluate the impact of the intervention on the quantity and quality of CHW work, such as the number of households visited, the range of tasks performed, and the provision of appropriate health messages. - Additionally, the study aimed to strengthen relationships between the CHW team and clinic staff, as well as with community organizations and political structures. |
| **Methodology** | - The study utilized a longitudinal design with a before, after, and 6 months post-intervention approach. - Three household surveys were conducted at different time points to assess household coverage of the CHW service. - Structured observations of CHWs working in households were carried out to assess the quality of care provided. - The intervention involved the implementation of a roving nurse mentor to build the skills of supervisors and CHW teams. - Data collection methods included household surveys, stratified sampling, and logistic regression analysis. - An observation tool called QoCAT (Quality of Care Assessment Tool) was developed to assess the quality of care delivered by CHWs before and after the intervention. - Patient and public involvement were not directly included in the study design, but feedback sessions were conducted with CHW teams and facility, district, and provincial managers. |
| **Intervention description** | - The intervention in the study involved the employment of a roving nurse mentor on a full-time basis, who had experience in community nursing and adult training. - The nurse mentor moved between two facility-based teams with junior nurse supervisors over a period of 15 months. - The aim of the intervention was to mentor the team as a whole, including supervisors and CHWs, by increasing their clinical knowledge and skills in client engagement. - The nurse mentor also provided supportive supervision to improve supervision skills and enhance the understanding of its benefits. - The intervention aimed to strengthen relationships between the CHW team and clinic staff, as well as with community organizations and political structures. - The nurse mentor had a 4-year nursing degree and 15 years of experience, including supervisory roles in other CHW programs. |
| **Intervention outcomes** | - The intervention of a roving nurse mentor led to a sustained 50% increase in the number of households visited by a CHW. - CHWs were more likely to visit new households, provide care to a greater range of people, and perform a greater range of more complex tasks. - The proportion of appropriate health messages given to household members by CHWs remained constant at approximately 50%. - CHWs were more likely to discuss with clients the barriers they were facing in accessing care and take notes during a visit. - The intervention resulted in increased CHW participation and confidence to voice concerns at the intervention facilities. - The study found that a nurse mentor providing training and supportive supervision can have a significant effect on the quantity and quality of CHW's work. |
| **Challenges and Limitations** | - The study lacked a control group, which limits the ability to compare the intervention group with a non-intervention group. - The assessment of quality of care using the QoCAT tool may have low validity, as there is no gold standard for assessing CHW quality of care. - The study experienced strike actions by CHWs during the intervention period, which may have impacted the study results. However, the number of working days lost was considered insufficient to have a significant impact. - Turnover among the fieldworker team occurred due to the long duration of the study, but extensive training and piloting were conducted with each team before fieldwork. - The study was conducted in two specific sites in South Africa, which may limit the generalizability of the findings to other settings. - The study did not assess the long-term sustainability of the intervention beyond the 6-month post-intervention period. |

**Table 1 – Context**

| **Including (but not limited to) beliefs, social and cultural norms, regulations, and economic factors. For example:**   - Individual capabilities (i.e. values, roles, knowledge, purpose). - Interpersonal relationships supporting the interventions (i.e. communication, collaboration, network, influences). - Institutional settings (i.e. informal rules, organisational culture, leadership, policies, resource allocation, local priorities). - Infra-structural system (i.e. political support). - Health System organisation - Country and rurality (i.e. small or large/rural vs regional vs remote). - Funding context or source (i.e. free, personalised, group vs. individual, government funded). |
| --- |
| **Country and rurality**  The study was conducted in South Africa, specifically in the Sedibeng District, which is relatively well off by South African standards but still faces challenges such as insufficient food, inadequate housing, and limited access to health services. The study sites were located 30 km from the nearest town, and the majority of the inhabitants were without work and relied on government grants. - the study focused on vulnerable communities in South Africa, indicating that the intervention and findings are relevant to the country's context.  **Institutional settings**  The institutional setting of the study was primarily focused on the two vulnerable communities in South Africa, with collaborations involving the University of the Witwatersrand Human Research Ethics Committee, facility, district, and provincial managers, as well as local political leaders and organizations.  **Interpersonal relationships supporting the interventions:**   - The study involved collaborations with the University of the Witwatersrand Human Research Ethics Committee for ethical clearance. - Facility, district, and provincial managers were involved in the design, conduct, reporting, and dissemination plans of the study. - Feedback sessions were conducted with CHW teams, facility, district, and provincial managers. - The nurse mentor collaborated with CHW supervisors, providing training, support, and supervision to improve the skills and performance of CHWs. - The nurse mentor also engaged with facility staff, including data clerks and the pharmacy assistant, to establish new work practices and discuss the support needed for CHWs.   **Infra-structural system**   - Meetings and workshops were held with local political leaders, relevant local organizations, and stakeholders to explain the work of CHWs and explore potential collaborations.   **Individual capabilities**   - **Community Health Workers (CHWs):** Undertook complex tasks, provided promotive and preventative care, made referrals, followed up on non-attendees, and performed a greater range of more complex tasks with the support of a nurse mentor. - **Roving Nurse Mentor:** Provided training, supportive supervision, and mentorship to CHWs and supervisors, improving their clinical knowledge, client engagement skills, supervision skills, and work systems. Established new working practices, held meetings with facility staff and local leaders, and supported CHWs and supervisors in their roles. - **CHW Supervisors:** Responsible for community engagement, received training and support from the nurse mentor, and played a role in implementing new practices and supporting CHWs. - **Household Members:** Participated in the study by giving permission for fieldworkers to observe CHWs in their households.   **Funding context**  The funding context of the study was supported by multiple organizations, including the Medical Research Council UK, DFID, ESRC, and Wellcome Trust. Additionally, the study received support from the NRF Chair in Health Systems and Policy at the Centre for Health Policy. |

**Table 2 – Mechanisms**

| - **“An element of reasoning and/or reactions of an individual or collective agents(s) in regard of resources available in a given context to bring about changes through the implementation of an intervention”. Check what are the mediators to produce outcomes (not only primary, but also secondary or unintended outcomes).** |
| --- |
| **Mechanisms available at Malatji et al. 2022. A process evaluation article** |

**Table 3 – Outcome**

| - **Please make note of all (intended or not) outcomes. Please also classify the role and functions outcomes of CHWs using the following Pillars for people-centred care:**   1. Engaging and empowering people and communities  2. Strengthening governance and accountability (Incl. Supervision, training, and support)  3. Reorienting the model of care  4. Coordinating services within and across sectors  5. Enabling environment |
| --- |
| **Engaging and empowering people and communities**   - The mentor's support and supervision of CHWs helped build their confidence and competence in engaging with household members, empowering them to effectively deliver healthcare services. - By integrating the CHW program into the healthcare facility, the intervention empowered CHWs by increasing their **visibility and recognition as contributing members of the healthcare system.**   **Strengthening governance and accountability (Incl. Supervision, training, and support)**   - The intervention supported the alignment of CHW initiatives with broader national health workforce policies, enhancing their effectiveness and impact within the community. - The intervention design workshop involved district and provincial stakeholders, promoting collaboration and shared accountability in the implementation of the intervention. - The research team conducted feedback meetings with CHWs and facility staff participants, allowing them to comment on study findings and refine data collection methods, promoting transparency and accountability in the research process. - The intervention aimed to strengthen relationships between the CHW team, their supervisor, clinic staff, and community organizations, fostering a collaborative and accountable approach to healthcare delivery. - Overall, the intervention of a roving nurse mentor in the CHW program strengthened governance and accountability through supportive supervision, training, stakeholder engagement, and feedback mechanisms.   **Reorienting the model of care**   - The intervention introduced a roving nurse mentor who worked with Community Health Worker (CHW) teams led by junior nurses, aiming to strengthen the capacity of the teams and improve the quality of care provided. - The nurse mentor focused on improving the clinical knowledge and skills of the CHW supervisors and CHWs, serving as a role model for supportive supervision. - The mentor aimed to strengthen relationships between the CHW team, their supervisor, and clinic staff, promoting collaboration and effective communication within the healthcare system. - The intervention also aimed to strengthen relationships with community organizations and political structures, recognizing the importance of community engagement in the model of care. - By integrating the CHW program into the healthcare facility and aligning it with national health workforce policies, the intervention reoriented the model of care to ensure the effective utilization of CHWs and their integration into the broader healthcare system.   **Coordinating services within and across sectors**  The intervention led to improved relationships between CHWs and other healthcare staff, addressing conflicts and fostering a sense of recognition and inclusion within the health team.  The nurse mentor worked with CHW teams led by junior nurses, providing supportive supervision and guidance, which facilitated coordination of services within the CHW teams.  By integrating the CHW program into the healthcare system and aligning it with national health workforce policies, the intervention promoted coordination of services within the healthcare sector.  The intervention design workshop involved district and provincial stakeholders, fostering collaboration and shared accountability, which could contribute to better coordination of services across sectors.  Overall, the intervention aimed to enhance coordination of services within the healthcare sector through improved relationships, integration of the CHW program, and stakeholder engagement. It also aimed to promote coordination of services across sectors by strengthening relationships with community organizations and political structures.  **Enabling environment**  The mentor's role as a point of authority within the CHW teams championed the role of CHWs and addressed conflicts, contributing to their empowerment and improved performance.  The nurse mentor facilitated engagement and empowerment of CHWs by providing training sessions and accompanying them on household visits, allowing them to practice and enhance their skills.  Overall, the intervention created an enabling environment by providing training, supportive supervision, fostering collaboration, and aligning the CHW program with national policies. |

**Table 4: If, then statements depicting the CMOs.**

| Please detail the CMOs for the study you reviewed using the If (Context), Then (Mechanism) statements. |
| --- |
| **If, Then statements**   - If CHWs are accessible, friendly, and have established positive relationships with the community, then the community will approach or engage with CHWs regarding their needs for services, fostering trust and rapport, as a result, there will be effective healthcare delivery of care that is tailored to community needs. - If CHWs receive support and guidance from nursing mentors, then they will feel more confident in their roles and responsibilities, alleviating any fears or concerns they may have, ultimately enhancing their performance as CHWs. - If CHWs receive both training to enhance their skills and knowledge and supportive supervision from nursing mentors, then CHWs will develop confidence, competence and feel empowered to perform their duties, leading to high quality care and effective delivery of care. - If CHWs are directly employed by the Ministry of health, then CHWs will feel valued and recognized as contributing members of the healthcare system, fostering a sense of belonging and support from the system, this will result in improved job satisfaction, motivation, and retention, ultimately benefiting both the healthcare system and the communities they serve. - If CHWs are compensated fairly for their work, then they will feel valued and motivated to carry out their responsibilities effectively, leading to improved performance in delivering healthcare services. |

**Test for relevance (Pearson et al. 2012; 2015; Brennan et al. 2017)**

| **Conceptually Rich** | **Thicker description’ but not ‘conceptually rich’** | **Conceptually Thin** |
| --- | --- | --- |
| Unambiguous theoretical concepts are described in sufficient depth. | Description of programme theory or sufficient information to enable it to ‘surface’. | Insufficient information to enable the programme theory to surface. |
| Relationships between, amongst concepts are clearly articulated. | Consideration of the context in which the programme takes place. | Limited or no consideration of the context in which the programme took place. |
| Concepts are sufficiently developed, defined to enable understanding without the reader needing to have first-hand experience of an area of practice. | Discussion of the differences between the design and orientation of programme theory (what was intended) and implementation (what really happened). | Limited or no discussion of the differences between the design and orientation of programme theory (what was intended) and implementation (what really happened). |
| Concepts are grounded strongly in a cited body of literature. | Recognition and discussion of the strengths/weaknesses of the implemented programme. | Limited or no discussion of the strengths/ weaknesses of the implemented programme. |
| Concepts are parsimonious (i.e., provide the simplest, but not over-simplified, explanation) | Some attempt to explain anomalous results and findings with reference to context and data. | No attempts to explain anomalous results and findings with reference to context and data. |
|  | Description of the factor affecting implementation. | Limited or no description of the factors affecting implementation. |

**Test for relevance (Pearson et al. 2012; 2015; Brennan et al. 2017)**

| **Conceptually Rich** | **Thicker description’ but not ‘conceptually rich’** | **Conceptually Thin** |
| --- | --- | --- |
| √ | √ |  |

**Test for rigour (Ohly et al. 2017)**

|  | **Yes** | **Fairly** | **No** |
| --- | --- | --- | --- |
| The study methods are clearly reported. | √ |  |  |
| The study methods are appropriate to answer RQ. | √ |  |  |
| The sample characteristics enable generalizability. |  |  | √ |
| Raw data supports the study findings (conclusions). | √ |  |  |
| Limitations of the study are acknowledged and clearly reported. | √ |  |  |

**References**

1. Wong G, Westhorp G, Manzano A, Greenhalgh J, Jagosh J, Greenhalgh T. (2016). RAMESES II

reporting standards for realist evaluations. *BMC Medicine*, 14(1), 1-18.

2. Wong G, Greenhalgh T, Westhorp G, Pawson R. (2012). Realist methods in medical education

research: what are they and what can they contribute? *Medical Education*, 46(1), 89-96.

3. Macfarlane F, Greenhalgh T, Humphrey C, Hughes J, Butler C, Pawson R. (2011). A new workforce

in the making?: A case study of strategic human resource management in a whole-system change

effort in healthcare. *Journal of Health Organization and Management*, 25(1), 55-72.

4. Lacouture A, Breton E, Guichard A, Ridde V. (2015). The concept of mechanism from a realist

approach: a scoping review to facilitate its operationalization in public health program evaluation.

*Implementation Science*, 10(1), 1-10.

5. Pearson M, Chilton R, Woods HB, Wyatt K, Ford T, Abraham C, et al. Implementing health promotion in schools: protocol for a realist systematic review of research and experience in the United Kingdom (UK). Syst Rev. 2012 Oct 20;1(1):48.

6. Pearson M, Brand SL, Quinn C, Shaw J, Maguire M, Michie S, et al. Using realist review to inform intervention development: methodological illustration and conceptual platform for collaborative care in offender mental health. Implementation Sci. 2015 Sep 28;10(1):134.

7. Brennan N, Bryce M, Pearson M, Wong G, Cooper C, Archer J. Towards an understanding of how appraisal of doctors produces its effects: a realist review. Medical Education. 2017;51(10):1002–13.

8. Ohly H, Crossland N, Dykes F, Lowe N, Hall-Moran V. A realist review to explore how low-income pregnant women use food vouchers from the UK’s Healthy Start programme. BMJ Open. 2017 Apr 21;7(4):e013731.

**A realist evaluation of the role and functions of community health workers in service of a people-centred community health system.**

**Investigator CMOc Data Extraction Sheet Data Extraction**

The purpose of this document is to extract data from the identified studies in order to formulate one or more explanatory theories that explain Context-Mechanism-Outcome Configurations (CMOC) and to gain insight into how these CMOCs contribute to optimizing the role and functions of community health workers within a people-centred community health system.

In realist methodologies, knowledge accumulation centres on our comprehension of how mechanisms behave in various contexts and the reasons behind how and why this interplay of context and mechanism leads to different outcomes (1). Please fill in the details below for the study you will be assessing. In the subsequent three tables, locate and specify the Context, Mechanism, and Outcomes (CMO) of the intervention discussed in the study you've examined. Definitions and illustrations of CMO are provided within the tables.

**Kindly fill out the information below for the study you will be reviewing.**

| **Reviewer Name** | Usangiphile Buthelezi |
| --- | --- |
| **Reference** | Jenson A, Roter DL, Mkocha H, Munoz B, West S. Patient-centered communication of community treatment assistants in Tanzania predicts coverage of future mass drug administration for trachoma. Patient Educ Couns. 2018 Jun;101(6):1075–81. |
| **Country** | Tanzania |
| **Setting and population** | - The study was conducted in Tanzania as part of the Partnership for Rapid Elimination of Trachoma (PRET) program. - The study included 32 villages that underwent mass drug administration (MDA) for trachoma using community treatment assistants (CTAs) supervised by the Tanzanian study team in 2011. - The villages were selected based on geographic diversity and higher rates of villager non-participation in previous research. - The CTAs were selected by the village leadership and the Tanzanian study team and were paid for their work. - The study focused on the interactions between the CTAs and the villagers during MDA. - The study population included adults (over 15 years of age) who received MDA in 2011 and their return for MDA in 2012 was the outcome of interest. - Both male and female CTAs were included in the study. |
| **Aim** | The aim of the study was to investigate the relationship between patient-centered communication by community treatment assistants (CTAs) and the coverage of mass drug administration (MDA) for trachoma in Tanzania. |
| **Objectives** | - To assess whether female CTAs had more patient-centered communication compared to male CTAs. - To determine if patient-centered communication by CTAs was associated with higher rates of return for MDA. - To explore the impact of CTA gender on communication patterns during MDA administration. - To provide insights into the importance of patient-centered communication and its potential role in improving participation in public health efforts, particularly when lay health workers are involved. |
| **Methodology** | - The study was conducted as part of the Partnership for Rapid Elimination of Trachoma (PRET) program in Tanzania. - The study included 32 villages that underwent mass drug administration (MDA) for trachoma using community treatment assistants (CTAs) supervised by the Tanzanian study team in 2011. - CTAs were selected by the village leadership and the Tanzanian study team and received training in best practices for providing and recording MDA. - Audio recordings of CTAvillager interactions during MDA were collected using digital voice recorders. - The audio recordings were analyzed using an adaptation of the Roter Interaction Analysis System to assess the content of the conversations between CTAs and villagers. - Patient-centered communication was measured using a patient-centeredness ratio, which was the ratio of facilitation and rapport building (patient-centered talk) to procedural statements, information giving, and data gathering (provider-centered talk). - Statistical analysis, including multiple linear regression, was conducted to examine the association between CTA gender, patient-centered communication, and the proportion of adults returning for MDA in the following year. - The analysis accounted for clustering by village and distribution site and controlled for other CTA demographics. |
| **Intervention description** | - The study focused on the communication style of community treatment assistants (CTAs) during mass drug administration (MDA) for trachoma in Tanzania. - The intervention involved training CTAs in best practices for providing and recording MDA. - The CTAs were supervised by the Tanzanian study team and received a daily payment for their work. - The CTAs distributed medication and information at multiple drug distribution sites within each village. - The audio recordings of CTAvillager interactions during MDA were analyzed to assess the content of the conversations and determine the level of patient-centered communication. - Patient-centered communication was measured using a patient-centeredness ratio, which compared facilitation and rapport building to procedural statements and information giving. - The study aimed to determine if patient-centered communication by CTAs was associated with higher rates of return for MDA. |
| **Intervention outcomes** | - The study found that sites with female community treatment assistants (CTAs) had significantly higher patient-centeredness ratios compared to sites with male CTAs. - Sites with more patient-centered interactions had a higher proportion of patients returning for mass drug administration (MDA). - Patient-centered communication by CTAs was associated with higher rates of return for MDA. - The study highlighted the importance of rapport building and patient-centeredness by CTAs on coverage rates in future MDA. - The findings suggest that patient-centered communication skills should be included in the training of both male and female CTAs to benefit all patients. - The study acknowledged the limitations of outcome analysis due to privacy concerns and the inability to decipher individual interactions from the recordings. - The study emphasized the need to go beyond basic task functions and include patient-centered communication in CTA training. - The study demonstrated the significance of patient-centered communication in the success of public health programs involving lay health workers. |
| **Challenges and limitations** | - The study faced limitations in analyzing individual interactions between community treatment assistants (CTAs) and villagers due to privacy and logistic concerns. This led to the aggregation of outcomes at the site level, making it difficult to determine the impact of specific CTAs on return rates. - The low number of sites providing return data and the uneven distribution of all-female and all-male CTA sites impacted the analysis of the initial hypothesis regarding CTA gender and return rates. - The study acknowledged the potential for unmeasured performance bias as CTAs were aware that their interactions were being recorded. However, previous studies have shown minimal effects of performance bias in response to tape recording. - The study focused specifically on CTAs in the context of trachoma MDA in Tanzania, limiting the generalizability of the findings to other public health programs and settings. - The study did not explore other potential factors influencing MDA coverage, such as distance to the distribution site, adverse side effects, or lack of understanding of the value of treatment. - Overall, while the study provides valuable insights into the importance of patient-centered communication by CTAs, there are limitations in terms of individual interaction analysis, generalizability, and the consideration of other factors affecting MDA coverage. |

**Table 1 – Context**

| **Including (but not limited to) beliefs, social and cultural norms, regulations and economic factors (2,3). For example:**   - Individual capabilities (i.e. values, roles, knowledge, purpose). - Interpersonal relationships supporting the interventions (i.e. communication, collaboration, network, influences). - Institutional settings (i.e. informal rules, organisational culture, leadership, policies, resource allocation, local priorities). - Infra-structural system (i.e. political support). - Country and rurality (i.e. small or large/rural vs regional vs remote). - Socio-demographic characteristics: - Funding context or source (i.e. free, personalised, group vs. individual, government funded). |
| --- |
| **Definition of patient-centered in this context**: refers to a communication approach in healthcare where the focus is on the patient's needs, preferences, and values. It involves actively listening to the patient, showing empathy, and involving them in decision-making regarding their care.   - The study focused on 32 villages that underwent mass drug administration (MDA) for trachoma prevention using community treatment assistants (CTAs) supervised by the Tanzanian study team. - CTAs were selected by the village leadership and the Tanzanian study team and received training in best practices for providing and recording MDA . - Within each village, MDA occurred at multiple drug distribution sites, with 2 to 3 CTAs distributing medication and information at each site . - The selected villages were chosen based on geographic diversity and higher rates of villager non-participation in previous research, aiming to maximize the potential observed impact of communication on return rates.   **Not much information is given in terms of context.** |

**Table 2 – Mechanisms**

| - **“An element of reasoning and/or reactions of an individual or collective agents(s) in regard of resources available in a given context to bring about changes through the implementation of an intervention” (4). Check what are the mediators to produce outcomes (not only primary, but also secondary or unintended outcomes).** |
| --- |
| There were no other specific mechanisms listed in this source except that:   - Patient-centered communication, including facilitation and rapport building, positively predicted return rates for MDA. - CTAs who engaged in patient-centered communication, including facilitation and rapport building, had a higher proportion of patients returning for MDA. |

**Table 3 – Outcomes**

| - **Please make note of all (intended or not) outcomes. Please also classify the role and functions outcomes of CHWs using the following Pillars for people-centred care:**   1. Engaging and empowering people and communities  2. Strengthening governance and accountability (Incl. Supervision, training, and support)  3. Reorienting the model of care  4. Coordinating services within and across sectors  5. Enabling environment |
| --- |
| - The study found that sites with female community treatment assistants (CTAs) had significantly higher patient-centeredness ratios compared to sites with male CTAs. - Sites with more patient-centered interactions had a higher proportion of patients returning for mass drug administration (MDA). - Patient-centered communication by CTAs was associated with higher rates of return for MDA. - The study highlighted the importance of rapport building and patient-centeredness by CTAs on coverage rates in future MDA. - The findings suggest that patient-centered communication skills should be included in the training of both male and female CTAs to benefit all patients. - The study acknowledged the limitations of outcome analysis due to privacy concerns and the inability to decipher individual interactions from the recordings. - The study emphasized the need to go beyond basic task functions and include patient-centered communication in CTA training. - The study demonstrated the significance of patient-centered communication in the success of public health programs involving lay health workers. |

**Table 4 – If, then statements depicting the CMOs.**

| **Please detail the CMOs for the study you reviewed using the If (Context), Then (Mechanism) statements.** |
| --- |
| **If, Then statements**   - If community treatment assistants engaged in patient-centred communication (understanding patients' needs, preferences, and concerns), then patients will feel heard and involved in their care decisions, fostering rapport and patient-centeredness. As a result, patients will adhere to treatment plans and there will be improved treatment outcomes. |

**Test for relevance (Pearson et al. 2012; 2015; Brennan et al. 2017)**

| **Conceptually Rich** | **Thicker description’ but not ‘conceptually rich’** | **Conceptually Thin** |
| --- | --- | --- |
| Unambiguous theoretical concepts are described in sufficient depth. | Description of programme theory or sufficient information to enable it to ‘surface’. | Insufficient information to enable the programme theory to surface. |
| Relationships between, amongst concepts are clearly articulated. | Consideration of the context in which the programme takes place. | Limited or no consideration of the context in which the programme took place. |
| Concepts are sufficiently developed, defined to enable understanding without the reader needing to have first-hand experience of an area of practice. | Discussion of the differences between the design and orientation of programme theory (what was intended) and implementation (what really happened). | Limited or no discussion of the differences between the design and orientation of programme theory (what was intended) and implementation (what really happened). |
| Concepts are grounded strongly in a cited body of literature. | Recognition and discussion of the strengths/weaknesses of the implemented programme. | Limited or no discussion of the strengths/ weaknesses of the implemented programme. |
| Concepts are parsimonious (i.e., provide the simplest, but not over-simplified, explanation) | Some attempt to explain anomalous results and findings with reference to context and data. | No attempts to explain anomalous results and findings with reference to context and data. |
|  | Description of the factor affecting implementation. | Limited or no description of the factors affecting implementation. |

**Test for relevance (Pearson et al. 2012; 2015; Brennan et al. 2017)**

| **Conceptually Rich** | **Thicker description’ but not ‘conceptually rich’** | **Conceptually Thin** |
| --- | --- | --- |
|  |  | **X** |

**Test for rigour (Ohly et al. 2017)**

|  | **Yes** | **Fairly** | **No** |
| --- | --- | --- | --- |
| The study methods are clearly reported. |  | X |  |
| The study methods are appropriate to answer RQ. |  | X |  |
| The sample characteristics enable generalizability. |  |  | X |
| Raw data supports the study findings (conclusions). |  | X |  |
| Limitations of the study are acknowledged and clearly reported. |  | X |  |

**References**

1. Wong G, Westhorp G, Manzano A, Greenhalgh J, Jagosh J, Greenhalgh T. (2016). RAMESES II

reporting standards for realist evaluations. *BMC Medicine*, 14(1), 1-18.

2. Wong G, Greenhalgh T, Westhorp G, Pawson R. (2012). Realist methods in medical education

research: what are they and what can they contribute? *Medical Education*, 46(1), 89-96.

3. Macfarlane F, Greenhalgh T, Humphrey C, Hughes J, Butler C, Pawson R. (2011). A new workforce

in the making?: A case study of strategic human resource management in a whole-system change

effort in healthcare. *Journal of Health Organization and Management*, 25(1), 55-72.

4. Lacouture A, Breton E, Guichard A, Ridde V. (2015). The concept of mechanism from a realist

approach: a scoping review to facilitate its operationalization in public health program evaluation.

*Implementation Science*, 10(1), 1-10.

5. Pearson M, Chilton R, Woods HB, Wyatt K, Ford T, Abraham C, et al. Implementing health promotion in schools: protocol for a realist systematic review of research and experience in the United Kingdom (UK). Syst Rev. 2012 Oct 20;1(1):48.

6. Pearson M, Brand SL, Quinn C, Shaw J, Maguire M, Michie S, et al. Using realist review to inform intervention development: methodological illustration and conceptual platform for collaborative care in offender mental health. Implementation Sci. 2015 Sep 28;10(1):134.

7. Brennan N, Bryce M, Pearson M, Wong G, Cooper C, Archer J. Towards an understanding of how appraisal of doctors produces its effects: a realist review. Medical Education. 2017;51(10):1002–13.

8. Ohly H, Crossland N, Dykes F, Lowe N, Hall-Moran V. A realist review to explore how low-income pregnant women use food vouchers from the UK’s Healthy Start programme. BMJ Open. 2017 Apr 21;7(4):e013731.

**A realist evaluation of the role and functions of community health workers in service of a people-centred community health system.**

**Investigator CMOc Data Extraction Sheet Data Extraction**

The purpose of this document is to extract data from the identified studies in order to formulate one or more explanatory theories that explain Context-Mechanism-Outcome Configurations (CMOC) and to gain insight into how these CMOCs contribute to optimizing the role and functions of community health workers within a people-centred community health system.

In realist methodologies, knowledge accumulation centres on our comprehension of how mechanisms behave in various contexts and the reasons behind how and why this interplay of context and mechanism leads to different outcomes (1). Please fill in the details below for the study you will be assessing. In the subsequent three tables, locate and specify the Context, Mechanism, and Outcomes (CMO) of the intervention discussed in the study you've examined. Definitions and illustrations of CMO are provided within the tables.

**Kindly fill out the information below for the study you will be reviewing.**

| **Reviewer Name** | Usangiphile Buthelezi |
| --- | --- |
| **Reference** | Kletter M, Harris B, Connolly E, Namathanga C, Nhlema B, Makungwa H, et al. Mixed method evaluation of a learning from excellence programme for community health workers in Neno, Malawi. BMC Health Serv Res. 2024 Mar 19;24(1):355. |
| **Country** | Malawi |
| **Setting and population** | - The study was conducted in Neno District, Malawi, focusing on Community Health Workers (CHWs) in that area. - The research specifically looked at the impact of the Learning from Excellence (LfE) programme on CHW motivation and work environment in Neno District. - The population included CHWs, key stakeholders involved in the programme, and site supervisors who oversee the CHWs. - The setting of Neno District provided a real-world context for evaluating the feasibility and effectiveness of the LfE programme in a rural community setting. |
| **Aim** | The research aimed to evaluate a program called Learning from Excellence (LfE) for Community Health Workers (CHWs) in Neno, Malawi   - The main goal was to see if the LfE program could improve CHW motivation and work environment |
| **Objectives** | - Objectives included assessing the feasibility, outputs, and impact of the co-designed LfE program. - The study sought to understand how the LfE intervention could positively influence CHWs in Neno District. - Through interviews and data analysis, the research aimed to assess the impact of the LfE intervention on CHW motivation and perceived supervision. |
| **Methodology** | - The study used a mixed method approach, combining qualitative and quantitative data to get a comprehensive view of the impact of the Learning from Excellence (LfE) program on Community Health Workers (CHWs) in Neno District. - Data from LfE forms and in-depth interviews with key stakeholders, site supervisors, and CHWs were collected and analyzed separately due to time constraints. - The researchers then merged the quantitative and qualitative data to create a logic model illustrating how the LfE program could affect CHWs in Neno District. - This methodology allowed for a more detailed understanding of how the LfE intervention influenced CHW motivation and work environment. - By using both types of data collection and analysis, the study could provide a richer and more nuanced explanation of the program's impact on CHWs in the community. |
| **Intervention description** | - The Learning from Excellence (LfE) programme is a tool used to recognize and appreciate acts of excellence among Community Health Workers (CHWs) in Neno, Malawi. - The intervention involves CHWs filling out a form to report on instances of excellence that they observe in their colleagues. - It encourages a positive work environment by highlighting the good work being done by CHWs, boosting morale and motivation. - The LfE forms submitted by CHWs are analyzed using descriptive statistics and memos to capture qualitative information about the reported excellence. - In addition to the written forms, the programme also includes feedback mechanisms to inform CHWs about who has been recognized for excellence and why. - The impact of LfE on CHWs' motivation and work environment is assessed through in-depth interviews with key stakeholders, CHWs, and site supervisors, as well as pre-post intervention questionnaires. - The programme was co-designed between October 2019 and January 2020 to ensure its feasibility and effectiveness in improving CHW motivation and supervision. - Data collected through questionnaires and interviews are used to create a logic model that outlines the outcomes and the mechanisms through which the LfE intervention influences CHW motivation and performance. - Overall, the LfE programme aims to address low staff motivation among CHWs by recognizing and appreciating their excellent work, ultimately enhancing their work environment and job satisfaction. |
| **Intervention outcomes** | - The intervention led to increased motivation and hard work among Community Health Workers (CHWs) through the recognition and appreciation of their excellent work. - Stakeholders welcomed the intervention for its ability to allow appreciation of CHWs for their excellent work, filling a gap in recognizing their efforts. - While the intervention did not show significant improvement in perceived supervision, there were insights into the importance of effective communication and involvement of all stakeholders for better outcomes. - Positive outcomes included the development of peer support and collegial relations among CHWs, leading to an improved working environment and enhanced support for clients. - Collaborations between CHWs were identified through the intervention, indicating improved coordination and support mechanisms within the community health system. |
| **Challenges and limitations** | - The study faced limitations due to the impact of the COVID-19 pandemic on the workflows and workloads of Community Health Workers (CHWs), affecting the implementation of the Learning from Excellence (LfE) programme. - Remote coordination of the LfE implementation activities led to gaps and omissions, potentially influencing CHWs' understanding of LfE and the types of events reported, as well as their expectations regarding rewards. - Standardized implementation and hands-on guidance could have been improved for Site Supervisors and those implementing LfE, which might have enhanced consistency among different sites. - The limited piloting and co-production activities before the full roll-out of the intervention could have been more extensive, but were constrained by time limitations due to delays caused by the pandemic. - While a significant number of CHWs and Site Supervisors were interviewed, there was a limitation in not achieving data saturation, possibly impacting the comprehensiveness of the findings. - Selection of site supervisors to explain differences between sites revealed that discrepancies were not as substantial as initially thought, suggesting that site supervisors might not have been fully aware of their influence on the uptake of LfE, indicating potential limitations in their understanding or involvement. - The study was conducted in a specific rural setting in Neno District, which may limit the generalizability of the findings to other settings with different types of CHWs, such as fully volunteer or more professional CHWs. - Although the findings from Neno District are useful, the limitations imposed by the pandemic and the distinct characteristics of CHWs in the study should be considered when interpreting the applicability of the results to other regions. |

**Table 1 – Context**

| **Including (but not limited to)** **beliefs, social and cultural norms, regulations and economic factors (2,3). For example:**   - Individual capabilities (i.e. values, roles, knowledge, purpose). - Interpersonal relationships supporting the interventions (i.e. communication, collaboration, network, influences). - Institutional settings (i.e. informal rules, organisational culture, leadership, policies, resource allocation, local priorities). - Infra-structural system (i.e. political support). - Country and rurality (i.e. small or large/rural vs regional vs remote). - Socio-demographic characteristics: - Funding context or source (i.e. free, personalised, group vs. individual, government funded). |
| --- |
| - **Setting**:   - The study was conducted in Neno District, Malawi, in collaboration with Partners in Health (PIH) and the local Ministry of Health.   - Neno District is a rural area with limited accessibility, consisting of 14 catchment areas served by two hospitals and 12 primary facilities.   - Despite the healthcare facilities in place, over 60% of the population faces challenges in accessing healthcare services due to distance and geographical barriers. - **Population**:   - The study focused on Community Health Workers (CHWs) in two catchment areas within Neno District.   - Additionally, key stakeholders, Site Supervisors, and CHWs were interviewed to gather a comprehensive understanding of the Learning from Excellence (LfE) programme. - **Training and Education Level:** - CHWs in the study received training related to patient adherence, psychosocial support, and clinical outcomes in diseases like tuberculosis and HIV. - The CHW programme transitioned to a polyvalent model focusing on various public health areas, requiring a certain level of training and education to address different health concerns effectively. - **Available Resources in the Community:** - Neno District faces challenges in healthcare accessibility due to limited tarmac roads and mountainous terrain, hindering the population's ability to reach healthcare facilities. - Despite the healthcare facilities in place, over 60% of the population reports difficulty accessing services, highlighting resource constraints and geographical barriers. - **Funding:** - The study's funding sources were not explicitly mentioned in the provided contexts. However, it is essential to consider that conducting community health interventions like the Learning from Excellence (LfE) programme requires financial resources to support training, implementation, and data collection efforts. - Partnerships with organizations like Partners in Health and the Ministry of Health indicate collaborative funding mechanisms to support the implementation of healthcare programmes in resource-limited settings like Neno District. |

**Table 2 – Mechanisms**

| - **“An element of reasoning and/or reactions of an individual or collective agents(s) in regard of resources available in a given context to bring about changes through the implementation of an intervention” (4). Check what are the mediators to produce outcomes (not only primary, but also secondary or unintended outcomes).** |
| --- |
| - **Recognition and Appreciation:**   - The intervention allowed for the appreciation of excellent work by Community Health Workers (CHWs), leading to increased motivation and hard work.   - CHWs felt valued and appreciated through the recognition of their work, resulting in a boost in morale and confidence. - **Peer Support and Collegial Relations:**   - Positive outcomes were linked to fostering peer support and collegial relations among CHWs, creating a supportive working environment. - **Increased Motivation:**   - While quantitative data did not show significant improvement, qualitative findings suggested that motivation could be enhanced through recognition and careful introduction of the intervention. - **Refinement and Co-design Process:**   - Further refinement of the intervention through a co-design process with CHWs was recommended to enhance accessibility and usefulness. - **Improved Stakeholder Insights:**   - The intervention improved stakeholder insights into CHW work, facilitating collaborations between CHWs to better support clients. |

**Table 3 – Outcomes**

| - **Please make note of all (intended or not) outcomes. Please also classify the role and functions outcomes of CHWs using the following Pillars for people-centred care:**   1. Engaging and empowering people and communities  2. Strengthening governance and accountability (Incl. Supervision, training, and support)  3. Reorienting the model of care  4. Coordinating services within and across sectors  5. Enabling environment |
| --- |
| - The intervention led to increased motivation and hard work among Community Health Workers (CHWs) through the recognition and appreciation of their excellent work. - Stakeholders welcomed the intervention for its ability to allow appreciation of CHWs for their excellent work, filling a gap in recognizing their efforts. - While the intervention did not show significant improvement in perceived supervision, there were insights into the importance of effective communication and involvement of all stakeholders for better outcomes. - Positive outcomes included the development of peer support and collegial relations among CHWs, leading to an improved working environment and enhanced support for clients. - Collaborations between CHWs were identified through the intervention, indicating improved coordination and support mechanisms within the community health system.   **Outcomes according to PCC pillars:**   - **Engagement and Empowerment:**   - The intervention recognized and appreciated the work of CHWs, enhancing motivation and creating a sense of value and purpose among them. - **Governance and Accountability:**   - While no significant impact was found on perceived supervision, there were insights suggesting the need for improved communication and involvement of all stakeholders. - **Model of Care Reorientation:**   - The intervention aimed to improve stakeholder insights into CHW work, fostering collaborations and support mechanisms that could enhance care delivery. - **Service Coordination:**   - Positive outcomes included the development of peer support and collegial relations among CHWs, which contributed to an improved working environment and client support. - **Enabling Environment:**   - By engaging in a co-design process with CHWs, there were recommendations to refine the intervention to ensure it is accessible and beneficial to all participants. |

**Table 4 – If, then statements depicting the CMOs.**

| **Please detail the CMOs for the study you reviewed using the If (Context), Then (Mechanism) statements.** |
| --- |
| **IF** there is peer support and collegial relationships amongst Community Health Workers (CHWs), **THEN** this leads to a supportive environment, fostering morale boost and confidence, **BECAUSE** a supportive environment built on peer relationships and collegial support encourages CHWs to feel valued and confident in their roles, which in turn results in enhanced support for clients.  **IF** Community Health Workers (CHWs) recognize each other's excellence and give feedback, **THEN** this leads to increased motivation and hard work among CHWs, **BECAUSE** the recognition and appreciation of their excellent work boosts their morale and confidence, resulting in better delivery of health services. |

**Test for relevance (Pearson et al. 2012; 2015; Brennan et al. 2017)**

| **Conceptually Rich** | **Thicker description’ but not ‘conceptually rich’** | **Conceptually Thin** |
| --- | --- | --- |
| Unambiguous theoretical concepts are described in sufficient depth. | Description of programme theory or sufficient information to enable it to ‘surface’. | Insufficient information to enable the programme theory to surface. |
| Relationships between, amongst concepts are clearly articulated. | Consideration of the context in which the programme takes place. | Limited or no consideration of the context in which the programme took place. |
| Concepts are sufficiently developed, defined to enable understanding without the reader needing to have first-hand experience of an area of practice. | Discussion of the differences between the design and orientation of programme theory (what was intended) and implementation (what really happened). | Limited or no discussion of the differences between the design and orientation of programme theory (what was intended) and implementation (what really happened). |
| Concepts are grounded strongly in a cited body of literature. | Recognition and discussion of the strengths/weaknesses of the implemented programme. | Limited or no discussion of the strengths/ weaknesses of the implemented programme. |
| Concepts are parsimonious (i.e., provide the simplest, but not over-simplified, explanation) | Some attempt to explain anomalous results and findings with reference to context and data. | No attempts to explain anomalous results and findings with reference to context and data. |
|  | Description of the factor affecting implementation. | Limited or no description of the factors affecting implementation. |

**Test for relevance (Pearson et al. 2012; 2015; Brennan et al. 2017)**

| **Conceptually Rich** | **Thicker description’ but not ‘conceptually rich’** | **Conceptually Thin** |
| --- | --- | --- |
|  | X |  |

**Test for rigour (Ohly et al. 2017)**

|  | **Yes** | **Fairly** | **No** |
| --- | --- | --- | --- |
| The study methods are clearly reported. | X |  |  |
| The study methods are appropriate to answer RQ. | X |  |  |
| The sample characteristics enable generalizability. |  |  | X |
| Raw data supports the study findings (conclusions). | X |  |  |
| Limitations of the study are acknowledged and clearly reported. | X |  |  |

**References**

1. Wong G, Westhorp G, Manzano A, Greenhalgh J, Jagosh J, Greenhalgh T. (2016). RAMESES II

reporting standards for realist evaluations. *BMC Medicine*, 14(1), 1-18.

2. Wong G, Greenhalgh T, Westhorp G, Pawson R. (2012). Realist methods in medical education

research: what are they and what can they contribute? *Medical Education*, 46(1), 89-96.

3. Macfarlane F, Greenhalgh T, Humphrey C, Hughes J, Butler C, Pawson R. (2011). A new workforce

in the making?: A case study of strategic human resource management in a whole-system change

effort in healthcare. *Journal of Health Organization and Management*, 25(1), 55-72.

4. Lacouture A, Breton E, Guichard A, Ridde V. (2015). The concept of mechanism from a realist

approach: a scoping review to facilitate its operationalization in public health program evaluation.

*Implementation Science*, 10(1), 1-10.

5. Pearson M, Chilton R, Woods HB, Wyatt K, Ford T, Abraham C, et al. Implementing health promotion in schools: protocol for a realist systematic review of research and experience in the United Kingdom (UK). Syst Rev. 2012 Oct 20;1(1):48.

6. Pearson M, Brand SL, Quinn C, Shaw J, Maguire M, Michie S, et al. Using realist review to inform intervention development: methodological illustration and conceptual platform for collaborative care in offender mental health. Implementation Sci. 2015 Sep 28;10(1):134.

7. Brennan N, Bryce M, Pearson M, Wong G, Cooper C, Archer J. Towards an understanding of how appraisal of doctors produces its effects: a realist review. Medical Education. 2017;51(10):1002–13.

8. Ohly H, Crossland N, Dykes F, Lowe N, Hall-Moran V. A realist review to explore how low-income pregnant women use food vouchers from the UK’s Healthy Start programme. BMJ Open. 2017 Apr 21;7(4):e013731.

**A realist evaluation of the role and functions of community health workers in service of a people-centred community health system.**

**Investigator CMOc Data Extraction Sheet Data Extraction**

The purpose of this document is to extract data from the identified studies in order to formulate one or more explanatory theories that explain Context-Mechanism-Outcome Configurations (CMOC) and to gain insight into how these CMOCs contribute to optimizing the role and functions of community health workers within a people-centred community health system.

In realist methodologies, knowledge accumulation centres on our comprehension of how mechanisms behave in various contexts and the reasons behind how and why this interplay of context and mechanism leads to different outcomes (1). Please fill in the details below for the study you will be assessing. In the subsequent three tables, locate and specify the Context, Mechanism, and Outcomes (CMO) of the intervention discussed in the study you've examined. Definitions and illustrations of CMO are provided within the tables.

**Kindly fill out the information below for the study you will be reviewing.**

| **Reviewer Name** | Usangiphile Buthelezi |
| --- | --- |
| **Reference** | Klingberg S, van Sluijs EMF, Jong ST, Draper CE. Can public sector community health workers deliver a nurturing care intervention in South Africa? The Amagugu Asakhula feasibility study. Pilot and Feasibility Studies. 2021 Feb 27;7(1):60. |
| **Country** | South Africa |
| **Setting and population** | - The study was conducted in Soweto, a predominantly low-income, urban setting in South Africa. - The population consisted of caregivers of preschool-age children (3-5 years) in Soweto. - Community health workers (CHWs) linked to a public primary health care facility in Soweto delivered the intervention. |
| **Aim** | - The primary aim of the study was to evaluate the feasibility and acceptability of delivering the Amagugu Asakhula intervention by community health workers (CHWs) linked to a public primary health care facility in Soweto, South Africa. |
| **Objectives** | - To assess the delivery of the intervention in a new setting and generate context-specific insights about implementation to support its optimization. - Specific objectives included assessing the feasibility, acceptability, adoption, appropriateness, implementation, fidelity, and context of delivering the intervention through CHWs in Soweto. |
| **Methodology** | - The study utilized a qualitative design to assess the feasibility, acceptability, adoption, appropriateness, implementation, fidelity, and context of delivering the Amagugu Asakhula intervention through community health workers (CHWs) linked to a public sector primary health care facility in Soweto. - CHWs delivered the intervention to caregivers of preschool-age children over a period of 6 weeks. Focus group discussions were held with CHWs and caregivers, and additional data were obtained through observations, study records, and key informant interviews. |
| **Intervention description** | - The Amagugu Asakhula intervention aimed to promote developmentally important dietary and movement behaviors among preschool-age children in South Africa. It was delivered by community health workers (CHWs) linked to a public sector primary health care facility in Soweto. - The intervention consisted of weekly sessions over a six-week period, where CHWs interacted with individual caregivers of preschool-age children. The sessions focused on nurturing interactions and developmentally important health behaviors, such as cognitive development, physical activity, screen time, diet, and sleep. - The intervention was adapted from a previous intervention called Amagugu, which was successful in promoting pediatric HIV disclosure. The Amagugu Asakhula intervention retained key elements of caregiver support, strengthening the caregiver-child relationship, counseling approach, and home-based delivery. However, the HIV messages were replaced with messages related to child development and health. |
| **Intervention outcomes** | - The Amagugu Asakhula intervention was well received by both community health workers (CHWs) and caregivers, and it was considered a good fit with the CHWs' scope of work. - The intervention was found to be acceptable to caregivers of preschool children. - However, the delivery of the intervention through CHWs linked to a public sector primary health care facility in Soweto was not found to be feasible due to contextual challenges such as late payment of salaries influencing CHW performance and willingness to deliver the intervention. - Despite the barriers to successful delivery, the intervention was still delivered, but it was not found to be feasible due to the challenges faced. - There were contextual challenges, such as late payment of salaries, which influenced CHW performance and willingness to deliver the intervention. - CHWs also expressed dissatisfaction with their general working conditions, which made them reluctant to take on new tasks. Despite these barriers, the intervention was still delivered, but it was not found to be feasible due to the challenges faced. |
| **Challenges and limitations** | - Late payment of salaries influenced the performance and willingness of community health workers (CHWs) to deliver the Amagugu Asakhula intervention, making it less feasible. - CHWs expressed dissatisfaction with their general working conditions, which made them reluctant to take on new tasks, further hindering the delivery of the intervention. - Contextual challenges, such as organizational conflicts and challenges within the public health care system, were described as considerable challenges to delivering the intervention. - The delivery of the intervention through CHWs linked to a public sector primary health care facility in Soweto was not found to be feasible due to the aforementioned challenges. - The study did not provide specific quantitative outcomes or results of the intervention, focusing more on the feasibility and acceptability of the delivery process.   Note: The article primarily focuses on the feasibility and acceptability of the intervention delivery, rather than providing specific study limitations and challenges. |

**Table 1 – Context**

| **Including (but not limited to) beliefs, social and cultural norms, regulations and economic factors (2,3). For example:**   - Individual capabilities (i.e. values, roles, knowledge, purpose). - Interpersonal relationships supporting the interventions (i.e. communication, collaboration, network, influences). - Institutional settings (i.e. informal rules, organisational culture, leadership, policies, resource allocation, local priorities). - Infra-structural system (i.e. political support). - Country and rurality (i.e. small or large/rural vs regional vs remote). - Socio-demographic characteristics: - Funding context or source (i.e. free, personalised, group vs. individual, government funded). |
| --- |
| **Country and rurality:**   - The study was conducted in Soweto, a predominantly low-income, urban setting in South Africa, indicating an urban context.   **Socio-demographic characteristics:**   - The study focused on caregivers of preschool-age children in South Africa but did not provide specific socio-demographic characteristics of the participants. |

**Table 2 – Mechanisms**

| - **“An element of reasoning and/or reactions of an individual or collective agents(s) in regard of resources available in a given context to bring about changes through the implementation of an intervention” (4). Check what are the mediators to produce outcomes (not only primary, but also secondary or unintended outcomes).** |
| --- |
| **Positive mechanisms that lead to outcomes:**   - The intervention was well received by both community health workers (CHWs) and caregivers, indicating positive engagement and empowerment of individuals and communities in promoting child health and development. - The intervention aligned with the CHWs' scope of work, suggesting a good fit with their roles and values. - Caregivers expressed positive views about the intervention, valuing its focus on children's well-being and learning.   **Negative mechanisms that lead to outcomes:**   - Contextual challenges, such as late payment of salaries and dissatisfaction with working conditions, influenced CHW performance and willingness to deliver the intervention, potentially hindering its effectiveness. - CHWs expressed reluctance to take on new tasks, possibly due to dissatisfaction with their general working conditions. |

**Table 3 – Outcomes**

| - **Please make note of all (intended or not) outcomes. Please also classify the role and functions outcomes of CHWs using the following Pillars for people-centred care:**   1. Engaging and empowering people and communities  2. Strengthening governance and accountability (Incl. Supervision, training, and support)  3. Reorienting the model of care  4. Coordinating services within and across sectors  5. Enabling environment |
| --- |
| - The Amagugu Asakhula intervention was well received by both community health workers (CHWs) and caregivers, and it was considered a good fit with the CHWs' scope of work. - The intervention was found to be acceptable to caregivers of preschool children. - However, the delivery of the intervention through CHWs linked to a public sector primary health care facility in Soweto was not found to be feasible due to contextual challenges such as late payment of salaries influencing CHW performance and willingness to deliver the intervention. - Despite the barriers to successful delivery, the intervention was still delivered, but it was not found to be feasible due to the challenges faced. - There were contextual challenges, such as late payment of salaries, which influenced CHW performance and willingness to deliver the intervention. - CHWs also expressed dissatisfaction with their general working conditions, which made them reluctant to take on new tasks. Despite these barriers, the intervention was still delivered, but it was not found to be feasible due to the challenges faced.   **Intervention Outcomes by PCC Pillars:**  **Engaging and empowering people and communities:**   - The intervention was well received by both community health workers (CHWs) and caregivers, indicating engagement and empowerment of individuals and communities in promoting child health and development. - Caregivers expressed positive views about the intervention, valuing its focus on children's well-being and learning.   **Strengthening governance and accountability:**   - The study highlighted contextual challenges, such as late payment of salaries and dissatisfaction with working conditions, which influenced CHW performance and willingness to deliver the intervention. - The findings emphasized the need for comprehensive training and strategies to monitor delivery and ensure fidelity of the intervention, indicating the importance of supervision, training, and support for strengthening governance and accountability.   **Reorienting the model of care:**   - The intervention aimed to promote nurturing interactions and developmentally important health behaviors among preschool-age children, aligning with the nurturing care framework. - The study provided insights for optimizing the intervention in the future, suggesting the need for enhanced training and flexibility in evaluations to examine modifications and their impact on outcomes.   **Coordinating services within and across sectors:**   - The study focused on the delivery of the intervention by CHWs linked to a public sector primary health care facility, indicating coordination within the health sector. - However, the study did not specifically address coordination across sectors.   **Enabling environment:**   - The study highlighted contextual challenges, such as late payment of salaries and dissatisfaction with working conditions, which may have hindered the delivery of the intervention, suggesting the need for an enabling environment that supports the well-being and motivation of CHWs. |

**Table 4 – If, then statements depicting the CMOs.**

| **Please detail the CMOs for the study you reviewed using the If (Context), Then (Mechanism) statements.** |
| --- |
| **If, Then statements:**   - If CHWs faces contextual challenges such as late payment of salaries, poor renumeration and dissatisfaction with their working conditions, then CHWs will be demotivated, reluctant to take upon new tasks and to perform their roles and responsibilities, leading to poor performance and delivery of services to the community. |

**Test for relevance (Pearson et al. 2012; 2015; Brennan et al. 2017)**

| **Conceptually Rich** | **Thicker description’ but not ‘conceptually rich’** | **Conceptually Thin** |
| --- | --- | --- |
| Unambiguous theoretical concepts are described in sufficient depth. | Description of programme theory or sufficient information to enable it to ‘surface’. | Insufficient information to enable the programme theory to surface. |
| Relationships between, amongst concepts are clearly articulated. | Consideration of the context in which the programme takes place. | Limited or no consideration of the context in which the programme took place. |
| Concepts are sufficiently developed, defined to enable understanding without the reader needing to have first-hand experience of an area of practice. | Discussion of the differences between the design and orientation of programme theory (what was intended) and implementation (what really happened). | Limited or no discussion of the differences between the design and orientation of programme theory (what was intended) and implementation (what really happened). |
| Concepts are grounded strongly in a cited body of literature. | Recognition and discussion of the strengths/weaknesses of the implemented programme. | Limited or no discussion of the strengths/ weaknesses of the implemented programme. |
| Concepts are parsimonious (i.e., provide the simplest, but not over-simplified, explanation) | Some attempt to explain anomalous results and findings with reference to context and data. | No attempts to explain anomalous results and findings with reference to context and data. |
|  | Description of the factor affecting implementation. | Limited or no description of the factors affecting implementation. |

**Test for relevance (Pearson et al. 2012; 2015; Brennan et al. 2017)**

| **Conceptually Rich** | **Thicker description’ but not ‘conceptually rich’** | **Conceptually Thin** |
| --- | --- | --- |
| X |  |  |

**Test for rigour (Ohly et al. 2017)**

|  | **Yes** | **Fairly** | **No** |
| --- | --- | --- | --- |
| The study methods are clearly reported. | X |  |  |
| The study methods are appropriate to answer RQ. | X |  |  |
| The sample characteristics enable generalizability. |  |  | X |
| Raw data supports the study findings (conclusions). | X |  |  |
| Limitations of the study are acknowledged and clearly reported. | X |  |  |

**References**

1. Wong G, Westhorp G, Manzano A, Greenhalgh J, Jagosh J, Greenhalgh T. (2016). RAMESES II

reporting standards for realist evaluations. *BMC Medicine*, 14(1), 1-18.

2. Wong G, Greenhalgh T, Westhorp G, Pawson R. (2012). Realist methods in medical education

research: what are they and what can they contribute? *Medical Education*, 46(1), 89-96.

3. Macfarlane F, Greenhalgh T, Humphrey C, Hughes J, Butler C, Pawson R. (2011). A new workforce

in the making?: A case study of strategic human resource management in a whole-system change

effort in healthcare. *Journal of Health Organization and Management*, 25(1), 55-72.

4. Lacouture A, Breton E, Guichard A, Ridde V. (2015). The concept of mechanism from a realist

approach: a scoping review to facilitate its operationalization in public health program evaluation.

*Implementation Science*, 10(1), 1-10.

5. Pearson M, Chilton R, Woods HB, Wyatt K, Ford T, Abraham C, et al. Implementing health promotion in schools: protocol for a realist systematic review of research and experience in the United Kingdom (UK). Syst Rev. 2012 Oct 20;1(1):48.

6. Pearson M, Brand SL, Quinn C, Shaw J, Maguire M, Michie S, et al. Using realist review to inform intervention development: methodological illustration and conceptual platform for collaborative care in offender mental health. Implementation Sci. 2015 Sep 28;10(1):134.

7. Brennan N, Bryce M, Pearson M, Wong G, Cooper C, Archer J. Towards an understanding of how appraisal of doctors produces its effects: a realist review. Medical Education. 2017;51(10):1002–13.

8. Ohly H, Crossland N, Dykes F, Lowe N, Hall-Moran V. A realist review to explore how low-income pregnant women use food vouchers from the UK’s Healthy Start programme. BMJ Open. 2017 Apr 21;7(4):e013731.

**A realist evaluation of the role and functions of community health workers in service of a people-centred community health system.**

**Investigator CMOc Data Extraction Sheet Data Extraction**

The purpose of this document is to extract data from the identified studies in order to formulate one or more explanatory theories that explain Context-Mechanism-Outcome Configurations (CMOC) and to gain insight into how these CMOCs contribute to optimizing the role and functions of community health workers within a people-centred community health system.

In realist methodologies, knowledge accumulation centres on our comprehension of how mechanisms behave in various contexts and the reasons behind how and why this interplay of context and mechanism leads to different outcomes (1). Please fill in the details below for the study you will be assessing. In the subsequent three tables, locate and specify the Context, Mechanism, and Outcomes (CMO) of the intervention discussed in the study you've examined. Definitions and illustrations of CMO are provided within the tables.

**Kindly fill out the information below for the study you will be reviewing.**

| **Reviewer Name** | Usangiphile Buthelezi |
| --- | --- |
| **Reference** | le Roux K, le Roux I, Mbewu N, Davis E. The Role of Community Health Workers in the Re-Engineering of Primary Health Care in Rural Eastern Cape. S Afr Fam Pract. 2015 Mar 1;57(2):116–20. |
| **Country** | South Africa |
| **Setting and population** | - The study was conducted in the Oliver Tambo district of the rural Eastern Cape, South Africa. The specific area mentioned is the King Sabata Dalindyebo sub-district, known to be one of the poorest areas in the country. - The population in the catchment area of Zithulele Hospital is approximately 135,000 people. - The area is characterized by high unemployment, dire poverty, and limited access to basic resources such as electricity, running water, and sanitation. - Many young men work in the Rustenburg platinum mines, and young women often leave their children with grandmothers to look for work in cities. - Education levels are low, especially in the older generation, which frequently become the caregivers of young children. - The hospital receives referrals from 13 clinics in the area, which have variable facilities and staffing levels. |
| **Aim** | The aim of the study was to describe the role of community health workers (CHWs) in the re-engineering of primary health care in the rural Eastern Cape of South Africa |
| **Objectives** | - The study aimed to integrate CHWs as generalists into a multi-level health system, focusing on prevention, health promotion, and advocacy for healthy lifestyles and well-being, in addition to clinical services. - The study aimed to assess the functions of CHWs in finding and referring new TB/HIV cases, providing support for TB and HIV treatment adherence, treating various health conditions, and distributing Vitamin A. - The study also aimed to evaluate the effectiveness of the Mentor Mother model in preventing severe malnutrition through early identification and intervention and improving maternal and child health outcomes. - The study aimed to address the challenges of delivering healthcare in a rural setting with limited resources and poor access to health services. - The study aimed to integrate the Philani Mentor Mother program into the district health system, with the support and participation of local chiefs, headmen, clinic staff, nurses, doctors, and administrators. |
| **Methodology** | - The study implemented a model of integrating community health workers (CHWs) trained as generalists into a multi-level health system in the Oliver Tambo district of the rural Eastern Cape, South Africa. - The CHWs, known as Mentor Mothers, were selected based on a "positive deviant model" that utilized the knowledge and coping mechanisms of successful mothers with healthy children within the community. - The Mentor Mothers conducted home visits in their allocated geographical areas, weighing children using a robust electronic scale and identifying underweight children for intervention. - The intervention program focused on maternal, child health, nutrition, and early childhood development, with specific outcome measures such as rehabilitation and improvement rates of malnourished children, HIV/TB testing and treatment adherence, antenatal clinic attendance, and Child Support Grant uptake. - The Mentor Mothers received continuous training, supervision, and support from trained nursing sisters and experienced Mentor Mothers, with a strong commitment to ongoing in-service training. - The effectiveness of the model was assessed through monitoring and accountability of home visits, documenting and assessing each visit and contact based on specific outcome measures. |
| **Intervention description** | - The intervention described in the sources is the integration of community health workers (CHWs), known as Mentor Mothers, into the primary health care system in the rural Eastern Cape of South Africa. - The Mentor Mothers conduct home visits and provide maternal, child health, nutrition, and early childhood development services. - They are selected based on a "positive deviant model" that utilizes the knowledge and coping mechanisms of successful mothers with healthy children within the community. - The Mentor Mothers visit households, weigh children using electronic scales, and identify underweight children for intervention. - They provide support for TB and HIV treatment adherence, treat various health conditions, distribute Vitamin A, and assist in accessing social grants. - The intervention is supported by the Department of Health and requested by senior hospital doctors, with the enthusiastic support of chiefs, headmen, and families in the area. - The Mentor Mothers receive continuous training, supervision, and support from trained nursing sisters and experienced Mentor Mothers. - The effectiveness of the intervention is monitored through documentation and assessment of each home visit and contact based on specific outcome measures. |
| **Intervention outcomes** | - The integration of community health workers (CHWs) trained as generalists into the primary healthcare system in the rural Eastern Cape of South Africa has shown positive outcomes. - CHWs were able to find and refer new TB/HIV cases, provide care for ill children and at-risk pregnant women, rehabilitate malnourished children at home, support TB and HIV treatment adherence, treat common ailments, and distribute essential nutrients. - They also provided follow-up care after clinic and hospital visits, assisted families in applying health information, and helped in accessing social grants. - The Philani Maternal, Child Health and Nutrition Project, which implemented the CHW model, has shown success in improving maternal and child health outcomes. - The intervention program, carried out by Mentor Mothers, has led to the prevention of severe malnutrition through early identification and intervention. After a year of intervention, the malnutrition rates in the Philani intervention areas were half those of the control areas in Cape Town. - The Mentor Mothers in the Philani model have built strong rapport and trust with the families they visit through the positive deviant model, continuous supervision and support, personalized home visits, problem-solving support, and commitment to the families' well-being. - This rapport and trust have facilitated behavior change and improved health outcomes for the families. - CHWs have been effective in identifying and referring new TB/HIV cases, ill children, and at-risk pregnant women. - They have successfully rehabilitated malnourished children at home and supported TB and HIV treatment adherence. - Mentor Mothers have provided treatment for conditions such as diarrhea, worm infestation, and skin problems, as well as distributed Vitamin A. - The intervention has shown improved nutrition and HIV-related outcomes, with lower malnutrition rates in the intervention areas compared to control areas. - The program has successfully identified and provided support for pregnant women with risk factors such as HIV positivity, underage, alcohol use during pregnancy, high blood pressure, or diabetes. - After 3 months in the program, 32.5% of underweight-for-age children were fully rehabilitated, and another 44% showed improvement. - The exclusive breastfeeding rate at 3 months was 26.5%, higher than the national rate of 4-8%. |
| **Challenges and limitations** | - Access to health services in rural South Africa is challenging due to poor infrastructure, lack of transportation, and limited staffing and resources in clinics and hospitals. - CHWs are often poorly selected, trained, and supervised, and may not be respected or well-integrated into the healthcare system. - The Philani Mentor Mother model has addressed some of these challenges by integrating CHWs as part of a multi-level health service system, linking homes with clinics and hospitals. - The rural context of poverty, limited access to basic amenities, and high unemployment further exacerbate health outcomes in the area. - Limited road infrastructure and public transport make accessing healthcare services expensive and difficult, with some patients having to walk long distances to reach a clinic or hospital.   Note: limitations and challenges given in this source are not those relating to the study design, etc. But are contextual. |

**Table 1 – Context**

| **Including (but not limited to) beliefs, social and cultural norms, regulations and economic factors (2,3). For example:**   - Individual capabilities (i.e. values, roles, knowledge, purpose). - Interpersonal relationships supporting the interventions (i.e. communication, collaboration, network, influences). - Institutional settings (i.e. informal rules, organisational culture, leadership, policies, resource allocation, local priorities). - Infra-structural system (i.e. political support). - Country and rurality (i.e. small or large/rural vs regional vs remote). - Socio-demographic characteristics: - Funding context or source (i.e. free, personalised, group vs. individual, government funded). |
| --- |
| - The study was conducted in the Oliver Tambo district of the rural Eastern Cape, South Africa. The specific area mentioned is the King Sabata Dalindyebo sub-district, known to be one of the poorest areas in the country. - The population in the catchment area of Zithulele Hospital is approximately 135,000 people. - The area is characterized by high unemployment, dire poverty, and limited access to basic resources such as electricity, running water, and sanitation. - Many young men work in the Rustenburg platinum mines, and young women often leave their children with grandmothers to look for work in cities. - Education levels are low, especially in the older generation, which frequently become the caregivers of young children.   The hospital receives referrals from 13 clinics in the area, which have variable facilities and staffing levels. |

**Table 2 – Mechanisms**

| - **“An element of reasoning and/or reactions of an individual or collective agents(s) in regard of resources available in a given context to bring about changes through the implementation of an intervention” (4). Check what are the mediators to produce outcomes (not only primary, but also secondary or unintended outcomes).** |
| --- |
| - The Mentor Mothers in the Philani model utilize a positive deviant model, selecting successful mothers with healthy children within the community as role models for struggling mothers, which helps in building rapport and trust. - The Mentor Mothers make home visits, allowing them to establish a personal connection with the families, understand their unique challenges, and provide individualized support, which contributes to building rapport and trust. - The Mentor Mothers provide information, guidance, and support to the families, addressing their health and social challenges, which helps in problem-solving and building trust. - The Mentor Mothers receive continuous supervision and support from trained nursing sisters and experienced Mentor Mothers, which helps in building their confidence and ensuring they provide quality care, leading to trust from the families. - The Mentor Mothers receive continuous in-service training and supervision, which helps in building their confidence and ensuring that they are equipped with the necessary skills to provide quality care. - The training provided to the Mentor Mothers includes knowledge on maternal and child health, nutrition, HIV, TB, mental health, and early childhood development, which enhances their awareness and knowledge to address the health problems of their clients effectively. - The Mentor Mothers follow-up on referrals from the hospital, ensuring continuity of care and showing their commitment to the well-being of the families, which further strengthens trust. - The supportive clinic and hospital leadership, as well as the enthusiastic support from chiefs, headmen, and families in the area, contribute to the recognition and satisfaction of the Mentor Mothers, making them feel respected and appreciated in their role. - The social connection and psychosocial support provided by the Mentor Mothers help families in coping with the challenges of maintaining good maternal and child health in a resource-limited setting. - Overall, these mechanisms contribute to positive intervention outcomes by addressing the holistic needs of the families, promoting behavior change, and improving health outcomes in rural South Africa. |

**Table 3 – Outcomes**

| - **Please make note of all (intended or not) outcomes. Please also classify the role and functions outcomes of CHWs using the following Pillars for people-centred care:**   1. Engaging and empowering people and communities  2. Strengthening governance and accountability (Incl. Supervision, training, and support)  3. Reorienting the model of care  4. Coordinating services within and across sectors  5. Enabling environment |
| --- |
| - The integration of community health workers (CHWs) trained as generalists into the primary healthcare system in the rural Eastern Cape of South Africa has shown positive outcomes. - CHWs were able to find and refer new TB/HIV cases, provide care for ill children and at-risk pregnant women, rehabilitate malnourished children at home, support TB and HIV treatment adherence, treat common ailments, and distribute essential nutrients. - They also provided follow-up care after clinic and hospital visits, assisted families in applying health information, and helped in accessing social grants. - The Philani Maternal, Child Health and Nutrition Project, which implemented the CHW model, has shown success in improving maternal and child health outcomes. - The intervention program, carried out by Mentor Mothers, has led to the prevention of severe malnutrition through early identification and intervention. After a year of intervention, the malnutrition rates in the Philani intervention areas were half those of the control areas in Cape Town. - The Mentor Mothers in the Philani model have built strong rapport and trust with the families they visit through the positive deviant model, continuous supervision and support, personalized home visits, problem-solving support, and commitment to the families' well-being. - This rapport and trust have facilitated behavior change and improved health outcomes for the families.   **Outcomes by PCC pillars:**   1. **Engaging and empowering people and communities:**    - The Philani model engages and empowers the community by utilizing Mentor Mothers who are selected from within the community as role models for struggling mothers, building trust and rapport.    - Mentor Mothers provide personalized home visits, establishing a personal connection with families and addressing their unique challenges, leading to behavior change and improved health outcomes. 2. **Strengthening governance and accountability (Incl. Supervision, training, and support):**    - The Mentor Mothers receive continuous supervision and support from trained nursing sisters and experienced Mentor Mothers, ensuring quality care and building their confidence.    - Regular contact and workshops with local chiefs and headmen help maintain strong relationships and support for the program, contributing to its successful integration into the district health system. 3. **Reorienting the model of care:**    - The Philani model reorients the model of care by integrating CHWs trained as generalists into the primary healthcare system, focusing on prevention, health promotion, and advocacy for healthy lifestyles.    - CHWs provide follow-up care after clinic and hospital visits, support families in applying health information, and assist in accessing social grants, ensuring continuity of care, and addressing the health and social challenges of daily living. 4. **Coordinating services within and across sectors:**    - The Philani model creates a healthcare network between the hospital, clinics, and CHWs, ensuring coordination and collaboration among different levels of care.    - The integration of CHWs into the healthcare system allows for the identification and referral of new TB/HIV cases, ill children, and at-risk pregnant women, ensuring timely and appropriate care. 5. **Enabling environment:**    - The success of the Philani model can be attributed to factors such as building relationships with health teams, having shared goals, and supportive clinic and hospital leadership.    - The enthusiastic support of chiefs, headmen, and the welcoming attitudes of families in the area have made Mentor Mothers feel respected and appreciated, creating an enabling environment for their work. |

**Table 4 – If, then statements depicting the CMOs.**

| **Please detail the CMOs for the study you reviewed using the If (Context), Then (Mechanism) statements.** |
| --- |
| **If, Then statements**   - If CHWs are chosen from community members seen as role models, then it enables them to establish a personal connection with families, understand their unique challenges, and build trust. This, in turn, empowers CHWs to deliver individualized care tailored to each family's specific needs and circumstances. - If CHWs receive ongoing supervision and support from trained professionals (i.e. trained nursing sisters and experienced CHWs), then they gain confidence and the necessary skills to fulfil their roles, leading to the delivery of quality care. - If CHWs follow-up on referrals of community members from the hospital, this will strengthen trust between CHWs and communities due to perceived commitment to the well-being of their families by CHWs, which leads to continuity of care and further community engagement. - If CHWs receive support from the clinic and hospital leadership, as well as from chiefs, headmen, and families in the area, then CHWs will feel motivated, recognised, respected and appreciated in their roles, leading to improved performance, enhanced service delivery, and sustainability. |

**Test for relevance (Pearson et al. 2012; 2015; Brennan et al. 2017)**

| **Conceptually Rich** | **Thicker description’ but not ‘conceptually rich’** | **Conceptually Thin** |
| --- | --- | --- |
| Unambiguous theoretical concepts are described in sufficient depth. | Description of programme theory or sufficient information to enable it to ‘surface’. | Insufficient information to enable the programme theory to surface. |
| Relationships between, amongst concepts are clearly articulated. | Consideration of the context in which the programme takes place. | Limited or no consideration of the context in which the programme took place. |
| Concepts are sufficiently developed, defined to enable understanding without the reader needing to have first-hand experience of an area of practice. | Discussion of the differences between the design and orientation of programme theory (what was intended) and implementation (what really happened). | Limited or no discussion of the differences between the design and orientation of programme theory (what was intended) and implementation (what really happened). |
| Concepts are grounded strongly in a cited body of literature. | Recognition and discussion of the strengths/weaknesses of the implemented programme. | Limited or no discussion of the strengths/ weaknesses of the implemented programme. |
| Concepts are parsimonious (i.e., provide the simplest, but not over-simplified, explanation) | Some attempt to explain anomalous results and findings with reference to context and data. | No attempts to explain anomalous results and findings with reference to context and data. |
|  | Description of the factor affecting implementation. | Limited or no description of the factors affecting implementation. |

**Test for relevance (Pearson et al. 2012; 2015; Brennan et al. 2017)**

| **Conceptually Rich** | **Thicker description’ but not ‘conceptually rich’** | **Conceptually Thin** |
| --- | --- | --- |
|  | X |  |

**Test for rigour (Ohly et al. 2017)**

|  | **Yes** | **Fairly** | **No** |
| --- | --- | --- | --- |
| The study methods are clearly reported. | X |  |  |
| The study methods are appropriate to answer RQ. | X |  |  |
| The sample characteristics enable generalizability. |  |  | X |
| Raw data supports the study findings (conclusions). | X |  |  |
| Limitations of the study are acknowledged and clearly reported. |  |  |  |

**References**

1. Wong G, Westhorp G, Manzano A, Greenhalgh J, Jagosh J, Greenhalgh T. (2016). RAMESES II

reporting standards for realist evaluations. *BMC Medicine*, 14(1), 1-18.

2. Wong G, Greenhalgh T, Westhorp G, Pawson R. (2012). Realist methods in medical education

research: what are they and what can they contribute? *Medical Education*, 46(1), 89-96.

3. Macfarlane F, Greenhalgh T, Humphrey C, Hughes J, Butler C, Pawson R. (2011). A new workforce

in the making?: A case study of strategic human resource management in a whole-system change

effort in healthcare. *Journal of Health Organization and Management*, 25(1), 55-72.

4. Lacouture A, Breton E, Guichard A, Ridde V. (2015). The concept of mechanism from a realist

approach: a scoping review to facilitate its operationalization in public health program evaluation.

*Implementation Science*, 10(1), 1-10.

5. Pearson M, Chilton R, Woods HB, Wyatt K, Ford T, Abraham C, et al. Implementing health promotion in schools: protocol for a realist systematic review of research and experience in the United Kingdom (UK). Syst Rev. 2012 Oct 20;1(1):48.

6. Pearson M, Brand SL, Quinn C, Shaw J, Maguire M, Michie S, et al. Using realist review to inform intervention development: methodological illustration and conceptual platform for collaborative care in offender mental health. Implementation Sci. 2015 Sep 28;10(1):134.

7. Brennan N, Bryce M, Pearson M, Wong G, Cooper C, Archer J. Towards an understanding of how appraisal of doctors produces its effects: a realist review. Medical Education. 2017;51(10):1002–13.

8. Ohly H, Crossland N, Dykes F, Lowe N, Hall-Moran V. A realist review to explore how low-income pregnant women use food vouchers from the UK’s Healthy Start programme. BMJ Open. 2017 Apr 21;7(4):e013731.

**A realist evaluation of the role and functions of community health workers in service of a people-centred community health system.**

**Investigator CMOc Data Extraction Sheet Data Extraction**

The purpose of this document is to extract data from the identified studies in order to formulate one or more explanatory theories that explain Context-Mechanism-Outcome Configurations (CMOC) and to gain insight into how these CMOCs contribute to optimizing the role and functions of community health workers within a people-centred community health system.

In realist methodologies, knowledge accumulation centres on our comprehension of how mechanisms behave in various contexts and the reasons behind how and why this interplay of context and mechanism leads to different outcomes (1). Please fill in the details below for the study you will be assessing. In the subsequent three tables, locate and specify the Context, Mechanism, and Outcomes (CMO) of the intervention discussed in the study you've examined. Definitions and illustrations of CMO are provided within the tables.

**Kindly fill out the information below for the study you will be reviewing.**

| **Reviewer Name** | Usangiphile Buthelezi |
| --- | --- |
| **Reference** | Busza J, Dauya E, Bandason T, Simms V, Chikwari CD, Makamba M, et al. The role of community health workers in improving HIV treatment outcomes in children: lessons learned from the ZENITH trial in Zimbabwe. Health Policy Plan. 2018 Apr 1;33(3):328–34. |
| **Country** | Zimbambwe |
| **Setting and population** | - The study was conducted in Zimbabwe as part of the Zimbabwe study for Enhancing Testing and Improving Treatment of HIV in Children (ZENITH) randomized controlled trial. - The intervention was delivered by community health workers (CHWs) from the Child Protection Society (CPS), who were trained in HIV counseling and home-based care. - The CHWs were selected from the local communities where the intervention was implemented, ensuring that they had relevant experience and a track record of sustained motivation for community work. - The study focused on improving HIV treatment outcomes in children, indicating that the population of interest was children living with HIV |
| **Aim** | - The aim of the study was to assess the experiences of community health workers (CHWs) in delivering an HIV intervention as part of the Zimbabwe study for Enhancing Testing and Improving Treatment of HIV in Children (ZENITH) trial |
| **Objectives** | - To explore CHWs' perceptions of how the intervention's structure and management affected their performance and job satisfaction. - To identify the factors that contribute to the effectiveness of CHW programs, including workload, supervision, training, and supplies. - The study aimed to consider the implications of the findings for the future scale-up and adoption of similar CHW programs in other settings. |
| **Methodology** | - The study utilized a longitudinal, qualitative research design. - Semi-structured interviews were conducted with the 19 community health workers (CHWs) who delivered the intervention at three different time periods: baseline, midline (after 1 year of implementation), and at the end of the intervention. - The interviews explored CHWs' positive and negative experiences, their views on the intervention's structure, materials, procedures, and training. - An independent social scientist conducted the interviews in the CHWs' homes or study clinics, and the interviews were conducted in Shona. - Thematic content analysis was performed using NVIVO 10 to code the transcripts into a priori and data-driven themes. - The analysis compared the themes across the three interview rounds to identify changes in CHWs' perceptions over the intervention period. |
| **Intervention description** | **Note:** The provided sources do not provide a detailed description of the intervention. However, the information provided suggests that the intervention involved home visits by trained CHWs to provide support, counseling, and referrals to caregivers of children living with HIV. The intervention also included intensive supervision, job aids, and training for the CHWs. - Described in the actual trial |
| **Intervention outcomes** | - CHWs expressed strong motivation, commitment, and job satisfaction, and their satisfaction levels increased over time. - Intensive supervision and mentoring were critical to ensuring CHWs' long-term satisfaction. - Provision of job aids, standardized manuals, and refresher training were important for CHWs' performance. - Formalized links between clinics and CHWs were found to be important. - CHWs raised concerns about poor remuneration, reluctance to stop providing support to families after the required number of home visits, and disappointment at the lack of program sustainability after the trial (M-). - The study showed that existing criteria for designing successful CHW programs were useful for maximizing effectiveness, but challenges remained for ensuring long-term sustainability. |
| **Challenges and limitations** | - CHWs expressed concerns about poor remuneration, which can impact their motivation and job satisfaction. - CHWs were reluctant to stop providing support to families after the required number of home visits, indicating a challenge in adhering to program guidelines. - The lack of program sustainability following the completion of the trial was a disappointment for CHWs, highlighting the challenge of ensuring long-term sustainability of CHW-led programs. - CHWs felt that they were reaching only a small proportion of families in need and for insufficient time, indicating limitations in the program's coverage and duration. - The discomfort of CHWs about randomization to a control group, where children known to be living with HIV did not receive CHW support, highlights ethical concerns and challenges in implementing randomized controlled trials in this context. |

**Table 1 – Context**

| **Including (but not limited to) beliefs, social and cultural norms, regulations and economic factors (2,3). For example:**   - Individual capabilities (i.e. values, roles, knowledge, purpose). - Interpersonal relationships supporting the interventions (i.e. communication, collaboration, network, influences). - Institutional settings (i.e. informal rules, organisational culture, leadership, policies, resource allocation, local priorities). - Infra-structural system (i.e. political support). - Country and rurality (i.e. small or large/rural vs regional vs remote). - Socio-demographic characteristics: - Funding context or source (i.e. free, personalised, group vs. individual, government funded). |
| --- |
|  |

**Table 2 – Mechanisms**

| - **“An element of reasoning and/or reactions of an individual or collective agents(s) in regard of resources available in a given context to bring about changes through the implementation of an intervention” (4). Check what are the mediators to produce outcomes (not only primary, but also secondary or unintended outcomes).** |
| --- |
| - CHWs expressed strong motivation, commitment, and job satisfaction, which contributed to their effectiveness in delivering the intervention. - Intensive supervision and mentoring were critical in ensuring CHWs' long-term satisfaction and improving their performance. - Provision of job aids, standardized manuals, and refresher training helped CHWs in delivering the intervention effectively. - Formalized links between clinics and CHWs were important for the success of the program. - Selection of CHWs who understood the community and were trusted by the community played a crucial role in the success of the program. - The use of CHWs to deliver HIV care and support programs was effective in improving treatment outcomes for children. - The availability of regular refresher courses and continuous learning opportunities were important for maintaining knowledge and skills. - The sustainability and scale-up of the program were important for its long-term success.   **Negative Mechanisms in the Study:**   - CHWs expressed concerns about poor remuneration, which can impact their motivation and job satisfaction. - CHWs were reluctant to stop providing support to families after the required number of home visits, indicating a challenge in adhering to program guidelines. - The lack of program sustainability following the completion of the trial was a disappointment for CHWs, highlighting the challenge of ensuring long-term sustainability of CHW-led programs. - CHWs felt that they were reaching only a small proportion of families in need and for insufficient time, indicating limitations in the program's coverage and duration. - The discomfort of CHWs about randomization to a control group, where children known to be living with HIV did not receive CHW support, highlights ethical concerns and challenges in implementing randomized controlled trials in this context. |

**Table 3 – Outcomes**

| - **Please make note of all (intended or not) outcomes. Please also classify the role and functions outcomes of CHWs using the following Pillars for people-centred care:**   1. Engaging and empowering people and communities  2. Strengthening governance and accountability (Incl. Supervision, training, and support)  3. Reorienting the model of care  4. Coordinating services within and across sectors  5. Enabling environment |
| --- |

- The intervention aimed to improve HIV treatment outcomes in children, specifically focusing on increasing retention in care and adherence to treatment.
- CHWs expressed strong motivation, commitment, and job satisfaction, indicating positive outcomes in terms of their engagement and dedication to the intervention.
- Levels of satisfaction among CHWs increased over time, suggesting positive experiences and perceptions of the intervention.
- Intensive supervision and mentoring were identified as critical factors in ensuring CHWs' long-term satisfaction, indicating the importance of supportive supervision in achieving positive outcomes.
- Provision of job aids, standardized manuals, and refresher training were also considered important for successful implementation, highlighting the role of training and resources in achieving positive outcomes.
- The intervention focused on overcoming barriers to attending clinical appointments, establishing reminders for routine drug-taking, and building an enabling environment for the child's care, indicating a comprehensive approach to improving treatment outcomes.

**Intervention outcomes according to PCC:**

**Engaging and empowering people and communities:**

- The intervention relied on community health workers (CHWs) to deliver HIV care and support, which empowered the community to take an active role in the treatment of children living with HIV. CHWs expressed strong motivation, commitment, and job satisfaction, indicating positive outcomes in terms of their engagement and dedication to the intervention.

**Strengthening governance and accountability:**

- Intensive supervision and mentoring were identified as critical factors in ensuring CHWs' long-term satisfaction, highlighting the importance of supportive supervision in achieving positive outcomes. Provision of job aids, standardized manuals, and refresher training were also considered important for successful implementation, indicating the role of training and support in strengthening governance and accountability.

**Reorienting the model of care:**

- The intervention focused on overcoming barriers to attending clinical appointments, establishing reminders for routine drug-taking, and building an enabling environment for the child's care. This comprehensive approach aimed to reorient the model of care towards improving treatment outcomes in children.

**Coordinating services within and across sectors:**

- The intervention emphasized the importance of formalized links between clinics and CHWs, ensuring coordination and collaboration between different sectors involved in HIV care.

**Enabling environment:**

- The intervention provided an enabling work environment for CHWs, including reasonable workload, supportive supervision, and adequate training and supplies. This contributed to the positive outcomes observed in terms of CHWs' motivation, commitment, and job satisfaction.

**Table 4 – If, then statements depicting the CMOs.**

**Please detail the CMOs for the study you reviewed using the If (Context), Then (Mechanism) statements.**

**If, Then statements:**

- If CHWs receive ongoing intensive training, supervision and mentoring (equipping CHWs with the necessary knowledge and skills), then CHWs will feel confident and competent to do their tasks effectively, leading to increased job satisfaction over time.
- If there are formalized links between CHWs and clinics, CHWs will feel valued and recognized, fostering motivation and job satisfaction, which leads to retention, Integration into the formal health system and increased effectiveness in delivering healthcare services to the community.
- If CHWs' have an understanding of the community they serve or understand the local context, then this familiarity builds a sense of trust among community members as they perceive CHWs as individuals who understand and respect their local customs and challenges, leading to tailored services that meet specific needs of the community effectively.
- If CHWs have personal experiences related to the condition of the patient, either through their own lives or within their communities, then CHWs can empathize; and this shared experience can create a deeper connection with community members, fostering trust and acceptability of the CHWs as credible sources of support and information, As a result, community members will be more to engage with CHWs, follow their advice, and adhere to treatment plans.

**Test for relevance (Pearson et al. 2012; 2015; Brennan et al. 2017)**

| **Conceptually Rich** | **Thicker description’ but not ‘conceptually rich’** | **Conceptually Thin** |
| --- | --- | --- |
| Unambiguous theoretical concepts are described in sufficient depth. | Description of programme theory or sufficient information to enable it to ‘surface’. | Insufficient information to enable the programme theory to surface. |
| Relationships between, amongst concepts are clearly articulated. | Consideration of the context in which the programme takes place. | Limited or no consideration of the context in which the programme took place. |
| Concepts are sufficiently developed, defined to enable understanding without the reader needing to have first-hand experience of an area of practice. | Discussion of the differences between the design and orientation of programme theory (what was intended) and implementation (what really happened). | Limited or no discussion of the differences between the design and orientation of programme theory (what was intended) and implementation (what really happened). |
| Concepts are grounded strongly in a cited body of literature. | Recognition and discussion of the strengths/weaknesses of the implemented programme. | Limited or no discussion of the strengths/ weaknesses of the implemented programme. |
| Concepts are parsimonious (i.e., provide the simplest, but not over-simplified, explanation) | Some attempt to explain anomalous results and findings with reference to context and data. | No attempts to explain anomalous results and findings with reference to context and data. |
|  | Description of the factor affecting implementation. | Limited or no description of the factors affecting implementation. |

**Test for relevance (Pearson et al. 2012; 2015; Brennan et al. 2017)**

| **Conceptually Rich** | **Thicker description’ but not ‘conceptually rich’** | **Conceptually Thin** |
| --- | --- | --- |
| X |  |  |

**Test for rigour (Ohly et al. 2017)**

|  | **Yes** | **Fairly** | **No** |
| --- | --- | --- | --- |
| The study methods are clearly reported. | X |  |  |
| The study methods are appropriate to answer RQ. | X |  |  |
| The sample characteristics enable generalizability. |  |  | X |
| Raw data supports the study findings (conclusions). | X |  |  |
| Limitations of the study are acknowledged and clearly reported. | X |  |  |

**References**

1. Wong G, Westhorp G, Manzano A, Greenhalgh J, Jagosh J, Greenhalgh T. (2016). RAMESES II

reporting standards for realist evaluations. *BMC Medicine*, 14(1), 1-18.

2. Wong G, Greenhalgh T, Westhorp G, Pawson R. (2012). Realist methods in medical education

research: what are they and what can they contribute? *Medical Education*, 46(1), 89-96.

3. Macfarlane F, Greenhalgh T, Humphrey C, Hughes J, Butler C, Pawson R. (2011). A new workforce

in the making?: A case study of strategic human resource management in a whole-system change

effort in healthcare. *Journal of Health Organization and Management*, 25(1), 55-72.

4. Lacouture A, Breton E, Guichard A, Ridde V. (2015). The concept of mechanism from a realist

approach: a scoping review to facilitate its operationalization in public health program evaluation.

*Implementation Science*, 10(1), 1-10.

5. Pearson M, Chilton R, Woods HB, Wyatt K, Ford T, Abraham C, et al. Implementing health promotion in schools: protocol for a realist systematic review of research and experience in the United Kingdom (UK). Syst Rev. 2012 Oct 20;1(1):48.

6. Pearson M, Brand SL, Quinn C, Shaw J, Maguire M, Michie S, et al. Using realist review to inform intervention development: methodological illustration and conceptual platform for collaborative care in offender mental health. Implementation Sci. 2015 Sep 28;10(1):134.

7. Brennan N, Bryce M, Pearson M, Wong G, Cooper C, Archer J. Towards an understanding of how appraisal of doctors produces its effects: a realist review. Medical Education. 2017;51(10):1002–13.

8. Ohly H, Crossland N, Dykes F, Lowe N, Hall-Moran V. A realist review to explore how low-income pregnant women use food vouchers from the UK’s Healthy Start programme. BMJ Open. 2017 Apr 21;7(4):e013731.

**A realist evaluation of the role and functions of community health workers in service of a people-centred community health system.**

**Investigator CMOc Data Extraction Sheet Data Extraction**

The purpose of this document is to extract data from the identified studies in order to formulate one or more explanatory theories that explain Context-Mechanism-Outcome Configurations (CMOC) and to gain insight into how these CMOCs contribute to optimizing the role and functions of community health workers within a people-centred community health system.

In realist methodologies, knowledge accumulation centres on our comprehension of how mechanisms behave in various contexts and the reasons behind how and why this interplay of context and mechanism leads to different outcomes (1). Please fill in the details below for the study you will be assessing. In the subsequent three tables, locate and specify the Context, Mechanism, and Outcomes (CMO) of the intervention discussed in the study you've examined. Definitions and illustrations of CMO are provided within the tables.

**Kindly fill out the information below for the study you will be reviewing.**

| **Reviewer Name** | Usangiphile Buthelezi |
| --- | --- |
| **Reference** | Lindsay BR, Mwango L, Toeque MG, Malupande SL, Nkhuwa E, Moonga CN, et al. Peer community health workers improve HIV testing and ART linkage among key populations in Zambia: retrospective observational results from the Z-CHECK project, 2019–2020. Journal of the International AIDS Society. 2022;25(11):e26030. |
| **Country** | Zambia |
| **Setting and population** | - The Z-CHECK project was implemented in Zambia, specifically in five districts in the Southern Province: Mazabuka, Choma, Monze, Kalomo, and Livingstone. - The project targeted key populations (KPs) who face barriers to accessing health services, including female sex workers (FSWs), men who have sex with men (MSM), people who inject drugs (PWID), and people in prisons and enclosed settings. - The interventions were implemented at the community level, utilizing peer community health workers (CHWs) who offered HIV testing in safe spaces and escorted newly diagnosed clients for same-day ART initiation. - KP community leaders and gatekeepers were also engaged to mobilize the KP communities and increase access to health resources. |
| **Aim** | - The aim of the Z-CHECK project was to improve HIV case-finding, linkage to antiretroviral therapy (ART), and treatment adherence among key populations (KPs) in Zambia. |
| **Objectives** | - To address the gaps in HIV control among KPs, including female sex workers (FSWs), men who have sex with men (MSM), people who inject drugs (PWID), and people in prisons and enclosed settings. |
| **Methodology** | - The Z-CHECK project utilized a retrospective observational study design, analyzing routinely collected aggregate data from January 2019 to December 2020. - The project recruited, trained, and deployed peer community health workers (CHWs) for key population (KP) groups, with ongoing mentorship in community engagement. - CHWs offered HIV testing in safe spaces and escorted newly diagnosed clients for same-day ART initiation. - KP community leaders and gatekeepers were engaged for KP mobilization, and healthcare workers (HCWs) were trained on KP services and sensitivity. - Aggregate data from MOH facility testing and linkage registers were abstracted and manually entered into the District Health Information Software 2 data platform for analysis. - Descriptive statistics were calculated on outcomes of interest, and the Mann-Kendall test was used to evaluate trends over time. |
| **Intervention description** | - The Z-CHECK project in Zambia implemented several strategies to improve HIV case-finding, linkage, and treatment adherence among key populations (KPs) such as female sex workers (FSWs), men who have sex with men (MSM), people who inject drugs (PWID), and people in prisons and enclosed settings. - Peer community health workers (CHWs) were recruited, trained, and deployed to KP groups, offering HIV testing in safe spaces and escorting newly diagnosed clients for same-day ART initiation. - The project also engaged KP community leaders and gatekeepers for mobilization and trained healthcare workers (HCWs) on KP services and sensitivity. - Strategies that contributed to high HIV positivity yield and ART linkage included the use of peer KP CHWs, social network testing strategies, and opportunities for same-day ART initiation. - The program also offered psychosocial support, risk assessment, prevention interventions, and counseling on injection-related harm reduction for PWID. - Differentiated service delivery (DSD) models were used to decentralize and integrate HIV care and treatment with other services, leveraging existing healthcare delivery and task-shifting from clinical to non-clinical staff. - The program aimed to reach underserved KPs who are not served by standard healthcare through community-based approaches. |
| **Intervention outcomes** | - The Z-CHECK project provided HIV testing for 9,211 key populations (KPs), with a positivity yield of 24% and 85% of those diagnosed with HIV linked to antiretroviral therapy (ART). - The project achieved high linkage rates for different KP groups, with 95% linkage for men who have sex with men (MSM), 89% for female sex workers (FSWs), 86% for people who inject drugs (PWID), and 65% for people in prisons and enclosed settings. - The use of peer community health workers (CHWs), social network testing strategies, and opportunities for same-day ART initiation contributed to the high positivity yield and linkage rates. - The project also engaged KP community leaders and gatekeepers, resulting in increased access to health resources and diffusion of health messages. - Challenges to program implementation included stigma and discrimination among healthcare workers (HCWs) and attrition of KP CHWs due to high mobility. - Differentiated service delivery (DSD) models and task-shifting from clinical to non-clinical staff were used to decentralize and integrate HIV care and treatment with other services. - The program aimed to reach underserved KPs and improve HIV case-finding, linkage, and treatment adherence at the community level. |
| **Challenges and limitations** | - Stigma and discrimination among healthcare workers (HCWs) posed challenges to program implementation. - Attrition of peer community health workers (CHWs) due to high mobility was a challenge for the program. - The study did not have longitudinal, patient-level data on viral load follow-up, retention in care, and viral suppression, which are critical components of effective treatment and transmission prevention. - The use of a retrospective non-randomized observational study design may introduce bias due to the availability of existing data and the lack of a control group. - The convenience sample used in the study may not represent the general population, leading to selection bias. - The study was conducted in specific districts in Southern Province, which may limit the generalizability of the findings to other regions or countries. |

**Table 1 – Context**

**Including (but not limited to) beliefs, social and cultural norms, regulations and economic factors (2,3). For example:**

- Individual capabilities (i.e. values, roles, knowledge, purpose).
- Interpersonal relationships supporting the interventions (i.e. communication, collaboration, network, influences).
- Institutional settings (i.e. informal rules, organisational culture, leadership, policies, resource allocation, local priorities).
- Infra-structural system (i.e. political support).
- Country and rurality (i.e. small or large/rural vs regional vs remote).
- Socio-demographic characteristics:
- Funding context or source (i.e. free, personalised, group vs. individual, government funded).

- **Health System Organization:** The study was conducted in Zambia, specifically in five districts in the Southern Province. The project, known as Z-CHECK, aimed to improve HIV case-finding, linkage, and treatment adherence among key populations (KPs) in the community. Peer community health workers (CHWs) were recruited, trained, and deployed to provide HIV testing and support services to KPs. Healthcare workers (HCWs) were also trained on KP services and sensitivity.
- **Rurality:** The study was conducted in both urban and rural areas of the five districts in the Southern Province of Zambia.
- **Socio-demographic and Socio-economic Factors:** The study focused on key populations, including female sex workers (FSWs), men who have sex with men (MSM), people who inject drugs (PWID), and people in prisons and enclosed settings. These populations face cultural, social, and legal barriers to accessing healthcare services.
- **Psychosocial Factors:** Stigma and discrimination among healthcare workers (HCWs) were identified as challenges to program implementation.

**Table 2 – Mechanisms**

- **“An element of reasoning and/or reactions of an individual or collective agents(s) in regard of resources available in a given context to bring about changes through the implementation of an intervention” (4). Check what are the mediators to produce outcomes (not only primary, but also secondary or unintended outcomes).**

- Peer community health workers (CHWs) played a crucial role in reaching key populations (KPs), establishing rapport, and building trust within the communities, which contributed to the positive outcomes.
- The use of social network testing strategies allowed for testing within existing social networks, which may have increased motivation and confidence among KPs to get tested for HIV.
- Same-day ART initiation provided immediate access to treatment, reducing barriers and increasing motivation for linkage to care.
- Engagement of KP community leaders and gatekeepers helped in mobilizing the community, increasing awareness, and improving access to health resources.
- Challenges such as stigma and discrimination among healthcare workers (HCWs) may have negatively impacted the outcomes by creating barriers to testing and linkage to care.
- Attrition of KP CHWs due to high mobility may have affected the continuity and effectiveness of the program.

**Table 3 – Outcomes**

- **Please make note of all (intended or not) outcomes. Please also classify the role and functions outcomes of CHWs using the following Pillars for people-centred care:**

1. Engaging and empowering people and communities

2. Strengthening governance and accountability (Incl. Supervision, training, and support)

3. Reorienting the model of care

4. Coordinating services within and across sectors

5. Enabling environment

- The Z-CHECK project provided HIV testing for 9,211 key populations (KPs), with a positivity yield of 24% and 85% of those diagnosed with HIV linked to antiretroviral therapy (ART).
- The project achieved high linkage rates for different KP groups, with 95% linkage for men who have sex with men (MSM), 89% for female sex workers (FSWs), 86% for people who inject drugs (PWID), and 65% for people in prisons and enclosed settings.
- The use of peer community health workers (CHWs), social network testing strategies, and opportunities for same-day ART initiation contributed to the high positivity yield and linkage rates.
- The project also engaged KP community leaders and gatekeepers, resulting in increased access to health resources and diffusion of health messages.
- Challenges to program implementation included stigma and discrimination among healthcare workers (HCWs) and attrition of KP CHWs due to high mobility.
- Differentiated service delivery (DSD) models and task-shifting from clinical to non-clinical staff were used to decentralize and integrate HIV care and treatment with other services.
- The program aimed to reach underserved KPs and improve HIV case-finding, linkage, and treatment adherence at the community level.

**Outcomes by PCC pillars:**

1. **Engaging and empowering people and communities**:
   - Peer community health workers (CHWs) effectively reached key populations (KPs), identified persons living with HIV, and linked them to care, demonstrating the engagement and empowerment of KPs.
   - Engaging KP community gatekeepers resulted in high diffusion of health messages and increased access to health resources.
2. **Strengthening governance and accountability (Incl. Supervision, training, and support)**:
   - Training and mentorship of peer CHWs in community engagement and healthcare workers (HCWs) on KP services and sensitivity contributed to the success of the program.
   - Community liaison officers (CLOs) supervised CHWs to ensure the quality of HIV services and acted as a bridge between CHWs, healthcare facilities, and the community being served.
3. **Reorienting the model of care**:
   - The use of peer CHWs and social network testing effectively delivered HIV prevention and treatment services to KPs.
   - Same-day ART initiation provided immediate access to treatment, reorienting the model of care and reducing barriers to linkage.
4. **Coordinating services within and across sectors**:
   - The Z-CHECK project collaborated with KP civil society organizations, local governments, and healthcare facilities to increase awareness, improve knowledge, and build trust, ensuring coordination of services.
5. **Enabling environment**:
   - The Z-CHECK program aimed to address cultural, social, and legal barriers faced by KPs, creating an enabling environment for HIV case-finding, linkage, and treatment adherence.

**Table 4 – If, then statements depicting the CMOs.**

**Please detail the CMOs for the study you reviewed using the If (Context), Then (Mechanism) statements.**

**If, Then statements**

- If CHWs engage their clients in safe spaces (places preferred by individual clients – this includes location and time), then this creates a supportive and familiar environment for the client, facilitating open communication and trust between CHWs and clients, this results in a better understanding of context, improved disclosure, enhanced participation, and individualized care.

**Test for relevance (Pearson et al. 2012; 2015; Brennan et al. 2017)**

| **Conceptually Rich** | **Thicker description’ but not ‘conceptually rich’** | **Conceptually Thin** |
| --- | --- | --- |
| Unambiguous theoretical concepts are described in sufficient depth. | Description of programme theory or sufficient information to enable it to ‘surface’. | Insufficient information to enable the programme theory to surface. |
| Relationships between, amongst concepts are clearly articulated. | Consideration of the context in which the programme takes place. | Limited or no consideration of the context in which the programme took place. |
| Concepts are sufficiently developed, defined to enable understanding without the reader needing to have first-hand experience of an area of practice. | Discussion of the differences between the design and orientation of programme theory (what was intended) and implementation (what really happened). | Limited or no discussion of the differences between the design and orientation of programme theory (what was intended) and implementation (what really happened). |
| Concepts are grounded strongly in a cited body of literature. | Recognition and discussion of the strengths/weaknesses of the implemented programme. | Limited or no discussion of the strengths/ weaknesses of the implemented programme. |
| Concepts are parsimonious (i.e., provide the simplest, but not over-simplified, explanation) | Some attempt to explain anomalous results and findings with reference to context and data. | No attempts to explain anomalous results and findings with reference to context and data. |
|  | Description of the factor affecting implementation. | Limited or no description of the factors affecting implementation. |

**Test for relevance (Pearson et al. 2012; 2015; Brennan et al. 2017)**

| **Conceptually Rich** | **Thicker description’ but not ‘conceptually rich’** | **Conceptually Thin** |
| --- | --- | --- |
|  | **X** |  |

**Test for rigour (Ohly et al. 2017)**

|  | **Yes** | **Fairly** | **No** |
| --- | --- | --- | --- |
| The study methods are clearly reported. | X |  |  |
| The study methods are appropriate to answer RQ. | X |  |  |
| The sample characteristics enable generalizability. |  |  | X |
| Raw data supports the study findings (conclusions). | X |  |  |
| Limitations of the study are acknowledged and clearly reported. | X |  |  |

**References**

1. Wong G, Westhorp G, Manzano A, Greenhalgh J, Jagosh J, Greenhalgh T. (2016). RAMESES II

reporting standards for realist evaluations. *BMC Medicine*, 14(1), 1-18.

2. Wong G, Greenhalgh T, Westhorp G, Pawson R. (2012). Realist methods in medical education

research: what are they and what can they contribute? *Medical Education*, 46(1), 89-96.

3. Macfarlane F, Greenhalgh T, Humphrey C, Hughes J, Butler C, Pawson R. (2011). A new workforce

in the making?: A case study of strategic human resource management in a whole-system change

effort in healthcare. *Journal of Health Organization and Management*, 25(1), 55-72.

4. Lacouture A, Breton E, Guichard A, Ridde V. (2015). The concept of mechanism from a realist

approach: a scoping review to facilitate its operationalization in public health program evaluation.

*Implementation Science*, 10(1), 1-10.

5. Pearson M, Chilton R, Woods HB, Wyatt K, Ford T, Abraham C, et al. Implementing health promotion in schools: protocol for a realist systematic review of research and experience in the United Kingdom (UK). Syst Rev. 2012 Oct 20;1(1):48.

6. Pearson M, Brand SL, Quinn C, Shaw J, Maguire M, Michie S, et al. Using realist review to inform intervention development: methodological illustration and conceptual platform for collaborative care in offender mental health. Implementation Sci. 2015 Sep 28;10(1):134.

7. Brennan N, Bryce M, Pearson M, Wong G, Cooper C, Archer J. Towards an understanding of how appraisal of doctors produces its effects: a realist review. Medical Education. 2017;51(10):1002–13.

8. Ohly H, Crossland N, Dykes F, Lowe N, Hall-Moran V. A realist review to explore how low-income pregnant women use food vouchers from the UK’s Healthy Start programme. BMJ Open. 2017 Apr 21;7(4):e013731.

**A realist evaluation of the role and functions of community health workers in service of a people-centred community health system.**

**Investigator CMOc Data Extraction Sheet Data Extraction**

The purpose of this document is to extract data from the identified studies in order to formulate one or more explanatory theories that explain Context-Mechanism-Outcome Configurations (CMOC) and to gain insight into how these CMOCs contribute to optimizing the role and functions of community health workers within a people-centred community health system.

In realist methodologies, knowledge accumulation centres on our comprehension of how mechanisms behave in various contexts and the reasons behind how and why this interplay of context and mechanism leads to different outcomes (1). Please fill in the details below for the study you will be assessing. In the subsequent three tables, locate and specify the Context, Mechanism, and Outcomes (CMO) of the intervention discussed in the study you've examined. Definitions and illustrations of CMO are provided within the tables.

**Kindly fill out the information below for the study you will be reviewing.**

| **Reviewer Name** | Usangiphile Buthelezi |
| --- | --- |
| **Reference** | Malatji H, Griffiths F, Goudge J. **Supportive supervision from a roving nurse mentor in a community health worker programme: a process evaluation in South Africa. BMC health services research**. 2022 Mar 10;22(1):323. |
| **Country** | South Africa |
| **Setting and population** | - The study was conducted in a semi-rural area of South Africa, focusing on a community health worker (CHW) program. - Participants included CHWs, their supervisors, clients, facility staff members, and community representatives involved in or knowledgeable about the CHW program in the district. - CHWs worked in pairs for household visits, and observations were made during these visits, CHW meetings, and supervision sessions in both the community and health facility settings. - The CHWs were predominantly women, with very few men involved in the program. The study included approximately 10 participants in each of the four focus groups conducted with CHWs, both before and after the intervention. - Semi-structured interviews were conducted with CHWs, their supervisors, health facility staff members, patients, and community representatives, totaling 168 interviews. The interviews aimed to gather insights on the CHW program, interactions, benefits, challenges, care provision, and community relationships. |
| **Aim** | - The study aimed to evaluate the impact of a roving nurse mentor providing supportive supervision to CHW teams in a semi-rural area of South Africa, focusing on how the intervention was generated and sustained. |
| **Objectives** | - The research sought to assess the effects of the intervention on CHWs, their supervisors, clients, facility staff members, and community representatives, particularly in improving service provision, knowledge and skills development, and operational systems within the CHW program. - Additionally, the research aimed to understand the challenges faced by the CHW program before the intervention, such as insufficient supervision, fragmented programs, and low literacy levels among CHWs, and to identify opportunities for improvement and sustainability |
| **Methodology** | - **Data Collection:**   - Data collection methods included observations, interviews, and focus groups conducted by the first author and a data collection team.   - The data collection process involved training data collectors in community-oriented healthcare, qualitative data collection, and research ethics.   - Audio recordings of interviews and focus groups were transcribed verbatim and checked for accuracy by the research team. - **Data Analysis:**   - Thematic analysis was employed following data extraction, where a coding system was developed to categorize data related to CHWs' priority activities and emerging themes.   - The coding process was conducted using NVIVO 12 software, with the first author completing the coding and engaging in weekly discussions with the research team to ensure data accuracy and completeness.   - Emerging themes were grouped together based on CHWs' priority activities, and the coded data was synthesized to understand the responses to the nurse mentor's activities and the outcomes achieved. - **Study Participants:**   - Participants included CHWs, supervisors, facility staff members, clients, and community representatives involved in or knowledgeable about the CHW program in the district.   - Focus groups were conducted with CHWs, and semi-structured interviews were carried out with various stakeholders to gather insights on the CHW program and its impact. |
| **Intervention description** | - The intervention involved a roving nurse mentor working with CHW teams to provide supportive supervision in a semi-rural area of South Africa. - The nurse mentor, an experienced professional nurse, worked with CHW teams led by junior nurses to improve their integration into the healthcare system and enhance the quality of care provided. - The mentor's role included training CHWs and supervisors, addressing fears of failure, establishing operational systems, and improving service provision. - The intervention design workshop involved sharing findings with stakeholders and planning for the mentor to work with multiple CHW teams to build capacity and strengthen relationships within the healthcare system and community structures. |
| **Intervention outcomes** | - The roving nurse mentor successfully supported and trained CHWs and their supervisors to acquire and practice new skills, addressing their fears of failure and establishing operational systems to enhance the efficiency of CHWs' activities, leading to improved service provision. - Towards the end of the intervention, the direct employment of CHWs by the Department of Health and an increase in their stipend boosted motivation and integration into the local primary care clinic team. - Despite challenges like community focus on housing rather than healthcare and community volatility, the nurse mentor's intervention significantly enhanced CHWs' knowledge and skills development, capacity building of junior supervisors, and overall service provision quality. - The long-term sustainability of the intervention's effects was contingent on the formal employment of CHWs by the Department of Health, emphasizing the importance of institutional support for ongoing success. |
| **Challenges and limitations** |  |

**Table 1 – Context**

**Including (but not limited to) beliefs, social and cultural norms, regulations and economic factors (2,3). For example:**

- Individual capabilities (i.e. values, roles, knowledge, purpose).
- Interpersonal relationships supporting the interventions (i.e. communication, collaboration, network, influences).
- Institutional settings (i.e. informal rules, organisational culture, leadership, policies, resource allocation, local priorities).
- Infra-structural system (i.e. political support).
- Country and rurality (i.e. small or large/rural vs regional vs remote).
- Socio-demographic characteristics:
- Funding context or source (i.e. free, personalised, group vs. individual, government funded).

**Table 2 – Mechanisms**

- **“An element of reasoning and/or reactions of an individual or collective agents(s) in regard of resources available in a given context to bring about changes through the implementation of an intervention” (4). Check what are the mediators to produce outcomes (not only primary, but also secondary or unintended outcomes).**

**CHWs building positive relationships with community members.**

- Community members often approached CHWs informally to seek health-related information, indicating a level of **trust** and **rapport** between the CHWs and the community members.
- CHWs were informed **(engagement)** by community members about individuals in need of their services, showing that the community members felt comfortable reaching out to the CHWs for assistance.
- CHWs had a **positive relationship** with individual community members, which suggests that they were able to establish a **connection and build trust** with the people they served.
- Overall, the CHWs built positive relationships with community members through their **availability, approachability, and the trust** that community members placed in them to seek health-related information and support.

**Roving Nurse intervention**

- The mentor addressed the fears of CHWs and established operational systems to address inefficiencies in the CHWs' activities, further enhancing service delivery.
  - **Providing support and guidance to CHWs**, the nurse mentor helped alleviate their fears and concerns, ensuring they felt more confident in their roles and responsibilities.
  - Working with the CHW teams, the nurse mentor identified the inefficiencies in their activities and developed operational systems to address them. This involved **negotiating with facility staff and establishing necessary systems to improve CHW performance.**
  - The nurse mentor played a critical role in **navigating problems that arose and finding solutions to overcome challenges faced by CHWs**, such as the lack of equipment and limited space.
  - By being a dedicated senior person within the CHW teams, the nurse mentor was able to **work out what systems were required, negotiate with facility staff to establish them**, and navigate problems when they arose. This contributed to the improvement of CHW performance.
  - The change to being employed by the **Department of Health (DoH) meant that facility managers took greater responsibility for the CHW team** and were able to build on the improvements established by the nurse mentor, leading to better integration of the CHW program into the facility.
- The intervention of a roving nurse mentor contributed to the knowledge and skills development of CHWs and enhanced the capacity of junior supervisors.
  - The mentor's experience and expertise contributed to the capacity building of CHWs, as they shared their knowledge and provided training opportunities.
  - By working closely with CHWs, the nurse mentor facilitated **continuous learning and professional growth**, creating a supportive environment for skill development.
  - The mentor's presence and involvement in the CHW teams allowed for ongoing knowledge transfer and the enhancement of practical skills in delivering healthcare services.
  - The nurse mentor also contributed to the development of junior supervisors by enhancing their capacity to effectively supervise and support CHWs.
  - Through **collaboration with junior supervisors**, the nurse mentor helped improve the overall performance and coordination of the CHW program.
- Towards the end of the intervention, the **direct employment of CHWs by the Department of Health and an increase in their stipend** added to their motivation and integration into the local primary care clinic team.
- The intervention also improved the **working relationships between the CHW team and health facility staff**, leading to better integration of CHWs into the healthcare system.

**Table 3 – Outcomes**

- **Please make note of all (intended or not) outcomes. Please also classify the role and functions outcomes of CHWs using the following Pillars for people-centred care:**

1. Engaging and empowering people and communities

2. Strengthening governance and accountability (Incl. Supervision, training, and support)

3. Reorienting the model of care

4. Coordinating services within and across sectors

5. Enabling environment

- The roving nurse mentor successfully supported and trained CHWs and their supervisors to acquire and practice new skills, addressing their fears of failure and establishing operational systems to enhance the efficiency of CHWs' activities, leading to improved service provision.
- Towards the end of the intervention, the direct employment of CHWs by the Department of Health and an increase in their stipend boosted motivation and integration into the local primary care clinic team.
- Despite challenges like community focus on housing rather than healthcare and community volatility, the nurse mentor's intervention significantly enhanced CHWs' knowledge and skills development, capacity building of junior supervisors, and overall service provision quality.
- The long-term sustainability of the intervention's effects was contingent on the formal employment of CHWs by the Department of Health, emphasizing the importance of institutional support for ongoing success.

**Outcomes according to PCC:**

**Engaging and Empowering People and Communities**

- The roving nurse mentor's intervention struggled to engage community leaders and representatives effectively, with limited collaboration with local structures and NGOs, hindering the establishment of a link between the CHW programme and the community.

**Strengthening Governance and Accountability**

- The nurse mentor successfully supported and trained CHWs and supervisors, addressing their fears and enhancing operational systems, resulting in improved service provision, highlighting the importance of supportive supervision in enhancing governance and accountability.

**Reorienting the Model of Care**

- The intervention focused on enhancing the capacity of CHWs and supervisors, improving clinical knowledge, client engagement skills, and relationships with clinic staff, thereby reorienting the model of care towards more comprehensive and effective service provision.

**Coordinating Services within and Across Sectors**

- The mentor's efforts led to improved collaboration between CHWs, facility staff, and community structures, emphasizing the importance of coordination for effective service delivery.

**Enabling Environment**

- Despite challenges like community priorities and volatility, the intervention significantly contributed to knowledge and skills development, capacity building, and service quality, creating a more enabling environment for CHWs and supervisors.

**Table 4 – If, then statements depicting the CMOs.**

**Please detail the CMOs for the study you reviewed using the If (Context), Then (Mechanism) statements.**

**If, Then statements:**

- If CHWs are accessible, friendly, and have established positive relationships with the community, then the community will approach or engage with CHWs regarding their needs for services, fostering trust and rapport, as a result, there will be effective healthcare delivery of care that is tailored to community needs.
- If CHWs receive support and guidance from nursing mentors, then they will feel more confident in their roles and responsibilities, alleviating any fears or concerns they may have, ultimately enhancing their performance as CHWs.
- If CHWs receive both training to enhance their skills and knowledge and supportive supervision from nursing mentors, then CHWs will develop confidence, competence and feel empowered to perform their duties, leading to high quality care and effective delivery of care.
- If CHWs are directly employed by the Ministry of health, then CHWs will feel valued and recognized as contributing members of the healthcare system, fostering a sense of belonging and support from the system, this will result in improved job satisfaction, motivation, and retention, ultimately benefiting both the healthcare system and the communities they serve.
- If CHWs are compensated fairly for their work, then they will feel valued and motivated to carry out their responsibilities effectively, leading to improved performance in delivering healthcare services.

**Test for relevance (Pearson et al. 2012; 2015; Brennan et al. 2017)**

| **Conceptually Rich** | **Thicker description’ but not ‘conceptually rich’** | **Conceptually Thin** |
| --- | --- | --- |
| Unambiguous theoretical concepts are described in sufficient depth. | Description of programme theory or sufficient information to enable it to ‘surface’. | Insufficient information to enable the programme theory to surface. |
| Relationships between, amongst concepts are clearly articulated. | Consideration of the context in which the programme takes place. | Limited or no consideration of the context in which the programme took place. |
| Concepts are sufficiently developed, defined to enable understanding without the reader needing to have first-hand experience of an area of practice. | Discussion of the differences between the design and orientation of programme theory (what was intended) and implementation (what really happened). | Limited or no discussion of the differences between the design and orientation of programme theory (what was intended) and implementation (what really happened). |
| Concepts are grounded strongly in a cited body of literature. | Recognition and discussion of the strengths/weaknesses of the implemented programme. | Limited or no discussion of the strengths/ weaknesses of the implemented programme. |
| Concepts are parsimonious (i.e., provide the simplest, but not over-simplified, explanation) | Some attempt to explain anomalous results and findings with reference to context and data. | No attempts to explain anomalous results and findings with reference to context and data. |
|  | Description of the factor affecting implementation. | Limited or no description of the factors affecting implementation. |

**Test for relevance (Pearson et al. 2012; 2015; Brennan et al. 2017)**

| **Conceptually Rich** | **Thicker description’ but not ‘conceptually rich’** | **Conceptually Thin** |
| --- | --- | --- |
|  | X |  |

**Test for rigour (Ohly et al. 2017)**

|  | **Yes** | **Fairly** | **No** |
| --- | --- | --- | --- |
| The study methods are clearly reported. | X |  |  |
| The study methods are appropriate to answer RQ. | X |  |  |
| The sample characteristics enable generalizability. |  |  | X |
| Raw data supports the study findings (conclusions). | X |  |  |
| Limitations of the study are acknowledged and clearly reported. | X |  |  |

**References**

1. Wong G, Westhorp G, Manzano A, Greenhalgh J, Jagosh J, Greenhalgh T. (2016). RAMESES II

reporting standards for realist evaluations. *BMC Medicine*, 14(1), 1-18.

2. Wong G, Greenhalgh T, Westhorp G, Pawson R. (2012). Realist methods in medical education

research: what are they and what can they contribute? *Medical Education*, 46(1), 89-96.

3. Macfarlane F, Greenhalgh T, Humphrey C, Hughes J, Butler C, Pawson R. (2011). A new workforce

in the making?: A case study of strategic human resource management in a whole-system change

effort in healthcare. *Journal of Health Organization and Management*, 25(1), 55-72.

4. Lacouture A, Breton E, Guichard A, Ridde V. (2015). The concept of mechanism from a realist

approach: a scoping review to facilitate its operationalization in public health program evaluation.

*Implementation Science*, 10(1), 1-10.

5. Pearson M, Chilton R, Woods HB, Wyatt K, Ford T, Abraham C, et al. Implementing health promotion in schools: protocol for a realist systematic review of research and experience in the United Kingdom (UK). Syst Rev. 2012 Oct 20;1(1):48.

6. Pearson M, Brand SL, Quinn C, Shaw J, Maguire M, Michie S, et al. Using realist review to inform intervention development: methodological illustration and conceptual platform for collaborative care in offender mental health. Implementation Sci. 2015 Sep 28;10(1):134.

7. Brennan N, Bryce M, Pearson M, Wong G, Cooper C, Archer J. Towards an understanding of how appraisal of doctors produces its effects: a realist review. Medical Education. 2017;51(10):1002–13.

8. Ohly H, Crossland N, Dykes F, Lowe N, Hall-Moran V. A realist review to explore how low-income pregnant women use food vouchers from the UK’s Healthy Start programme. BMJ Open. 2017 Apr 21;7(4):e013731.

**A realist evaluation of the role and functions of community health workers in service of a people-centred community health system.**

**Investigator CMOc Data Extraction Sheet Data Extraction**

The purpose of this document is to extract data from the identified studies in order to formulate one or more explanatory theories that explain Context-Mechanism-Outcome Configurations (CMOC) and to gain insight into how these CMOCs contribute to optimizing the role and functions of community health workers within a people-centred community health system.

In realist methodologies, knowledge accumulation centres on our comprehension of how mechanisms behave in various contexts and the reasons behind how and why this interplay of context and mechanism leads to different outcomes (1). Please fill in the details below for the study you will be assessing. In the subsequent three tables, locate and specify the Context, Mechanism, and Outcomes (CMO) of the intervention discussed in the study you've examined. Definitions and illustrations of CMO are provided within the tables.

**Kindly fill out the information below for the study you will be reviewing.**

| **Reviewer Name** | Usangiphile Buthelezi |
| --- | --- |
| **Reference** | Olakkengil M, Said S, Abdalla O, Hofmann R, Hedt-Gauthier B, Fulcher I. Are populations of postpartum women differentially served by community health worker programs: an observational cohort study from Zanzibar, Tanzania. BMC Pregnancy Childbirth. 2024 Mar 7;24(1):183. |
| **Country** | Tanzania |
| **Setting and population** | - The study took place in Zanzibar, Tanzania, specifically in 10 out of 11 districts, focusing on maternal health interventions. - Pregnant women enrolled in the Safer Deliveries program between January 2017 and June 2019 were included in the research. - Community health workers (CHWs) conducted the interventions by visiting the homes of the pregnant women within 42 days after childbirth. - Factors such as the women's parity, reporting of HIV status, use of phone consultations, and type of delivery facility they chose influenced the success of the interventions. - Women residing in different districts within Zanzibar experienced variations in the timing and likelihood of receiving the complete postpartum intervention. |
| **Aim** | The aim of the research was to investigate how the implementation of a community health worker-led maternal health intervention in Zanzibar affected different populations of pregnant women |
| **Objectives** | - The primary objective was to assess if certain demographic and health factors influenced the receipt of the complete postpartum intervention, consisting of three CHW visits, and the time taken for the first postpartum visit. - Another objective was to analyze the associations between variables like parity, HIV status reporting, phone consultations, and delivery facility type, to understand their impact on the effectiveness of the intervention. - The study aimed to identify disparities in receiving the postpartum intervention among women from different districts in Zanzibar to improve the targeting of maternal health services. |
| **Methodology** | - The study included pregnant women enrolled in the Safer Deliveries program in Zanzibar, Tanzania, between January 2017 and June 2019. - Data analysis involved investigating the associations between demographic/health characteristics and outcomes like receiving all postpartum visits and time to the first postpartum visit. - Statistical tests and multivariate regression analyses were conducted to estimate the relationships between various factors and the intervention outcomes. - Factors like parity, HIV status reporting, phone consultations, and delivery facility type were assessed for their impact on the intervention's effectiveness. - Distinct districts in Zanzibar were compared to understand differences in the timing and likelihood of receiving the postpartum intervention based on the women's residential areas. |
| **Intervention description** | - The Safer Deliveries program in Zanzibar provided a postpartum intervention to women enrolled from January 1, 2017, to June 19, 2019. - The intervention included three community health worker (CHW) visits to the women's homes within the 42-day postpartum period. - When CHWs couldn't reach the women physically due to various reasons like distance or relocation, a phone consultation option was available to avoid delays in the intervention. - Women who had postpartum phone consultations were found to be less likely to receive the entire postpartum intervention and experienced delays in their first postpartum visit compared to those who did not have phone consultations. - The intervention's success was influenced by factors like delivery facility type, HIV status reporting, parity, and district of residence, affecting the odds of receiving all postpartum visits and the time to the first postpartum visit |
| **Intervention outcomes** | - Factors impacting receipt of postpartum visits:   - Parity and unknown HIV status were associated with a lower likelihood of receiving all postpartum visits.   - Phone consultations also decreased the odds of receiving the full intervention. - Influence of delivery facility type:   - The type of delivery facility significantly affected the receipt of the full postpartum intervention. - Impact of postpartum phone consultations:   - Women who had postpartum phone consultations experienced delays in receiving the complete postpartum intervention. - Primary intervention method:   - Home-based visits were the main intervention, with phone consultations as an alternative for inaccessible cases. - Geographical residence effect:   - The district of residence played a role in the timely receipt of postpartum visits. |
| **Challenges and limitations** | - Exclusion of a proportion of women from the study due to loss to follow-up affected the representativeness of the sample, potentially biasing the results. - The study solely focused on women enrolled in the specific Safer Deliveries program in Zanzibar, limiting the generalizability of the findings to broader populations. - Omittance of women with unknown or unreported HIV status from the main analysis could have impacted the comprehensiveness of the results. - The reliance on program-enrolled women might introduce selection bias, as their health-seeking behaviors and cultural beliefs may differ from women not enrolled in such programs. |

**Table 1 – Context**

**Including (but not limited to) beliefs, social and cultural norms, regulations and economic factors (2,3). For example:**

- Individual capabilities (i.e. values, roles, knowledge, purpose).
- Interpersonal relationships supporting the interventions (i.e. communication, collaboration, network, influences).
- Institutional settings (i.e. informal rules, organisational culture, leadership, policies, resource allocation, local priorities).
- Infra-structural system (i.e. political support).
- Country and rurality (i.e. small or large/rural vs regional vs remote).
- Socio-demographic characteristics:
- Funding context or source (i.e. free, personalised, group vs. individual, government funded).

**Study Setting and Population**

- The study was conducted in Zanzibar, Tanzania, specifically in 10 out of the 11 districts.
- The population included pregnant women enrolled in the Safer Deliveries program, totaling 33,914 individuals.

**Training and Education Level**

- Community Health Workers (CHWs) received training to provide maternal health interventions through the program.
- CHWs directly visited pregnant women's homes and connected them to community resources for support and education.

**Available Resources in the Community**

- Women were linked to existing community resources like community transport systems and health facilities for delivery support and education.

**Funding and Economic Status**

- Details about the specific funding sources or economic status of the participants were not explicitly mentioned in the study contexts.

**Facilities and Education Level of CHWs**

- CHWs operated within communities and were guided by a mobile app for postpartum home visits.
- The education level of the CHWs was not detailed in the study; however, programs with educational requirements for CHWs tended to recruit from communities with higher educational levels.

**Table 2 – Mechanisms**

- **“An element of reasoning and/or reactions of an individual or collective agents(s) in regard of resources available in a given context to bring about changes through the implementation of an intervention” (4). Check what are the mediators to produce outcomes (not only primary, but also secondary or unintended outcomes).**

- CHWs operated within the community, ensuring that the intervention was delivered in a **familiar and comfortable setting** for the women.
- The program acknowledged the importance of **understanding and respecting the community's cultural beliefs and practices** regarding maternal health, potentially enhancing the effectiveness of the intervention.
- The success or failure of the intervention was influenced by factors such as **trust and rapport**, which were essential for building positive relationships between CHWs and pregnant women.
- **Motivation and accountability** played a crucial role, with CHWs needing to be motivated to provide timely interventions and be accountable for their actions to ensure the program's effectiveness.
- **Accessibility** was key, as the ease of CHWs reaching women in their homes positively impacted the intervention outcomes.
- **Competence and confidence** of the CHWs were vital, as their ability to provide quality maternal health services and their confidence in doing so contributed to the success of the program.
- **Recognition and support** for CHWs were mentioned as factors that boosted their dedication to the intervention, potentially impacting its success.
- **Cultural sensitivity** was highlighted, indicating that tailoring interventions to fit the community's cultural context enhanced acceptance and effectiveness of the program.

**Table 3 – Outcomes**

- **Please make note of all (intended or not) outcomes. Please also classify the role and functions outcomes of CHWs using the following Pillars for people-centred care:**

1. Engaging and empowering people and communities

2. Strengthening governance and accountability (Incl. Supervision, training, and support)

3. Reorienting the model of care

4. Coordinating services within and across sectors

5. Enabling environment

- Factors impacting receipt of postpartum visits:
  - Parity and unknown HIV status were associated with a lower likelihood of receiving all postpartum visits.
  - Phone consultations also decreased the odds of receiving the full intervention.
- Influence of delivery facility type:
  - The type of delivery facility significantly affected the receipt of the full postpartum intervention.
- Impact of postpartum phone consultations:
  - Women who had postpartum phone consultations experienced delays in receiving the complete postpartum intervention.
- Primary intervention method:
  - Home-based visits were the main intervention, with phone consultations as an alternative for inaccessible cases.
- Geographical residence effect:
  - The district of residence played a role in the timely receipt of postpartum visits.

**Outcomes according to PCC:**

- **Engaging and empowering people and communities:**
  - Parity and unknown HIV status were negatively associated with the likelihood of receiving all postpartum visits.
  - Phone consultations also reduced the odds of receiving the full intervention.
- **Strengthening governance and accountability:**
  - Type of delivery facility significantly influenced the receipt of the full postpartum intervention.
- **Reorienting the model of care:**
  - Women who had a postpartum phone consultation experienced delays in receiving the full postpartum intervention.
- **Coordinating services within and across sectors:**
  - Home-based visits were the primary intervention, with phone consultations as a backup option for inaccessible cases.
- **Enabling environment:**
  - Delays in the first postpartum visit were noted when women had postpartum phone consultations or unknown HIV status.
  - District of residence played a role in the timely receipt of postpartum visits.

**Table 4 – If, then statements depicting the CMOs.**

**Please detail the CMOs for the study you reviewed using the If (Context), Then (Mechanism) statements.**

**Test for relevance (Pearson et al. 2012; 2015; Brennan et al. 2017)**

| **Conceptually Rich** | **Thicker description’ but not ‘conceptually rich’** | **Conceptually Thin** |
| --- | --- | --- |
| Unambiguous theoretical concepts are described in sufficient depth. | Description of programme theory or sufficient information to enable it to ‘surface’. | Insufficient information to enable the programme theory to surface. |
| Relationships between, amongst concepts are clearly articulated. | Consideration of the context in which the programme takes place. | Limited or no consideration of the context in which the programme took place. |
| Concepts are sufficiently developed, defined to enable understanding without the reader needing to have first-hand experience of an area of practice. | Discussion of the differences between the design and orientation of programme theory (what was intended) and implementation (what really happened). | Limited or no discussion of the differences between the design and orientation of programme theory (what was intended) and implementation (what really happened). |
| Concepts are grounded strongly in a cited body of literature. | Recognition and discussion of the strengths/weaknesses of the implemented programme. | Limited or no discussion of the strengths/ weaknesses of the implemented programme. |
| Concepts are parsimonious (i.e., provide the simplest, but not over-simplified, explanation) | Some attempt to explain anomalous results and findings with reference to context and data. | No attempts to explain anomalous results and findings with reference to context and data. |
|  | Description of the factor affecting implementation. | Limited or no description of the factors affecting implementation. |

**Test for relevance (Pearson et al. 2012; 2015; Brennan et al. 2017)**

| **Conceptually Rich** | **Thicker description’ but not ‘conceptually rich’** | **Conceptually Thin** |
| --- | --- | --- |
|  | **X** |  |

**Test for rigour (Ohly et al. 2017)**

|  | **Yes** | **Fairly** | **No** |
| --- | --- | --- | --- |
| The study methods are clearly reported. | X |  |  |
| The study methods are appropriate to answer RQ. | X |  |  |
| The sample characteristics enable generalizability. |  |  | X |
| Raw data supports the study findings (conclusions). | X |  |  |
| Limitations of the study are acknowledged and clearly reported. | X |  |  |

**References**

1. Wong G, Westhorp G, Manzano A, Greenhalgh J, Jagosh J, Greenhalgh T. (2016). RAMESES II

reporting standards for realist evaluations. *BMC Medicine*, 14(1), 1-18.

2. Wong G, Greenhalgh T, Westhorp G, Pawson R. (2012). Realist methods in medical education

research: what are they and what can they contribute? *Medical Education*, 46(1), 89-96.

3. Macfarlane F, Greenhalgh T, Humphrey C, Hughes J, Butler C, Pawson R. (2011). A new workforce

in the making?: A case study of strategic human resource management in a whole-system change

effort in healthcare. *Journal of Health Organization and Management*, 25(1), 55-72.

4. Lacouture A, Breton E, Guichard A, Ridde V. (2015). The concept of mechanism from a realist

approach: a scoping review to facilitate its operationalization in public health program evaluation.

*Implementation Science*, 10(1), 1-10.

5. Pearson M, Chilton R, Woods HB, Wyatt K, Ford T, Abraham C, et al. Implementing health promotion in schools: protocol for a realist systematic review of research and experience in the United Kingdom (UK). Syst Rev. 2012 Oct 20;1(1):48.

6. Pearson M, Brand SL, Quinn C, Shaw J, Maguire M, Michie S, et al. Using realist review to inform intervention development: methodological illustration and conceptual platform for collaborative care in offender mental health. Implementation Sci. 2015 Sep 28;10(1):134.

7. Brennan N, Bryce M, Pearson M, Wong G, Cooper C, Archer J. Towards an understanding of how appraisal of doctors produces its effects: a realist review. Medical Education. 2017;51(10):1002–13.

8. Ohly H, Crossland N, Dykes F, Lowe N, Hall-Moran V. A realist review to explore how low-income pregnant women use food vouchers from the UK’s Healthy Start programme. BMJ Open. 2017 Apr 21;7(4):e013731.

**A realist evaluation of the role and functions of community health workers in service of a people-centred community health system.**

**Investigator CMOc Data Extraction Sheet Data Extraction**

The purpose of this document is to extract data from the identified studies in order to formulate one or more explanatory theories that explain Context-Mechanism-Outcome Configurations (CMOC) and to gain insight into how these CMOCs contribute to optimizing the role and functions of community health workers within a people-centred community health system.

In realist methodologies, knowledge accumulation centres on our comprehension of how mechanisms behave in various contexts and the reasons behind how and why this interplay of context and mechanism leads to different outcomes (1). Please fill in the details below for the study you will be assessing. In the subsequent three tables, locate and specify the Context, Mechanism, and Outcomes (CMO) of the intervention discussed in the study you've examined. Definitions and illustrations of CMO are provided within the tables.

**Kindly fill out the information below for the study you will be reviewing.**

| **Reviewer Name** | Usangiphile Buthelezi |
| --- | --- |
| **Reference** | Pollard R, Kennedy CE, Hutton HE, Mulamba J, Mbabali I, Anok A, et al. HIV Prevention and Treatment Behavior Change and the Situated Information Motivation Behavioral Skills (sIMB) Model: A Qualitative Evaluation of a Community Health Worker Intervention in Rakai, Uganda. AIDS Behav. 2022 Feb;26(2):375–84. |
| **Country** | Uganda |
| **Setting and population** | - The study was conducted in a fishing community in Rakai, Uganda. - The population consisted of highly mobile individuals, which presented challenges to implementation. - The community's history of high HIV prevalence, patterns of mobility, cultural norms of substance use, widespread HIV stigma, and perceptions of HIV influenced by the community's history of high HIV prevalence were important contextual elements. - The fishing community's social and structural environment played a defining role in shaping client behaviors. Factors such as high HIV prevalence, periodic migration, and mobility were identified as structural factors with implications for HIV transmission and behavior change. |
| **Aim** | - The aim of the study was to evaluate the effectiveness of a community health worker (CHW) intervention called Health Scouts in promoting HIV prevention and treatment behaviors in a fishing community in Rakai, Uganda |
| **Objectives** | - The study aimed to assess how the situated Information, Motivation, and Behavioral Skills (sIMB) framework informed the design and evaluation of the intervention. - The study aimed to understand the role of Health Scouts in providing information about HIV prevention and treatment behaviors and helping clients manage personal and social motivations to carry out health-promoting behavior. - The study aimed to explore the impact of the Health Scout intervention on clients' knowledge and attitudes towards HIV treatment, including dispelling misconceptions and increasing comfort with the idea of living healthily with HIV. |
| **Methodology** | - The study used a qualitative evaluation approach, conducting semi-structured, in-depth interviews with 51 clients of the Health Scout intervention in Rakai, Uganda. - Clients were recruited through random purposeful sampling, with a sampling frame based on gender, HIV status, Health Scout assignment, and age categories. - Trained ethnographers conducted the interviews in Luganda, which were digitally recorded, transcribed, and translated to English. - The interviews focused on the overall experience with the intervention, sIMB framework dimensions, interactions with Health Scouts, and the context of carrying out health behaviors in the fishing village. - The transcripts were coded using an iterative process, with an initial codebook developed deductively based on the sIMB framework. Additional codes were added inductively to capture contextual factors. - Data analysis involved mapping themes across the sIMB elements and contextual factors, using QSR NVivo Version 12.3 for data management and coding. - The study also considered the situated context, including community-level factors (health services, commodities, HIV stigma) and structural factors (patterns of mobility, cultural norms) that influenced behavior change. |
| **Intervention description** | - The Health Scout intervention was implemented by the Rakai Health Sciences Program in a fishing community in Rakai, Uganda. - The intervention employed community health workers (CHWs) called Health Scouts to provide one-on-one counseling sessions on HIV prevention and treatment behaviors. - Health Scouts used counseling skills informed by motivational interviewing (MI), which emphasizes a nonjudgmental and nonconfrontational approach to mobilize the client's own motivation to change. - The intervention targeted behaviors such as PrEP use, condom use, HIV testing, engagement in male circumcision, antiretroviral therapy (ART) adherence, and engagement in HIV care services. - During each visit, Health Scouts used a smartphone application with theory-based, MI-informed prompts for tailored messaging and psychosocial support. - The intervention aimed to provide information about HIV prevention and treatment behaviors, help clients manage personal and social motivations, and promote health-promoting behavior. - Health Scouts worked to maintain visit continuity despite client mobility, using phone or in-person contact to schedule visits and making multiple attempts to locate unfound clients. - The intervention also focused on providing support and counseling to clients, which helped in their adoption and maintenance of behaviors. |
| **Intervention outcomes** | - The Health Scout intervention improved HIV care and antiretroviral therapy (ART) coverage in intervention clusters compared to control clusters. - The intervention did not clearly improve male circumcision coverage or HIV viral suppression. - Clients reported that the support and counseling provided by Health Scouts bolstered their adoption and maintenance of HIV prevention and treatment behaviors. - The intervention successfully addressed personal and social motivations for behavior change, such as self-preservation for a long and healthy life and navigating partner reactions to HIV preventive behaviors. - Elements that moved clients away from behavior change included daily pill burdens, serostatus disclosure, fear of testing positive, substance use, and anticipated partner reactions. - Clients' level of familiarity and trust with Health Scouts shaped their willingness to engage in open discussion and entertain ideas of behavior change. |
| **Challenges and limitations** | - The highly mobile nature of the study population presented challenges to the implementation of the Health Scout intervention, requiring strategies such as maintaining visit continuity and designating new Health Scouts for clients who moved into another intervention cluster. - Despite the intervention's efforts, some clients still faced barriers to adopting risk-reducing sexual behaviors, such as frequent social gatherings and substance use facilitating casual sexual encounters. - Stigma and fear of disclosure were identified as barriers to accessing and remaining in HIV care, which is consistent with previous research in sub-Saharan Africa. - The study focused on a specific fishing community in Rakai, Uganda, which may limit the generalizability of the findings to other populations or settings. - The intervention outcomes showed improvements in HIV care and ART coverage but did not clearly improve male circumcision coverage or HIV viral suppression. - The study relied on qualitative interviews with intervention clients, which may be subject to recall bias or social desirability bias. |

**Table 1 – Context**

**Including (but not limited to) beliefs, social and cultural norms, regulations and economic factors (2,3). For example:**

- Individual capabilities (i.e. values, roles, knowledge, purpose).
- Interpersonal relationships supporting the interventions (i.e. communication, collaboration, network, influences).
- Institutional settings (i.e. informal rules, organisational culture, leadership, policies, resource allocation, local priorities).
- Infra-structural system (i.e. political support).
- Country and rurality (i.e. small or large/rural vs regional vs remote).
- Socio-demographic characteristics:
- Funding context or source (i.e. free, personalised, group vs. individual, government funded).

- The study was conducted in a fishing community in Rakai, Uganda.
- The population consisted of highly mobile individuals, which presented challenges to implementation.
- The community's history of high HIV prevalence, patterns of mobility, cultural norms of substance use, widespread HIV stigma, and perceptions of HIV influenced by the community's history of high HIV prevalence were important contextual elements.
- The fishing community's social and structural environment played a defining role in shaping client behaviors. Factors such as high HIV prevalence, periodic migration, and mobility were identified as structural factors with implications for HIV transmission and behavior change.
- The Health Scout intervention was designed to be relevant to highly mobile and HIV-hyperendemic populations, emphasizing the need to consider the situated context of the community.

**Table 2 – Mechanisms**

- **“An element of reasoning and/or reactions of an individual or collective agents(s) in regard of resources available in a given context to bring about changes through the implementation of an intervention” (4). Check what are the mediators to produce outcomes (not only primary, but also secondary or unintended outcomes).**

- Clients' perceptions of the Health Scouts and their level of familiarity and trust influenced their willingness to engage in open discussion and behavior change.
- The emotional support and encouragement provided by the Health Scouts contributed to clients' motivation and activation of HIV prevention and treatment behaviors.
- The Health Scouts' counseling sessions helped clients manage personal and social motivations, leading to the adoption and maintenance of health-promoting behaviors.
- The intervention provided information about HIV prevention and treatment behaviors, increasing clients' awareness and knowledge.
- The support and counseling from the Health Scouts helped clients overcome barriers such as stigma, fear of disclosure, and anticipated reactions of partners, leading to behavior change.
- The use of motivational interviewing techniques by the Health Scouts facilitated value clarification, communication, and the development of behavioral skills.
- The socioecological context, including community-level factors and structural factors, influenced behavior change by shaping clients' perceptions and experiences of HIV.

**Table 3 – Outcomes**

- **Please make note of all (intended or not) outcomes. Please also classify the role and functions outcomes of CHWs using the following Pillars for people-centred care:**

1. Engaging and empowering people and communities

2. Strengthening governance and accountability (Incl. Supervision, training, and support)

3. Reorienting the model of care

4. Coordinating services within and across sectors

5. Enabling environment

- The Health Scout intervention improved HIV care and antiretroviral therapy (ART) coverage in intervention clusters compared to control clusters.
- The intervention did not clearly improve male circumcision coverage or HIV viral suppression.
- Clients reported that the support and counseling provided by Health Scouts bolstered their adoption and maintenance of HIV prevention and treatment behaviors.
- The intervention successfully addressed personal and social motivations for behavior change, such as self-preservation for a long and healthy life and navigating partner reactions to HIV preventive behaviors.
- Elements that moved clients away from behavior change included daily pill burdens, serostatus disclosure, fear of testing positive, substance use, and anticipated partner reactions.
- Clients' level of familiarity and trust with Health Scouts shaped their willingness to engage in open discussion and entertain ideas of behavior change.

**Outcomes by PCC pillars:**

**Engaging and empowering people and communities:**

- The Health Scout intervention engaged community health workers (CHWs) called Health Scouts to provide one-on-one counseling sessions on HIV prevention and treatment behaviors, empowering individuals to take control of their health.
- The intervention aimed to provide information, motivation, and behavioral skills to clients, empowering them to carry out health-promoting behaviors.
- Clients reported that the Health Scouts helped them manage personal and social motivations, leading to behavior change and empowerment.

**Strengthening governance and accountability (Incl. Supervision, training, and support):**

- The Rakai Health Sciences Program implemented the Health Scout intervention, which involved training and support for the community health workers (Health Scouts) who delivered the intervention.

**Reorienting the model of care:**

- The Health Scout intervention employed a community health worker (CHW) model, which reoriented the model of care by utilizing CHWs to provide one-on-one counseling sessions on HIV prevention and treatment behaviors.

**Coordinating services within and across sectors:**

- The Health Scout intervention aimed to coordinate services by providing one-on-one counseling sessions on various HIV prevention and treatment behaviors, including PrEP use, condom use, HIV testing, engagement in male circumcision, antiretroviral therapy (ART) adherence, and engagement in HIV care services.

**Enabling environment:**

- The intervention took into account the socioecological context, including community-level factors and structural factors, which influenced behavior change and created an enabling environment for HIV prevention and treatment.

**Table 4 – If, then statements depicting the CMOs.**

**Please detail the CMOs for the study you reviewed using the If (Context), Then (Mechanism) statements.**

**If, Then statements**

- If CHWs apply motivational interviewing techniques, then this facilitates value clarification by helping clients explore and articulate their personal values and goals related to the intervention, enhancing motivation for action, leading to increased engagement in behavior change and improved health outcomes.
- If CHWs apply motivational interviewing techniques, then there will be enhanced communication, fostered by a nonjudgmental and empathetic environment for clients to express their thoughts and concerns, leading to greater trust, openness, and collaboration in the client-provider relationship, ultimately behavior change and improved health outcomes.
- If clients are familiar with their CHW, then they develop trust which influences their willingness to engage in open discussions, leading to behaviour change.
- If CHWs provide emotional support and encouragement to the client, then they become motivated and actively participate in the intervention offered by the CHW, leading to better engagement and behaviour change.
- If the CHWs offers health information to the clients, then this increases client’s awareness and knowledge, fostering empowerment, which results in self-efficacy and ownership of their health.

**Test for relevance (Pearson et al. 2012; 2015; Brennan et al. 2017)**

| **Conceptually Rich** | **Thicker description’ but not ‘conceptually rich’** | **Conceptually Thin** |
| --- | --- | --- |
| Unambiguous theoretical concepts are described in sufficient depth. | Description of programme theory or sufficient information to enable it to ‘surface’. | Insufficient information to enable the programme theory to surface. |
| Relationships between, amongst concepts are clearly articulated. | Consideration of the context in which the programme takes place. | Limited or no consideration of the context in which the programme took place. |
| Concepts are sufficiently developed, defined to enable understanding without the reader needing to have first-hand experience of an area of practice. | Discussion of the differences between the design and orientation of programme theory (what was intended) and implementation (what really happened). | Limited or no discussion of the differences between the design and orientation of programme theory (what was intended) and implementation (what really happened). |
| Concepts are grounded strongly in a cited body of literature. | Recognition and discussion of the strengths/weaknesses of the implemented programme. | Limited or no discussion of the strengths/ weaknesses of the implemented programme. |
| Concepts are parsimonious (i.e., provide the simplest, but not over-simplified, explanation) | Some attempt to explain anomalous results and findings with reference to context and data. | No attempts to explain anomalous results and findings with reference to context and data. |
|  | Description of the factor affecting implementation. | Limited or no description of the factors affecting implementation. |

**Test for relevance (Pearson et al. 2012; 2015; Brennan et al. 2017)**

| **Conceptually Rich** | **Thicker description’ but not ‘conceptually rich’** | **Conceptually Thin** |
| --- | --- | --- |
| X |  |  |

**Test for rigour (Ohly et al. 2017)**

|  | **Yes** | **Fairly** | **No** |
| --- | --- | --- | --- |
| The study methods are clearly reported. | X |  |  |
| The study methods are appropriate to answer RQ. | X |  |  |
| The sample characteristics enable generalizability. |  |  | X |
| Raw data supports the study findings (conclusions). | X |  |  |
| Limitations of the study are acknowledged and clearly reported. | X |  |  |

**References**

1. Wong G, Westhorp G, Manzano A, Greenhalgh J, Jagosh J, Greenhalgh T. (2016). RAMESES II

reporting standards for realist evaluations. *BMC Medicine*, 14(1), 1-18.

2. Wong G, Greenhalgh T, Westhorp G, Pawson R. (2012). Realist methods in medical education

research: what are they and what can they contribute? *Medical Education*, 46(1), 89-96.

3. Macfarlane F, Greenhalgh T, Humphrey C, Hughes J, Butler C, Pawson R. (2011). A new workforce

in the making?: A case study of strategic human resource management in a whole-system change

effort in healthcare. *Journal of Health Organization and Management*, 25(1), 55-72.

4. Lacouture A, Breton E, Guichard A, Ridde V. (2015). The concept of mechanism from a realist

approach: a scoping review to facilitate its operationalization in public health program evaluation.

*Implementation Science*, 10(1), 1-10.

5. Pearson M, Chilton R, Woods HB, Wyatt K, Ford T, Abraham C, et al. Implementing health promotion in schools: protocol for a realist systematic review of research and experience in the United Kingdom (UK). Syst Rev. 2012 Oct 20;1(1):48.

6. Pearson M, Brand SL, Quinn C, Shaw J, Maguire M, Michie S, et al. Using realist review to inform intervention development: methodological illustration and conceptual platform for collaborative care in offender mental health. Implementation Sci. 2015 Sep 28;10(1):134.

7. Brennan N, Bryce M, Pearson M, Wong G, Cooper C, Archer J. Towards an understanding of how appraisal of doctors produces its effects: a realist review. Medical Education. 2017;51(10):1002–13.

8. Ohly H, Crossland N, Dykes F, Lowe N, Hall-Moran V. A realist review to explore how low-income pregnant women use food vouchers from the UK’s Healthy Start programme. BMJ Open. 2017 Apr 21;7(4):e013731.

**A realist evaluation of the role and functions of community health workers in service of a people-centred community health system.**

**Investigator CMOc Data Extraction Sheet Data Extraction**

The purpose of this document is to extract data from the identified studies in order to formulate one or more explanatory theories that explain Context-Mechanism-Outcome Configurations (CMOC) and to gain insight into how these CMOCs contribute to optimizing the role and functions of community health workers within a people-centred community health system.

In realist methodologies, knowledge accumulation centres on our comprehension of how mechanisms behave in various contexts and the reasons behind how and why this interplay of context and mechanism leads to different outcomes (1). Please fill in the details below for the study you will be assessing. In the subsequent three tables, locate and specify the Context, Mechanism, and Outcomes (CMO) of the intervention discussed in the study you've examined. Definitions and illustrations of CMO are provided within the tables.

**Kindly fill out the information below for the study you will be reviewing.**

| **Reviewer Name** | Usangiphile Buthelezi |
| --- | --- |
| **Reference** | Razafinjato B, Rakotonirina L, Cordier LF, Rasoarivao A, Andrianomenjanahary M, Marovavy L, et al. Evaluation of a novel approach to community health care delivery in Ifanadiana District, Madagascar. PLOS Glob Public Health. 2024;4(3):e0002888. |
| **Country** | Madagascar |
| **Setting and population** | - The study was conducted in remote rural Madagascar, specifically in the Ifanadiana District. - It focused on community health care delivery within the local administrative units called communes. - The intervention commune implemented the Enhanced Community Health (ECH) model, with one stationary Community Health Worker (CHW) and additional CHWs conducting proactive household visits. - The comparison communes had different models of community health programs but also received support. - The population studied included children under five years old who needed health care services. |
| **Aim** | The aim of the study was to evaluate a new approach called Enhanced Community Health (ECH) in Ifanadiana District, Madagascar. |
| **Objectives** | - The primary objective was to assess the impact of the ECH model on health outcomes, including care utilization and quality of service delivery. - Specific objectives included comparing health service utilization between the intervention commune and comparison communes, analyzing the effectiveness of proactive household visits by CHWs, and measuring adherence to the iCCM protocol. - The study aimed to provide evidence on how the ECH model could improve access to care and enhance the quality of health services in rural settings with limited healthcare access. |
| **Methodology** | - The study used a quasi-experimental design to compare the Enhanced Community Health (ECH) model in one commune with five other comparison communes. - Data collected from October 2019 to March 2021 focused on health service utilization and quality indicators. - The ECH model involved targeted recruitment, extended training, financial compensation for Community Health Workers (CHWs), and twice monthly supervision. - Programmatic data was analyzed to assess the impact of ECH on health outcomes like sick child visits and adherence to care protocols. - Statistical analysis was used to compare results between the intervention commune and comparison communes to evaluate the effectiveness of the ECH model. - The study's methodology aimed to provide evidence on how the ECH approach could positively impact healthcare delivery in remote settings with limited access to health services. |
| **Intervention description** | - The study introduced the Enhanced Community Health (ECH) model in Ifanadiana District, Madagascar. - In this model, Community Health Workers (CHWs) provided care at a stationary site and conducted proactive household visits to deliver health services. - CHWs received special training, financial compensation, and regular supervision to enhance their skills and service delivery. - Supervisors monitored the quality of care provided by CHWs, completing 376 supervision visits and observing 636 sick child visits to ensure proper care. - The ECH model aimed to improve access to health care in remote areas and enhance the quality of services delivered to the community, especially for children under five years old. |
| **Intervention outcomes** | - The ECH intervention resulted in a significant increase in consultations with children under-five by Community Health Workers (CHWs) in the intervention commune. - The average monthly per-capita utilization of health services was higher in the ECH area compared to the comparison communes. - In the ECH intervention commune, 40.3% of visits were completed at households through proactive care, improving access to healthcare services. - CHWs in the intervention commune demonstrated a higher adherence to the integrated Community Case Management (iCCM) protocol compared to CHWs in the comparison communes, indicating improved quality of care. - Overall, the outcomes of the ECH model highlighted improved access to care, increased utilization of health services, and enhanced quality of service delivery in the rural health district of Ifanadiana, Madagascar. |
| **Challenges and limitations** | - The study faced challenges due to the lack of reliable data from Community Health Workers' registries, making it difficult to compare the intervention area with the national model. - The implementation of the Community Health program in Madagascar encountered obstacles such as limited supervision, resources, medication stock outs, variable training quality, and incomplete data on service provision. - Another limitation was the national policy that does not provide salaries to CHWs, leading them to rely on selling medications for income, potentially affecting their financial stability and commitment to healthcare services. - Additionally, CHWs often have other formal work commitments, which may influence their availability to consistently deliver healthcare according to a regular schedule. |

**Table 1 – Context**

**Including (but not limited to) beliefs, social and cultural norms, regulations and economic factors (2,3). For example:**

- Individual capabilities (i.e. values, roles, knowledge, purpose).
- Interpersonal relationships supporting the interventions (i.e. communication, collaboration, network, influences).
- Institutional settings (i.e. informal rules, organisational culture, leadership, policies, resource allocation, local priorities).
- Infra-structural system (i.e. political support).
- Country and rurality (i.e. small or large/rural vs regional vs remote).
- Socio-demographic characteristics:
- Funding context or source (i.e. free, personalised, group vs. individual, government funded).

- Community Health Workers (CHWs) in Madagascar face challenges due to limited supervision and resources, medication stock outs, variable training quality, and insufficient data on service provision.
- The existing policy design and its implementation fidelity contribute to the limitations of Madagascar's CH program.
- National policy dictates that CHWs are not salaried but instead rely on selling medications for profit, which can impact their financial stability and dedication to service provision.
- CHWs also engage in other formal work, which may affect their availability to provide healthcare consistently according to a set schedule.

**Table 2 – Mechanisms**

- **“An element of reasoning and/or reactions of an individual or collective agents(s) in regard of resources available in a given context to bring about changes through the implementation of an intervention” (4). Check what are the mediators to produce outcomes (not only primary, but also secondary or unintended outcomes).**

- **Supervision and Training:** Regular supervision and quality training sessions for Community Health Workers (CHWs) were vital for ensuring competence and confidence in service delivery.
- **Financial Compensation:** Providing financial incentives to CHWs could enhance their motivation, financial stability, and commitment to healthcare services.
- **Policy Support:** Clear national policies ensuring consistent salaries and support for CHWs could improve their accountability, sense of purpose, and feeling valued within the healthcare system.
- **Resources Availability:** Ensuring adequate provision of resources and medications can build trust, enhance credibility, and improve the overall quality of care provided by CHWs.
- **Peer Support and Recognition:** Fostering a culture of peer support, recognition for their work, and feeling supported could boost morale and increase the effectiveness of CHWs in delivering care.

**Table 3 – Outcomes**

- **Please make note of all (intended or not) outcomes. Please also classify the role and functions outcomes of CHWs using the following Pillars for people-centred care:**

1. Engaging and empowering people and communities

2. Strengthening governance and accountability (Incl. Supervision, training, and support)

3. Reorienting the model of care

4. Coordinating services within and across sectors

5. Enabling environment

- The ECH intervention resulted in a significant increase in consultations with children under-five by Community Health Workers (CHWs) in the intervention commune.
- The average monthly per-capita utilization of health services was higher in the ECH area compared to the comparison communes.
- In the ECH intervention commune, 40.3% of visits were completed at households through proactive care, improving access to healthcare services.
- CHWs in the intervention commune demonstrated a higher adherence to the integrated Community Case Management (iCCM) protocol compared to CHWs in the comparison communes, indicating improved quality of care.
- Overall, the outcomes of the ECH model highlighted improved access to care, increased utilization of health services, and enhanced quality of service delivery in the rural health district of Ifanadiana, Madagascar.

**Outcomes according to PCC pillars:**

***Engaging and Empowering People and Communities***

- Community Health Workers (CHWs) in the intervention commune demonstrated improved evaluation of danger signs, correct treatment of illness, and counselling of caregivers, empowering them to provide better care.

***Strengthening Governance and Accountability***

- The ECH model introduced professionalized CHWs and enhanced supervision, leading to increased adherence to care protocols and improved quality of care.

***Reorienting the Model of Care***

- The ECH model focused on proactive household visits by CHWs, resulting in better access to healthcare services for the community, especially for children under-five.

***Coordinating Services Within and Across Sectors***

- Supervisors from other communes were involved in providing supervision to CHWs in the intervention area, ensuring consistent support and quality of care.

***Enabling Environment***

- The ECH model created a supportive environment for CHWs through targeted recruitment, extended training, financial compensation, and regular supervision, enabling them to deliver high-quality care.

**Table 4 – If, then statements depicting the CMOs.**

**Please detail the CMOs for the study you reviewed using the If (Context), Then (Mechanism) statements.**

**IF** regular supervision and quality training sessions, financial compensation, clear national policies, adequate provision of resources, peer support, and recognition are provided to Community Health Workers (CHWs), and the ECH model is implemented to focus on proactive household visits, professionalized CHWs, and enhanced supervision with coordinated services across sectors, **THEN** CHWs will feel supported and empowered to deliver high-quality, comprehensive, and timely care, **BECAUSE** these combined efforts address the critical needs for competence, motivation, job satisfaction, resource availability, and a supportive working environment, leading to improved health outcomes for the community.

**Test for relevance (Pearson et al. 2012; 2015; Brennan et al. 2017)**

| **Conceptually Rich** | **Thicker description’ but not ‘conceptually rich’** | **Conceptually Thin** |
| --- | --- | --- |
| Unambiguous theoretical concepts are described in sufficient depth. | Description of programme theory or sufficient information to enable it to ‘surface’. | Insufficient information to enable the programme theory to surface. |
| Relationships between, amongst concepts are clearly articulated. | Consideration of the context in which the programme takes place. | Limited or no consideration of the context in which the programme took place. |
| Concepts are sufficiently developed, defined to enable understanding without the reader needing to have first-hand experience of an area of practice. | Discussion of the differences between the design and orientation of programme theory (what was intended) and implementation (what really happened). | Limited or no discussion of the differences between the design and orientation of programme theory (what was intended) and implementation (what really happened). |
| Concepts are grounded strongly in a cited body of literature. | Recognition and discussion of the strengths/weaknesses of the implemented programme. | Limited or no discussion of the strengths/ weaknesses of the implemented programme. |
| Concepts are parsimonious (i.e., provide the simplest, but not over-simplified, explanation) | Some attempt to explain anomalous results and findings with reference to context and data. | No attempts to explain anomalous results and findings with reference to context and data. |
|  | Description of the factor affecting implementation. | Limited or no description of the factors affecting implementation. |

**Test for relevance (Pearson et al. 2012; 2015; Brennan et al. 2017)**

| **Conceptually Rich** | **Thicker description’ but not ‘conceptually rich’** | **Conceptually Thin** |
| --- | --- | --- |
|  | **X** |  |

**Test for rigour (Ohly et al. 2017)**

|  | **Yes** | **Fairly** | **No** |
| --- | --- | --- | --- |
| The study methods are clearly reported. | X |  |  |
| The study methods are appropriate to answer RQ. | X |  |  |
| The sample characteristics enable generalizability. |  |  | X |
| Raw data supports the study findings (conclusions). | X |  |  |
| Limitations of the study are acknowledged and clearly reported. | X |  |  |

**References**

1. Wong G, Westhorp G, Manzano A, Greenhalgh J, Jagosh J, Greenhalgh T. (2016). RAMESES II

reporting standards for realist evaluations. *BMC Medicine*, 14(1), 1-18.

2. Wong G, Greenhalgh T, Westhorp G, Pawson R. (2012). Realist methods in medical education

research: what are they and what can they contribute? *Medical Education*, 46(1), 89-96.

3. Macfarlane F, Greenhalgh T, Humphrey C, Hughes J, Butler C, Pawson R. (2011). A new workforce

in the making?: A case study of strategic human resource management in a whole-system change

effort in healthcare. *Journal of Health Organization and Management*, 25(1), 55-72.

4. Lacouture A, Breton E, Guichard A, Ridde V. (2015). The concept of mechanism from a realist

approach: a scoping review to facilitate its operationalization in public health program evaluation.

*Implementation Science*, 10(1), 1-10.

5. Pearson M, Chilton R, Woods HB, Wyatt K, Ford T, Abraham C, et al. Implementing health promotion in schools: protocol for a realist systematic review of research and experience in the United Kingdom (UK). Syst Rev. 2012 Oct 20;1(1):48.

6. Pearson M, Brand SL, Quinn C, Shaw J, Maguire M, Michie S, et al. Using realist review to inform intervention development: methodological illustration and conceptual platform for collaborative care in offender mental health. Implementation Sci. 2015 Sep 28;10(1):134.

7. Brennan N, Bryce M, Pearson M, Wong G, Cooper C, Archer J. Towards an understanding of how appraisal of doctors produces its effects: a realist review. Medical Education. 2017;51(10):1002–13.

8. Ohly H, Crossland N, Dykes F, Lowe N, Hall-Moran V. A realist review to explore how low-income pregnant women use food vouchers from the UK’s Healthy Start programme. BMJ Open. 2017 Apr 21;7(4):e013731.

**A realist evaluation of the role and functions of community health workers in service of a people-centred community health system.**

**Investigator CMOc Data Extraction Sheet Data Extraction**

The purpose of this document is to extract data from the identified studies in order to formulate one or more explanatory theories that explain Context-Mechanism-Outcome Configurations (CMOC) and to gain insight into how these CMOCs contribute to optimizing the role and functions of community health workers within a people-centred community health system.

In realist methodologies, knowledge accumulation centres on our comprehension of how mechanisms behave in various contexts and the reasons behind how and why this interplay of context and mechanism leads to different outcomes (1). Please fill in the details below for the study you will be assessing. In the subsequent three tables, locate and specify the Context, Mechanism, and Outcomes (CMO) of the intervention discussed in the study you've examined. Definitions and illustrations of CMO are provided within the tables.

**Kindly fill out the information below for the study you will be reviewing.**

| **Reviewer Name** | Usangiphile Buthelezi |
| --- | --- |
| **Reference** | Rogers A, Goore LL, Wamae J, Starnes JR, Okong’o SO, Okoth V, et al. Training and experience outperform literacy and formal education as predictors of community health worker knowledge and performance, results from Rongo sub-county, Kenya. Front Public Health [Internet]. 2023 Apr 27 [cited 2024 Apr 3];11. Available from: https://www.frontiersin.org/journals/public-health/articles/10.3389/fpubh.2023.1120922/full |
| **Country** | Kenya |
| **Setting and population** | - The study was conducted in Rongo sub-county, Kenya, as part of an intervention implemented by Lwala Community Alliance and the Kenya Ministry of Health to professionalize Community Health Worker (CHW) cadres. - The study also took place in Migori County, Kenya, where the Ministry of Health partnered with Lwala Community Alliance to implement a professionalized CHW program. - The population of interest in the study was Community Health Workers (CHWs) who were part of the intervention program. - The CHWs included in the study were from four different cohorts, with some cohorts receiving the intervention prior to the baseline and others receiving it between the baseline and endline. - The study surveyed CHWs both at the beginning and end of the study period, with the entire CHW population being surveyed to avoid sampling bias. |
| **Aim** | The aim of the study was to examine the predictors of CHW knowledge and performance in achieving antenatal care and immunization uptake among their clients in Rongo sub-county, Kenya. |
| **Objectives** | - The study aimed to assess the impact of an intervention implemented by Lwala Community Alliance and the Kenya Ministry of Health, which aimed to professionalize CHW cadres through enhanced training, payment, and supervision. - The study aimed to explore the association between education, literacy, experience, training, and gender with CHW performance. It aimed to determine which factors were better predictors of CHW competency. |
| **Methodology** | - The study was conducted in Rongo sub-county, Kenya, as part of an intervention implemented by Lwala Community Alliance and the Kenya Ministry of Health to professionalize CHW cadres through enhanced training, payment, and supervision. - Four cohorts of CHWs were included in the study, with data collected on their demographics, knowledge tests, and key performance indicators. - The data were collected for 234 CHWs. - Regression analyses were used to explore the predictors of CHW performance, including education, literacy, experience, training, and gender. - The study assessed the impact of the intervention on CHW performance by comparing the immunization uptake and completion of antenatal care visits among clients of CHWs who received the intervention versus those who did not. - Literacy tests consisting of 7 questions were conducted, with a passing score of 6 out of 7. The literacy rates varied across different regions, with the East region having the highest literacy rate and the Central region having the lowest. - Validated tools were used to assess CHW knowledge, including questions derived from Jhpiego's training materials and a perception of supervision scale. Literacy was assessed using a brief assessment based on Kenya's nationwide literacy exam. - The sample size of CHWs surveyed was determined based on the number of CHWs working in the geographic area, ensuring adequate power for the primary outcome of CHW knowledge and secondary outcomes. |
| **Intervention description** | - The intervention in the study aimed to professionalize Community Health Worker (CHW) cadres through enhanced training, payment, and supervision. - The intervention was implemented jointly by Lwala Community Alliance and the Kenya Ministry of Health in Rongo sub-county, Kenya. - CHWs in the intervention received enhanced training, including a 7-day training on Kenya's Community Health Volunteer Basic Module and a 5-day training on the Maternal, Newborn, and Child Health Technical Module. - Additional training on technical modules and periodic refresher training were provided as CHWs progressed through the program. - CHWs in the intervention were also provided with monthly payment based on the service package they were delivering, ranging from 40-60 USD. - The intervention aimed to improve CHW performance in achieving antenatal care and immunization uptake among their clients. - The intervention was found to be predictive of increased CHW performance, with clients of trained CHWs being more likely to be fully immunized and complete four or more antenatal care visits. |
| **Intervention outcomes** | - The intervention aimed to professionalize Community Health Worker (CHW) cadres through enhanced training, payment, and supervision. - Trained CHWs were 15% more likely to have fully immunized clients and 14% more likely to have clients who completed four or more antenatal care visits. - Recency of training and experience caring for pregnant women were associated with increased CHW knowledge. - There was no association between gender and CHW knowledge or performance. - The link between education and literacy and CHW performance and knowledge was inconsistent, suggesting that robust training and supervision may compensate for lower education or literacy levels. - Further research is recommended to investigate the influence of other program components, such as payment, supervision, and refresher training, on CHW performance. - CHWs in the intervention showed significant increases in knowledge scores across all domains, particularly in pregnancy-related knowledge. - The intervention's training was significantly associated with CHW performance, particularly in immunization and antenatal care. - Overall, the intervention demonstrated positive outcomes in terms of CHW performance and knowledge, with training and experience playing significant roles. The findings suggest that education and literacy may not be strong predictors of CHW competency, highlighting the importance of robust training and supervision in CHW programs. Further research is recommended to explore the influence of other program components on CHW performance. |
| **Challenges and limitations** | - The study did not include other intervention elements, such as payment and supervision, which may have influenced CHW knowledge and performance. - The study took place during the COVID-19 pandemic, and additional training and support, including personal protective equipment, were provided to many CHWs, potentially introducing confounding factors. - The study only included CHWs from geographically adjacent areas, which may have led to spillover effects between intervention and non-intervention cohorts. - The sample size for the study was based on the number of CHWs in the geographic area, which may have limited the statistical power for certain outcomes. - The study did not include sufficient numbers of former Traditional Birth Attendants (TBAs) to analyze the impact of TBA experience on CHW performance |

**Table 1 – Context**

**Including (but not limited to) beliefs, social and cultural norms, regulations and economic factors (2,3). For example:**

- Individual capabilities (i.e. values, roles, knowledge, purpose).
- Interpersonal relationships supporting the interventions (i.e. communication, collaboration, network, influences).
- Institutional settings (i.e. informal rules, organisational culture, leadership, policies, resource allocation, local priorities).
- Infra-structural system (i.e. political support).
- Country and rurality (i.e. small or large/rural vs regional vs remote).
- Socio-demographic characteristics:
- Funding context or source (i.e. free, personalised, group vs. individual, government funded).

- This study took place in the context of a professionalized CHW program implemented by the Migori County, Kenya Ministry of Health in partnership with Lwala Community Alliance (Lwala). The intervention works with existing cadres of government CHWs and incorporates practicing traditional birth attendants (TBAs) to create a new cadre of professionalized CHWs, who are then trained, paid, supervised, and equipped to complete their work.

**Table 2 – Mechanisms**

- **“An element of reasoning and/or reactions of an individual or collective agents(s) in regard of resources available in a given context to bring about changes through the implementation of an intervention” (4). Check what are the mediators to produce outcomes (not only primary, but also secondary or unintended outcomes).**

**There were no specific mechanisms mentioned in the study, however:**

- Training through the intervention was predictive of increased CHW performance, including improved immunization rates and antenatal care completion rates.
- Recency of training and experience caring for pregnant women were associated with increased CHW knowledge.
- Education and literacy showed tenuous associations with CHW competency.
- Gender was not significantly associated with CHW competency.
- Payment, frequent supervision, and continuous training were associated with increased CHW performance.
- Community selection played a crucial role in CHW performance (“The existing cadres of government CHWs were originally selected through a participatory process where community members are proposed and endorsed to become CHWs.”)

**Table 3 – Outcomes**

- **Please make note of all (intended or not) outcomes. Please also classify the role and functions outcomes of CHWs using the following Pillars for people-centred care:**

1. Engaging and empowering people and communities

2. Strengthening governance and accountability (Incl. Supervision, training, and support)

3. Reorienting the model of care

4. Coordinating services within and across sectors

5. Enabling environment

- The intervention aimed to professionalize Community Health Worker (CHW) cadres through enhanced training, payment, and supervision.
- Trained CHWs were 15% more likely to have fully immunized clients and 14% more likely to have clients who completed four or more antenatal care visits.
- Recency of training and experience caring for pregnant women were associated with increased CHW knowledge.
- There was no association between gender and CHW knowledge or performance.
- The link between education and literacy and CHW performance and knowledge was inconsistent, suggesting that robust training and supervision may compensate for lower education or literacy levels.
- Further research is recommended to investigate the influence of other program components, such as payment, supervision, and refresher training, on CHW performance.
- CHWs in the intervention showed significant increases in knowledge scores across all domains, particularly in pregnancy-related knowledge.
- The intervention's training was significantly associated with CHW performance, particularly in immunization and antenatal care.
- Overall, the intervention demonstrated positive outcomes in terms of CHW performance and knowledge, with training and experience playing significant roles. The findings suggest that education and literacy may not be strong predictors of CHW competency, highlighting the importance of robust training and supervision in CHW programs. Further research is recommended to explore the influence of other program components on CHW performance.

Outcomes by PCC pillars:

1. **Engaging and empowering people and communities**:
   - The intervention aimed to professionalize CHW cadres through enhanced training, payment, and supervision, which led to increased CHW performance and improved health outcomes.
   - Community acceptability and experience caregiving were important factors in CHW selection, highlighting the importance of engaging and empowering the community in the CHW program.
2. **Strengthening governance and accountability (Incl. Supervision, training, and support)**:
   - The intervention included frequent training and supervision, which were associated with increased CHW performance.
   - Payment and supportive supervision were important program components that may have influenced CHW knowledge and performance.
   - The intervention's training was significantly associated with CHW performance, suggesting the importance of training in strengthening governance and accountability.
3. **Reorienting the model of care**:
   - The intervention focused on providing support to pregnant women for antenatal care visits and delivery with a skilled provider, as well as tracking the health of infants and children for early diagnosis and treatment, and on-time immunizations.
   - The intervention led to increased CHW knowledge of obstetric and early infant danger signs, indicating a reorientation of care towards maternal and child health.
4. **Coordinating services within and across sectors**:
   - The intervention was implemented jointly by Lwala Community Alliance and the Kenya Ministry of Health, highlighting the coordination between different sectors in the implementation of the program.
   - CHWs were responsible for enrolling households in the community and providing preventative and curative services, as well as referring clients to government health facilities for care, indicating coordination between CHWs and the formal healthcare system.
5. **Enabling environment**:
   - The intervention aimed to professionalize CHWs through enhanced training, payment, and supervision, creating an enabling environment for CHWs to perform effectively.
   - The study recommends further research into intervention design choices, including payment, supervision, and refresher training, to create an enabling environment for CHW performance.

**Table 4 – If, then statements depicting the CMOs.**

**Please detail the CMOs for the study you reviewed using the If (Context), Then (Mechanism) statements.**

**If, Then statements**

- If CHWs receive fair remuneration, frequent supervision, and continuous training, they feel motivated and competent in executing their tasks (fair remuneration acknowledges their value, while frequent supervision offers support and empowerment, and continuous training enhances their knowledge and skills). This leads to increased job satisfaction, higher retention rates, and enhanced quality of services provided.
- If CHWs are selected through a participatory process (i.e. selected through a participatory process where community members are proposed and endorsed to become CHWs), then this process helps in identifying individuals who are likely to be accepted and respected within the community, fostering trust, cultural competence and understanding of local health needs among the chosen candidates, leading to increased effectiveness in healthcare delivery, better acceptance of health interventions, and improved health outcomes within the community.

**Test for relevance (Pearson et al. 2012; 2015; Brennan et al. 2017)**

| **Conceptually Rich** | **Thicker description’ but not ‘conceptually rich’** | **Conceptually Thin** |
| --- | --- | --- |
| Unambiguous theoretical concepts are described in sufficient depth. | Description of programme theory or sufficient information to enable it to ‘surface’. | Insufficient information to enable the programme theory to surface. |
| Relationships between, amongst concepts are clearly articulated. | Consideration of the context in which the programme takes place. | Limited or no consideration of the context in which the programme took place. |
| Concepts are sufficiently developed, defined to enable understanding without the reader needing to have first-hand experience of an area of practice. | Discussion of the differences between the design and orientation of programme theory (what was intended) and implementation (what really happened). | Limited or no discussion of the differences between the design and orientation of programme theory (what was intended) and implementation (what really happened). |
| Concepts are grounded strongly in a cited body of literature. | Recognition and discussion of the strengths/weaknesses of the implemented programme. | Limited or no discussion of the strengths/ weaknesses of the implemented programme. |
| Concepts are parsimonious (i.e., provide the simplest, but not over-simplified, explanation) | Some attempt to explain anomalous results and findings with reference to context and data. | No attempts to explain anomalous results and findings with reference to context and data. |
|  | Description of the factor affecting implementation. | Limited or no description of the factors affecting implementation. |

**Test for relevance (Pearson et al. 2012; 2015; Brennan et al. 2017)**

| **Conceptually Rich** | **Thicker description’ but not ‘conceptually rich’** | **Conceptually Thin** |
| --- | --- | --- |
|  | X |  |

**Test for rigour (Ohly et al. 2017)**

|  | **Yes** | **Fairly** | **No** |
| --- | --- | --- | --- |
| The study methods are clearly reported. | X |  |  |
| The study methods are appropriate to answer RQ. | X |  |  |
| The sample characteristics enable generalizability. |  |  | X |
| Raw data supports the study findings (conclusions). | X |  |  |
| Limitations of the study are acknowledged and clearly reported. | X |  |  |

**References**

1. Wong G, Westhorp G, Manzano A, Greenhalgh J, Jagosh J, Greenhalgh T. (2016). RAMESES II

reporting standards for realist evaluations. *BMC Medicine*, 14(1), 1-18.

2. Wong G, Greenhalgh T, Westhorp G, Pawson R. (2012). Realist methods in medical education

research: what are they and what can they contribute? *Medical Education*, 46(1), 89-96.

3. Macfarlane F, Greenhalgh T, Humphrey C, Hughes J, Butler C, Pawson R. (2011). A new workforce

in the making?: A case study of strategic human resource management in a whole-system change

effort in healthcare. *Journal of Health Organization and Management*, 25(1), 55-72.

4. Lacouture A, Breton E, Guichard A, Ridde V. (2015). The concept of mechanism from a realist

approach: a scoping review to facilitate its operationalization in public health program evaluation.

*Implementation Science*, 10(1), 1-10.

5. Pearson M, Chilton R, Woods HB, Wyatt K, Ford T, Abraham C, et al. Implementing health promotion in schools: protocol for a realist systematic review of research and experience in the United Kingdom (UK). Syst Rev. 2012 Oct 20;1(1):48.

6. Pearson M, Brand SL, Quinn C, Shaw J, Maguire M, Michie S, et al. Using realist review to inform intervention development: methodological illustration and conceptual platform for collaborative care in offender mental health. Implementation Sci. 2015 Sep 28;10(1):134.

7. Brennan N, Bryce M, Pearson M, Wong G, Cooper C, Archer J. Towards an understanding of how appraisal of doctors produces its effects: a realist review. Medical Education. 2017;51(10):1002–13.

8. Ohly H, Crossland N, Dykes F, Lowe N, Hall-Moran V. A realist review to explore how low-income pregnant women use food vouchers from the UK’s Healthy Start programme. BMJ Open. 2017 Apr 21;7(4):e013731.

**A realist evaluation of the role and functions of community health workers in service of a people-centred community health system.**

**Investigator CMOc Data Extraction Sheet Data Extraction**

The purpose of this document is to extract data from the identified studies in order to formulate one or more explanatory theories that explain Context-Mechanism-Outcome Configurations (CMOC) and to gain insight into how these CMOCs contribute to optimizing the role and functions of community health workers within a people-centred community health system.

In realist methodologies, knowledge accumulation centres on our comprehension of how mechanisms behave in various contexts and the reasons behind how and why this interplay of context and mechanism leads to different outcomes (1). Please fill in the details below for the study you will be assessing. In the subsequent three tables, locate and specify the Context, Mechanism, and Outcomes (CMO) of the intervention discussed in the study you've examined. Definitions and illustrations of CMO are provided within the tables.

**Kindly fill out the information below for the study you will be reviewing.**

| **Reviewer Name** | Usangiphile Buthelezi |
| --- | --- |
| **Reference** | Mulubwa C, Hurtig AK, Zulu JM, Michelo C, Sandøy IF, Goicolea I. Can sexual health interventions make community-based health systems more responsive to adolescents? A realist informed study in rural Zambia. Reprod Health. 2020 Jan 8;17(1):1. |
| **Country** | Zambia |
| **Setting and population** | - The study was conducted in rural Zambia as part of the Research Initiative to Support the Empowerment of Girls (RISE) project. - The intervention targeted adolescents in community-based health systems (CBHS) in Zambia. - The participants included stakeholders involved in the development and/or implementation of the RISE trial, such as researchers, community-based health workers (CBHWs), and teachers. - The RISE project enrolled girls who were in grade 7, with an average age of approximately 14 years. - The study focused on the full intervention arm of RISE, referred to as the 'combined intervention arm', which involved 157 schools and targeted approximately 4900 girls. |
| **Aim** | - The aim of the intervention was to address the sexual reproductive health and rights (SRHR) needs of adolescents in community-based health systems (CBHS) in rural Zambia. |
| **Objectives** | - To transform the CBHS into systems that are responsive to the SRHR needs of adolescents. - The intervention aimed to create safe spaces for parents, community members, and adolescents to interact and address SRHR issues collectively. |
| **Methodology** | - The study adopted a realist evaluation approach to explore how and why community-based sexual reproductive health interventions can transform community-based health systems (CBHS) to be responsive to the sexual reproductive health needs of adolescents. - A case study design was used, nested within the full intervention arm of the Research Initiative to Support the Empowerment of Girls (RISE) trial in Zambia. - Sixteen in-depth interviews were conducted with stakeholders involved in the development and/or implementation of the RISE trial. - The interviews were recorded and analyzed using NVIVO version 12.0, and thematic analysis was conducted guided by realist evaluation concepts. - The findings were synthesized using the Intervention-Context-Actors-Mechanism-Outcomes (ICAMOs) conceptualization tool. - The researchers developed two initial testable program theories based on the findings, exploring the role of contextual factors and individual and collective mechanisms in transforming CBHS. |
| **Intervention description** | - The study focuses on community-based sexual reproductive health interventions for adolescents in rural Zambia. - The interventions aim to transform ordinary community-based health systems (CBHSs) into systems that are responsive to the sexual reproductive health needs of adolescents. - The interventions are supported by existing national policies, socio-cultural norms, and CBHS structures. - The interventions involve improving awareness and knowledge among individuals, clarifying values on sexual reproductive health and rights (SRHR), and promoting communication, cohesion, social connections, and linkages among stakeholders. - The interventions are expected to encourage uptake of SRHR services and information among adolescents. - The combined intervention arm of the Research Initiative to Support the Empowerment of Girls trial in Zambia is implemented by key stakeholders, including the Ministry of Health, trial supervisors, teachers, and community-based health workers. - The interventions aim to address the prevailing negative environment at health facilities and transform CBHSs into systems that support SRHR services for adolescents. |
| **Intervention outcomes** | - The study identified two initial testable program theories for community-based sexual reproductive health interventions in rural Zambia. - The first theory suggests that interventions supported by contextual factors such as existing policies, socio-cultural norms, and CBHS structures are more likely to trigger mechanisms among different actors, leading to the transformation of CBHSs and responsiveness to the sexual reproductive health needs of adolescents. - The second theory proposes that interventions not supported by contextual factors are less likely to transform CBHSs. - The study also highlighted the importance of contextual factors in promoting or hindering the transformation of CBHSs into systems that are responsive to adolescents' sexual reproductive health needs. - The expected outcomes of the interventions include improved access to sexual reproductive health services and information for adolescents, increased awareness and knowledge, value clarification, communication, cohesion, social connection, and linkages among stakeholders. - Implementing the interventions in line with existing policies can strengthen national policies and laws related to sexual reproductive health. |
| **Challenges and limitations** | - Program theories developed using data from stakeholders can become "messy" and unstructured, making it challenging to relate to different social strata. - The study acknowledges that stakeholders may have found it challenging to shift their focus from the planned outcomes of the intervention to the abstract level of how the sexual reproductive health interventions could transform the community-based health systems (CBHSs). - The study also mentions the limitation of social desirability bias, which was minimized by emphasizing that the evaluation was not focused on the specific intervention but rather on how interventions like the one studied can transform CBHSs. - The study was conducted in rural Zambia, which may limit the generalizability of the findings to other settings |

**Table 1 – Context**

**Including (but not limited to) beliefs, social and cultural norms, regulations and economic factors (2,3). For example:**

- Individual capabilities (i.e. values, roles, knowledge, purpose).
- Interpersonal relationships supporting the interventions (i.e. communication, collaboration, network, influences).
- Institutional settings (i.e. informal rules, organisational culture, leadership, policies, resource allocation, local priorities).
- Infra-structural system (i.e. political support).
- Country and rurality (i.e. small or large/rural vs regional vs remote).
- Socio-demographic characteristics:
- Funding context or source (i.e. free, personalised, group vs. individual, government funded).

- Existing policies and guidelines related to sexual reproductive health and rights (SRHR).
- Socio-cultural norms and beliefs surrounding adolescent sexual activities and SRHR.
- Community-based health systems (CBHSs) in rural areas with low socio-economic status.
- Poverty, high rates of adolescent pregnancies, early marriages, and school dropouts in the selected communities.
- Limited access to and adoption of SRHR information due to social-cultural norms and religious beliefs

**Table 2 – Mechanisms**

- **“An element of reasoning and/or reactions of an individual or collective agents(s) in regard of resources available in a given context to bring about changes through the implementation of an intervention” (4). Check what are the mediators to produce outcomes (not only primary, but also secondary or unintended outcomes).**

- Individual mechanisms, such as awareness and knowledge, contribute to the development of a supportive attitude towards adolescent sexual reproductive health and rights (SRHR).
- Increased awareness and knowledge among parents and adolescents can lead to a better understanding of SRHR issues and the importance of addressing them, such as avoiding early pregnancies and marriages.
- These individual mechanisms can trigger collective mechanisms within the community-based health systems (CBHSs), such as communication, cohesion, social connection, and linkages, which further contribute to the responsiveness of the CBHSs to adolescent SRHR needs.
- The development of supportive attitudes and increased awareness among individuals can also lead to improved collaboration between parents, teachers, and healthcare workers, creating a conducive environment for addressing adolescent SRHR issues.
- Increased awareness and knowledge among adolescents and parents contribute to a better understanding of sexual reproductive health and rights (SRHR) issues and the importance of addressing them.
- Value clarification, which involves understanding and letting go of societal norms that negatively affect adolescents' SRHR, can lead to a supportive attitude towards adolescent SRHR.
- Communication and cohesion within the community-based health systems (CBHSs) facilitate the sharing of information and support for seeking SRHR services.
- Social connection and linkages among adolescents, community-based health workers (CBHWs), and other individuals in the CBHS enhance collaboration and access to SRHR services.
- Trust and rapport between parents, teachers, and healthcare workers create a conducive environment for addressing adolescent SRHR needs.
- Recognition and satisfaction with the interventions and services provided contribute to the overall responsiveness of the CBHSs to adolescent SRHR.

**Table 3 – Outcomes**

- **Please make note of all (intended or not) outcomes. Please also classify the role and functions outcomes of CHWs using the following Pillars for people-centred care:**

1. Engaging and empowering people and communities

2. Strengthening governance and accountability (Incl. Supervision, training, and support)

3. Reorienting the model of care

4. Coordinating services within and across sectors

5. Enabling environment

- The study identified two initial testable program theories for community-based sexual reproductive health interventions in rural Zambia.
- The first theory suggests that interventions supported by contextual factors such as existing policies, socio-cultural norms, and CBHS structures are more likely to trigger mechanisms among different actors, leading to the transformation of CBHSs and responsiveness to the sexual reproductive health needs of adolescents.
- The second theory proposes that interventions not supported by contextual factors are less likely to transform CBHSs.
- The study also highlighted the importance of contextual factors in promoting or hindering the transformation of CBHSs into systems that are responsive to adolescents' sexual reproductive health needs.
- The expected outcomes of the interventions include improved access to sexual reproductive health services and information for adolescents, increased awareness and knowledge, value clarification, communication, cohesion, social connection, and linkages among stakeholders.
- Implementing the interventions in line with existing policies can strengthen national policies and laws related to sexual reproductive health**.**

**Outcomes according to PCC pillars:**

1. **Engaging and empowering people and communities:**
   - Community-based sexual reproductive health (SRH) interventions can empower adolescents by providing them with knowledge, clarifying misconceptions, and improving their ability to make informed decisions about SRH.
   - Engaging adolescents in youth clubs and other activities can empower them to communicate effectively about SRH with their partners and negotiate for their needs.
2. **Strengthening governance and accountability (Incl. Supervision, training, and support):**
   - The presence of policies and guidelines related to SRH, such as comprehensive sexuality education guidelines, can provide an enabling environment for SRH interventions and contribute to the responsiveness of community-based health systems (CBHSs).
   - Adequate supervision, training, and support for stakeholders involved in the implementation of SRH interventions can enhance the effectiveness and quality of services provided.
3. **Reorienting the model of care:**
   - Community-based SRH interventions aim to transform CBHSs into systems that are responsive to the SRH needs of adolescents.
   - By providing SRH information, linkages to health facilities, and access to contraceptives, these interventions aim to reorient the model of care towards addressing the specific needs of adolescents.
4. **Coordinating services within and across sectors:**
   - Effective communication, cohesion, and social connection within CBHSs can facilitate coordination of SRH services for adolescents.
   - Collaboration between different stakeholders, including the Ministry of Health and Ministry of Education, can ensure alignment and coordination of SRH interventions and policies.
5. **Enabling environment:**
   - The presence of contextual factors, such as existing policies, socio-cultural norms, and CBHS structures, can support the effectiveness of SRH interventions and contribute to the responsiveness of CBHSs to adolescent SRH needs.

**Table 4 – Intervention-Context-Actors-Mechanism-Outcome Configurations (ICAMOc)**

**Please detail the CMOs for the study you reviewed using the If (Context), Then (Mechanism) statements.**

**If, Then statements**

- IF interventions that aim to address the SRHR issues of adolescents in the community-based health systems (CBHS) are supported by existing national policies, socio-cultural norms, existing CBHS structures and functioning local health facilities THEN the interventions are likely to make all key stakeholders in the CBHS develop a positive attitude towards adolescents’ SRHR, which will encourage uptake of SRHR services and information BECAUSE, at individual level, the intervention will improve awareness and knowledge, clarify the values on SRHR among all the actors (adolescents, CBHWs, teachers and community members), which will at collective level lead to improved communication and cohesion, social connections and linkages among the actors. AS A RESULT, the ‘ordinary’ CBHSs will be transformed.

**Test for relevance (Pearson et al. 2012; 2015; Brennan et al. 2017)**

| **Conceptually Rich** | **Thicker description’ but not ‘conceptually rich’** | **Conceptually Thin** |
| --- | --- | --- |
| Unambiguous theoretical concepts are described in sufficient depth. | Description of programme theory or sufficient information to enable it to ‘surface’. | Insufficient information to enable the programme theory to surface. |
| Relationships between, amongst concepts are clearly articulated. | Consideration of the context in which the programme takes place. | Limited or no consideration of the context in which the programme took place. |
| Concepts are sufficiently developed, defined to enable understanding without the reader needing to have first-hand experience of an area of practice. | Discussion of the differences between the design and orientation of programme theory (what was intended) and implementation (what really happened). | Limited or no discussion of the differences between the design and orientation of programme theory (what was intended) and implementation (what really happened). |
| Concepts are grounded strongly in a cited body of literature. | Recognition and discussion of the strengths/weaknesses of the implemented programme. | Limited or no discussion of the strengths/ weaknesses of the implemented programme. |
| Concepts are parsimonious (i.e., provide the simplest, but not over-simplified, explanation) | Some attempt to explain anomalous results and findings with reference to context and data. | No attempts to explain anomalous results and findings with reference to context and data. |
|  | Description of the factor affecting implementation. | Limited or no description of the factors affecting implementation. |

**Test for relevance (Pearson et al. 2012; 2015; Brennan et al. 2017)**

| **Conceptually Rich** | **Thicker description’ but not ‘conceptually rich’** | **Conceptually Thin** |
| --- | --- | --- |
| X |  |  |

**Test for rigour (Ohly et al. 2017)**

|  | **Yes** | **Fairly** | **No** |
| --- | --- | --- | --- |
| The study methods are clearly reported. | X |  |  |
| The study methods are appropriate to answer RQ. | X |  |  |
| The sample characteristics enable generalizability. |  |  | X |
| Raw data supports the study findings (conclusions). | X |  |  |
| Limitations of the study are acknowledged and clearly reported. | X |  |  |

**References**

1. Wong G, Westhorp G, Manzano A, Greenhalgh J, Jagosh J, Greenhalgh T. (2016). RAMESES II

reporting standards for realist evaluations. *BMC Medicine*, 14(1), 1-18.

2. Wong G, Greenhalgh T, Westhorp G, Pawson R. (2012). Realist methods in medical education

research: what are they and what can they contribute? *Medical Education*, 46(1), 89-96.

3. Macfarlane F, Greenhalgh T, Humphrey C, Hughes J, Butler C, Pawson R. (2011). A new workforce

in the making?: A case study of strategic human resource management in a whole-system change

effort in healthcare. *Journal of Health Organization and Management*, 25(1), 55-72.

4. Lacouture A, Breton E, Guichard A, Ridde V. (2015). The concept of mechanism from a realist

approach: a scoping review to facilitate its operationalization in public health program evaluation.

*Implementation Science*, 10(1), 1-10.

5. Pearson M, Chilton R, Woods HB, Wyatt K, Ford T, Abraham C, et al. Implementing health promotion in schools: protocol for a realist systematic review of research and experience in the United Kingdom (UK). Syst Rev. 2012 Oct 20;1(1):48.

6. Pearson M, Brand SL, Quinn C, Shaw J, Maguire M, Michie S, et al. Using realist review to inform intervention development: methodological illustration and conceptual platform for collaborative care in offender mental health. Implementation Sci. 2015 Sep 28;10(1):134.

7. Brennan N, Bryce M, Pearson M, Wong G, Cooper C, Archer J. Towards an understanding of how appraisal of doctors produces its effects: a realist review. Medical Education. 2017;51(10):1002–13.

8. Ohly H, Crossland N, Dykes F, Lowe N, Hall-Moran V. A realist review to explore how low-income pregnant women use food vouchers from the UK’s Healthy Start programme. BMJ Open. 2017 Apr 21;7(4):e013731.

**A realist evaluation of the role and functions of community health workers in service of a people-centred community health system.**

**Investigator CMOc Data Extraction Sheet Data Extraction**

The purpose of this document is to extract data from the identified studies in order to formulate one or more explanatory theories that explain Context-Mechanism-Outcome Configurations (CMOC) and to gain insight into how these CMOCs contribute to optimizing the role and functions of community health workers within a people-centred community health system.

In realist methodologies, knowledge accumulation centres on our comprehension of how mechanisms behave in various contexts and the reasons behind how and why this interplay of context and mechanism leads to different outcomes (1). Please fill in the details below for the study you will be assessing. In the subsequent three tables, locate and specify the Context, Mechanism, and Outcomes (CMO) of the intervention discussed in the study you've examined. Definitions and illustrations of CMO are provided within the tables.

**Kindly fill out the information below for the study you will be reviewing.**

| **Reviewer Name** | Usangiphile Buthelezi |
| --- | --- |
| **Reference** | Mendin SF, Krause JA, Gweh A, Baysah M, Nyumah J, Gaye CJ, et al. Measuring health system responsiveness in a national community health worker primary care programme in rural Liberia. Int J Qual Health Care. 2023 May 17;35(2): mzad027. |
| **Country** | Liberia |
| **Setting and population** | - The study was conducted in Rivercess (RC) and Grand Gedeh (GG) counties in Liberia, where the national Community Health Assistants (CHAs) programme has been implemented. - These counties are characterized by poor road connectivity, high poverty rates, and isolated communities. - The population in Rivercess county was 71,509, and in Grand Gedeh county, it was 140,934, according to the 2008 national and household census. - The study included women aged 18-49 years who sought care from a CHA in the 3 months prior to the survey. - The household survey used a two-stage cross-sectional cluster sampling approach, with communities as the primary sample units and households as the secondary sample units. - The total number of households surveyed was 2,111, with an almost equal number of households from each county |
| **Aim** | - The aim of the study was to measure health system responsiveness (HSR) and the quality of care delivered by Community Health Assistants (CHAs) in the national community health worker (CHW) programme in rural Liberia |
| **Objectives** | - The study aimed to assess patient-reported health system outcomes, including satisfaction, trust, and confidence in the CHA, and determine the association between responsiveness and these outcomes. - The study aimed to fill the gap in measuring patient experience and outcomes of care in the context of CHW-delivered care, as this domain of quality is important for the design and delivery of community health programs. - The study aimed to inform more comprehensive measurement and improvement of quality CHW services in Liberia and similar contexts globally, to ensure people-centered primary care and achieve quality universal health coverage. |
| **Methodology** | - The study used a cross-sectional population-based household survey conducted in Rivercess (RC) and Grand Gedeh (GG) counties in Liberia. - The survey employed a two-stage cross-sectional cluster sampling approach, with communities as the primary sample units and households as the secondary sample units. - The study included women aged 18-49 years who reported seeking care from a Community Health Assistant (CHA) in the 3 months prior to the survey. - Validated Health System Responsiveness (HSR) questions were administered to the participants, covering six responsiveness domains and patient-reported health system outcomes such as satisfaction, trust, and confidence in the CHA's skills and abilities. - A composite responsiveness score was calculated and divided into tertiles to assess the association between responsiveness and patient-reported health system outcomes. - Multivariable analysis using Poisson regression with log link and adjusting for respondent characteristics was conducted to determine the association between responsiveness and patient-reported health system outcomes. - The study aimed to measure patients' experience and outcomes of care to complement measures of technical quality and ensure that this domain of quality is central to the design and delivery of the community health program. |
| **Intervention description** | - The national community health worker (CHW) programme in Liberia, known as the Community Health Assistants (CHAs) programme, was established in 2016. CHAs are recruited from their communities and trained to provide primary health care services in rural areas that are at least 5 km away from healthcare facilities. - Last Mile Health, a non-governmental organization, has provided support for the implementation of the CHA programme in Rivercess (RC), Grand Gedeh (GG), and Grand Bassa counties. - CHAs are permanent residents of their communities and are trained in modules covering various aspects of care, including home visits, data gathering, care and treatment for malaria, diarrhoea, and pneumonia, malnutrition screening, and referral services. - They are supervised twice a month, receive supplies and a monthly cash incentive, and are expected to work approximately 20 hours per week. - The CHA programme has been successful in expanding young child illness treatment coverage and quality of care in areas such as suspected malaria cases assessed with rapid diagnostic tests. - The programme aims to provide quality and equitable health services to remote communities, with a focus on ensuring patient-centered care and meeting the goals of universal health coverage. |
| **Intervention outcomes** | - The national community health worker (CHW) programme in Liberia has shown positive outcomes in terms of health system responsiveness (HSR) and patient-reported health system outcomes. - The study conducted in Rivercess (RC) and Grand Gedeh (GG) counties found that higher responsiveness scores were associated with better patient-reported health system outcomes, including satisfaction, trust, and confidence in the CHA. - Women in the highest tertile of responsiveness scores were significantly more likely to report high quality of CHA-delivered care, meeting health needs, high confidence in the CHA, and a high level of trust in CHA's skills and abilities. - Age, wealth, and education were not significantly associated with patient-reported health system outcomes, indicating that the CHA programme's benefits were not limited to specific demographic groups. - The CHA programme aims to provide people-centered primary care necessary to help countries achieve quality universal health coverage. - Overall, the national CHA programme in Liberia has demonstrated positive outcomes in terms of patient-reported health system outcomes, indicating that the programme is effective in delivering quality and patient-centered care in rural areas. |
| **Challenges and limitations** | - Limited reporting of Health System Responsiveness (HSR) measurement in the literature for Community Health Worker (CHW)-delivered care. - Similar studies conducted in Uganda, Liberia, Burkina Faso, and Ghana also highlighted the need for improvement in responsiveness of CHW-delivered care. - Potential social desirability bias due to close-knit communities and potential recall bias as questions were asked up to 3 months since the visit. - The cross-sectional nature of the survey limited direct causal inference. - Technical quality was not assessed for the visits for which experiential quality was asked. - The study did not include men or women of other ages in the study population, limiting the generalizability of the findings. |

**Table 1 – Context**

**Including (but not limited to) beliefs, social and cultural norms, regulations and economic factors (2,3). For example:**

- Individual capabilities (i.e. values, roles, knowledge, purpose).
- Interpersonal relationships supporting the interventions (i.e. communication, collaboration, network, influences).
- Institutional settings (i.e. informal rules, organisational culture, leadership, policies, resource allocation, local priorities).
- Infra-structural system (i.e. political support).
- Country and rurality (i.e. small or large/rural vs regional vs remote).
- Socio-demographic characteristics:
- Funding context or source (i.e. free, personalised, group vs. individual, government funded).

- **Country:** The study was conducted in Liberia, a low-income country in West Africa
- Health System Organization: The study focused on the national community health worker (CHW) program in Liberia, specifically the Community Health Assistants (CHAs) who provide primary health care services in rural communities.
- **Rurality:** The study was conducted in rural areas of Liberia, specifically in Rivercess (RC) and Grand Gedeh (GG) counties, which are characterized by poor road connectivity and high poverty rates
- **Socio-demographic and Socio-economic status:** The study included women aged 18-49 years who sought care from CHAs in the 3 months prior to the survey. Respondent characteristics such as age, education, wealth quintile, and distance to the nearest health facility were considered.
- **Population and Setting:** The study population consisted of individuals living in rural communities 5 km from any healthcare facility. These communities were isolated and relied on farming, hunting, palm oil production, and gold mining as their main sources of income.
- **Access to Resources:** The study aimed to assess the quality of CHA-delivered care and health system responsiveness in areas with limited access to healthcare facilities. The CHAs were trained, supervised, salaried, and supplied with the necessary resources to provide care in these communities.
- **Psychosocial:** The study measured patient-reported health system outcomes, including satisfaction, trust, and confidence in the CHAs' skills and abilities, to assess the psychosocial aspects of care delivery.

**Table 2 – Mechanisms**

- **“An element of reasoning and/or reactions of an individual or collective agents(s) in regard of resources available in a given context to bring about changes through the implementation of an intervention” (4). Check what are the mediators to produce outcomes (not only primary, but also secondary or unintended outcomes).**

- Trust and confidence in CHAs' abilities were high due to their selection from the community and their training, which promoted positive patient perceptions and behavior change practices.
- Higher responsiveness scores in CHW-delivered care were associated with better patient-reported health system outcomes, including satisfaction, overall quality of care, and trust in CHA's skills and abilities.
- Proximity of CHWs to individuals in need of care engendered greater trust and confidence, contributing to positive patient perceptions and outcomes.
- Measuring patients' experience and outcomes of care was important to ensure that health system responsiveness was central to the design and delivery of community health programs. This included measuring satisfaction, trust, and confidence in the CHA.

**Table 3 – Outcomes**

- **Please make note of all (intended or not) outcomes. Please also classify the role and functions outcomes of CHWs using the following Pillars for people-centred care:**

1. Engaging and empowering people and communities

2. Strengthening governance and accountability (Incl. Supervision, training, and support)

3. Reorienting the model of care

4. Coordinating services within and across sectors

5. Enabling environment

- Higher health system responsiveness (HSR) scores were associated with important patient-reported health system quality outcomes, including satisfaction, trust, and confidence in the CHA.
- Women in the highest tertile of responsiveness scores were significantly more likely to report high quality of CHA-delivered care, meeting health needs, high confidence in the CHA to provide future care, and a high level of trust in CHA's skills and abilities.
- Trust and confidence in CHAs' abilities were high, contributing to positive patient perceptions and behavior change practices.
- Proximity of CHWs to individuals in need of care engendered greater trust and confidence, promoting positive patient perceptions and outcomes.
- Measuring patients' experience and outcomes of care is important to ensure that health system responsiveness is central to the design and delivery of community health programs.

**Outcomes according to PCC pillars:**

- **Engaging and empowering people and communities:** The study found that higher health system responsiveness (HSR) scores were associated with better patient-reported outcomes, including satisfaction, trust, and confidence in the CHA. This suggests that engaging and empowering individuals in their own healthcare decisions and involving them in the community health worker (CHW) program can lead to positive outcomes.
- **Strengthening governance and accountability:** The study did not specifically address the outcomes related to strengthening governance and accountability, such as supervision, training, and support. Therefore, there is no direct evidence from the study regarding the impact of these factors on the outcomes.
- **Reorienting the model of care:** The study emphasized the importance of measuring patients' experience and outcomes of care to ensure that health system responsiveness is central to the design and delivery of community health programs. This highlights the need to reorient the model of care towards patient-centeredness and quality of care.
- **Coordinating services within and across sectors:** The study did not directly address the outcomes related to coordinating services within and across sectors. However, the findings suggest that a well-implemented CHW program can contribute to improved coordination and integration of services, as CHAs were trusted and seen as capable of providing future care.
- **Enabling environment:** The study did not specifically discuss the outcomes related to the enabling environment. However, the findings highlight the importance of ensuring an enabling environment for CHW-delivered care, including factors such as proximity to health facilities and community trust, which were associated with positive patient perceptions and outcomes.

**Table 4 – If, then statements depicting the CMOs.**

**Please detail the CMOs for the study you reviewed using the If (Context), Then (Mechanism) statements.**

**If, Then statements**

- If CHWs are selected within the community, then the community will have positive perceptions towards them because their familiarity with local customs, language, and culture fosters a sense of trust, leading to better acceptance of healthcare messages and services, resulting in behavior change.

**Test for relevance (Pearson et al. 2012; 2015; Brennan et al. 2017)**

| **Conceptually Rich** | **Thicker description’ but not ‘conceptually rich’** | **Conceptually Thin** |
| --- | --- | --- |
| Unambiguous theoretical concepts are described in sufficient depth. | Description of programme theory or sufficient information to enable it to ‘surface’. | Insufficient information to enable the programme theory to surface. |
| Relationships between, amongst concepts are clearly articulated. | Consideration of the context in which the programme takes place. | Limited or no consideration of the context in which the programme took place. |
| Concepts are sufficiently developed, defined to enable understanding without the reader needing to have first-hand experience of an area of practice. | Discussion of the differences between the design and orientation of programme theory (what was intended) and implementation (what really happened). | Limited or no discussion of the differences between the design and orientation of programme theory (what was intended) and implementation (what really happened). |
| Concepts are grounded strongly in a cited body of literature. | Recognition and discussion of the strengths/weaknesses of the implemented programme. | Limited or no discussion of the strengths/ weaknesses of the implemented programme. |
| Concepts are parsimonious (i.e., provide the simplest, but not over-simplified, explanation) | Some attempt to explain anomalous results and findings with reference to context and data. | No attempts to explain anomalous results and findings with reference to context and data. |
|  | Description of the factor affecting implementation. | Limited or no description of the factors affecting implementation. |

**Test for relevance (Pearson et al. 2012; 2015; Brennan et al. 2017)**

| **Conceptually Rich** | **Thicker description’ but not ‘conceptually rich’** | **Conceptually Thin** |
| --- | --- | --- |
| X |  |  |

**Test for rigour (Ohly et al. 2017)**

|  | **Yes** | **Fairly** | **No** |
| --- | --- | --- | --- |
| The study methods are clearly reported. | X |  |  |
| The study methods are appropriate to answer RQ. |  | X |  |
| The sample characteristics enable generalizability. |  | X |  |
| Raw data supports the study findings (conclusions). |  | X |  |
| Limitations of the study are acknowledged and clearly reported. | X |  |  |

**References**

1. Wong G, Westhorp G, Manzano A, Greenhalgh J, Jagosh J, Greenhalgh T. (2016). RAMESES II

reporting standards for realist evaluations. *BMC Medicine*, 14(1), 1-18.

2. Wong G, Greenhalgh T, Westhorp G, Pawson R. (2012). Realist methods in medical education

research: what are they and what can they contribute? *Medical Education*, 46(1), 89-96.

3. Macfarlane F, Greenhalgh T, Humphrey C, Hughes J, Butler C, Pawson R. (2011). A new workforce

in the making?: A case study of strategic human resource management in a whole-system change

effort in healthcare. *Journal of Health Organization and Management*, 25(1), 55-72.

4. Lacouture A, Breton E, Guichard A, Ridde V. (2015). The concept of mechanism from a realist

approach: a scoping review to facilitate its operationalization in public health program evaluation.

*Implementation Science*, 10(1), 1-10.

5. Pearson M, Chilton R, Woods HB, Wyatt K, Ford T, Abraham C, et al. Implementing health promotion in schools: protocol for a realist systematic review of research and experience in the United Kingdom (UK). Syst Rev. 2012 Oct 20;1(1):48.

6. Pearson M, Brand SL, Quinn C, Shaw J, Maguire M, Michie S, et al. Using realist review to inform intervention development: methodological illustration and conceptual platform for collaborative care in offender mental health. Implementation Sci. 2015 Sep 28;10(1):134.

7. Brennan N, Bryce M, Pearson M, Wong G, Cooper C, Archer J. Towards an understanding of how appraisal of doctors produces its effects: a realist review. Medical Education. 2017;51(10):1002–13.

8. Ohly H, Crossland N, Dykes F, Lowe N, Hall-Moran V. A realist review to explore how low-income pregnant women use food vouchers from the UK’s Healthy Start programme. BMJ Open. 2017 Apr 21;7(4):e013731.

**A realist evaluation of the role and functions of community health workers in service of a people-centred community health system.**

**Investigator CMOc Data Extraction Sheet Data Extraction**

The purpose of this document is to extract data from the identified studies in order to formulate one or more explanatory theories that explain Context-Mechanism-Outcome Configurations (CMOC) and to gain insight into how these CMOCs contribute to optimizing the role and functions of community health workers within a people-centred community health system.

In realist methodologies, knowledge accumulation centres on our comprehension of how mechanisms behave in various contexts and the reasons behind how and why this interplay of context and mechanism leads to different outcomes (1). Please fill in the details below for the study you will be assessing. In the subsequent three tables, locate and specify the Context, Mechanism, and Outcomes (CMO) of the intervention discussed in the study you've examined. Definitions and illustrations of CMO are provided within the tables.

**Kindly fill out the information below for the study you will be reviewing.**

| **Reviewer Name** | Usangiphile Buthelezi |
| --- | --- |
| **Reference** | Murphy JP, Moolla A, Kgowedi S, Mongwenyana C, Mngadi S, Ngcobo N, et al. Community health worker models in South Africa: a qualitative study on policy implementation of the 2018/19 revised framework. Health Policy and Planning. 2021 May 1;36(4):384–96. |
| **Country** | South Africa |
| **Setting and population** | - The study was conducted in six provinces in South Africa, namely Gauteng, KwaZulu-Natal, Limpopo, Mpumalanga, Northwest, and Western Cape. - The participants included Department of Health Managers at the National, Provincial, and District level, healthcare providers, implementing partners (including NGOs), and Community Health Workers (CHWs). - The focus group discussions (FGDs) were conducted with 101 CHWs, providing insights from their perspective. - The study also involved in-depth interviews (IDIs) with healthcare providers, managers, and experts familiar with CHW work. - The districts and communities selected for the study were based on existing relationships and familiarity with the study sites. |
| **Aim** | - The study aimed to explore the CHW policy implementation landscape and barriers/facilitators to implementation in these provinces |
| **Objectives** | - Same as the aim |
| **Methodology** | - The study utilized a qualitative research design to explore the CHW policy implementation landscape in South Africa. - Data were collected through in-depth interviews (IDIs) with healthcare providers, managers, and experts familiar with CHW work, as well as focus group discussions (FGDs) with CHWs themselves. - A top-down non-random convenience sampling approach was followed, starting with IDIs with national and provincial experts, who then suggested participants at the district and facility levels. - The researchers analyzed the IDIs and FGDs to gain insights into the variations in implementation models, the involvement of NGOs, leadership structures, CHW working structure, and working hours |
| **Intervention description** | - The study focused on the implementation of the Community Health Worker (CHW) policy framework in South Africa. - The CHW policy aimed to establish Ward-Based Primary Healthcare Outreach Teams (WBPHCOT) as a means to bridge the gap between communities and healthcare service provision. - The study documented the current models of CHW policy implementation across six provinces in South Africa, including Gauteng, KwaZulu-Natal, Limpopo, Mpumalanga, Northwest, and Western Cape. - Data were collected through in-depth interviews (IDIs) with healthcare providers, managers, and experts familiar with CHW work, as well as focus group discussions (FGDs) with CHWs themselves. - The researchers explored the reasons for local adaptation of CHW models, identified barriers and facilitators to implementation, and gathered recommendations for improving the CHW program. - The study highlighted the variations in implementation models, the involvement of NGOs, leadership structures, CHW working structure, and working hours. |
| **Intervention outcomes** | - The study provided a detailed landscape of CHW policy implementation across several South African provinces, highlighting the variations in implementation models and the challenges that still exist. - The presence of an involved OTL was found to moderate the relationship between facility-based staff and CHWs, acting as a facilitator or barrier to implementation. - The study emphasized the need for permanent employment, uniforms, and higher wages for CHWs to ensure their visibility and recognition. - Mobile health solutions were recommended to help CHWs effectively plan their work and avoid wasting efforts on patients who are no longer within their communities. - The study identified the importance of better integrating CHWs into the current primary healthcare system to enhance collaboration and understanding between facility-based staff and CHWs. - CHWs demonstrated purpose and passion in their work, acting as a bridge between communities and healthcare facilities. - CHWs continued to promote health and link their communities to healthcare facilities despite challenges such as lack of permanent employment, limited resources, and low wages. - Effective communication and collaboration between CHWs and the community were reported to enhance the effectiveness of their work, particularly in tracing defaulters. - The presence of NGOs sometimes created confusion about work roles, while lack of communication between CHWs and healthcare facilities was a symptom of transitioning from NGO to Department of Health supervision. - The implementation of CHW policy varied across provinces, highlighting the adaptability required under resource constraints. - Clarity on elements of the policy framework, such as required qualifications of CHWs, was raised as an issue at the facility and community level . |
| **Challenges and limitations** | - Limited resources and competing priorities posed challenges to the implementation of the CHW policy framework in South Africa. - Incomplete staff complements, inconsistent use of data, safety concerns, and few pathways for professional development were identified as challenges to optimal implementation. - Insufficient numbers of CHWs, OTLs (Outreach Team Leaders), equipment, and supplies were frequently reported as major challenges. - Lack of respect and recognition from some community members and facility staff, low wages, absence of a uniform, burdensome paperwork, and limited opportunities for professional growth were consistently highlighted by CHWs. - Allocation of a full staff complement, and appropriate consideration of the catchment area size were reported as challenges. - Limited resources, including forms and stationery, were mentioned as barriers to policy implementation. |

**Table 1 – Context**

**Including (but not limited to) beliefs, social and cultural norms, regulations and economic factors (2,3). For example:**

- Individual capabilities (i.e. values, roles, knowledge, purpose).
- Interpersonal relationships supporting the interventions (i.e. communication, collaboration, network, influences).
- Institutional settings (i.e. informal rules, organisational culture, leadership, policies, resource allocation, local priorities).
- Infra-structural system (i.e. political support).
- Country and rurality (i.e. small or large/rural vs regional vs remote).
- Socio-demographic characteristics:
- Funding context or source (i.e. free, personalised, group vs. individual, government funded).

- **Health System Organization:** The study focused on the implementation of the Community Health Worker (CHW) policy framework in South Africa, specifically the Ward-Based Primary Healthcare Outreach Teams (WBPHCOT) that act as a bridge between communities and healthcare service provision.
- **Country and Rurality:** The study was conducted in six provinces in South Africa, including Gauteng, KwaZulu-Natal, Limpopo, Mpumalanga, North-West, and Western Cape.
- **Population and Setting:** The study involved Department of Health Managers, healthcare providers, implementing partners, and CHWs across various districts and provinces in South Africa.
- **Access to Resources:** The study highlighted challenges related to limited resources, including insufficient numbers of CHWs, OTLs, equipment, supplies, forms, and stationery.

**Table 2 – Mechanisms**

- **“An element of reasoning and/or reactions of an individual or collective agents(s) in regard of resources available in a given context to bring about changes through the implementation of an intervention” (4). Check what are the mediators to produce outcomes (not only primary, but also secondary or unintended outcomes).**

- CHWs had deep knowledge of their clients' culture, which enhanced communication, cooperation, and trust.
- Effective communication and collaboration between CHWs and the community facilitated the tracing of defaulters and improved outcomes.
- Clarity in the policy framework and understanding of their roles and responsibilities provided CHWs with a sense of purpose and clarity in their work.
- Consistent health priorities, such as TB, maternal health, and HIV/AIDS, provided a clear focus for CHWs and facilitated their work.
- The presence of effective OTLs (Organizational Team Leaders) and individualized solutions to facility planning improved CHW integration and tracing success.
- The adaptability of CHW models under resource constraints played a crucial role in successful implementation.
- The trust and recognition of CHWs by the communities they serve contributed to their ability to promote health and link communities to healthcare facilities.

**Table 3 – Outcomes**

- **Please make note of all (intended or not) outcomes. Please also classify the role and functions outcomes of CHWs using the following Pillars for people-centred care:**

1. Engaging and empowering people and communities

2. Strengthening governance and accountability (Incl. Supervision, training, and support)

3. Reorienting the model of care

4. Coordinating services within and across sectors

5. Enabling environment

- The study provided a detailed landscape of CHW policy implementation across several South African provinces, highlighting the variations in implementation models and the challenges that still exist.
- The presence of an involved OTL was found to moderate the relationship between facility-based staff and CHWs, acting as a facilitator or barrier to implementation.
- The study emphasized the need for permanent employment, uniforms, and higher wages for CHWs to ensure their visibility and recognition.
- Mobile health solutions were recommended to help CHWs effectively plan their work and avoid wasting efforts on patients who are no longer within their communities.
- The study identified the importance of better integrating CHWs into the current primary healthcare system to enhance collaboration and understanding between facility-based staff and CHWs.
- CHWs demonstrated purpose and passion in their work, acting as a bridge between communities and healthcare facilities.
- CHWs continued to promote health and link their communities to healthcare facilities despite challenges such as lack of permanent employment, limited resources, and low wages.
- Effective communication and collaboration between CHWs and the community were reported to enhance the effectiveness of their work, particularly in tracing defaulters.
- The presence of NGOs sometimes created confusion about work roles, while lack of communication between CHWs and healthcare facilities was a symptom of transitioning from NGO to Department of Health supervision.
- The implementation of CHW policy varied across provinces, highlighting the adaptability required under resource constraints.
- Clarity on elements of the policy framework, such as required qualifications of CHWs, was raised as an issue at the facility and community level.

**Outcome by PCC pillars:**

**Engaging and empowering people and communities:**

- CHWs act as a bridge between communities and healthcare facilities, promoting health and linking communities to healthcare services.
- CHWs demonstrate purpose, passion, and deep knowledge of their clients' culture, enhancing communication, cooperation, and trust within the community.
- CHWs continue to promote health and link their communities to healthcare facilities, despite challenges such as lack of permanent employment, limited resources, and low wages.

**Strengthening governance and accountability (Incl. Supervision, training, and support):**

- Effective OTLs (Organizational Team Leaders) and individualized solutions to facility planning improve CHW integration and tracing success.
- Clarity in the policy framework and understanding of roles and responsibilities provide CHWs with a sense of purpose and clarity in their work.
- CHWs receive support from implementing partners, including NGOs, who work with them.

**Reorienting the model of care:**

- CHWs serve as part of South Africa's Ward-Based Primary Healthcare Outreach Teams, acting as a bridge between communities and healthcare service provision within health facilities.
- CHWs provide community-based care, including medication and minor treatments, and play a crucial role in promoting health and linking communities to healthcare facilities.

**Coordinating services within and across sectors:**

- CHWs facilitate the tracing of patients who miss visits for HIV or TB services, improving coordination and continuity of care.
- Effective communication and collaboration between CHWs and the community enhance the coordination of services and improve outcomes.

**Enabling environment:**

- CHWs work under resource constraints, requiring adaptability for successful implementation.
- The trust and recognition of CHWs by the communities they serve contribute to their ability to promote health and link communities to healthcare facilities

**Table 4 – If, then statements depicting the CMOs.**

**Please detail the CMOs for the study you reviewed using the If (Context), Then (Mechanism) statements.**

**If, Then statements**

- If there is deep cultural understanding among CHWs, then this fosters respectful and culturally appropriate communication, which in turn enhances cooperation and trust. This understanding helps prevent misunderstandings and ensures a positive reception of healthcare messages, ultimately leading to effective care delivery.
- If CHWs have a clear understanding of the policy framework governing their roles and responsibilities, then CHWs will develop a sense of purpose and clarity in their work, leading to better integration into the system and enhanced professionalism. Therefore, improved performance.
- If there is a presence of effective Outreach Team Leaders, then CHWs will feel supported and motivated to perform their tasks because OTLs offer mentorship, feedback, and resources, leading to the successful delivery of healthcare services to the communities they serve.

**Test for relevance (Pearson et al. 2012; 2015; Brennan et al. 2017)**

| **Conceptually Rich** | **Thicker description’ but not ‘conceptually rich’** | **Conceptually Thin** |
| --- | --- | --- |
| Unambiguous theoretical concepts are described in sufficient depth. | Description of programme theory or sufficient information to enable it to ‘surface’. | Insufficient information to enable the programme theory to surface. |
| Relationships between, amongst concepts are clearly articulated. | Consideration of the context in which the programme takes place. | Limited or no consideration of the context in which the programme took place. |
| Concepts are sufficiently developed, defined to enable understanding without the reader needing to have first-hand experience of an area of practice. | Discussion of the differences between the design and orientation of programme theory (what was intended) and implementation (what really happened). | Limited or no discussion of the differences between the design and orientation of programme theory (what was intended) and implementation (what really happened). |
| Concepts are grounded strongly in a cited body of literature. | Recognition and discussion of the strengths/weaknesses of the implemented programme. | Limited or no discussion of the strengths/ weaknesses of the implemented programme. |
| Concepts are parsimonious (i.e., provide the simplest, but not over-simplified, explanation) | Some attempt to explain anomalous results and findings with reference to context and data. | No attempts to explain anomalous results and findings with reference to context and data. |
|  | Description of the factor affecting implementation. | Limited or no description of the factors affecting implementation. |

**Test for relevance (Pearson et al. 2012; 2015; Brennan et al. 2017)**

| **Conceptually Rich** | **Thicker description’ but not ‘conceptually rich’** | **Conceptually Thin** |
| --- | --- | --- |
| X |  |  |

**Test for rigour (Ohly et al. 2017)**

|  | **Yes** | **Fairly** | **No** |
| --- | --- | --- | --- |
| The study methods are clearly reported. | X |  |  |
| The study methods are appropriate to answer RQ. |  | X |  |
| The sample characteristics enable generalizability. |  |  | X |
| Raw data supports the study findings (conclusions). | X |  |  |
| Limitations of the study are acknowledged and clearly reported. | X |  |  |

**References**

1. Wong G, Westhorp G, Manzano A, Greenhalgh J, Jagosh J, Greenhalgh T. (2016). RAMESES II

reporting standards for realist evaluations. *BMC Medicine*, 14(1), 1-18.

2. Wong G, Greenhalgh T, Westhorp G, Pawson R. (2012). Realist methods in medical education

research: what are they and what can they contribute? *Medical Education*, 46(1), 89-96.

3. Macfarlane F, Greenhalgh T, Humphrey C, Hughes J, Butler C, Pawson R. (2011). A new workforce

in the making?: A case study of strategic human resource management in a whole-system change

effort in healthcare. *Journal of Health Organization and Management*, 25(1), 55-72.

4. Lacouture A, Breton E, Guichard A, Ridde V. (2015). The concept of mechanism from a realist

approach: a scoping review to facilitate its operationalization in public health program evaluation.

*Implementation Science*, 10(1), 1-10.

5. Pearson M, Chilton R, Woods HB, Wyatt K, Ford T, Abraham C, et al. Implementing health promotion in schools: protocol for a realist systematic review of research and experience in the United Kingdom (UK). Syst Rev. 2012 Oct 20;1(1):48.

6. Pearson M, Brand SL, Quinn C, Shaw J, Maguire M, Michie S, et al. Using realist review to inform intervention development: methodological illustration and conceptual platform for collaborative care in offender mental health. Implementation Sci. 2015 Sep 28;10(1):134.

7. Brennan N, Bryce M, Pearson M, Wong G, Cooper C, Archer J. Towards an understanding of how appraisal of doctors produces its effects: a realist review. Medical Education. 2017;51(10):1002–13.

8. Ohly H, Crossland N, Dykes F, Lowe N, Hall-Moran V. A realist review to explore how low-income pregnant women use food vouchers from the UK’s Healthy Start programme. BMJ Open. 2017 Apr 21;7(4):e013731.

**A realist evaluation of the role and functions of community health workers in service of a people-centred community health system.**

**Investigator CMOc Data Extraction Sheet Data Extraction**

The purpose of this document is to extract data from the identified studies in order to formulate one or more explanatory theories that explain Context-Mechanism-Outcome Configurations (CMOC) and to gain insight into how these CMOCs contribute to optimizing the role and functions of community health workers within a people-centred community health system.

In realist methodologies, knowledge accumulation centres on our comprehension of how mechanisms behave in various contexts and the reasons behind how and why this interplay of context and mechanism leads to different outcomes (1). Please fill in the details below for the study you will be assessing. In the subsequent three tables, locate and specify the Context, Mechanism, and Outcomes (CMO) of the intervention discussed in the study you've examined. Definitions and illustrations of CMO are provided within the tables.

**Kindly fill out the information below for the study you will be reviewing.**

| **Reviewer Name** | Usangiphile Buthelezi |
| --- | --- |
| **Reference** | Yuh MN, Ndum Okwen GA, Miong RHP, Bragazzi NL, Kong JD, Movahedi Nia Z, et al. Using an innovative family-centered evidence toolkit to improve the livelihood of people with disabilities in Bamenda (Cameroon): a mixed-method study. Front Public Health. 2023;11:1190722. |
| **Country** | Cameroon |
| **Setting and population** | - The study was conducted in Bamenda, Cameroon, which is the third largest city in the country, with approximately 573,000 inhabitants, representing about 2.6% of the total population of Cameroon. - The study population consisted of children and adults living with disabilities in Bamenda, Cameroon, ranging from 2 to 38 years old. - In terms of demographics, the population included 26 individuals, with 18 females (69.2%) and 8 males (30.8%). - Age distribution within the study group varied, with 26.9% aged 0-9 years, 57.7% aged 10-20 years, 7.7% aged 21-30 years, and 7.7% aged 31-40 years. - The most prevalent types of impairments reported were physical or motor impairments (84.6%), followed by mental disabilities (26.9%) and sensory (hearing) impairments (11.5%). - Notably, there were no cases of visual impairment or other types of disabilities such as intellectual, relational, or developmental disabilities included in the study population. |
| **Aim** | The study aimed to create an intervention framework that would focus on enhancing the livelihoods of individuals with disabilities in low- and middle-income countries, where existing strategies may not be as effective |
| **Objectives** | - The objectives included designing an innovative toolkit that could be used by families and communities to better support and empower people with disabilities in the Cameroonian context. - Another aim was to pilot this toolkit and assess its impact on various aspects of the lives of individuals with disabilities, such as their access to healthcare, education, and opportunities for economic empowerment. - The study also sought to explore the challenges and opportunities faced by people with disabilities in Bamenda, Cameroon, with a focus on engaging the community in participatory research to address these issues. |
| **Methodology** | - The research used a mixed-method approach, which means it combined different research methods, including structured questionnaires, to gather information from the participants effectively. - Initially, 56 people with disabilities were included in the study, and a snowball sampling technique was employed to expand the participant pool through referrals by stakeholders, service providers, community members, and families with individuals having disabilities. - Quantitative data was collected from 26 participants, with a majority being females (69.2%) and primarily reporting physical or motor impairments (84.6%). - The study focused on addressing the lack of accessible interventions for people with disabilities in low- and middle-income countries by creating a culturally sensitive toolkit to improve their livelihoods, education, and overall well-being. |
| **Intervention description** | - The intervention in the study focused on using an innovative family-centered evidence toolkit called EFCETD to improve the livelihoods of people with disabilities in Bamenda, Cameroon. - Community health workers conducted regular household visits every 2 to 3 months over a 12-month period to implement the intervention. These visits aimed at providing counseling, guidance, and support to families with individuals with disabilities. - Through the EFCETD toolkit, community health workers administered evidence-based recommendations to help families manage and care for their children or adults with disabilities effectively. This approach aimed at empowering families and individuals with disabilities to make informed choices and lead independent lives. - The intervention also involved data collection through structured questionnaires and interviews guided by the toolkit's questions. This data collection process was crucial in assessing the impact of the intervention on the livelihoods and well-being of people with disabilities in Bamenda. - The intervention's holistic approach addressed various aspects of livelihood, education, and overall well-being of individuals with disabilities. By incorporating best practices and innovation, the EFCETD toolkit was designed to be culturally sensitive and tailored to the specific needs of the community in Bamenda. - Overall, the intervention's goal was to bridge the gap in access to quality interventions for people with disabilities in low- and middle-income countries like Cameroon. By empowering families and individuals with disabilities through evidence-based support and guidance, the intervention aimed to enhance their quality of life and promote inclusive development in the community. |
| **Intervention outcomes** | - The study results showed that individuals with disabilities in Bamenda, Cameroon faced significant challenges that limited their full participation and inclusion in society. - Despite efforts to improve livelihoods through interventions like household visits by community health workers, discriminatory attitudes and ableism still posed obstacles to the inclusion of people with disabilities. - The findings highlighted the complexities of addressing the multidimensional issues faced by persons living with disabilities and the importance of a human rights-based approach to disability. - While the intervention aimed to empower families, enable informed decision-making, and enhance independence for individuals with disabilities, limited funding hindered the ability to reach a larger sample size and impact more livelihoods. - The study limitations acknowledged that the sample size may not fully represent all age groups and areas of children and adults with disabilities in Bamenda, impacting the generalizability of the findings. - Despite these limitations, the study provided valuable insights into the well-being and challenges faced by individuals with disabilities in Cameroon, offering a foundation for future research and interventions to further improve livelihoods and inclusivity. - Overall, the results showcased the need for continued efforts to support disability-inclusive development, advance the rights of individuals with disabilities, and expand interventions like the EFCETD toolkit to create positive change within the community and beyond. |
| **Challenges and limitations** | - **Limited Funding:**   - The study had constraints due to limited funding, impacting the number of participants the intervention could reach. - **Sample Size and Representation:**   - The sampling technique used may not have represented all age groups and residential areas of individuals with disabilities in Bamenda, affecting the study's generalizability. - **Impact and Statistical Power:**   - The small sample size of 30 out of 56 recruited individuals limited the statistical power of the study and the breadth of livelihoods impacted. |

**Table 1 – Context**

**Including (but not limited to) beliefs, social and cultural norms, regulations and economic factors (2,3). For example:**

- Individual capabilities (i.e. values, roles, knowledge, purpose).
- Interpersonal relationships supporting the interventions (i.e. communication, collaboration, network, influences).
- Institutional settings (i.e. informal rules, organisational culture, leadership, policies, resource allocation, local priorities).
- Infra-structural system (i.e. political support).
- Country and rurality (i.e. small or large/rural vs regional vs remote).
- Socio-demographic characteristics:
- Funding context or source (i.e. free, personalised, group vs. individual, government funded).

**Study Setting and Population:**

- The study was conducted in Bamenda, Cameroon, where community health workers visited families of people with disabilities over 12 months, with visits every 2-3 months.
- Due to a socio-political crisis, there was attrition in participants during the follow-up visits, with some families constantly displaced.

**Training and Education Level of the Population:**

- The study engaged community health workers who visited households to provide support and education. The exact training and education levels of the population were not explicitly mentioned in the provided contexts.

**Available Resources in the Community:**

- The study utilized an innovative Family-Centered Evidence Toolkit for Disabilities (EFCETD) installed on smartphones to collect data during household visits by community health workers.
- The community health workers were assigned specific households to visit, ensuring coverage and effective data collection.

**Funding and Economic Status:**

- The details regarding funding for the study and the economic status of the population were not specifically outlined in the contexts provided.

**Facilities and Education Level of Community Health Workers:**

- Community health workers carried out household visits, administered the toolkit, and provided counseling on evidence-based recommendations for managing disabilities.
- The community health workers were responsible for educating families on improving the livelihood of people with disabilities, indicating a level of training and expertise, though their formal education levels were not detailed in the contexts provided.

**Table 2 – Mechanisms**

- **“An element of reasoning and/or reactions of an individual or collective agents(s) in regard of resources available in a given context to bring about changes through the implementation of an intervention” (4). Check what are the mediators to produce outcomes (not only primary, but also secondary or unintended outcomes).**

- **Trust:** Building trust between community health workers and families of people with disabilities was crucial for the success of the intervention. Trust allowed for open communication, collaboration, and acceptance of recommendations, fostering a positive environment for change.
- **Empowerment:** Empowering families through education and support empowered them to make informed choices and take actions to enhance the livelihood of people with disabilities. This sense of empowerment increased their confidence and ability to address challenges effectively.
- **Rapport and Credibility:** Establishing a strong rapport and credibility between community health workers and families facilitated effective communication and implementation of recommended practices. This helped in gaining the families' trust and cooperation.
- **Recognition and Support:** Providing recognition, support, and a sense of purpose to families enhanced their motivation to actively engage in improving the livelihoods of people with disabilities. Feeling valued and supported encouraged families to persist in overcoming obstacles.
- **Competence and Accountability:** Ensuring that community health workers demonstrated competence in their roles and were held accountable for their actions was essential. Their expertise and accountability instilled confidence in families, ensuring the credibility of the intervention.
- **Culture and Motivation:** Considering the cultural context of the community and aligning the intervention with local beliefs and practices played a significant role in motivating families to participate. Cultural sensitivity enhanced acceptance and engagement in the intervention, contributing to its success.

**Table 3 – Outcomes**

- **Please make note of all (intended or not) outcomes. Please also classify the role and functions outcomes of CHWs using the following Pillars for people-centred care:**

1. Engaging and empowering people and communities

2. Strengthening governance and accountability (Incl. Supervision, training, and support)

3. Reorienting the model of care

4. Coordinating services within and across sectors

5. Enabling environment

**Outcomes according to PCC pillars:**

- **Engaging and Empowering People and Communities:**
  - Engaging families through household visits by community health workers helped in providing direct support and guidance to improve the livelihood of persons with disabilities.
  - Empowering families with knowledge and resources enabled them to make informed decisions and actively participate in activities that enhanced the well-being of their loved ones.
- **Strengthening Governance and Accountability:**
  - Implementation of supervision, training, and ongoing support for community health workers ensured the quality and effectiveness of the intervention.
  - By holding workers accountable for their actions, competence was maintained, building trust with families and promoting credibility.
- **Reorienting the Model of Care:**
  - The intervention reoriented care by focusing on holistic support, encompassing health, education, livelihood, social aspects, and empowerment.
  - Shifting the care model to address the diverse needs of persons with disabilities emphasized a comprehensive approach to improving livelihoods.
- **Coordinating Services within and across Sectors:**
  - Coordinated efforts among various sectors such as health, social services, and civil society organizations were crucial in ensuring a comprehensive support system for persons with disabilities.
  - Collaboration and communication between different service providers facilitated a smooth delivery of resources and assistance to families in need.
- **Enabling Environment:**
  - Creating an enabling environment involved aligning the intervention with local culture and practices, promoting acceptance and engagement.
  - By fostering trust, recognition, and support, the intervention encouraged a conducive environment for families to actively participate in improving the livelihood of individuals with disabilities.

**Table 4 – If, then statements depicting the CMOs.**

**Please detail the CMOs for the study you reviewed using the If (Context), Then (Mechanism) statements.**

- **IF** families are engaged through household visits by community health workers and empowered with knowledge and resources, **THEN** families can make informed decisions and actively participate in activities that enhance the well-being of their loved ones, **BECAUSE** direct support and guidance, along with empowerment, enable families to take effective actions to improve the livelihood of persons with disabilities.
- **IF** supervision, training, and ongoing support for community health workers are implemented, and workers are held accountable for their actions, **THEN** the quality and effectiveness of the intervention will be ensured, building trust with families and promoting credibility, **BECAUSE** maintaining competence and accountability instills confidence in families regarding the intervention's credibility and effectiveness.
- **IF** the intervention reorients care to focus on holistic support encompassing health, education, livelihood, social aspects, and empowerment, **THEN** the diverse needs of persons with disabilities will be addressed, leading to a comprehensive approach to improving livelihoods, **BECAUSE** addressing multiple aspects of well-being emphasizes a holistic approach that fosters significant improvements in the lives of persons with disabilities.
- **IF** coordinated efforts among various sectors such as health, social services, and civil society organizations are made, **THEN** a comprehensive support system for persons with disabilities will be ensured, **BECAUSE** collaboration and communication between different service providers facilitate a smooth delivery of resources and assistance to families in need.
- **IF** the intervention aligns with local culture and practices and fosters trust, recognition, and support, **THEN** families will be more likely to actively participate in improving the livelihood of individuals with disabilities, **BECAUSE** cultural sensitivity promotes acceptance and engagement, creating a conducive environment for active participation and support.

**Test for relevance (Pearson et al. 2012; 2015; Brennan et al. 2017)**

| **Conceptually Rich** | **Thicker description’ but not ‘conceptually rich’** | **Conceptually Thin** |
| --- | --- | --- |
| Unambiguous theoretical concepts are described in sufficient depth. | Description of programme theory or sufficient information to enable it to ‘surface’. | Insufficient information to enable the programme theory to surface. |
| Relationships between, amongst concepts are clearly articulated. | Consideration of the context in which the programme takes place. | Limited or no consideration of the context in which the programme took place. |
| Concepts are sufficiently developed, defined to enable understanding without the reader needing to have first-hand experience of an area of practice. | Discussion of the differences between the design and orientation of programme theory (what was intended) and implementation (what really happened). | Limited or no discussion of the differences between the design and orientation of programme theory (what was intended) and implementation (what really happened). |
| Concepts are grounded strongly in a cited body of literature. | Recognition and discussion of the strengths/weaknesses of the implemented programme. | Limited or no discussion of the strengths/ weaknesses of the implemented programme. |
| Concepts are parsimonious (i.e., provide the simplest, but not over-simplified, explanation) | Some attempt to explain anomalous results and findings with reference to context and data. | No attempts to explain anomalous results and findings with reference to context and data. |
|  | Description of the factor affecting implementation. | Limited or no description of the factors affecting implementation. |

**Test for relevance (Pearson et al. 2012; 2015; Brennan et al. 2017)**

| **Conceptually Rich** | **Thicker description’ but not ‘conceptually rich’** | **Conceptually Thin** |
| --- | --- | --- |
|  | **X** |  |

**Test for rigour (Ohly et al. 2017)**

|  | **Yes** | **Fairly** | **No** |
| --- | --- | --- | --- |
| The study methods are clearly reported. | X |  |  |
| The study methods are appropriate to answer RQ. | X |  |  |
| The sample characteristics enable generalizability. |  |  | X |
| Raw data supports the study findings (conclusions). | X |  |  |
| Limitations of the study are acknowledged and clearly reported. | X |  |  |

**References**

1. Wong G, Westhorp G, Manzano A, Greenhalgh J, Jagosh J, Greenhalgh T. (2016). RAMESES II

reporting standards for realist evaluations. *BMC Medicine*, 14(1), 1-18.

2. Wong G, Greenhalgh T, Westhorp G, Pawson R. (2012). Realist methods in medical education

research: what are they and what can they contribute? *Medical Education*, 46(1), 89-96.

3. Macfarlane F, Greenhalgh T, Humphrey C, Hughes J, Butler C, Pawson R. (2011). A new workforce

in the making?: A case study of strategic human resource management in a whole-system change

effort in healthcare. *Journal of Health Organization and Management*, 25(1), 55-72.

4. Lacouture A, Breton E, Guichard A, Ridde V. (2015). The concept of mechanism from a realist

approach: a scoping review to facilitate its operationalization in public health program evaluation.

*Implementation Science*, 10(1), 1-10.

5. Pearson M, Chilton R, Woods HB, Wyatt K, Ford T, Abraham C, et al. Implementing health promotion in schools: protocol for a realist systematic review of research and experience in the United Kingdom (UK). Syst Rev. 2012 Oct 20;1(1):48.

6. Pearson M, Brand SL, Quinn C, Shaw J, Maguire M, Michie S, et al. Using realist review to inform intervention development: methodological illustration and conceptual platform for collaborative care in offender mental health. Implementation Sci. 2015 Sep 28;10(1):134.

7. Brennan N, Bryce M, Pearson M, Wong G, Cooper C, Archer J. Towards an understanding of how appraisal of doctors produces its effects: a realist review. Medical Education. 2017;51(10):1002–13.

8. Ohly H, Crossland N, Dykes F, Lowe N, Hall-Moran V. A realist review to explore how low-income pregnant women use food vouchers from the UK’s Healthy Start programme. BMJ Open. 2017 Apr 21;7(4):e013731.

**A realist evaluation of the role and functions of community health workers in service of a people-centred community health system.**

**Investigator CMOc Data Extraction Sheet Data Extraction**

The purpose of this document is to extract data from the identified studies in order to formulate one or more explanatory theories that explain Context-Mechanism-Outcome Configurations (CMOC) and to gain insight into how these CMOCs contribute to optimizing the role and functions of community health workers within a people-centred community health system.

In realist methodologies, knowledge accumulation centres on our comprehension of how mechanisms behave in various contexts and the reasons behind how and why this interplay of context and mechanism leads to different outcomes (1). Please fill in the details below for the study you will be assessing. In the subsequent three tables, locate and specify the Context, Mechanism, and Outcomes (CMO) of the intervention discussed in the study you've examined. Definitions and illustrations of CMO are provided within the tables.

**Kindly fill out the information below for the study you will be reviewing.**

| **Reviewer Name** | Usangiphile Buthelezi |
| --- | --- |
| **Reference** | Ndaba T, Taylor M, Mabaso M. Training and Evaluation of Community Health Workers (CHWs): Towards Improving Maternal and Newborn Survival in an Urban Setting in KwaZulu-Natal, South Africa. The Open Public Health Journal [Internet]. 2019 Oct 29 [cited 2024 Apr 3];12(1). Available from: https://openpublichealthjournal.com/VOLUME/12/PAGE/406/ |
| **Country** | South Africa |
| **Setting and population** | - The study was conducted in Umlazi, a peri-urban area in KwaZulu-Natal, South Africa, with a population of approximately one million people. - Umlazi is characterized by overcrowding, formal and informal settlements, and a high rate of unemployment. - The EThekwini district, of which Umlazi is a part, has a high antenatal HIV prevalence and a high rate of Tuberculosis infections in adults and children due to overcrowding. - The study focused on three purposefully selected Primary Health Care (PHC) centers in Umlazi, which have a high workload and serve a catchment population of over 60,000 per clinic. - The population in this area experiences high neonatal and infant mortality rates. - A total of 47 Community Health Workers (CHWs) were recruited from the three selected PHC centers for the study. |
| **Aim** | - The primary aim of the study was to train and evaluate Community Health Workers (CHWs) on maternal and neonatal care in an impoverished urban setting in KwaZulu-Natal, South Africa, with the goal of improving maternal-newborn survival. |
| **Objectives** | - To assess the level of knowledge and understanding of CHWs before and after the training, as well as their ability to conduct home visits, provide support and care, and identify newborn danger signs for referrals. - To measure the impact of the CHW training on the utilization of maternal and neonatal health services in the community. - To provide valuable insights into the implementation of the CHW strategy and contribute to the general body of knowledge on the training of CHWs towards improved maternal and neonatal health. |
| **Methodology** | - The study utilized a descriptive study design to train and evaluate Community Health Workers (CHWs) on maternal and neonatal care in an impoverished urban setting in KwaZulu-Natal, South Africa. - A total of 47 CHWs were recruited from three purposefully selected Primary Health Care (PHC) centers with high workloads. - The training was conducted over three days and included pre-test and post-test assessments to measure the level of knowledge and understanding of the CHWs. - The training manual was developed based on UNICEF and WHO training materials, and the training sessions covered areas such as home visits, child feeding practices, and postnatal care at a community level. - The performance of CHWs was evaluated through shadow visits during home visits and spot checks with pregnant women. - The study assessed the CHWs' ability to conduct visits during pregnancy and after delivery, provide care for mothers and newborns, and identify newborn danger signs for referrals. - The study also evaluated the CHWs' skills in community mobilization and sensitization to stimulate demand for maternal and neonatal health services. |
| **Intervention description** | - The intervention in the study involved training Community Health Workers (CHWs) on maternal and neonatal care in an impoverished urban setting in KwaZulu-Natal, South Africa. - The training program was designed based on UNICEF and WHO training materials and covered areas such as home visits, child feeding practices, and postnatal care at a community level. - The training sessions included lectures, reading, presentations, discussions, demonstrations, and individual and group exercises. Role-plays and simulated home visits were also conducted to practice skills and encounter different scenarios. - The training aimed to equip CHWs with the necessary skills to conduct home visits, assess and support mothers, check on newborns' health and hygiene, provide breastfeeding support, and promote Kangaroo mother care. - The performance of CHWs was evaluated through pre-and post-test scores, shadow visits, and spot checks. The evaluation showed that most CHWs demonstrated the necessary skills for referrals, caring for newborns and their mothers at home, and community mobilization. |
| **Intervention outcomes** | - The training of Community Health Workers (CHWs) on maternal and neonatal care resulted in improved performance and skills among the CHWs. - CHWs demonstrated social commitment and purpose in the short term observed. - CHWs demonstrated the necessary skills for referrals to prevent complications, caring for newborns and their mothers at home immediately after discharge from health care centers. - CHWs showed competency in conducting visits during pregnancy and after delivery, providing care for mothers and newborns, and identifying newborn danger signs for referrals. - CHWs exhibited skills in community mobilization and sensitization to stimulate demand for maternal and neonatal health services. - The training program effectively recruited pregnant women, instituted referrals to avert complications, and followed up with newborns and their mothers at home. |
| **Challenges and limitations** | - Relocation of some participants made it difficult to locate them, limiting follow-up with mothers. - The relatively short study period of one month restricted the extent of follow-up with the mothers. - The study did not have a control arm, which limited the ability to measure improvements in maternal and newborn survival. - The small sample size weakened the statistical power of the study. - Generalizability of the findings was limited due to the small study sample and specific catchment area. - The study focused on evaluating the training outcomes through pre- and post-tests, shadow visits, and spot checks, rather than directly measuring improvements in maternal and newborn survival. |

**Table 1 – Context**

**Including (but not limited to) beliefs, social and cultural norms, regulations and economic factors (2,3). For example:**

- Individual capabilities (i.e. values, roles, knowledge, purpose).
- Interpersonal relationships supporting the interventions (i.e. communication, collaboration, network, influences).
- Institutional settings (i.e. informal rules, organisational culture, leadership, policies, resource allocation, local priorities).
- Infra-structural system (i.e. political support).
- Country and rurality (i.e. small or large/rural vs regional vs remote).
- Socio-demographic characteristics:
- Funding context or source (i.e. free, personalised, group vs. individual, government funded).

- The study was conducted in Umlazi, a peri-urban area in KwaZulu-Natal, South Africa, with a population of approximately one million people.
- Umlazi is characterized by overcrowding, formal and informal settlements, and a high rate of unemployment.
- The EThekwini district, of which Umlazi is a part, has a high antenatal HIV prevalence and a high rate of Tuberculosis infections in adults and children due to overcrowding.
- The study focused on three purposefully selected Primary Health Care (PHC) centers in Umlazi, which have a high workload and serve a catchment population of over 60,000 per clinic.
- The population in this area experiences high neonatal and infant mortality rates.
- A total of 47 Community Health Workers (CHWs) were recruited from the three selected PHC centers for the study.
- CHWs were selected from within the affected communities, indicating a close geographical and social proximity to the communities they served.
- CHWs were integrated into the formal health-care system, with defined roles and responsibilities.

**Table 2 – Mechanisms**

- **“An element of reasoning and/or reactions of an individual or collective agents(s) in regard of resources available in a given context to bring about changes through the implementation of an intervention” (4). Check what are the mediators to produce outcomes (not only primary, but also secondary or unintended outcomes).**

- CHWs demonstrated knowledge and skills in conducting visits, caring for mothers and newborns, and identifying danger signs for referrals, which increased their confidence and rapport with the community.
- Trust and recognition from the community, especially the mothers, played a crucial role in the success of CHWs.
- Effective communication and collaboration between CHWs and pregnant women, mothers, and their communities facilitated the mobilization and sensitization efforts to stimulate demand for maternal and neonatal health services.
- The integration of CHWs into the formal health-care system, with defined roles and responsibilities, provided institutional support for their training and performance.
- The Primary Health Care (PHC) re-engineering initiative, which prioritized maternal-newborn services and upskilling of CHWs, created an infrastructural system that supported the implementation and success of the interventions.
- CHWs demonstrated social commitment and purpose in the short term observed, indicating their strong motivation and dedication to their role.
- CHWs acquired competencies and knowledge on conducting visits during pregnancy and after delivery, caring for mothers and newborns, and identifying newborn danger signs for referrals. This knowledge and skill set contributed to their perception of being capable and effective in providing maternal and neonatal care.
- The training and evaluation of CHWs revealed that they demonstrated the necessary skills for referrals to prevent complications and provide care for newborns and their mothers at home. This suggests that their perception of their own abilities and the value of their work contributed to their social commitment and purpose.
- CHWs' values, such as a sense of social responsibility and a desire to make a positive impact on their communities, likely played a significant role in their commitment to providing maternal and neonatal care services.

**Table 3 – Outcomes**

- **Please make note of all (intended or not) outcomes. Please also classify the role and functions outcomes of CHWs using the following Pillars for people-centred care:**

1. Engaging and empowering people and communities

2. Strengthening governance and accountability (Incl. Supervision, training, and support)

3. Reorienting the model of care

4. Coordinating services within and across sectors

5. Enabling environment

- The training of Community Health Workers (CHWs) on maternal and neonatal care resulted in improved performance and skills among the CHWs.
- CHWs demonstrated social commitment and purpose in the short term observed.
- CHWs demonstrated the necessary skills for referrals to prevent complications, caring for newborns and their mothers at home immediately after discharge from health care centers.
- CHWs showed competency in conducting visits during pregnancy and after delivery, providing care for mothers and newborns, and identifying newborn danger signs for referrals.
- CHWs exhibited skills in community mobilization and sensitization to stimulate demand for maternal and neonatal health services.
- The training program effectively recruited pregnant women, instituted referrals to avert complications, and followed up with newborns and their mothers at home.

**Outcomes by PCC pillars:**

**Engaging and empowering people and communities:**

- Community Health Workers (CHWs) demonstrated social commitment and purpose in the short term observed, showing their engagement and empowerment in providing maternal and neonatal care.
- CHWs were trained to conduct home visits, assess and support mothers, check on babies' well-being, and provide advice on Kangaroo mother care, promoting community engagement and empowerment.

**Strengthening governance and accountability (Incl. Supervision, training, and support):**

- CHWs received training on maternal and newborn care, including home visits, child feeding practices, and postnatal care, to enhance their skills and knowledge.
- Pre-and post-test scores, shadow visits, and spot checks were used to evaluate the performance of CHWs, ensuring accountability and quality improvement.

**Reorienting the model of care:**

- The training of CHWs focused on improving maternal and newborn survival by utilizing CHWs from the community, emphasizing the importance of community-based care.
- CHWs were trained to provide support and care during pregnancy and after delivery, promoting a reoriented model of care that prioritizes the health of newborns and their mothers.

**Coordinating services within and across sectors:**

- CHWs were trained to check if mothers were given the Road to Health card, inquire about immunization and follow-up visits, and encourage adherence to follow-up dates at Primary Health Care (PHC) clinics, promoting coordination between CHWs and PHC services.
- CHWs were also trained on the roles and responsibilities of Community Advisory Groups (CAG) and Primary Health Care Development Committees (PDQ), enhancing coordination between CHWs and community structures.

**Enabling environment:**

- The training of CHWs on maternal-newborn services aimed to prioritize the most affected areas, creating an enabling environment for improved health outcomes in impoverished communities.

**Table 4 – If, then statements depicting the CMOs.**

**Please detail the CMOs for the study you reviewed using the If (Context), Then (Mechanism) statements.**

**If, Then statements**

- If CHWs receive training, they will gain knowledge and skills necessary for conducting visits effectively, fostering confidence and rapport with the community they serve, leading to improved performance, high-quality care delivery, and ultimately better health outcomes for the community.
- If there is effective communication and collaboration between CHWs and pregnant women, mothers, and their communities, then mobilization and sensitization efforts will be facilitated, stimulating demand for maternal and neonatal health services because recognizing the value of these services will motivate individuals to use them for their well-being and that of their families, ultimately improving access and utilization of healthcare services for mothers and newborns.
- If CHWs are integrated into the formal healthcare system with defined roles and responsibilities, they feel recognized and acknowledged as legitimate healthcare providers. This integration ensures that their contributions are valued and respected, leading to enhanced effectiveness in delivering healthcare services to the community.
- If there is a Primary Healthcare (PHC) re-engineering initiative prioritizing upskilling and infrastructure support for CHWs, then CHWs will feel motivated, empowered, and competent to perform their duties due to the availability of resources and enhanced knowledge and skills. Consequently, this results in the delivery of high-quality care to the community.
- If CHWs receive training and acquire skills for referrals to prevent complications and provide care at home, then CHWs will perceive themselves capable of providing services that contribute to the well-being of their community, fostering empowerment and sense of purpose in their work. As a result, CHWs will demonstrate increased dedication and effectiveness in their efforts to improve the health outcomes of their community members.

**Test for relevance (Pearson et al. 2012; 2015; Brennan et al. 2017)**

| **Conceptually Rich** | **Thicker description’ but not ‘conceptually rich’** | **Conceptually Thin** |
| --- | --- | --- |
| Unambiguous theoretical concepts are described in sufficient depth. | Description of programme theory or sufficient information to enable it to ‘surface’. | Insufficient information to enable the programme theory to surface. |
| Relationships between, amongst concepts are clearly articulated. | Consideration of the context in which the programme takes place. | Limited or no consideration of the context in which the programme took place. |
| Concepts are sufficiently developed, defined to enable understanding without the reader needing to have first-hand experience of an area of practice. | Discussion of the differences between the design and orientation of programme theory (what was intended) and implementation (what really happened). | Limited or no discussion of the differences between the design and orientation of programme theory (what was intended) and implementation (what really happened). |
| Concepts are grounded strongly in a cited body of literature. | Recognition and discussion of the strengths/weaknesses of the implemented programme. | Limited or no discussion of the strengths/ weaknesses of the implemented programme. |
| Concepts are parsimonious (i.e., provide the simplest, but not over-simplified, explanation) | Some attempt to explain anomalous results and findings with reference to context and data. | No attempts to explain anomalous results and findings with reference to context and data. |
|  | Description of the factor affecting implementation. | Limited or no description of the factors affecting implementation. |

**Test for relevance (Pearson et al. 2012; 2015; Brennan et al. 2017)**

| **Conceptually Rich** | **Thicker description’ but not ‘conceptually rich’** | **Conceptually Thin** |
| --- | --- | --- |
| X |  |  |

**Test for rigour (Ohly et al. 2017)**

|  | **Yes** | **Fairly** | **No** |
| --- | --- | --- | --- |
| The study methods are clearly reported. | X |  |  |
| The study methods are appropriate to answer RQ. | X |  |  |
| The sample characteristics enable generalizability. |  |  | X |
| Raw data supports the study findings (conclusions). | X |  |  |
| Limitations of the study are acknowledged and clearly reported. | X |  |  |

**References**

1. Wong G, Westhorp G, Manzano A, Greenhalgh J, Jagosh J, Greenhalgh T. (2016). RAMESES II

reporting standards for realist evaluations. *BMC Medicine*, 14(1), 1-18.

2. Wong G, Greenhalgh T, Westhorp G, Pawson R. (2012). Realist methods in medical education

research: what are they and what can they contribute? *Medical Education*, 46(1), 89-96.

3. Macfarlane F, Greenhalgh T, Humphrey C, Hughes J, Butler C, Pawson R. (2011). A new workforce

in the making?: A case study of strategic human resource management in a whole-system change

effort in healthcare. *Journal of Health Organization and Management*, 25(1), 55-72.

4. Lacouture A, Breton E, Guichard A, Ridde V. (2015). The concept of mechanism from a realist

approach: a scoping review to facilitate its operationalization in public health program evaluation.

*Implementation Science*, 10(1), 1-10.

5. Pearson M, Chilton R, Woods HB, Wyatt K, Ford T, Abraham C, et al. Implementing health promotion in schools: protocol for a realist systematic review of research and experience in the United Kingdom (UK). Syst Rev. 2012 Oct 20;1(1):48.

6. Pearson M, Brand SL, Quinn C, Shaw J, Maguire M, Michie S, et al. Using realist review to inform intervention development: methodological illustration and conceptual platform for collaborative care in offender mental health. Implementation Sci. 2015 Sep 28;10(1):134.

7. Brennan N, Bryce M, Pearson M, Wong G, Cooper C, Archer J. Towards an understanding of how appraisal of doctors produces its effects: a realist review. Medical Education. 2017;51(10):1002–13.

8. Ohly H, Crossland N, Dykes F, Lowe N, Hall-Moran V. A realist review to explore how low-income pregnant women use food vouchers from the UK’s Healthy Start programme. BMJ Open. 2017 Apr 21;7(4):e013731.

**A realist evaluation of the role and functions of community health workers in service of a people-centred community health system.**

**Investigator CMOc Data Extraction Sheet Data Extraction**

The purpose of this document is to extract data from the identified studies in order to formulate one or more explanatory theories that explain Context-Mechanism-Outcome Configurations (CMOC) and to gain insight into how these CMOCs contribute to optimizing the role and functions of community health workers within a people-centred community health system.

In realist methodologies, knowledge accumulation centres on our comprehension of how mechanisms behave in various contexts and the reasons behind how and why this interplay of context and mechanism leads to different outcomes (1). Please fill in the details below for the study you will be assessing. In the subsequent three tables, locate and specify the Context, Mechanism, and Outcomes (CMO) of the intervention discussed in the study you've examined. Definitions and illustrations of CMO are provided within the tables.

**Kindly fill out the information below for the study you will be reviewing.**

| **Reviewer Name** | Usangiphile Buthelezi |
| --- | --- |
| **Reference** | Soepnel LM, Norris SA, Mabetha K, Motlhatlhedi M, Nkosi N, Lye S, et al. A qualitative analysis of community health worker perspectives on the implementation of the preconception and pregnancy phases of the Bukhali randomised controlled trial. PLOS Glob Public Health. 2024;4(3):e0002578. |
| **Country** | South Africa |
| **Setting and population** | - The research was conducted in Soweto, South Africa, focusing on young women to improve their health outcomes through a CHW-delivered intervention called Bukhali. - The study involved 13 Health Helpers (HHs), which are a type of CHWs employed by the Bukhali trial to deliver the intervention to the participants. - The population targeted for the intervention included young women in the preconception and pregnancy phases, emphasizing the importance of improving health trajectories for this specific group. - The HHs interacted with the existing public healthcare sector, dealt with participant perceptions of health, addressed health literacy and language barriers, considered socioeconomic constraints, acknowledged family, partner, and community views, and maintained the HH-participant relationship to ensure effective implementation of the intervention. |
| **Aim** | The aim of the study was to explore the factors influencing the implementation of the preconception and pregnancy phases of the Bukhali intervention from the perspective of Community Health Workers (CHWs) known as Health Helpers (HHs) |
| **Objectives** | - The main objective was to qualitatively analyze the perspectives of the CHWs delivering the Bukhali intervention in Soweto, South Africa, by conducting focus group discussions to understand the challenges and facilitators they encountered during the implementation process. - Through these discussions, the study aimed to identify key themes affecting the roles of HHs in delivering the intervention, such as interactions with the healthcare sector, participant perceptions of health, health literacy and language barriers, socioeconomic constraints, family and community views, and the HH-participant relationship. - Additionally, the study intended to provide recommendations for adaptations in the implementation of the Bukhali intervention based on the identified factors and insights from the CHWs, to improve the effectiveness of similar trials focusing on preconception and pregnancy phases, and to enhance the role of CHWs in healthcare settings facing similar challenges. |
| **Methodology** | - Qualitative research approach used to explore the factors influencing the implementation of the Bukhali intervention from the perspective of Community Health Workers (CHWs) in Soweto, South Africa. - Three focus group discussions conducted with 13 Health Helpers (HHs) employed by the Bukhali trial to gather insights and perspectives on the challenges and facilitators encountered during the implementation process. - Thematic analysis applied to identify key themes affecting the HHs' roles in delivering the intervention, such as interactions with the healthcare sector, participant perceptions of health, and addressing socioeconomic constraints. - Data analysis involved elements of both reflexive thematic and codebook approaches to comprehensively understand the factors influencing the implementation of the preconception and pregnancy phases of the Bukhali intervention. - Recommendations provided for adaptations in the implementation process based on the findings to enhance the effectiveness of similar trials focusing on preconception and pregnancy phases and strengthen the role of CHWs in healthcare settings facing similar challenges. |
| **Intervention description** | - The Bukhali intervention is a program delivered by trained Community Health Workers (CHWs) called Health Helpers (HHs) in Soweto, South Africa, focusing on improving the health of young women during preconception and pregnancy phases. - CHWs interact with the public healthcare sector, address participant health perceptions, consider health literacy and language barriers, tackle socioeconomic constraints, account for family and community views, and maintain a strong relationship with participants. - The intervention aims to overcome implementation challenges by providing targeted support to participants facing food insecurity, unemployment, transportation issues, and other barriers to adopting healthier behaviors and following through on healthcare recommendations. - Strategies include navigating the healthcare system, adapting to individual participant needs, and addressing health-related stigma, with the HH-participant relationship identified as a critical mechanism for intervention success. - Findings from the study provide valuable insights for enhancing similar CHW-delivered interventions in preconception and pregnancy trials and strengthening CHW roles in challenging healthcare settings. |
| **Intervention outcomes** | - A key outcome of the intervention was the identification of three strategic areas for improving CHW-delivered interventions: navigating healthcare systems, adapting to individual needs, and addressing health-related stigma. - The relationship between CHWs and participants was highlighted as a pivotal mechanism for the intervention's impact, emphasizing the importance of trust and communication. - Socioeconomic factors, such as food insecurity and unemployment, impacted how participants engaged with the intervention, affecting behaviors like dietary changes and supplement intake. - To address implementation challenges, CHWs used trial-based tools and strategies to enhance their roles and ensure the intervention's success. - Recommendations from the study included intensified follow-up on referrals, ongoing training for CHWs, increased community engagement, and a focus on health-related education and stigma reduction |
| **Challenges and limitations** | - The study only presents a snapshot of current perspectives on the implementation of Bukhali, rather than a comprehensive overview representing all trial phases. - Since the Health Helpers (HHs) were employed by the trial, power dynamics between researchers/interviewers and HHs could have influenced the HHs' responses during focus group discussions. - The qualitative results may not be universally applicable to different trial populations due to potential researcher subjectivity influencing the analyses. - The study's focus on the preconception and pregnancy phases might limit a broader understanding of the entire trial and all its phases. |

**Table 1 – Context**

**Including (but not limited to) beliefs, social and cultural norms, regulations and economic factors (2,3). For example:**

- Individual capabilities (i.e. values, roles, knowledge, purpose).
- Interpersonal relationships supporting the interventions (i.e. communication, collaboration, network, influences).
- Institutional settings (i.e. informal rules, organisational culture, leadership, policies, resource allocation, local priorities).
- Infra-structural system (i.e. political support).
- Country and rurality (i.e. small or large/rural vs regional vs remote).
- Socio-demographic characteristics:
- Funding context or source (i.e. free, personalised, group vs. individual, government funded).

- The study was conducted in Soweto, South Africa, involving young women within the community as the target population.
- Community Health Workers (CHWs), also known as Health Helpers, were employed to deliver the Bukhali intervention. The education level of these CHWs was not explicitly mentioned in the provided contexts.
- Available resources in the community for the implementation of the intervention included the services provided by CHWs, such as health screening, education, and advocacy for various health conditions like HIV and maternal health-related care.
- Funding for the Bukhali trial was not explicitly discussed in the contexts provided.
- The economic status of the community members, including factors like unemployment and food insecurity, impacted their engagement with the intervention.
- Challenges faced during the implementation included issues like resource limitations, limited availability of supervisors, tensions with facility-based public healthcare, and safety concerns for CHWs in the public health sector.

**Table 2 – Mechanisms**

- **“An element of reasoning and/or reactions of an individual or collective agents(s) in regard of resources available in a given context to bring about changes through the implementation of an intervention” (4). Check what are the mediators to produce outcomes (not only primary, but also secondary or unintended outcomes).**

- The success of the intervention was influenced by the strong relationship of trust between Community Health Workers (CHWs) and participants, which served as a pivotal mechanism for the intervention's impact.
- CHWs' adaptability to individual participant needs and circumstances also played a crucial role in achieving positive outcomes.
- Overcoming health-related stigma within the community helped in the successful implementation of the intervention, highlighting the importance of cultural sensitivity and rapport.
- The credibility, competence, and confidence of CHWs in delivering the intervention effectively contributed to its success.
- Participants feeling valued, supported, and recognized for their efforts during the intervention enhanced engagement and positive outcomes.

**Table 3 – Outcomes**

- **Please make note of all (intended or not) outcomes. Please also classify the role and functions outcomes of CHWs using the following Pillars for people-centred care:**

1. Engaging and empowering people and communities

2. Strengthening governance and accountability (Incl. Supervision, training, and support)

3. Reorienting the model of care

4. Coordinating services within and across sectors

5. Enabling environment

**Outcomes according to PCC pillars:**

- The intervention outcomes were positively influenced by **engaging with participants**, tailoring interventions to individual needs, and fostering trust between Community Health Workers (CHWs) and participants.
- **Governance and accountability** were strengthened through effective supervision, training, and support, ensuring CHWs were well-prepared to deliver the intervention.
- **The model of care** was reoriented to focus on addressing individual participant needs, adapting to healthcare system challenges, and navigating health-related stigma.
- **Services were coordinated** within and across sectors by CHWs, enhancing the continuity of care and support for participants throughout the intervention.
- **An enabling environment** was created by promoting community engagement, addressing socioeconomic constraints, and enhancing the HH-participant relationship, which facilitated the successful implementation of the intervention.

**Table 4 – If, then statements depicting the CMOs.**

**Please detail the CMOs for the study you reviewed using the If (Context), Then (Mechanism) statements.**

**Test for relevance (Pearson et al. 2012; 2015; Brennan et al. 2017)**

| **Conceptually Rich** | **Thicker description’ but not ‘conceptually rich’** | **Conceptually Thin** |
| --- | --- | --- |
| Unambiguous theoretical concepts are described in sufficient depth. | Description of programme theory or sufficient information to enable it to ‘surface’. | Insufficient information to enable the programme theory to surface. |
| Relationships between, amongst concepts are clearly articulated. | Consideration of the context in which the programme takes place. | Limited or no consideration of the context in which the programme took place. |
| Concepts are sufficiently developed, defined to enable understanding without the reader needing to have first-hand experience of an area of practice. | Discussion of the differences between the design and orientation of programme theory (what was intended) and implementation (what really happened). | Limited or no discussion of the differences between the design and orientation of programme theory (what was intended) and implementation (what really happened). |
| Concepts are grounded strongly in a cited body of literature. | Recognition and discussion of the strengths/weaknesses of the implemented programme. | Limited or no discussion of the strengths/ weaknesses of the implemented programme. |
| Concepts are parsimonious (i.e., provide the simplest, but not over-simplified, explanation) | Some attempt to explain anomalous results and findings with reference to context and data. | No attempts to explain anomalous results and findings with reference to context and data. |
|  | Description of the factor affecting implementation. | Limited or no description of the factors affecting implementation. |

**Test for relevance (Pearson et al. 2012; 2015; Brennan et al. 2017)**

| **Conceptually Rich** | **Thicker description’ but not ‘conceptually rich’** | **Conceptually Thin** |
| --- | --- | --- |
|  |  |  |

**Test for rigour (Ohly et al. 2017)**

|  | **Yes** | **Fairly** | **No** |
| --- | --- | --- | --- |
| The study methods are clearly reported. |  |  |  |
| The study methods are appropriate to answer RQ. |  |  |  |
| The sample characteristics enable generalizability. |  |  |  |
| Raw data supports the study findings (conclusions). |  |  |  |
| Limitations of the study are acknowledged and clearly reported. |  |  |  |

**References**

1. Wong G, Westhorp G, Manzano A, Greenhalgh J, Jagosh J, Greenhalgh T. (2016). RAMESES II

reporting standards for realist evaluations. *BMC Medicine*, 14(1), 1-18.

2. Wong G, Greenhalgh T, Westhorp G, Pawson R. (2012). Realist methods in medical education

research: what are they and what can they contribute? *Medical Education*, 46(1), 89-96.

3. Macfarlane F, Greenhalgh T, Humphrey C, Hughes J, Butler C, Pawson R. (2011). A new workforce

in the making?: A case study of strategic human resource management in a whole-system change

effort in healthcare. *Journal of Health Organization and Management*, 25(1), 55-72.

4. Lacouture A, Breton E, Guichard A, Ridde V. (2015). The concept of mechanism from a realist

approach: a scoping review to facilitate its operationalization in public health program evaluation.

*Implementation Science*, 10(1), 1-10.

5. Pearson M, Chilton R, Woods HB, Wyatt K, Ford T, Abraham C, et al. Implementing health promotion in schools: protocol for a realist systematic review of research and experience in the United Kingdom (UK). Syst Rev. 2012 Oct 20;1(1):48.

6. Pearson M, Brand SL, Quinn C, Shaw J, Maguire M, Michie S, et al. Using realist review to inform intervention development: methodological illustration and conceptual platform for collaborative care in offender mental health. Implementation Sci. 2015 Sep 28;10(1):134.

7. Brennan N, Bryce M, Pearson M, Wong G, Cooper C, Archer J. Towards an understanding of how appraisal of doctors produces its effects: a realist review. Medical Education. 2017;51(10):1002–13.

8. Ohly H, Crossland N, Dykes F, Lowe N, Hall-Moran V. A realist review to explore how low-income pregnant women use food vouchers from the UK’s Healthy Start programme. BMJ Open. 2017 Apr 21;7(4):e013731.

**A realist evaluation of the role and functions of community health workers in service of a people-centred community health system.**

**Investigator CMOc Data Extraction Sheet Data Extraction**

The purpose of this document is to extract data from the identified studies in order to formulate one or more explanatory theories that explain Context-Mechanism-Outcome Configurations (CMOC) and to gain insight into how these CMOCs contribute to optimizing the role and functions of community health workers within a people-centred community health system.

In realist methodologies, knowledge accumulation centres on our comprehension of how mechanisms behave in various contexts and the reasons behind how and why this interplay of context and mechanism leads to different outcomes (1). Please fill in the details below for the study you will be assessing. In the subsequent three tables, locate and specify the Context, Mechanism, and Outcomes (CMO) of the intervention discussed in the study you've examined. Definitions and illustrations of CMO are provided within the tables.

**Kindly fill out the information below for the study you will be reviewing.**

| **Reviewer Name** | Usangiphile Buthelezi |
| --- | --- |
| **Reference** | Soepnel LM, Mabetha K, Norris SA, Motlhatlhedi M, Nkosi N, Klingberg S, et al. The role of a community health worker-delivered preconception and pregnancy intervention in achieving a more positive pregnancy experience: the Bukhali trial in Soweto, South Africa. BMC Womens Health. 2024 Mar 5;24(1):161. |
| **Country** | South Africa |
| **Setting and population** | - The research was conducted in Soweto, South Africa. - The study focused on women aged 18-28 who were pregnant or planning to conceive. - Community Health Workers (CHWs) delivered the intervention to these women. |
| **Aim** | The aim of the study was to explore the impact of a community health worker-delivered intervention on the pregnancy experiences of women aged 18-28 in South Africa. |
| **Objectives** | - The objectives of the research were to examine the various factors influencing participants' pregnancy experiences, such as feelings about pregnancy, motherhood responsibilities, health challenges, social support, traumatic experiences, and socioeconomic pressures. - Another objective was to understand how support, information, and care practices provided by CHWs influenced these factors positively. - The study aimed to determine the effectiveness of the intervention in enhancing acceptance, bonding between mother and child, maternal adaptation, health empowerment, and coping mechanisms for challenging circumstances. - Overall, the research sought to show how a comprehensive intervention delivered by CHWs could improve the overall pregnancy experience of young women in the target population. |
| **Methodology** | - The study employed community-based recruitment in Soweto, South Africa, to enroll women aged 18-28 who provided informed consent. - The intervention, called Bukhali, was delivered by Community Health Workers called 'Health Helpers' (HHs) and included various components like Healthy Conversation Skills, trauma-informed care, health literacy materials, multi-micronutrient supplementation, health screening, and ultrasound scans. - Researchers used a phased approach in the trial to assess outcomes at different stages, including preconception, pregnancy, infancy, and early childhood. - Qualitative analysis was conducted to understand the intervention's impact on participants' pregnancy experiences, focusing on support, information, and care practices as the primary pillars. - The study aimed to explore how these intervention components influenced factors such as acceptance, bonding between mother and child, maternal adaptation, health empowerment, and coping mechanisms. |
| **Intervention description** | - The study employed community-based recruitment in Soweto, South Africa, to enroll women aged 18-28 who provided informed consent. - The intervention, called Bukhali, was delivered by Community Health Workers called 'Health Helpers' (HHs) and included various components like Healthy Conversation Skills, trauma-informed care, health literacy materials, multi-micronutrient supplementation, health screening, and ultrasound scans. - Researchers used a phased approach in the trial to assess outcomes at different stages, including preconception, pregnancy, infancy, and early childhood. - Qualitative analysis was conducted to understand the intervention's impact on participants' pregnancy experiences, focusing on support, information, and care practices as the primary pillars. - The study aimed to explore how these intervention components influenced factors such as acceptance, bonding between mother and child, maternal adaptation, health empowerment, and coping mechanisms. |
| **Intervention outcomes** | - The study suggests that a community health worker-delivered intervention can help in enhancing participants' feelings about being pregnant, addressing the responsibilities of motherhood, managing physical and mental health challenges, stabilizing social support, and dealing with traumatic experiences and socioeconomic pressures. - Through the intervention, participants reported acceptance and improved bonding with their child, growth and adaptation in their motherhood role, receiving tools for better health, and developing coping mechanisms for challenging situations. - The outcomes of the community health worker-delivered intervention were closely linked to the participants' context and needs, indicating the effectiveness of personalized support and care practices in achieving a more positive pregnancy experience. |
| **Challenges and limitations** | - The study's interviews conducted between 2-11 months postpartum may have led participants to reflect on their pregnancy experiences through a postpartum lens, potentially affecting their responses. - Exploring participant experiences at various pregnancy stages and disentangling the intervention's impact from the influence of it being their second pregnancy could have provided more specific insights. - The study focused on a directed and applied process evaluation, limiting entirely open, interpretive qualitative analysis and potentially reducing the exploration of pregnancy complexities. - The methodological approach employed in the study provided relevant insights for the research questions but may have restricted a more in-depth analysis of the multifaceted aspects of pregnancy experiences. |

**Table 1 – Context**

**Including (but not limited to) beliefs, social and cultural norms, regulations and economic factors (2,3). For example:**

- Individual capabilities (i.e. values, roles, knowledge, purpose).
- Interpersonal relationships supporting the interventions (i.e. communication, collaboration, network, influences).
- Institutional settings (i.e. informal rules, organisational culture, leadership, policies, resource allocation, local priorities).
- Infra-structural system (i.e. political support).
- Country and rurality (i.e. small or large/rural vs regional vs remote).
- Socio-demographic characteristics:
- Funding context or source (i.e. free, personalised, group vs. individual, government funded).

**Study Setting and Population**

- The study was conducted in Soweto, South Africa, focusing on young women aged 18-28 living in the area.
- Participants were from diverse backgrounds, experiencing challenges like gender-based violence, unemployment, food insecurity, mental health issues, and limited health literacy.

**Available Resources and Economic Status**

- The socioeconomic milieu in Soweto presented challenges such as traumatic events, gender-based violence, unemployment, and food insecurity, impacting the study's participants and intervention.

**Challenges Faced**

Challenges in the community included the convergence of communicable and infectious diseases, high rates of unintended pregnancies, socioeconomic disparities, and gender-based violence, affecting the maternal care landscape in South Africa.

**Table 2 – Mechanisms**

- **“An element of reasoning and/or reactions of an individual or collective agents(s) in regard of resources available in a given context to bring about changes through the implementation of an intervention” (4). Check what are the mediators to produce outcomes (not only primary, but also secondary or unintended outcomes).**

- **Trust and rapport** built between community health workers (CHWs) and participants were crucial for the intervention's success.
- **Empowerment** through tailored support and information helped women grow in their maternal role, fostering a positive pregnancy experience.
- Providing tools for women's health and enhancing their health literacy contributed to their **confidence and autonomy** in decision-making.
- Cultivating a sense of purpose by supporting women in difficult circumstances improved their coping mechanisms and maternal well-being.
- **Recognizing and valuing** the experiences and challenges of participants created a supportive environment that enhanced the effectiveness of the intervention.

**Table 3 – Outcomes**

- **Please make note of all (intended or not) outcomes. Please also classify the role and functions outcomes of CHWs using the following Pillars for people-centred care:**

1. Engaging and empowering people and communities

2. Strengthening governance and accountability (Incl. Supervision, training, and support)

3. Reorienting the model of care

4. Coordinating services within and across sectors

5. Enabling environment

**Outcomes according to PCC pillars:**

- The intervention aimed to **engage and empower** women through tailored support and health information, improving their confidence in decision-making and maternal role.
- To strengthen **governance and accountability,** community health workers received specific training and support tailored to address local contextual needs, enhancing the intervention's effectiveness.
- The **model of care was reoriented** towards providing comprehensive support, including health screening, nutrition counselling, mental health support, and health literacy resources to enhance maternal well-being and pregnancy experiences.
- Services were **coordinated across sectors and within the community,** focusing on addressing women's holistic health needs, such as mental health, nutrition, and physical well-being, to provide comprehensive support during preconception and pregnancy.
- The intervention fostered an **enabling environment** by creating a supportive and understanding atmosphere, recognizing, and valuing participants' experiences, and addressing their unique social and structural realities to improve their pregnancy experiences.

**Table 4 – If, then statements depicting the CMOs.**

**Please detail the CMOs for the study you reviewed using the If (Context), Then (Mechanism) statements.**

**Test for relevance (Pearson et al. 2012; 2015; Brennan et al. 2017)**

| **Conceptually Rich** | **Thicker description’ but not ‘conceptually rich’** | **Conceptually Thin** |
| --- | --- | --- |
| Unambiguous theoretical concepts are described in sufficient depth. | Description of programme theory or sufficient information to enable it to ‘surface’. | Insufficient information to enable the programme theory to surface. |
| Relationships between, amongst concepts are clearly articulated. | Consideration of the context in which the programme takes place. | Limited or no consideration of the context in which the programme took place. |
| Concepts are sufficiently developed, defined to enable understanding without the reader needing to have first-hand experience of an area of practice. | Discussion of the differences between the design and orientation of programme theory (what was intended) and implementation (what really happened). | Limited or no discussion of the differences between the design and orientation of programme theory (what was intended) and implementation (what really happened). |
| Concepts are grounded strongly in a cited body of literature. | Recognition and discussion of the strengths/weaknesses of the implemented programme. | Limited or no discussion of the strengths/ weaknesses of the implemented programme. |
| Concepts are parsimonious (i.e., provide the simplest, but not over-simplified, explanation) | Some attempt to explain anomalous results and findings with reference to context and data. | No attempts to explain anomalous results and findings with reference to context and data. |
|  | Description of the factor affecting implementation. | Limited or no description of the factors affecting implementation. |

**Test for relevance (Pearson et al. 2012; 2015; Brennan et al. 2017)**

| **Conceptually Rich** | **Thicker description’ but not ‘conceptually rich’** | **Conceptually Thin** |
| --- | --- | --- |
|  | **X** |  |

**Test for rigour (Ohly et al. 2017)**

|  | **Yes** | **Fairly** | **No** |
| --- | --- | --- | --- |
| The study methods are clearly reported. | X |  |  |
| The study methods are appropriate to answer RQ. | X |  |  |
| The sample characteristics enable generalizability. |  |  | X |
| Raw data supports the study findings (conclusions). | X |  |  |
| Limitations of the study are acknowledged and clearly reported. | X |  |  |

**References**

1. Wong G, Westhorp G, Manzano A, Greenhalgh J, Jagosh J, Greenhalgh T. (2016). RAMESES II

reporting standards for realist evaluations. *BMC Medicine*, 14(1), 1-18.

2. Wong G, Greenhalgh T, Westhorp G, Pawson R. (2012). Realist methods in medical education

research: what are they and what can they contribute? *Medical Education*, 46(1), 89-96.

3. Macfarlane F, Greenhalgh T, Humphrey C, Hughes J, Butler C, Pawson R. (2011). A new workforce

in the making?: A case study of strategic human resource management in a whole-system change

effort in healthcare. *Journal of Health Organization and Management*, 25(1), 55-72.

4. Lacouture A, Breton E, Guichard A, Ridde V. (2015). The concept of mechanism from a realist

approach: a scoping review to facilitate its operationalization in public health program evaluation.

*Implementation Science*, 10(1), 1-10.

5. Pearson M, Chilton R, Woods HB, Wyatt K, Ford T, Abraham C, et al. Implementing health promotion in schools: protocol for a realist systematic review of research and experience in the United Kingdom (UK). Syst Rev. 2012 Oct 20;1(1):48.

6. Pearson M, Brand SL, Quinn C, Shaw J, Maguire M, Michie S, et al. Using realist review to inform intervention development: methodological illustration and conceptual platform for collaborative care in offender mental health. Implementation Sci. 2015 Sep 28;10(1):134.

7. Brennan N, Bryce M, Pearson M, Wong G, Cooper C, Archer J. Towards an understanding of how appraisal of doctors produces its effects: a realist review. Medical Education. 2017;51(10):1002–13.

8. Ohly H, Crossland N, Dykes F, Lowe N, Hall-Moran V. A realist review to explore how low-income pregnant women use food vouchers from the UK’s Healthy Start programme. BMJ Open. 2017 Apr 21;7(4):e013731.

**A realist evaluation of the role and functions of community health workers in service of a people-centred community health system.**

**Investigator CMOc Data Extraction Sheet Data Extraction**

The purpose of this document is to extract data from the identified studies in order to formulate one or more explanatory theories that explain Context-Mechanism-Outcome Configurations (CMOC) and to gain insight into how these CMOCs contribute to optimizing the role and functions of community health workers within a people-centred community health system.

In realist methodologies, knowledge accumulation centres on our comprehension of how mechanisms behave in various contexts and the reasons behind how and why this interplay of context and mechanism leads to different outcomes (1). Please fill in the details below for the study you will be assessing. In the subsequent three tables, locate and specify the Context, Mechanism, and Outcomes (CMO) of the intervention discussed in the study you've examined. Definitions and illustrations of CMO are provided within the tables.

**Kindly fill out the information below for the study you will be reviewing.**

| **Reviewer Name** | Usangiphile Buthelezi |
| --- | --- |
| **Reference** | Stansert Katzen L, le Roux KW, Almirol E, Hayati Rezvan P, le Roux IM, Mbewu N, et al. Community health worker home visiting in deeply rural South Africa: 12-month outcomes. Glob Public Health. 2021 Nov;16(11):1757–70. |
| **Country** | South Africa |
| **Setting and population** | - The study was conducted in deeply rural areas in the Eastern Cape of South Africa. - Two non-contiguous rural areas of about equal size and density were selected for the study. - The intervention group consisted of mothers in one of the rural areas where community health workers (CHWs) conducted perinatal home visits. - The comparison group consisted of mothers in another rural area who received standard clinic care. - A total of 1469 mother-infant pairs were recruited over a period of 33 months for the study. - The study focused on evaluating the impact of the CHW program on maternal and child outcomes in the rural Eastern Cape region. |
| **Aim** | The aim of the study was to evaluate the impact of a community health worker (CHW) program on maternal and child health outcomes in a deeply rural area of the Eastern Cape, South Africa |
| **Objectives** | - To assess the effectiveness of the CHW program in improving child development and reducing maternal depressive symptoms. - To compare maternal and child outcomes between the intervention group, where CHWs conducted perinatal home visits, and the comparison group, which received standard clinic care. |
| **Methodology** | - The study was a non-randomized comparative cohort study conducted in deeply rural areas of the Eastern Cape, South Africa. - Two non-contiguous rural areas of equal size and density were selected for the study. - A total of 1469 mother-infant pairs were recruited over a period of 33 months. - The intervention group received perinatal home visits by community health workers (CHWs), while the comparison group received standard clinic care. - Maternal and child outcomes were compared between the two groups at one year. - Maternal depressive symptoms were assessed using the Edinburgh Postnatal Depression Scale (EPDS). - Child development was measured by assessing the proportion of developmental milestones achieved. - Maternal and child outcomes were analyzed using student's t-test, chi-square test, Fisher's exact test, and longitudinal mixed models. - Growth measures, immunizations, and breastfeeding practices were also recorded and analyzed. - Baseline demographic and risk characteristics of mothers lost to follow-up were compared to those retained in the study. |
| **Intervention description** | - The intervention involved community health workers (CHWs) conducting perinatal home visits in a deeply rural area of the Eastern Cape, South Africa. - The CHWs, also known as Mentor Mothers, were recruited from the local areas and underwent training before conducting the home visits. - The CHWs provided support and guidance to pregnant women, focusing on addressing maternal depressive symptoms and ensuring children achieved their developmental milestones. - The intervention group received these home visits by CHWs, while the comparison group received standard clinic care. - The effectiveness of the intervention was evaluated by comparing maternal and child outcomes between the intervention and comparison groups at one year. |
| **Intervention outcomes** | - Mothers in the intervention group, who received perinatal home visits by CHWs, had significantly fewer depressive symptoms compared to mothers in the comparison group. - Children of intervention mothers achieved a higher proportion of their developmental milestones compared to children in the comparison group. - There were no other significant differences between the two groups in terms of maternal and child outcomes. - Children in the intervention group were significantly less likely to have a consultation with a private doctor or a private pharmacy visit compared to children in the comparison group. - Children in the intervention group were more likely to be on or ahead of target based on WHO developmental milestones compared to children in the comparison group. - There were no significant differences between the intervention and comparison groups in terms of average weight-for-age z-score (WAZ), height-for-age z-score (HAZ), percentage of low-birth-weight infants, malnourished and stunted babies at birth, and symptoms of illness in the past few weeks. - The intervention showed positive impact on child development and maternal depressive symptoms. |
| **Challenges and limitations** | - The study design was a pragmatic, non-randomized two-group cohort study, which may have introduced unaccounted for differences between the intervention and comparison groups, potentially skewing the results. - There were significant differences between the intervention and comparison areas in terms of access to safe drinking water and electricity, which may have impacted the outcomes. However, these differences were corrected for in the analyses. - The study did not assess the intervention dosage or have access to maternal HIV testing results, relying on self-reporting instead. - Migration of mothers to their parents' homes or to join their husbands working in mines may have impacted the dosage of the intervention, as women who were away missed out on community health worker home visits. - The study was conducted over a relatively short period of 12-18 months, while the Mentor Mother program in Cape Town had been implemented for 10 years before evaluation. It is possible that longer implementation time is needed for interventions to become institutionalized and achieve greater impact. - The study faced challenges in implementing home visiting programs in rural areas, including logistical difficulties due to vast distances and topography, which may have reduced the ability of community health workers to significantly impact households. |

**Table 1 – Context**

**Including (but not limited to) beliefs, social and cultural norms, regulations and economic factors (2,3). For example:**

- Individual capabilities (i.e. values, roles, knowledge, purpose).
- Interpersonal relationships supporting the interventions (i.e. communication, collaboration, network, influences).
- Institutional settings (i.e. informal rules, organisational culture, leadership, policies, resource allocation, local priorities).
- Infra-structural system (i.e. political support).
- Country and rurality (i.e. small or large/rural vs regional vs remote).
- Socio-demographic characteristics:
- Funding context or source (i.e. free, personalised, group vs. individual, government funded).

- The study was conducted in the rural Eastern Cape of South Africa, specifically in the OR Tambo District, which is known to be one of the poorest and most under-developed districts in the country.
- The district has limited access to water, good-quality healthcare, and employment opportunities.
- The study area is characterized by a lack of resources, including limited access to electricity, toilets, and safe water.
- The healthcare services in the area are mainly provided through Zithulele Hospital, a government-run district hospital, and 13 primary healthcare clinics.
- The population in the study area is predominantly rural, with a high prevalence of poverty and limited access to basic amenities.

**Interpersonal relationships supporting the interventions:**

- The study emphasized the importance of community embeddedness, acceptance, and support for the community health worker (CHW) program, which is crucial for program effectiveness.

**Table 2 – Mechanisms**

- **“An element of reasoning and/or reactions of an individual or collective agents(s) in regard of resources available in a given context to bring about changes through the implementation of an intervention” (4). Check what are the mediators to produce outcomes (not only primary, but also secondary or unintended outcomes).**

- The CHW program engaged and empowered mothers in the intervention group by providing perinatal home visits and support.
- CHWs encouraged mothers to play and interact with their children, which may have empowered them to actively participate in their child's development.
- CHWs in the program were recruited from the same communities as the mothers, which may have helped in building rapport and trust.
- CHWs provided daily home visits, allowing for regular and consistent contact with the mothers.
- The CHWs offered support, advice, and guidance to the mothers, which may have motivated them to actively engage in their own and their child's health and well-being.
- The program emphasized the role of CHWs as role models, as they were recruited from the same communities and had similar backgrounds as the mothers.
- CHWs were trained to provide health advice and information, including the importance of breastfeeding, immunizations, and accessing government child grants.
- The program focused on empowering mothers by providing them with knowledge and skills to care for their children, which may have motivated them to take an active role in their child's development.
- The CHWs received daily in-the-field support visits by supervisors, which may have boosted their confidence and provided ongoing guidance and mentorship.
- CHWs in the program were trained over several weeks, which likely helped them develop the necessary skills and knowledge to provide effective care and support to the mothers and children.

**Table 3 – Outcomes**

- **Please make note of all (intended or not) outcomes. Please also classify the role and functions outcomes of CHWs using the following Pillars for people-centred care:**

1. Engaging and empowering people and communities

2. Strengthening governance and accountability (Incl. Supervision, training, and support)

3. Reorienting the model of care

4. Coordinating services within and across sectors

5. Enabling environment

- Mothers in the intervention group, who received perinatal home visits by CHWs, had significantly fewer depressive symptoms compared to mothers in the comparison group.
- Children of intervention mothers achieved a higher proportion of their developmental milestones compared to children in the comparison group.
- There were no other significant differences between the two groups in terms of maternal and child outcomes.
- Children in the intervention group were significantly less likely to have a consultation with a private doctor or a private pharmacy visit compared to children in the comparison group.
- Children in the intervention group were more likely to be on or ahead of target based on WHO developmental milestones compared to children in the comparison group.
- There were no significant differences between the intervention and comparison groups in terms of average weight-for-age z-score (WAZ), height-for-age z-score (HAZ), percentage of low-birth-weight infants, malnourished and stunted babies at birth, and symptoms of illness in the past few weeks.
- The intervention showed positive impact on child development and maternal depressive symptoms.

**Intervention outcomes by PCC pillars:**

**Engaging and empowering people and communities:**

- The CHW program engaged and empowered mothers in the intervention group by providing perinatal home visits and support.
- CHWs encouraged mothers to play and interact with their children, which may have empowered them to actively participate in their child's development.

**Strengthening governance and accountability (Incl. Supervision, training, and support):**

- The CHW program involved the training and support of CHWs who conducted the perinatal home visits.
- The program emphasized the importance of effective CHW supervision, which is crucial for program effectiveness.

**Reorienting the model of care:**

- The CHW program provided a different model of care by offering perinatal home visits, which complemented the standard clinic care received by the comparison group.
- The program aimed to shift tasks from healthcare professionals to CHWs, potentially improving access to care and health outcomes.

**Coordinating services within and across sectors:**

- The CHW program integrated with existing primary healthcare services, including antenatal care and HIV care, provided at clinics and a hospital.
- CHWs worked in collaboration with healthcare professionals to support mothers and children in the intervention group.

**Enabling environment:**

- The CHW program operated in a deeply rural context, where access to healthcare services and resources, such as safe drinking water and electricity, varied between the intervention and comparison areas.
- The program faced logistical difficulties due to the vast distances and topography of the rural areas, which may have impacted the effectiveness of CHW home visits.

**Table 4 – If, then statements depicting the CMOs.**

**Please detail the CMOs for the study you reviewed using the If (Context), Then (Mechanism) statements.**

**If, Then statements**

- If CHWs are recruited from the same communities as the people they serve, then CHWs are perceived as role models, as they are recruited from the same communities and have similar backgrounds as the people they serve, fostering rapport and trust, which can lead to enhanced acceptance of healthcare messages and services, increased community engagement in health initiatives, and improved health outcomes overall.
- If CHWs provide support, advice, and guidance to the mothers, this motivates them to actively engage in their own and their child’s health and well-being, leading to improved health-seeking behaviors, better adherence to healthcare recommendations, and ultimately, enhanced health outcomes for both the mothers and their children.

**Test for relevance (Pearson et al. 2012; 2015; Brennan et al. 2017)**

| **Conceptually Rich** | **Thicker description’ but not ‘conceptually rich’** | **Conceptually Thin** |
| --- | --- | --- |
| Unambiguous theoretical concepts are described in sufficient depth. | Description of programme theory or sufficient information to enable it to ‘surface’. | Insufficient information to enable the programme theory to surface. |
| Relationships between, amongst concepts are clearly articulated. | Consideration of the context in which the programme takes place. | Limited or no consideration of the context in which the programme took place. |
| Concepts are sufficiently developed, defined to enable understanding without the reader needing to have first-hand experience of an area of practice. | Discussion of the differences between the design and orientation of programme theory (what was intended) and implementation (what really happened). | Limited or no discussion of the differences between the design and orientation of programme theory (what was intended) and implementation (what really happened). |
| Concepts are grounded strongly in a cited body of literature. | Recognition and discussion of the strengths/weaknesses of the implemented programme. | Limited or no discussion of the strengths/ weaknesses of the implemented programme. |
| Concepts are parsimonious (i.e., provide the simplest, but not over-simplified, explanation) | Some attempt to explain anomalous results and findings with reference to context and data. | No attempts to explain anomalous results and findings with reference to context and data. |
|  | Description of the factor affecting implementation. | Limited or no description of the factors affecting implementation. |

**Test for relevance (Pearson et al. 2012; 2015; Brennan et al. 2017)**

| **Conceptually Rich** | **Thicker description’ but not ‘conceptually rich’** | **Conceptually Thin** |
| --- | --- | --- |
| X |  |  |

**Test for rigour (Ohly et al. 2017)**

|  | **Yes** | **Fairly** | **No** |
| --- | --- | --- | --- |
| The study methods are clearly reported. | X |  |  |
| The study methods are appropriate to answer RQ. | X |  |  |
| The sample characteristics enable generalizability. |  |  | X |
| Raw data supports the study findings (conclusions). | X |  |  |
| Limitations of the study are acknowledged and clearly reported. | X |  |  |

**References**

1. Wong G, Westhorp G, Manzano A, Greenhalgh J, Jagosh J, Greenhalgh T. (2016). RAMESES II

reporting standards for realist evaluations. *BMC Medicine*, 14(1), 1-18.

2. Wong G, Greenhalgh T, Westhorp G, Pawson R. (2012). Realist methods in medical education

research: what are they and what can they contribute? *Medical Education*, 46(1), 89-96.

3. Macfarlane F, Greenhalgh T, Humphrey C, Hughes J, Butler C, Pawson R. (2011). A new workforce

in the making?: A case study of strategic human resource management in a whole-system change

effort in healthcare. *Journal of Health Organization and Management*, 25(1), 55-72.

4. Lacouture A, Breton E, Guichard A, Ridde V. (2015). The concept of mechanism from a realist

approach: a scoping review to facilitate its operationalization in public health program evaluation.

*Implementation Science*, 10(1), 1-10.

5. Pearson M, Chilton R, Woods HB, Wyatt K, Ford T, Abraham C, et al. Implementing health promotion in schools: protocol for a realist systematic review of research and experience in the United Kingdom (UK). Syst Rev. 2012 Oct 20;1(1):48.

6. Pearson M, Brand SL, Quinn C, Shaw J, Maguire M, Michie S, et al. Using realist review to inform intervention development: methodological illustration and conceptual platform for collaborative care in offender mental health. Implementation Sci. 2015 Sep 28;10(1):134.

7. Brennan N, Bryce M, Pearson M, Wong G, Cooper C, Archer J. Towards an understanding of how appraisal of doctors produces its effects: a realist review. Medical Education. 2017;51(10):1002–13.

8. Ohly H, Crossland N, Dykes F, Lowe N, Hall-Moran V. A realist review to explore how low-income pregnant women use food vouchers from the UK’s Healthy Start programme. BMJ Open. 2017 Apr 21;7(4):e013731.

**A realist evaluation of the role and functions of community health workers in service of a people-centred community health system.**

**Investigator CMOc Data Extraction Sheet Data Extraction**

The purpose of this document is to extract data from the identified studies in order to formulate one or more explanatory theories that explain Context-Mechanism-Outcome Configurations (CMOC) and to gain insight into how these CMOCs contribute to optimizing the role and functions of community health workers within a people-centred community health system.

In realist methodologies, knowledge accumulation centres on our comprehension of how mechanisms behave in various contexts and the reasons behind how and why this interplay of context and mechanism leads to different outcomes (1). Please fill in the details below for the study you will be assessing. In the subsequent three tables, locate and specify the Context, Mechanism, and Outcomes (CMO) of the intervention discussed in the study you've examined. Definitions and illustrations of CMO are provided within the tables.

**Kindly fill out the information below for the study you will be reviewing.**

| **Reviewer Name** | Usangiphile Buthelezi |
| --- | --- |
| **Reference** | Tinago CB, Frongillo EA, Warren AM, Chitiyo V, Jackson TN, Cifarelli AK, et al. Testing the Effectiveness of a Community-Based Peer Support Intervention to Mitigate Social Isolation and Stigma of Adolescent Motherhood in Zimbabwe. Matern Child Health J. 2024 Apr;28(4):657–66. |
| **Country** | Zimbabwe |
| **Setting and population** |  |
| **Aim** | - The aim of the study was to test how effective a peer support intervention could be in helping adolescent mothers in Zimbabwe who face social isolation and stigma.   - This means the researchers wanted to see if the peer support groups could make a difference in the lives of young mothers who don't have much social support or feel judged by others. |
| **Objectives** | - The objective was to assess various aspects like mental health outcomes and social support of the adolescent mothers involved in the study.   - The study looked at things like depressive symptoms, common mental disorders, and the levels of support the young mothers felt from their family, friends, and significant others.   - They wanted to see if the intervention could help these young mothers feel less alone, more supported, and better equipped to handle the challenges of motherhood. - Another objective was to involve community members and key stakeholders to monitor progress and provide suggestions for improving the health of the young mothers.   - This means they wanted to work closely with people in the community and others who could give valuable input to make sure the intervention was going well and making a positive impact. - Through these aims and objectives, the study aimed to show that a community-based peer support intervention could make a real difference in the lives of adolescent mothers in Zimbabwe, helping them feel more supported, less isolated, and better able to cope with the challenges they face. |
| **Methodology** | - The study aimed to test the effectiveness of a peer support intervention for adolescent mothers in Zimbabwe.   - Community health workers and peer educators were trained to lead peer support groups for adolescent mothers aged 15-18 from low-income communities in Harare.   - The intervention involved 104 participants in the intervention arm and 79 in the control arm, following a quasi-experimental design. - Data collection and analysis methods:   - Pre-intervention and post-intervention surveys were conducted to assess outcomes related to mental health and social support.   - Surveys included measures of depressive symptoms, common mental disorders, and levels of overall, family, friends, and significant-other support.   - Stata 15 software was utilized for data analysis to compare outcomes between the intervention and control groups. |
| **Intervention description** | - Peer support groups met in-person twice a month, focusing on participant-identified topics like income generation and depression. - WhatsApp Messenger was used for training facilitators and providing support during the intervention. - Key stakeholders were involved in discussions to monitor project progress and provide recommendations for improving adolescent mothers' health. |
| **Intervention outcomes** | - The intervention arm reported lower depressive symptoms and common mental disorders compared to the control arm. - Participants in the intervention arm felt more supported, engaged with peers, and had increased knowledge about resources for help, coping strategies, and parenting skills. - The intervention successfully mitigated social isolation and stigma, leading to improved mental health and social support among adolescent mothers. - The intervention successfully engaged adolescent mothers in peer support groups, providing a sense of community and support. - Training and empowering community health workers and peer educators played a crucial role in the intervention's effectiveness. - Regular supervision, training, and support ensured governance and accountability, maintaining the quality of the intervention. - By focusing on peer support and addressing participant-identified topics, the intervention reoriented the model of care to meet the unique needs of adolescent mothers. - Coordinating services within the community and involving key stakeholders ensured a comprehensive approach to supporting adolescent mothers. - Using WhatsApp for facilitator training and support enhanced communication and coordination within the intervention, creating an enabling environment. - Factors like trust, rapport, empowerment, credibility, feeling valued, and recognition contributed to the success of the intervention. - Challenges such as lack of community engagement, insufficient resources, cultural barriers, and communication issues could potentially impact the intervention's effectiveness. - Addressing the mental health outcomes and social support needs of adolescent mothers in Zimbabwe, the intervention aimed to mitigate social isolation and stigma effectively. |
| **Challenges and limitations** | - Limited generalizability: The study was conducted in specific low-income high-density communities in Harare, Zimbabwe, potentially limiting how broadly the results can be applied to different settings. - Small sample size: The number of participants in the intervention arm (n = 104) and control arm (n = 79) may impact the statistical power and the ability to draw definitive conclusions. - Lack of long-term follow-up: The study's duration may not have been sufficient to assess the intervention's sustained effects over a longer period, potentially missing changes that occur beyond the study timeframe. - Self-reporting biases: The reliance on participants' self-reported data in surveys may introduce response biases or inaccuracies in reporting mental health outcomes and social support levels. - Quasi-experimental design: While the study used a quasi-experimental design, it may not provide the same level of rigor as a randomized controlled trial, potentially affecting the strength of the study findings. - Intervention implementation challenges: Factors such as community engagement issues, resource constraints, cultural differences, and communication barriers could have influenced the intervention's delivery and outcomes. - Potential confounding variables: Variables not accounted for in the study, such as other support services accessed concurrently by participants, could impact the observed outcomes, potentially confounding the results. - Social desirability bias: Participants may have responded in ways they deemed socially acceptable rather than providing genuine responses, leading to a bias in the reported outcomes. - Ethical considerations: Ensuring ethical conduct throughout the study, including issues related to confidentiality, privacy, and consent, is essential but could pose challenges in a community-based intervention. |

**Table 1 – Context**

**Including (but not limited to) beliefs, social and cultural norms, regulations and economic factors (2,3). For example:**

- Individual capabilities (i.e. values, roles, knowledge, purpose).
- Interpersonal relationships supporting the interventions (i.e. communication, collaboration, network, influences).
- Institutional settings (i.e. informal rules, organisational culture, leadership, policies, resource allocation, local priorities).
- Infra-structural system (i.e. political support).
- Country and rurality (i.e. small or large/rural vs regional vs remote).
- Socio-demographic characteristics:
- Funding context or source (i.e. free, personalised, group vs. individual, government funded).

**Study Setting and Population**

- The study took place in Harare, Zimbabwe.
- It focused on adolescent mothers aged 15-18 years from low-income high-density communities.
- Community health workers (CHWs) and peer educators were trained to support these young mothers.

**Training and Education Levels**

- CHWs had varying education levels, with most having a grade 7 education.
- Peer educators mostly had a high school education between Form 1-4.

**Available Resources and Facilities**

- The intervention included peer support groups that met in-person twice a month.
- WhatsApp Messenger was utilized for training and support communication.
- Key community stakeholders were involved in discussions to enhance project progress and recommendations.

**Funding and Economic Status**

- The specific funding details are not provided in the contexts.
- The study focused on adolescent mothers from low-income communities, implying a challenging economic status.

**Challenges and Contextual Issues**

- Adolescent mothers often face social isolation and stigma.
- Lack of social support and resources makes navigating motherhood difficult.
- There was a need for interventions tailored to the unique needs of adolescent mothers.

**Education Level of Community Health Workers**

- Most CHWs had a grade 7 education, highlighting the need for training to equip them with skills to support adolescent mothers effectively.

**Table 2 – Mechanisms**

- **“An element of reasoning and/or reactions of an individual or collective agents(s) in regard of resources available in a given context to bring about changes through the implementation of an intervention” (4). Check what are the mediators to produce outcomes (not only primary, but also secondary or unintended outcomes).**

- **Trust and Rapport**:
  - Building trust and rapport between the adolescent mothers and the facilitators helped create a safe and supportive environment for sharing experiences and seeking help.
- **Empowerment and Motivation**:
  - Empowering the young mothers through peer support groups motivated them to address challenges, build resilience, and engage positively with their circumstances.
- **Credibility and Competence**:
  - Facilitators' credibility and competence in addressing the needs of adolescent mothers instilled confidence and encouraged active participation in the intervention.
- **Feeling Valued and Supported**:
  - Making the participants feel valued and supported by peers and significant adults enhanced their sense of belonging and acceptance.
- **Recognition and Sense of Purpose**:
  - Acknowledging the participants' efforts and providing them with a sense of purpose within the support groups fostered a positive outlook and encouraged continued engagement.

**Challenges Leading to Potential Intervention Failures**

- **Lack of Community Engagement**:
  - Limited community involvement and engagement may hinder the sustainability and reach of the intervention among adolescent mothers.
- **Insufficient Training and Resources**:
  - Inadequate training and resources for facilitators could lead to ineffective support delivery and limit the intervention's impact.
- **Cultural Barriers**:
  - Cultural norms and stigmas surrounding adolescent motherhood may pose challenges in effectively addressing the needs and experiences of the participants.
- **Communication Barriers**:
  - Poor communication channels or misunderstandings between facilitators and participants could impede trust-building and support provision within the intervention.

**Table 3 – Outcomes**

- **Please make note of all (intended or not) outcomes. Please also classify the role and functions outcomes of CHWs using the following Pillars for people-centred care:**

1. Engaging and empowering people and communities

2. Strengthening governance and accountability (Incl. Supervision, training, and support)

3. Reorienting the model of care

4. Coordinating services within and across sectors

5. Enabling environment

- The intervention successfully engaged adolescent mothers in peer support groups, providing a sense of community and support.
- Training and empowering community health workers and peer educators played a crucial role in the intervention's effectiveness.
- Regular supervision, training, and support ensured governance and accountability, maintaining the quality of the intervention.
- By focusing on peer support and addressing participant-identified topics, the intervention reoriented the model of care to meet the unique needs of adolescent mothers.
- Coordinating services within the community and involving key stakeholders ensured a comprehensive approach to supporting adolescent mothers.
- Using WhatsApp for facilitator training and support enhanced communication and coordination within the intervention, creating an enabling environment.
- Factors like trust, rapport, empowerment, credibility, feeling valued, and recognition contributed to the success of the intervention.
- Challenges such as lack of community engagement, insufficient resources, cultural barriers, and communication issues could potentially impact the intervention's effectiveness.
- Addressing the mental health outcomes and social support needs of adolescent mothers in Zimbabwe, the intervention aimed to mitigate social isolation and stigma effectively.
- The intervention arm reported lower depressive symptoms and common mental disorders compared to the control arm.
- Participants in the intervention arm felt more supported, engaged with peers, and had increased knowledge about resources for help, coping strategies, and parenting skills.
- The intervention successfully mitigated social isolation and stigma, leading to improved mental health and social support among adolescent mothers.

**Outcomes according to PCC pillars:**

- The intervention successfully **engaged** adolescent mothers in peer support groups, fostering a sense of community and support.
- Training and **empowering community** health workers and peer educators contributed to the effectiveness of the intervention.
- **Governance and accountability** were maintained through regular supervision, training, and support provided to facilitators, ensuring the quality of the intervention.
- By **reorienting the model of care** to focus on peer support and addressing participant-identified topics, the intervention tailored its approach to the unique needs of adolescent mothers.
- **Coordinating services** within the community and involving key stakeholders ensured a holistic approach to supporting adolescent mothers.
- The intervention created **an enabling environment** by utilizing WhatsApp for facilitator training and implementation support, enhancing communication, and coordination within the intervention.
- By addressing these aspects, the intervention effectively mitigated social isolation and stigma, leading to improved mental health and social support among adolescent mothers in Zimbabwe.

**Table 4 – If, then statements depicting the CMOs.**

**Please detail the CMOs for the study you reviewed using the If (Context), Then (Mechanism) statements.**

**IF** the intervention successfully engages adolescent mothers in peer support groups, community health workers and peer educators are well-trained and empowered, regular supervision, training, and support are provided to facilitators, the model of care is reoriented to focus on peer support and participant-identified topics, services are coordinated within the community and key stakeholders are involved, and WhatsApp is utilized for facilitator training and implementation support, **THEN** a sense of community and support will be fostered, the effectiveness of the intervention will increase, governance and accountability will be maintained, the unique needs of adolescent mothers will be better addressed, a holistic approach to support will be ensured, and communication and coordination within the intervention will be enhanced **BECAUSE** participants will benefit from shared experiences and mutual encouragement, trained and empowered workers can provide relevant and impactful support, ongoing oversight ensures consistent quality, a customized approach meets specific circumstances and concerns, comprehensive support addresses multiple aspects of well-being, and digital tools facilitate timely and efficient information sharing and support.

**Test for relevance (Pearson et al. 2012; 2015; Brennan et al. 2017)**

| **Conceptually Rich** | **Thicker description’ but not ‘conceptually rich’** | **Conceptually Thin** |
| --- | --- | --- |
| Unambiguous theoretical concepts are described in sufficient depth. | Description of programme theory or sufficient information to enable it to ‘surface’. | Insufficient information to enable the programme theory to surface. |
| Relationships between, amongst concepts are clearly articulated. | Consideration of the context in which the programme takes place. | Limited or no consideration of the context in which the programme took place. |
| Concepts are sufficiently developed, defined to enable understanding without the reader needing to have first-hand experience of an area of practice. | Discussion of the differences between the design and orientation of programme theory (what was intended) and implementation (what really happened). | Limited or no discussion of the differences between the design and orientation of programme theory (what was intended) and implementation (what really happened). |
| Concepts are grounded strongly in a cited body of literature. | Recognition and discussion of the strengths/weaknesses of the implemented programme. | Limited or no discussion of the strengths/ weaknesses of the implemented programme. |
| Concepts are parsimonious (i.e., provide the simplest, but not over-simplified, explanation) | Some attempt to explain anomalous results and findings with reference to context and data. | No attempts to explain anomalous results and findings with reference to context and data. |
|  | Description of the factor affecting implementation. | Limited or no description of the factors affecting implementation. |

**Test for relevance (Pearson et al. 2012; 2015; Brennan et al. 2017)**

| **Conceptually Rich** | **Thicker description’ but not ‘conceptually rich’** | **Conceptually Thin** |
| --- | --- | --- |
|  | **X** |  |

**Test for rigour (Ohly et al. 2017)**

|  | **Yes** | **Fairly** | **No** |
| --- | --- | --- | --- |
| The study methods are clearly reported. | X |  |  |
| The study methods are appropriate to answer RQ. | X |  |  |
| The sample characteristics enable generalizability. |  |  | X |
| Raw data supports the study findings (conclusions). | X |  |  |
| Limitations of the study are acknowledged and clearly reported. | X |  |  |

**References**

1. Wong G, Westhorp G, Manzano A, Greenhalgh J, Jagosh J, Greenhalgh T. (2016). RAMESES II

reporting standards for realist evaluations. *BMC Medicine*, 14(1), 1-18.

2. Wong G, Greenhalgh T, Westhorp G, Pawson R. (2012). Realist methods in medical education

research: what are they and what can they contribute? *Medical Education*, 46(1), 89-96.

3. Macfarlane F, Greenhalgh T, Humphrey C, Hughes J, Butler C, Pawson R. (2011). A new workforce

in the making?: A case study of strategic human resource management in a whole-system change

effort in healthcare. *Journal of Health Organization and Management*, 25(1), 55-72.

4. Lacouture A, Breton E, Guichard A, Ridde V. (2015). The concept of mechanism from a realist

approach: a scoping review to facilitate its operationalization in public health program evaluation.

*Implementation Science*, 10(1), 1-10.

5. Pearson M, Chilton R, Woods HB, Wyatt K, Ford T, Abraham C, et al. Implementing health promotion in schools: protocol for a realist systematic review of research and experience in the United Kingdom (UK). Syst Rev. 2012 Oct 20;1(1):48.

6. Pearson M, Brand SL, Quinn C, Shaw J, Maguire M, Michie S, et al. Using realist review to inform intervention development: methodological illustration and conceptual platform for collaborative care in offender mental health. Implementation Sci. 2015 Sep 28;10(1):134.

7. Brennan N, Bryce M, Pearson M, Wong G, Cooper C, Archer J. Towards an understanding of how appraisal of doctors produces its effects: a realist review. Medical Education. 2017;51(10):1002–13.

8. Ohly H, Crossland N, Dykes F, Lowe N, Hall-Moran V. A realist review to explore how low-income pregnant women use food vouchers from the UK’s Healthy Start programme. BMJ Open. 2017 Apr 21;7(4):e013731.
