# Supplementary material for: Optimizing the role and functions of CHWs in service of a people-centred community health system in sub-Saharan Africa. A realist synthesis
Source: SSM Health Syst. 2025 Dec;5:100089. doi: 10.1016/j.ssmhs.2025.100089 (PMC12678229; doi:10.1016/j.ssmhs.2025.100089)
Supplement: Supplementary file 7 — Supplementary material [file mmc7.docx]

| **PROGRAMME THEORIES FOR THE ROLES AND FUNCTIONS OF CHWs UNDER THE IPCHS FRAMEWORK FOR PEOPLE-CENTRED CARE** | | | | |
| --- | --- | --- | --- | --- |
| **IPCHS framework strategies** | **Context ("If")** | **Mechanism ("Then")** | **Outcome** | **Source** |
| **Engaging and empowering people and communities** | **IF** Community Health Workers are familiar faces in the community, influential members, accessible, friendly, and are involved in the community healthcare initiatives. | **THEN** they will build trust, rapport, and credibility with the community. | leading to enhanced community engagement, and empowerment, acceptance of health interventions, and improving overall health outcomes. | (1–11) |
|  | **IF** CHWs involve community members in designing and producing communication materials. | **THEN** the community will develop a sense of ownership and empowerment | and there will be improved acceptance and uptake of healthcare services. | (12–15) |
|  | **IF** CHWs are selected through a community-based participatory process, they are role models within the community and recruited from the same communities as the people they serve. | **THEN** CHWs will be respected and accepted, fostering trust, cultural competency, and understanding of local needs | This will result in individualized care, CHW retention and commitment, acceptance of health messages. | (2–4,12,16,17) |
|  | **IF** CHWs offer training and health education to community members (Informal carers). | **THEN** community members will feel empowered. | leading to increased awareness, improved health literacy, enhanced self-efficacy, and positive behaviour change. | (1,2,18) |
|  | **IF** CHWs have personal experiences related to patient conditions either through their own lives or within their communities. | **THEN** CHWs can show empathy and develop meaningful connections. Fostering trust, and increase confidence and motivation for the patients to engage in care | This leads to increased adoption of healthier behaviours, individualized care, and greater engagement with CHWs for support and adherence to treatment plans. | (2,19–22) |
| **Strengthening governance and accountability** | **IF** CHWs operate within the scope of work defined by the Ministry of Health. | **THEN** there is a sense of accountability among CHWs as they deliver services according to established guidelines. | leading to alignment with the Ministry of Health objectives and  enhancing service quality and  efficiency. | (2) |
|  | **IF** CHWs receive support from the government to undertake their community-based tasks (e.g., equipment, stationery, uniform, or funds for transport or communication). | **THEN** CHWs will feel motivated to perform their roles and responsibilities, | leading to the provision of care that is comprehensive and covers a broader range of health needs. | (2,23,24) |
|  | **IF** CHWs receive regular technical mentorship, supervision, support, and ongoing intensive training by health service professionals and government officials, to equip them with the necessary knowledge, skills and resources to perform their duties, | **THEN** CHWs will feel supported, motivated, accountable, confident, and competent to perform their duties effectively | leading to improved retention, enhanced performance, high-quality care, and effective delivery of services. BECAUSE they will have the necessary guidance, resources, oversight, and preparation | (4,6–8,19,20,23–30) |
|  | **IF** CHWs face contextual challenges such as late payment of salaries, poor remuneration, and dissatisfaction with their working conditions**.** | THEN CHWs will be demotivated, reluctant to take on new tasks and to perform their roles and responsibilities | leading to poor performance and delivery of services to the community. | (6,7,16,19,30,31) |
|  | **IF** there are clear national policies that ensure fair and consistent salaries and support for CHWs. | **THEN** CHWs will feel appreciated, valued and motivated to carry out their responsibilities effectively, | leading to improved performance in delivering healthcare services. | (6,7,16) |
| **Reorienting the model of care** | **IF** CHWs are integrated into the formal health system with clearly defined roles and responsibilities; are directly employed by the Ministry of Health; and understand the policy framework governing their roles | **THEN** they feel valued and recognized as integral members of the healthcare system, assure communities that the care provided meets quality and standard requirements set by the health system (trust), and develop a strong sense of purpose and clarity in their work. | This results into greater acceptability and utilization of healthcare services by the community, stable and dedicated healthcare workforce and consistent healthcare delivery to communities, and improved performance and effective delivery of healthcare services that meet community needs. | (5–7,10,26,27) |
|  | **IF** the model of care integrates community support structures and families, and provide support at an interpersonal level | **THEN** this fosters stronger community bonds, trust, rapport, and recognition between the community and CHWs | leading to improved health outcomes. | (8,21,30) |
|  | IF there is gender matching between CHWs and patients, particularly when addressing sensitive healthcare topics (i.e. gender-sensitive topics like reproductive health, family planning, and maternal care). | **THEN** patients will choose CHWs based on their comfort, trust, and cultural norms | leading to enhanced  acceptability of CHW services within the community, reduced stigma, and improved community engagement. | (32) |
| **Coordinating services within and across sectors** | **IF** Community Health Workers have a good relationship with professional health staff, formal links to clinics, leadership support, structured management, and regular interactions with supervisors | **THEN** CHWs will be motivated, and develop morale, competence, and confidence to perform their duties | leading to improved retention, performance, service delivery, and better healthcare outcomes. | (2,4,10,12,19,20,22–24,27,30,33) |
|  | **IF** various sectors such as social services, and other civil society organizations work with CHWs | **THEN** CHWs will feel supported and provide comprehensive care | leading to high quality and holistic care**.** | (8,23,34) |
| **Creating an enabling Environment** | **IF** there is a supportive learning environment for Community Health Workers that includes regular spaces for dialogue, peer support, and mutual learning, and CHWs engage their clients in safe spaces (places preferred by individual clients – including location and time). | **THEN** CHWs will gain tools and skills to rework their agency in more empowered ways, leading to improved communication and better relationships amongst themselves and with the communities they serve, while also creating a supportive and familiar environment for clients fosters open communication and trust. | This results in a better understanding of context, improved disclosure, enhanced participation, and individualized care. | (14,30,35,36) |
|  | **IF** there is flexible management (i.e. management that allows innovation, is responsive, supportive, empowering, and adapts to change) of CHWs by senior staff | **THEN** CHWs will feel valued, heard and supported | leading to improvements in care practices, enhanced CHW performance, and better alignment of services with community needs. | (6,7,21,23,36) |

**References**

1. Pollard R, Kennedy CE, Hutton HE, Mulamba J, Mbabali I, Anok A, et al. HIV Prevention and Treatment Behavior Change and the Situated Information Motivation Behavioral Skills (sIMB) Model: A Qualitative Evaluation of a Community Health Worker Intervention in Rakai, Uganda. AIDS Behav. 2022 Feb;26(2):375–84.

2. Adam MB, Dillmann M, Chen M kuang, Mbugua S, Ndung’u J, Mumbi P, et al. Improving Maternal and Newborn Health: Effectiveness of a Community Health Worker Program in Rural Kenya. PLoS One. 2014 Aug 4;9(8):e104027.

3. Stansert Katzen L, le Roux KW, Almirol E, Hayati Rezvan P, le Roux IM, Mbewu N, et al. Community health worker home visiting in deeply rural South Africa: 12-month outcomes. Glob Public Health. 2021 Nov;16(11):1757–70.

4. le Roux K, le Roux I, Mbewu N, Davis E. The Role of Community Health Workers in the Re-Engineering of Primary Health Care in Rural Eastern Cape. S Afr Fam Pract (2004). 2015 Mar 1;57(2):116–20.

5. Murphy JP, Moolla A, Kgowedi S, Mongwenyana C, Mngadi S, Ngcobo N, et al. Community health worker models in South Africa: a qualitative study on policy implementation of the 2018/19 revised framework. Health Policy and Planning. 2021 May 1;36(4):384–96.

6. Goudge J, Babalola O, Malatji H, Levin J, Thorogood M, Griffiths F. The effect of a roving nurse mentor on household coverage and quality of care provided by community health worker teams in South Africa: a longitudinal study with a before, after and 6 months post design. BMC health services research. 2023 Feb 22;23(1):186.

7. Malatji H, Griffiths F, Goudge J. Supportive supervision from a roving nurse mentor in a community health worker programme: a process evaluation in South Africa. BMC health services research. 2022 Mar 10;22(1):323.

8. Yuh MN, Ndum Okwen GA, Miong RHP, Bragazzi NL, Kong JD, Movahedi Nia Z, et al. Using an innovative family-centered evidence toolkit to improve the livelihood of people with disabilities in Bamenda (Cameroon): a mixed-method study. Front Public Health. 2023;11:1190722.

9. Olakkengil M, Said S, Abdalla O, Hofmann R, Hedt-Gauthier B, Fulcher I. Are populations of postpartum women differentially served by community health worker programs: an observational cohort study from Zanzibar, Tanzania. BMC Pregnancy Childbirth. 2024 Mar 7;24(1):183.

10. Youngui BT, Atwine D, Otai D, Vasiliu A, Ssekyanzi B, Sih C, et al. Integration of HIV Testing in a Community Intervention for Tuberculosis Screening Among Household Contacts of Patients with Tuberculosis in Cameroon and Uganda. J Acquir Immune Defic Syndr. 2024 Apr 15;95(5):431–8.

11. Soepnel LM, Norris SA, Mabetha K, Motlhatlhedi M, Nkosi N, Lye S, et al. A qualitative analysis of community health worker perspectives on the implementation of the preconception and pregnancy phases of the Bukhali randomised controlled trial. PLOS Glob Public Health. 2024;4(3):e0002578.

12. Abbey M, Bartholomew LK, Nonvignon J, Chinbuah MA, Pappoe M, Gyapong M, et al. Factors related to retention of community health workers in a trial on community-based management of fever in children under 5 years in the Dangme West District of Ghana. Int Health. 2014 Jun;6(2):99–105.

13. Abbey M, Bartholomew LK, Pappoe M, van den Borne B. Treating fever in children under 5 years of age: caregiver perceptions of community health worker services in Dangme West district, Ghana. Int Health. 2015 Nov;7(6):455–63.

14. D’Ambruoso L, Abruquah NA, Mabetha D, van der Merwe M, Goosen G, Sigudla J, et al. Expanding Community Health Worker decision space: learning from a Participatory Action Research training intervention in a rural South African district. Human resources for health. 2023 Aug 18;21(1):66.

15. Jenson A, Roter DL, Mkocha H, Munoz B, West S. Patient-centered communication of community treatment assistants in Tanzania predicts coverage of future mass drug administration for trachoma. Patient Educ Couns. 2018 Jun;101(6):1075–81.

16. Rogers A, Goore LL, Wamae J, Starnes JR, Okong’o SO, Okoth V, et al. Training and experience outperform literacy and formal education as predictors of community health worker knowledge and performance, results from Rongo sub-county, Kenya. Front Public Health [Internet]. 2023 Apr 27 [cited 2024 Apr 3];11. Available from: https://www.frontiersin.org/journals/public-health/articles/10.3389/fpubh.2023.1120922/full

17. Mendin SF, Krause JA, Gweh A, Baysah M, Nyumah J, Gaye CJ, et al. Measuring health system responsiveness in a national community health worker primary care programme in rural Liberia. Int J Qual Health Care. 2023 May 17;35(2):mzad027.

18. Soepnel LM, Mabetha K, Norris SA, Motlhatlhedi M, Nkosi N, Klingberg S, et al. The role of a community health worker-delivered preconception and pregnancy intervention in achieving a more positive pregnancy experience: the Bukhali trial in Soweto, South Africa. BMC Womens Health. 2024 Mar 5;24(1):161.

19. Busza J, Dauya E, Bandason T, Simms V, Chikwari CD, Makamba M, et al. The role of community health workers in improving HIV treatment outcomes in children: lessons learned from the ZENITH trial in Zimbabwe. Health Policy Plan. 2018 Apr 1;33(3):328–34.

20. Dziva Chikwari C, Simms V, Busza J, Dauya E, Bandason T, Chonzi P, et al. Community health worker support to improve HIV treatment outcomes for older children and adolescents in Zimbabwe: a process evaluation of the ZENITH trial. Implement Sci. 2018 May 23;13:70.

21. Hayward SE, Vanqa N, Makanda G, Tisile P, Ngwatyu L, Foster I, et al. “As a patient I do not belong to the clinic, I belong to the community.” Co-developing a multi-level, person-centred tuberculosis stigma intervention in Cape Town, South Africa. Res Sq. 2024 Feb 8;rs.3.rs-3921970.

22. Ferrand RA, Simms V, Dauya E, Bandason T, Mchugh G, Mujuru H, et al. The effect of community-based support for caregivers on the risk of virological failure in children and adolescents with HIV in Harare, Zimbabwe (ZENITH): an open-label, randomised controlled trial. Lancet Child Adolesc Health. 2017 Nov;1(3):175–83.

23. Goudge J, de Kadt J, Babalola O, Muteba M, Tseng YH, Malatji H, et al. Household coverage, quality and costs of care provided by community health worker teams and the determining factors: findings from a mixed methods study in South Africa. BMJ Open. 2020 Aug 20;10(8):e035578.

24. Viljoen L, Bond VA, Reynolds LJ, Mubekapi‐Musadaidzwa C, Baloyi D, Ndubani R, et al. Universal HIV testing and treatment and HIV stigma reduction: a comparative thematic analysis of qualitative data from the HPTN 071 (PopART) trial in South Africa and Zambia. Sociol Health Illn. 2021;43(1):167–85.

25. Adesoro O, Oresanya O, Counihan H, Hamade P, Eguavon D, Emebo C, et al. A feasibility study to assess non-clinical community health workers’ capacity to use simplified protocols and tools to treat severe acute malnutrition in Niger state Nigeria. BMC Health Services Research. 2021 Oct 15;21(1):1102.

26. Enguita-Fernàndez C, Alonso Y, Lusengi W, Mayembe A, Manun’Ebo MF, Ranaivontiavina S, et al. Trust, community health workers and delivery of intermittent preventive treatment of malaria in pregnancy: a comparative qualitative analysis of four sub-Saharan countries. Global Public Health. 2021 Dec 2;16(12):1889–903.

27. Ndaba T, Taylor M, Mabaso M. Training and Evaluation of Community Health Workers (CHWs): Towards Improving Maternal and Newborn Survival in an Urban Setting in KwaZulu-Natal, South Africa. The Open Public Health Journal [Internet]. 2019 Oct 29 [cited 2024 Apr 3];12(1). Available from: https://openpublichealthjournal.com/VOLUME/12/PAGE/406/

28. Tseng YH, Griffiths F, de Kadt J, Nxumalo N, Rwafa T, Malatji H, et al. Integrating community health workers into the formal health system to improve performance: a qualitative study on the role of on-site supervision in the South African programme. BMJ Open. 2019 Feb 27;9(2):e022186.

29. Wanduru P, Tetui M, Tuhebwe D, Ediau M, Okuga M, Nalwadda C, et al. The performance of community health workers in the management of multiple childhood infectious diseases in Lira, northern Uganda – a mixed methods cross-sectional study. Glob Health Action. 2016 Nov 22;9:10.3402/gha.v9.33194.

30. Razafinjato B, Rakotonirina L, Cordier LF, Rasoarivao A, Andrianomenjanahary M, Marovavy L, et al. Evaluation of a novel approach to community health care delivery in Ifanadiana District, Madagascar. PLOS Glob Public Health. 2024;4(3):e0002888.

31. Klingberg S, van Sluijs EMF, Jong ST, Draper CE. Can public sector community health workers deliver a nurturing care intervention in South Africa? The Amagugu Asakhula feasibility study. Pilot and Feasibility Studies. 2021 Feb 27;7(1):60.

32. Feldhaus I, Silverman M, LeFevre AE, Mpembeni R, Mosha I, Chitama D, et al. Equally able, but unequally accepted: Gender differentials and experiences of community health volunteers promoting maternal, newborn, and child health in Morogoro Region, Tanzania. International Journal for Equity in Health. 2015 Aug 25;14(1):70.

33. Mulubwa C, Hurtig AK, Zulu JM, Michelo C, Sandøy IF, Goicolea I. Can sexual health interventions make community-based health systems more responsive to adolescents? A realist informed study in rural Zambia. Reprod Health. 2020 Jan 8;17(1):1.

34. Tinago CB, Frongillo EA, Warren AM, Chitiyo V, Jackson TN, Cifarelli AK, et al. Testing the Effectiveness of a Community-Based Peer Support Intervention to Mitigate Social Isolation and Stigma of Adolescent Motherhood in Zimbabwe. Matern Child Health J. 2024 Apr;28(4):657–66.

35. Lindsay BR, Mwango L, Toeque MG, Malupande SL, Nkhuwa E, Moonga CN, et al. Peer community health workers improve HIV testing and ART linkage among key populations in Zambia: retrospective observational results from the Z-CHECK project, 2019–2020. Journal of the International AIDS Society. 2022;25(11):e26030.

36. Kletter M, Harris B, Connolly E, Namathanga C, Nhlema B, Makungwa H, et al. Mixed method evaluation of a learning from excellence programme for community health workers in Neno, Malawi. BMC Health Serv Res. 2024 Mar 19;24(1):355.
